# Selected arylsulphonyl pyrazole derivatives as potential Chk1 kinase ligands—computational investigations

**DOI:** 10.1007/s00894-020-04407-3

**Published:** 2020-05-18

**Authors:** Kornelia Czaja, Jacek Kujawski, Karol Kamel, Marek K. Bernard

**Affiliations:** 1 Chair and Department of Organic Chemistry, Faculty of Pharmacy, Poznan University of Medical Sciences, ul. Grunwaldzka 6, 60-780 Poznan, Poland; 2 Institute of Bioorganic Chemistry, Polish Academy of Sciences, ul. Noskowskiego 12/14, 61-704 Poznan, Poland

**Keywords:** Azoles, Kinases, Checkpoint kinase 1, DFT calculations, Docking, Molecular dynamics, Hydrogen bond

## Abstract

Protein kinases control diversity of biochemical processes in human organism. Checkpoint 1 kinase (Chk1) is an important element of the checkpoint signalling pathways and is responsible for DNA damage repair. Hence, this kinase plays an essential role in cancer cells survival and has become an important target for anticancer agents. Our previous investigations showed that some arylsulphonyl indazole derivatives displayed anticancer effect in vitro. In the present study, in order to verify possibility of interactions of pyrazole and indazole derivatives with Chk1, we focused on the docking of selected tosyl derivatives of indazole and condensed pyrazole **1**–**7** to the Chk1 pocket, analysis of interactions involving optimized ligand–protein system using DFT formalism, and estimation of the interaction enthalpy of the ligand–protein complex by applying the PM7 method. The estimation of binding affinity seems to indicate that the indazole 5-substituted with 3,5-dimethylpyrazole **4** and condensed pyrazoloquinoline derivative **7** fit the best to the Chk1-binding pocket. The values of the energy of interaction, i.e. the enthalpy change (Δ*H*_int_), were between − 85.06 and − 124.04 kcal mol^−1^ for the optimized ligand–Chk1 complexes. The relaxation of the ligands within the complexes azole–protein as well as the distribution of hydrogen contacts between the ligands and kinase pocket amino acids was also analysed using molecular dynamics as a supporting method.

**Electronic supplementary material:**

The online version of this article (10.1007/s00894-020-04407-3) contains supplementary material, which is available to authorized users.

**Graphical Abstract:**
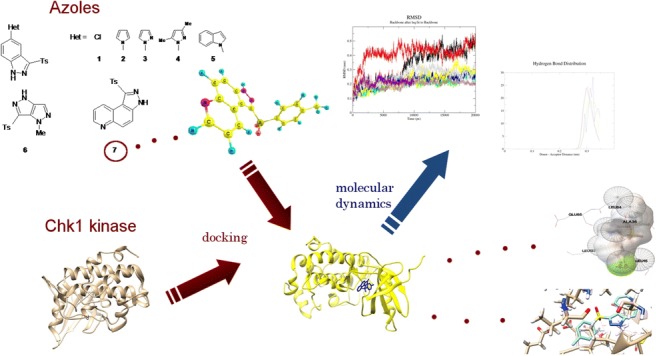
Presentation of methods used to describe the interactions between arylsulphonyl pyrazole derivatives and Chk1 kinase

## Introduction

Chemotherapy is one of the major treatments of cancer and often relies on DNA damage of fast proliferating cancer cells. The cancer cell response to such damage (DNA damage response, DDR) is activation of the S- and G_2_/M-Phase checkpoints that leads to effective repair of DNA and cell survival. The above checkpoints are controlled by checkpoint kinase 1 (Chk1), a serine/threonine kinase with self-protection function [1]. Tumour cells have developed a strong dependence on Chk1 for survival, so this kinase can be a promising target for anticancer agents. Inhibition of Chk1 results in the checkpoint abrogation and suppression of DNA repair. Therefore, the Chk1 inhibitors can increase the therapeutic effect of DNA-targeting anticancer agents and are typically used in combination with radiotherapy or DNA-damaging drugs [2].

Overexpression of Chk1 has been found to relate to pulmonary arterial hypertension (PAH), a progressive vascular remodelling of distal arteries, resulting in severe elevation of pulmonary artery pressure [3]. It has been shown that smooth muscle cells of these arteries exhibit cancer-like properties, i.e. fast proliferation and resistance to apoptosis. Hence, Chk1 might be an attractive target for the therapy of patients with PAH.

Chk1 is also involved in DNA repair by targeting repair kinases, like DNA-pyruvate kinase (DNA-PK), as well as some repair pathways, e.g. BRCA-mediated DNA repair [1]. Moreover, the ATR/Chk1 signalling pathway suppresses both a caspase-3-dependent and caspase-2-dependent apoptotic response; thus, there is supposedly a direct link between Chk1 and apoptosis [1].

Pyrazole, indazole, and condensed pyrazole derivatives constitute an important class of kinase inhibitors [4–12] including Chk1 blocking agents [6, 13, 14]. Our previous investigations showed that some indazole derivatives with an arylsulphonyl substituent at the position 3 of indazole displayed anticancer effect against the colon cancer cell line HT29 as well as breast cancer cell lines MCF7 and MDA-MB-231 [15, 16]. These derivatives can interact with magnesium ions, important for carcinogenesis [17, 18], as well as with several amino acids, present in the kinases binding site [19]. We suggested that the activity mechanism might involve either stimulation of proapoptotic proteins or inhibition of signalling pathways proteins, but we were unable to specify the target. To verify the above hypotheses, we decided to study interactions of indazole derivatives **1**–**7** with Chk1. The study focused on the docking of selected tosyl derivatives of indazole and condensed pyrazole to the Chk1 pocket with the use of *AutoDock Vina* suite, analysis of interactions involving optimized ligand–protein system with the help of DFT formalism, and estimation of the interaction enthalpy of the ligand–protein complex (PM7 method). For the analysis of the ligand relaxation within the azole–protein complexes, we used the molecular dynamics method only as a supporting technique.

Considering the potential affinity of pyrazole and indazole derivatives to kinases, we decided to investigate the interactions of azoles **1**–**7** with Chk1 using a Protein Data Bank deposit 2e9n.pdb [20, 21] in complex with A767085 ligand.

The clinically approved kinase inhibitors bind to the catalytic kinase domain—the ATP-binding site. All protein kinases, including Chk1, share the same catalytic domain that consists of an N-terminal lobe, constructed of a five-stranded β-sheet and a single α-helix, and a C-terminal lobe, mainly α-helical [6, 7]. The ATP-binding site forms a cleft between these two lobes and is composed of five regions, important for small molecule inhibitor binding, namely a linker (hinge) region for adenine, ribose pocket, phosphate binding loop (P-loop)-catalytic aspartate region, back hydrophobic (water) pocket, and front specificity pocket.

The linker region, a short, mostly hydrophobic, strand connecting C and N lobes, interacts with the adenine ring of ATP through the key site residues, i.e. glutamic acid E85, tyrosine Y86, and cysteine C87 (the numbering refers to the Chk1 sequence).

The P-loop interacts with the phosphate group of ATP through a glycine rich motif. The catalytic aspartate fragment at the active site gate contains a conserved Asp-Ph-Gly (DFG) motif at its N-terminal edge. The DFG motif adopts normally two conformations, namely DFG-in and DFG-out. In the first conformation, the aspartic acid side chain Asp148 is directed towards the active site and coordinates magnesium. As this orientation allows catalysis to proceed, it is called the active conformation, as opposed to the inactive DFG-out conformation in which the Asp148 side chain is pointed away from the active site. Most inhibitors interact with the active DFG-in orientation.

The ribose pocket in the neighbourhood of the linker region contains glutamic acid E91 that forms important contacts with the ribose hydroxylic groups. The back hydrophobic pocket is usually occupied by water molecules. The entry to this pocket is composed of the gatekeeper residue L84. The front specificity pocket is a relatively small hydrophobic region between the linker site and a hydrophilic, solvent-exposed sector of the protein [6].

## Computational methods

**Scheme 1:**
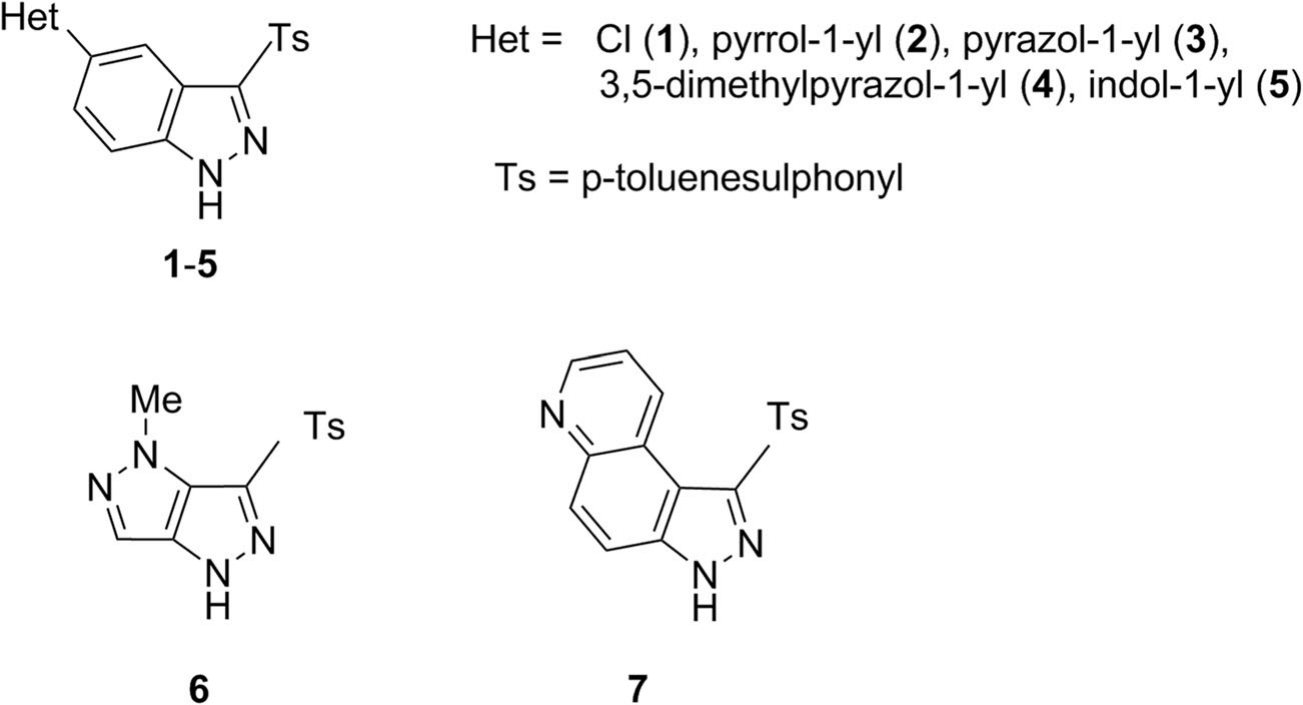
The investigated azoles as potential Chk1 kinase ligands

For the initial preparation of the analysed ligands, we obtained 1000 conformations of azoles **1**–**7** (Scheme 1) using the *Gabedit 2.4.7* program [22]. The following parameters were applied for energy minimization: heating—0.5–1000 ps, 0–1000 K, and Andersen thermostat [23] for conformation creation and the Verlet velocity algorithm for MD Trajectory [24]. For the conjugate gradient, we applied the Fletcher–Reeves method with maximum line searches of 25 [25]. In the next step, we employed minimization with molecular mechanics (MM), Amber force field with terms for the bond stretch, angle bend, torsion, nonbonded, and H-bonded. Then, all the resulting conformations were optimized with PM7 (*Mopac 2016*) [26, 27]. Each of the most energetically stable conformers of hetarenes **1**–**7**, i.e. with the lowest final heat of formation (HOF), was refined using density functional theory formalism [28] in the gaseous phase. The DFT calculations were executed, and geometries of each previously pre-optimized conformers of **1**–**7** (Scheme 1) were further refined using the *Gaussian 16 A.03* program [29] at the B3LYP/6-31G(d,p) level of theory.

**Fig. 1:**
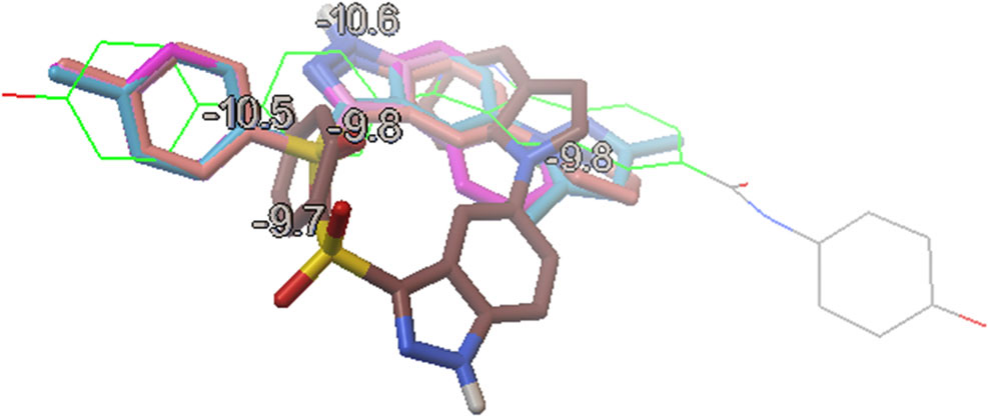
Superimposition of docked azoles **1**–**7** (1st poses, *MGLTools 1-5-6* program)

The human Chk1 kinase protein in complex with the A767085 ligand, acquired from the Protein Data Bank base (PDB entry: 2e9n.pdb), was selected as a biological target [20, 21]. An initial target for further optimization was prepared by removing the internal A767085 ligand, and all water molecules from the 2e9n.pdb file but the internal coordinates were kept unchanged. The genetic algorithm (GA) method implemented in the *AutoDock Vina* program [30] was employed to provide the appropriate binding orientations and conformations of the compounds in the Chk1-binding pocket. Polar hydrogen atoms were added, and partial charges were assigned to the protein. Then, the internal ligand was replaced by the optimized structure hetarenes **1**–**7**, and additionally, the residues were saturated with hydrogen atoms (an example of configuration file used for docking protocol of azole **1** is given in Table S1 in the Supplementary material; Cartesian coordinates of the lowest energy poses of all docked azoles **1**–**7** are given in Figure S1 in the Supplementary material). A grid box of 10-Å size (centre _x = − 1.0, centre_y = 10.0, centre_z = − 19.0) was defined to carry out the docking simulation. The outputs (*.*pdbqt* files) after docking procedure were visualized with the *Chimera 1.13.1* package [31]. The projections of the first poses of azoles **1**–**7** docked to the Chk1 pocket (Fig. 1) were visualized with *MGLTools 1-5-6* program [32]. The *Chemcraft 1.7* software was utilized for the visualization of all poses shown in Figure S1 [33]. For the semiempirical calculations with the use of PM7 method [34], we employed the *Mopac 2016* software [26]. For the initial optimization of hydrogen atoms, we used keywords “PM7 XYZ GNORM=5 T=14D DUMP=1200 NOOPT OPT-H MOZYME”; then, we carried out the refinement of complexes using “PM7 XYZ GNORM=1 T=14D DUMP=1200 MOZYME” keywords. For the molecular dynamics (MD) calculations, the *GROMACS 2016.4* [35, 36] was employed to simulate the solvated complexes. The Amber99SB-ILDN force field [37] was used to parameterize the protein and counter ions. The general GAFF force field [38] was utilized to represent the ligands and their topology with the help of *Topolbuild 1.2.1* [36]. Finally, the complexes were inserted into the cubic water boxes using the TIP3P water model [39] (10 × 10 × 10 nm). The soluble complex consisted of one molecule of Chk1 kinase, one ligand molecule, approximately 31,397 water molecules, and about six Na^+^ ions depending on the charge of the ligand. The soluble complexes were first minimized using the steepest descent scheme. Then, the minimized configurations were relaxed in NVT and NPT ensembles with 500-ps MD length per simulations. The complexes were restrained by NVT simulations using a small harmonic force. For the complexes free of restraints, we adopted NPT MD simulations. The relaxed system was then used as an initial conformation for 20-ns MD simulations. The time step used throughout the MD calculations was 2 fs.

## Results and discussion

### Ligand docking

Indazole derivatives **1**–**5**, as well as condensed pyrazoles **6** and **7**, were initially optimized by molecular mechanics; then, a series of 1000 conformers for each azole derivatives were further refined by the semiempirical method PM7. Next, for each of the azole derivatives **1**–**7**, a conformer with the smallest heat of formation (HOF) was selected and optimized in the SCF procedure (DFT method, B3LYP functional, *Gaussian 16 A.03* program [29]). The optimized ligands **1**–**7** were docked to the 2e9n.pdb protein using the *AutoDock Vina* suite [30]. Nine poses were obtained for each of azole **1**–**7** from which the first poses (Fig. 1; Figures S1–S22, Supplementary material) have the lowest negative value of binding affinity (Table S1, Supplementary material). It should be noted that azoles **1**–**4** and **7** adopt similar docking poses, whereas for azole **6**, only the tosyl group assumes a similar orientation in the binding site. Compound **5** differs from the remaining azoles—its first pose is not superimposable on the poses for azoles **1**–**4** and **6**–**7**.

Next, the molecular electrostatic potential (MEP) was determined by the B3LYP/6-311++G(2d,3p) approach for the conformers of azoles **1**–**7** (1st poses) with geometry previously optimized at B3LYP/6-31G(d,p) level of theory in gaseous phase (*Gaussian 16 A.03* program [29], Figures S1–S7, Supplementary material). In our investigations, involving the multilevel approach to the conformational rotamers search, the results were refined using a basis set enriched with the higher-level polarization functions.

The obtained results show that the pyrrolic nitrogen of the pyrazole ring, an H-bond donor, and the electron-withdrawing tosyl substituent are the most important for the azole–protein interactions. Moreover, the pyridinic nitrogen of the pyrazole ring as well as the same type of atom in the quinoline ring (compound **7**) may be of significance for each interaction. However, the contribution of these heteroatoms to the interaction strength with polar amino acids in the kinase pocket can particularly be determined by conformational factors. The stereochemical factors influence also contacts and interaction energy of the dimeric heterocycles containing an additional pyrrole, pyrazole, or indole ring. Crucial differences in the geometry of docked conformations can be expected for the fused azoles **6** and **7** as well.

**Fig. 2:**
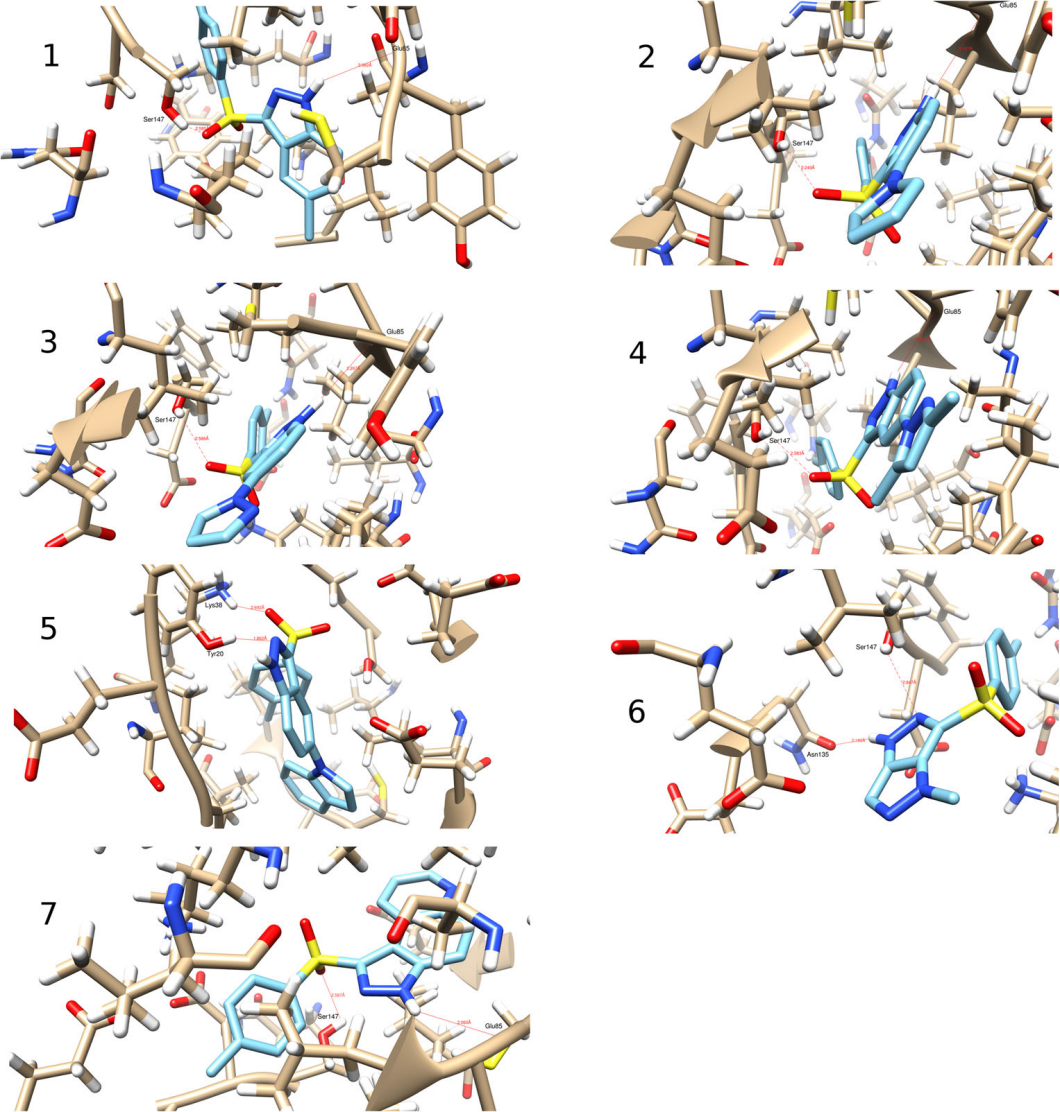
Docking poses of azoles **1**–**7** (1st poses)

Fitting of the first poses of azoles **1**–**7** in the Chk1 domain resulted in the formation of several hydrogen bonds between the ligands and the kinase amino acids (Fig. 2; Table S1, Figures S8–S15, Supplementary material). Apart from agents **5** and **6**, all other indazole derivatives form an H-bond between the pyrrolic nitrogen atoms and the carbonyl of the backbone Glu85. These results generally agree with those obtained in the previous studies, e.g. the interactions of carbazole NH atom with Glu85 [40] albeit the NH^…^O=CGlu85 contact for indazole derivatives seems to be shorter and stronger than those reported for dihydroindeno[2,3-*c*]pyrazoles, i.e. 2.03–2.267 vs 2.7 Å [20].

Let us now consider the role of the sulphone group for the ligand–Chk1 interactions. The sulphone or sulphonamide functionality is frequently present in the anticancer molecules where they act as hydrogen bond acceptors from amino acids with pendent amino or hydroxy groups often in the kinases domains [41–43]. Moreover, the sulphone functionality often works as an important spacer for kinase inhibitors [44].

Our studies have shown that the sulphone group in all azole derivatives, except indole **5**, form a hydrogen bond with the hydroxy group of Ser147, although for compound **6**, this polar interaction is of a long-range nature.

Another hydrogen bond is established between nitrogen N2 of the pyrazole ring in compound **6** and the hydroxy group of Ser147. The HB distance was observed to be shorter than 3 Å, thus within the limits for fairly strong hydrogen bonding. Such values agree with the previously reported HB for the kinase ligands containing pyrazole system [45, 46].

The major hydrogen bonding for ligand **5** is formed between the indazole nitrogen N2 and hydroxy group of Tyr20, whereas the interaction involving the sulphonyl group as an acceptor and hydrogen donating ammonium group of Lys38 is considerably weaker. This docking result agrees with the reported observation concerning interactions of Tyr20 or Lys38 with heterocyclic nitrogen atoms [45, 47, 48]. We also found that the hydrogen bond between the pyrrolic nitrogen NH and carbonyl group of Asn135 significantly stabilized ligand **6** in the kinase domain. Additional support for this finding comes from the literature data [4].

The HBs and hydrophobic interactions of docked azoles **1**–**7** with the kinase Chk1 pocket amino acids are given in Figures S16–S22 (Supplementary material). Moreover, π–π stacking interactions involving Tyr89 and ligands **1**–**4** and **7** as well as π–cation interactions involving Tyr86 and ligands **1**–**7** were observed for the best poses.

The above discussion leads to a conclusion that the 3-tosylindazole ligands with chlorine or small molecular azole substituent on position 5 of indazole form similar overlapping poses in the kinase Chk1 pocket. On the other hand, the presence of a larger substituent like indole (compound **5**), or an entirely different heterocyclic system like the pyrazolopyrazole condensed ring (compound **6**), results in a different pose and dissimilar distribution of hydrogen bonding network in the enzyme cavity.

### Optimization of ligands and residues involved in hydrogen bond formation

To further explore the geometry of docked ligands **1**–**7** (the best poses) in the Chk1-binding pocket and their interactions with the pocket amino acids, we decide to apply the DFT formalism and the B3LYP/6-31G(d,p) approach. To this end, we optimized only the ligands (Table 1; Cartesian coordinates in Supplementary material) or, additionally, the amino acid functionalities that form hydrogen contacts with the docked ligand (Table 2), while the remaining amino acids were treated as frozen. The computations were limited to a 4-Å sphere around the ligand. Furthermore, the C- and N-terminal amino acids were treated as close-shell systems, i.e. the carboxyl and amino groups were unionized. Analogously to the docking procedure, the protein coordinates were frozen for the calculations of energy changes.

**Table 1:** Ligand-amino acid distances (Å) between azoles **1**–**7** and amino acid residues within a 4-Å sphere around the ligand after ligand optimization with B3LYP/6-31G(d,p) method; RMSD_complex_ = 0.351 (**1**), 0.382 (**2**), 0.505 (**3**), 0.485 (**4**), 0.470 (**5**), 0.743 (**6**), 0.893 (**7**) Å, respectively; the corresponding contact distances for the ligands docked to 2e9n.pdbqt were given in brackets

Contacts	Distances calculated for optimized azoles **1**–**7**						
	**1**	**2**	**3**	**4**	**5**	**6**	**7**
N-H^…^O=C_Glu85_	2.841	2.854	3.077	2.826	☓	☓	2.630
SO_2_^…^H-O_Ser147_	2.517	2.500	3.266	2.817	☓	☓	2.961
N2_indol_^…^O-H_Tyr20_	☓	☓	☓	☓	3.063	☓	☓
N-H^…^OOC_Asp148_	☓	☓	☓	☓	3.787 (7.115)	☓	☓
N-H^…^O_Ser147_	☓	☓	☓	☓	☓	3.514	☓
N-H^…^O_Glu17_	☓	☓	☓	☓	3.787 (3.309)	☓	☓

**Table 2:** Ligand-amino acid distances (Å) between azoles **1**–**7** and amino acid residues within a 4-Å sphere around the ligand after ligand optimization with B3LYP/6-31G(d,p) method; RMSD_complex_ = 0.337 (**1**), 0.368 (**2**), 0.423 (**3**), 0.484 (**4**), 0.337 (**5**), 0.748 (**6**), 1.070 (**7**) Å; the contacts resulted from the docking procedure are given in brackets

Contacts	Distances calculated for optimized azoles **1**–**7**						
	**1**	**2**	**3**	**4**	**5**	**6**	**7**
N-H^…^O=C_Glu85_	2.866	2.926	2.661	2.871	☓	☓	2.654
SO_2_^…^H-O_Ser147_	2.644	2.593	3.377	2.791	☓	☓	2.846
SO_2_^…^H-N_Lys38_	☓	☓	☓	☓	3.060	☓	☓
N2_indol_^…^O-H_Tyr20_	☓	☓	☓	☓	2.402	☓	☓
N-H^…^OOC_Asp148_	☓	☓	☓	☓	3.330 (7.115)	☓	☓
N-H^…^O_Ser147_	☓	☓	☓	☓	☓	3.503	☓
N-H^…^O_Glu17_	☓	☓	☓	☓	3.330 (3.309)	☓	☓

The HB distances NH^…^O=CGlu85 for ligands **1**–**7** were elongated in the geometry optimization by DFT approach in relation to the docking results. For these contacts, the elongation was the most noticeable for pyrazole **3** (Δ = 0.81 Å) and the least significant for quinoline **7** (Δ = 0.57 Å), whereas for azoles **1**, **2**, and **4**, this lengthening was of a similar value (0.75–0.79 Å).

The H-bond distance SO_2_^…^HOSer147 was significantly elongated for pyrazole derivative **3** (Δ = 0.68 Å) and the least considerably lengthened for chlorine derivative **1** (Δ = 0.068 Å) while for the remaining azoles, this distance was elongated of 0.251 (**2**), 0.234 (**4**), or 0.374 Å (**7**).

The above data suggest that the stereoelectronic properties of Glu85 that influence energy and geometry changes of the optimized ligands were the most significant for azoles **1**–**4** and **7**. This effect involving Ser147 was negligible for azole **1**. The oxygen atom of the Ser 147 hydroxy group was closer to the hydrogen atom of pyrrolic NH in azole **6,** but still, it was a weak long-range interaction. The bond OH^…^N-pyridinic atom (ligand **6**) underwent a significant elongation during the refinement; thus, we regarded this bond as a negligible contact. Although the NH^…^OOCAsp148 contact involving indazole **5** was shortened, it was still within the long-range interactions. The polar interaction of pyrrolic NH^…^OGlu17 involving the same ligand **5** was considerably elongated from 3.309 to 3.787 Å in comparison with the docking results. If we consider in the optimization the residual functionalities of the amino acids involved in HBs together with the ligand geometry, this would affect the contacts arrangement (Table 2) in the kinase pocket. Similarly to the previous model, all contacts were elongated.

For the NH^…^O=CGlu85 interaction, the largest change was observed for 3,5-dimethylpyrazole derivative **4** (Δ = 0.840 Å), whereas the smallest alteration was noticed for pyrazole derivative **3** (Δ = 0.394 Å). For the remaining azoles **1**, **2**, and **7**, this contact was stretched out of 0.784, 0.677, and 0.594 Å, respectively.

The strongest elongation effect of the SO_2_^…^HOSer147 contact was observed for pyrazole **3** (Δ = 0.791 Å), while the weakest effect was detected for chlorine derivative **1** (Δ = 0.059 Å), thus similarly to the previous interaction model. This HB was lengthened of 0.344, 0.208, or 0.259 Å for azoles **2**, **4**, or **7**, respectively.

Analogously to the above model, the stereoelectronic impact of Glu85 upon the geometry changes of the optimized ligand was significant for azoles **1**–**4** and **7**. However, this effect concerning Ser147 was negligible for azole **1**.

The distance between the oxygen atom of Ser147 and the pyrrolic nitrogen atom of condensed azole **6** (3.503 Å) was within the limits of weak interactions. The contacts involving the pyridinic nitrogen of azole **6** and Ser147 hydroxy group as well as the Asp148 carboxyl and indazole **5** pyridinic nitrogen were practically imperceptible. However, the hydrogen bonding including the pyrrolic nitrogen in ligand **5** and carboxyl oxygen of Glu17 was almost unchanged in comparison with the docking result: 3.330 Å vs 3.309 Å, respectively (Table 2).

Next, we applied the semiempirical method PM7 for verification of the interactions between azole ligands **1**–**7** and the amino acids present in the kinase 2e9n.pdb pocket. The optimization was carried out for the functional amino acid groups that could form contacts with the best poses of ligands **1**–**7** within a 4-Å sphere leaving other coordinates of the kinase frozen. Then, we inspected distribution of the ligand–amino acid contacts in the optimized complexes (Table 3) and compared them with the previously obtained docking data (Table S1, Supplementary material). The ligand geometry in such optimized complexes did not differ from the corresponding best poses of azoles **1**–**7** obtained after docking procedure to the Chk1 pocket. The root mean square deviation (RMSD) value was 0.13–0.16 Å for the protein–ligand complexes **1**–**4**, **6**, and **7**, while for indole derivative **5**, it equalled 0.30 Å. In comparison with the docking results, the contacts involving pyrrolic atom NH in ligands **2**–**4** were virtually unchanged, whereas for chlorine and quinoline derivatives **1** and **7**, these bondings considerably weakened and were beyond the strong interactions (*d* ≥ 2.2 Å). This seems to indicate that the NH^…^O=CGlu85 contact is important for the structure of complexes involving ligands **2**–**4** but less significant for the other complexes. On the other hand, the interaction between the Ser147 hydroxy group and azoles sulphonyl function seems to be unimportant because it disappears upon computations. The contact between the Ser147 hydroxy group and pyridinic nitrogen atom N2 of the pyrazole moiety was elongated significantly for condensed azole **6**, but the contact NH^…^O=CAsn135 practically did not reshape in comparison with the docking results.

**Table 3:** Ligand-amino acid distances (Å) between azoles **1**–**7** and residues within a 4-Å sphere around the ligand after ligand optimization with PM7 method; RMSD_complex_ = 0.130 (**1**), 0.127 (**2**), 0.138 (**3**), 0.119 (**4**), 0.292 (**5**), 0.143 (**6**), 0.157 (**7**) Å, respectively; the contacts resulted from the docking procedure are given in brackets

Contacts	Distances calculated for optimized azoles **1**–**7**						
	**1**	**2**	**3**	**4**	**5**	**6**	**7**
N-H^…^O=C_Glu85_	2.432	1.974	2.060	2.047	☓	☓	2.493
SO_2_^…^H-O_Ser147_	5.774	5.409	5.371	5.089	☓	☓	5.386
SO_2_^…^H-N_Lys38_	2.947 (4.037)	3.478 (4.054)	3.352 (3.937)	3.492 (4.053)	1.699	1.591 (4.303)	2.953 (3.970)
N2_indol_^…^H-N_Lys38_	☓	☓	☓	☓	3.157 (5.320)	☓	☓
N2_indol_^…^O-H_Tyr20_	☓	☓	☓	☓	5.624	☓	☓
N-H^…^OOC_Asp148_	☓	☓	☓	☓	1.861 (7.115)	☓	☓
N-H^…^O=C_Asn135_	☓	☓	☓	☓	☓	2.584	☓
N2^…^H-O_Ser147_	☓	☓	☓	☓	☓	3.556	☓

The sulphonyl group on azoles **1**–**4** and **6**–**7** participated in a long-range polar interaction with the ammonium group of Lys38 or even could form a hydrogen bond (azole **6**) with this function as opposed to the docking results. A complete loss of interaction was observed for the pyrazole pyridinic nitrogen in ligand **5** and Tyr20 hydroxy group. However, a new hydrogen bond was spotted between the pyrazole pyridinic nitrogen and Lys38 ammonium group. The above described contact involving indazole pyrrolic nitrogen (indazole **5**) and Glu17 carboxyl disappeared.

### Estimation of interaction energy

In the next step, we focused on the assessment of enthalpy changes of the interactions of azole ligands **1**–**7** (Δ*H*_int_) in the Chk1 pocket. In this evaluation, we considered values of HOF under standard conditions using *Mopac 2016* program and its implemented module, Mozyme [26]. To study the interactions between ligand and kinase pocket, the binding sphere was limited to 4 Å from the best pose. The pocket amino acids were correctly protonated, and the C- and N-terminal amino acids were ionized to obtain COO^−^ or NH_3_^+^. Then, the hydrogen atoms of ligand–protein complex were optimized as well as the ligand environment leaving the COO^−^ or NH_3_^+^ groups frozen. The resulted hydrogen bonds and polar interactions in such optimized complexes are shown in Table 4.

**Table 4:** Ligand-amino acid distances (including hydrogen bonds) (Å) between azoles **1**–**7** and residues within a 4-Å sphere around the ligand after ligand optimization with PM7 method; RMSD_complex_ = 0.604 (**1**), 1.085 (**2**), 0.622 (**3**), 1.438 (**4**), 0.790 (**5**), 0.058 (**6**), 0.689 (**7**) Å, respectively; the contacts resulted from the docking procedure are given in brackets

Contacts	Contacts length calculated for optimized azoles **1**–**7**						
	**1**	**2**	**3**	**4**	**5**	**6**	**7**
N-H^…^O=C_Glu85_	1.841	1.913	1.855	1.793	☓	☓	3.244
SO_2_^…^H-O_Ser147_	2.919	4.406	3.257	3.754	☓	☓	1.792
SO_2_^…^H-N_Lys38_	☓	☓	☓	☓	2.475	☓	4.155 (3.970)
N2_indol_^…^H-N_Lys38_	☓	☓	☓	☓	4.289 (5.320)	☓	☓
N2_indol_^…^O-H_Tyr20_	☓	☓	☓	☓	3.758	☓	☓
N-H^…^OO=C_Asp148_	☓	☓	☓	☓	4.870 (7.115)	☓	☓
N-H^…^O=C_Asn135_	☓	☓	☓	☓	☓	1.750	☓
N2^…^H-O_Ser147_	☓	☓	☓	☓	☓	4.565	☓
N-H^…^O_Glu17_	☓	☓	☓	☓	3.486 (3.309)	☓	☓

For the interaction energy calculations, we adopted an approach based on the thermodynamic cycle of Raha and Merz [34]:Δ*H*_int_ = Δ*H*_f(PL)_ − [Δ*H*_fcomplex(P)_ + Δ*H*_fcomplex(L)_], where Δ*H*_f(X)_ were the heats of formation in vacuo of the protein–ligand complex, free ligand (L) or free protein (P), and the Δ*H*_fcomplex(X)_ parameter corresponds to the enthalpy of the protein or ligand molecule in the complex conformation. An important note must be made here: this procedure does not account for such processes as desolvation of the binding pocket. However, we compare energies of the structures docked into the same binding pocket; therefore, the desolvation energy should be comparable among the **1**–**7** series.

The application of the above equation to the complexes of ligands **1**–**7** with Chk1 led to the values shown in Tables 4 and 5. These values provide evidence that 3,5-dimethylpyrazole derivative **4** and indole derivative **5** are the ligands best fitted in the Chk1 pocket. For compound **4**, this conclusion is in accordance with the docking result (Table S1, Supplementary material), whereas for compound **5**, it seems that the estimation of binding affinity by molecular modelling is rather inconclusive. Moreover, this result is consistent with the above discussed models indicating that ligand **4** forms indeed the strongest interactions with Glu85 and Ser147 in the Chk1 pocket. Noteworthy is the fact that the NH^…^O=CGlu85 contact is shortened for ligands **1**–**4** but is elongated for quinoline derivative **7** in comparison with the docking results. The ligand **1** SO_2_^…^HOSer147 distance has almost similar value, but the same contact was significantly lengthened for ligands **2**–**4** or shortened for ligand **7**. The interaction energy for azoles for azoles **2**, **3**, and **6** was on the similar level, whereas azole **1** with chlorine substituent was energetically disfavoured (Table 5). Subsequently, the SO_2_^…^HNLys38 contact was virtually unchanged for indazole **5**, whereas the pyridinic N^…^HOTyr20 interaction was markedly stretched out and weakened. The interactions concerning the pyridinic nitrogen atom of indazole **5** with Lys 38 and Asp148, described in the previous model involving PM7 optimization of the best docking pose, practically disappeared. The ligand **5** pyrrolic NH^…^O=CGlu17 contact (3.486 Å; Table 4) was comparable to the docking results (3.309 Å; Table S1, Supplementary material) but Glu17 was in the periphery of the kinase pocket. We can conclude that the share of Tyr20 in the relatively high interaction energy (Δ*H*_int_ = − 111.88 kcal mol^−1^; Table 5) is marginal. The PM7 results concerning azoles **5** and **7** differed from the estimated binding affinity obtained in the docking protocol.

**Table 5:** Calculated heats of formations (kcal mol^−1^) for free ligands (Δ*H*_fcomplex(L)_), free protein (Δ*H*_fcomplex(P)_), ligand–protein complex (Δ*H*_f(PL)_), as well as ligand–protein interaction energy (Δ*H*_int_)

Compound	HOF of ligand (Δ*H*_fcomplex(L)_)	HOF of protein (Δ*H*_fcomplex(P)_)	HOF of complex (Δ*H*_f(PL)_)	Δ*H*_int_
1	− 1.11	− 1164.89	− 1251.06	− 85.06
2	43.41	− 2046.66	− 2105.93	− 102.67
3	62.88	− 1370.37	− 1405.87	− 98.39
4	46.76	− 2130.38	− 2207.67	− 124.04
5	55.48	− 1844.07	− 1900.47	− 111.88
6	49.99	− 1673.68	− 1723.83	− 100.14
7	34.23	− 1212.30	− 1276.91	− 92.18

### Molecular dynamics calculations

**Fig. 3:**
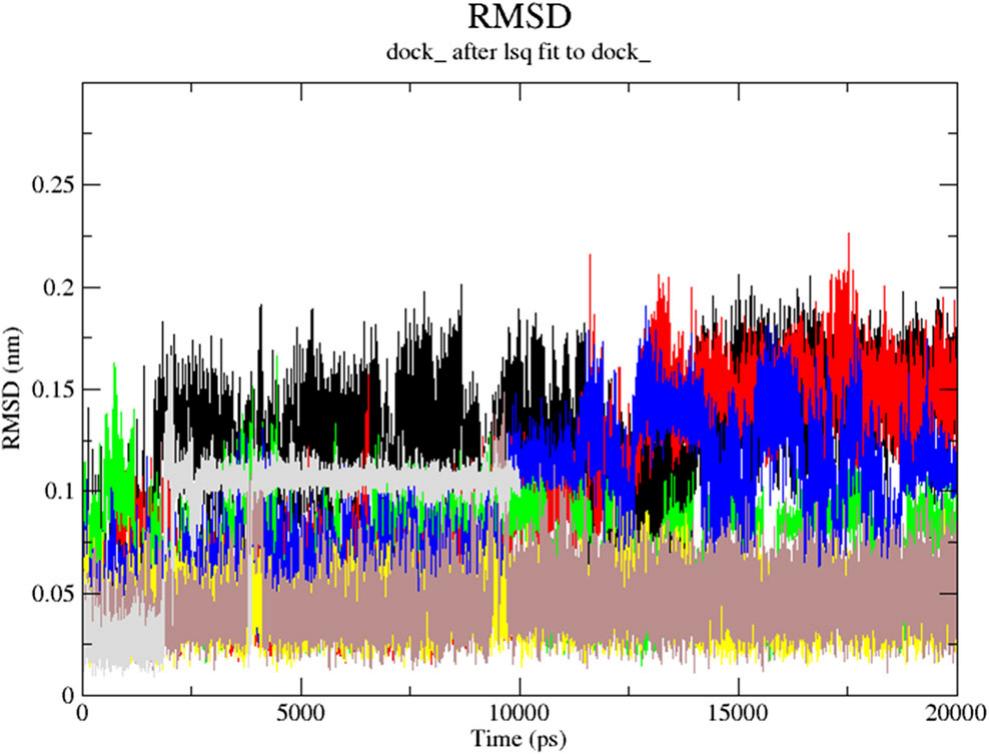
The RMSD plot for the backbone within ligand–protein complex during the productive phase calculated for free kinase (black), as well as its complex with the following: **1** (red), **2** (green), **3** (blue), **4** (yellow), **5** (grey), **6** (light grey), and **7** (purple)

Next, we applied molecular dynamics (MD) to explore stability of ligands **1**–**7** in the Chk1 pocket. For this purpose, the *GROMACS 2016.4* [35, 36] was employed to simulate the solvated ligand–protein complexes (cubic water boxes). The RMSDs of backbone atoms (with or without the presence of ligands **1**–**7**) were calculated with respect to the initial configuration. The time evolution of RMSD values of the backbone in the ligand–protein complexes is shown in Fig. 3.

The ligand RMSD plot shows that the docking poses of all azole ligands, apart from chlorine derivative **1**, are stable inside the kinase pocket, especially if we compare this plot with the RMSD simulation relative to the empty kinase pocket. Generally, ligands **2**–**7** remained the backbone stable (regarding their influence on protein) for ca 5 ns in their positions with RMSD oscillating at 1.50–2.00 Å; then, the RMSD curve moved upward and oscillated at 2.00–2.50 Å. In most cases, the RMSD values were smaller than 3.0 Å, which clearly indicates that these systems were quite stable during the last 12 ns. Examining the RMSD plot, we can conclude that apparently ligands **2** and **5** make the backbone most stable.

Hydrogen bonds are important interactions within an enzyme active site, especially these formed when an enzyme substrate reaches the transition state [49]. The phosphorylation process, characteristic for kinases, does not require strong HBs between the enzyme substrate and amino acids present in the active site.

However, HBs and short electrostatic contacts cannot be distinguished reliably by the RDF analysis. As this study was intended to initiate further computational research by us in the field of azole ligand affinity, we chose to proceed with a more detailed analysis of the hydrogen bonds along the MD trajectory.

The geometric criteria proposed for HBs indicate that the donor–acceptor distance *D* should be less than 3.5 Å and the *Ѳ* angle more than 130° [50]. However, these criteria for proteins are less restricted (*D* < 3.9 Å and *Ѳ* >  90° [51]) due to the large structural flexibility of the enzyme active site during the catalysis process. These structural alterations cause fluctuation of the hydrogen bonds array. Considering the dynamic nature of the contacts formed, we finally chose to use rather conservative values to define the occurrence of hydrogen bonding: *D* < 3.3 Å and *Ѳ* > 135°. Therefore, we focused on the occupancy parameter relative to HBs formed by azoles **1**–**7** with the above-discussed amino acids.

The occupancy parameter involving Glu85 has the following values: 12.66 (**1**), 23.72 (**2**), 14.58 (**3**), 0.11 (**4**), and 0.26% (**7**). We can conclude that the impact of Glu85 on the possible formation of hydrogen contacts is significant for azoles **1**–**3**. This matches with the previously discussed models (Tables 1, 2, 3, and 4). On the other hand, we did not observe significant interactions between Ser147 and azoles **1**–**3**, or the occupancy parameter had a small value of 0.03 or 0.04% for azoles **4** or **7**. This relatively minor role of Ser147 in the formation of HBs is confirmed by the previously obtained data shown in Tables 1, 2, 3, and 4. A similar value of the occupancy parameter (0.004%) was obtained for azole **5** and Tyr20. For azole **5** and Glu17, however, this parameter reached 9.79%. Therefore, Glu17 participates considerably in the electrostatic interaction with the NH atom of azole **5** that is in accordance with the data presented in Tables 1, 2, and 4.

For azole **6** and Ser147 or Asn135, we did not observe the occupancy parameter relative to the formation of HBs.

The half-life time for the hydrogen contacts involving azoles **1**–**4** and **7** and Glu85 was the longest in the simulation (Figure S23 in the Supplementary material). However, these contacts half-life time was shortened considerably in relation to the Ser147 interactions (Figure S24 in the Supplementary material). Therefore, Ser147 influence on the formation of HBs with azoles **4** and **7** seems to be less critical, which corresponds with the occupancy parameters and conclusions drawn from the previous models.

The corresponding half-life time involving azole **5** and Tyr20 was relatively short and did not exceed 1.45 ps (Figure S25 in the Supplementary material). Consequently, Tyr20 impact on the formation of HB with the azole **7** pyridinic nitrogen was insignificant.

In contrast to the previous case, the half-life time involving Glu17 and azole **5** was markedly longer at 21 ps (Figure S25 in the Supplementary material). Therefore, this interaction should be regarded as necessary for the ligand stabilization in the kinase pocket. Both the above conclusions concerning azole **5** follow the occupancy parameter and conclusions drawn from the previous models. A relatively high value of interaction enthalpy (Table 5) may be connected with the additional π–cation contact with Lys38.

The MD simulation for condensed pyrazole **6** did not show the formation of HBs with Ser147 and Asn135 that would fulfil the adopted cut-off criteria. This observation agrees with the occupancy parameters and data obtained from the DFT formalism and PM7 method.

## Conclusions

Checkpoint kinase 1 is a pivotal element of the checkpoint signalling involving DNA damage response. As the DDR occurs in reaction to DNA destruction by cytotoxic agents and radiation, Chk1 is a potential target for kinase inhibitors [52]. Our previous investigations showed that some indazole derivatives with a toluene sulphonyl substituent at the position 3 of indazole displayed anticancer effect against colon cancer cell line HT29 as well as breast cancer cell lines MCF7 and MDA-MB-231. In the present paper, we provided evidences that such derivatives of indazole and condensed pyrazole can represent a valuable template hit for the future Chk1 inhibitors. The molecular docking to the kinase ATP pocket has shown that the refined azole conformers **1**–**4** and **7** access the bonding region in Chk1 and form relatively strong hydrogen bonds with the key amino acids, i.e. Glu85 and Ser147. The docking has shown that that the 3-arylsulphonylindazole ligands with chlorine or small molecular azole substituent on position 5 of indazole form similar overlapping poses in the kinase Chk1 pocket. On the other hand, the presence of a larger substituent like indole (compound **5**), or an entirely different heterocyclic system like the pyrazolopyrazole condensed ring (compound **6**), results in a different pose and dissimilar distribution of hydrogen bondings in the enzyme cavity.

The above observation has been confirmed by the DFT and semiempirical PM7 methods although these computations have given somewhat inconsistent results for the interactions between the ligands and Ser147, particularly for compounds **1**, **5**, and **6**. The calculated interaction energy favours azoles **2**, **3**, **5**, and **6**, whereas the remaining azoles, particularly compound **1**, are relatively energetically disfavoured.

The molecular dynamics simulations indicate that almost all complexes ligand–kinase Chk1, apart from compound **1**, presented stable MD trajectories. We can conclude that the impact of Glu85 on the possible formation of hydrogen contacts is significant especially for azoles **1**–**3**. This matches the previously discussed models (Tables 1, 2, 3, and 4). However, the occupancy parameters for the interactions of Ser147, Tyr20, and Asn135 with ligands **1**–**7** are usually of small values (apart from azole **5** and Glu17). This may indicate that formation of hydrogen bonds involving the above amino acids is less probable.

## Electronic supplementary material

ESM 1 (DOC 41358 kb)

## Data Availability

Cartesian coordinates of all discussed ligands and adducts, MEP analysis visualization, docking input file, as well as results of docking are given in the Supplementary file. This information is available via the Internet or upon the request from the corresponding author.

## References

[1] Dai Y, Grant S (2010). New insights into checkpoint kinase 1 in the DNA damage response signaling network. Clin Cancer Res.

[2] Garrett MD, Collins I (2011). Anticancer therapy with checkpoint inhibitors: what, where and when?. Trends Pharmacol Sci.

[3] Bourgeois A, Bonnet S, Breuils-Bonnet S, Habbout K, Paradis R, Tremblay E, Lampron M-C, Orcholski ME, Potus F, Bertero T, Peterlini T, Chan SY, Norris KA, Paulin R, Provencher S, Boucherat O (2019). Inhibition of CHK 1 (checkpoint kinase 1) elicits therapeutic effects in pulmonary arterial hypertension. Arterioscler Thromb Vasc Biol.

[4] Matthews TP, Klair S, Burns S, Boxall K, Cherry M, Fisher M, Westwood IM, Walton MI, McHardy T, Cheung K-M J, Van Montfort R, Williams D, Aherne GW, Garrett MD, Reader J, Collins I (2009). Identification of inhibitors of checkpoint kinase 1 through template screening. J Med Chem.

[5] Harris PA, Stafford JA, Li R, Stafford JA (2009). Discovery of pazopanib: a pan vascular endothelial growth factor kinase inhibitor. Kinase inhibitor drugs.

[6] Matthews DJ, Gerritsen ME (2010). Targeting protein kinases for cancer therapy.

[7] Wang Q, Zorn JA, Kuriyan JA (2014) Structural atlas of kinases inhibited by clinically approved drugs. In: Shokat KM (ed) Protein kinase inhibitors in research and medicine, vol 548. Elsevier-Academic Press, Methods in Enzymology, pp 23–67 10.1016/B978-0-12-397918-6.00002-1 25399641

[8] Giraud F, Arizon F, Moreau P (2014) Advances in the synthesis and kinase inhibitory potencies of non-fused indazole derivatives. In: Attanasi OA, Neto R, Spinelli D (eds) Targets in heterocyclic systems, chemistry and properties, vol 18. Italian Society of Chemistry, pp 1–28

[9] Avendaño C, Menéndez JC (2015). Drugs that inhibit signaling pathways for tumor cell growth and proliferation: kinase inhibitors. Medicinal chemistry of anticancer drugs.

[10] Ma X, Lv X, Zhang J (2018). Exploiting polypharmacology for improving therapeutic outcome of kinase inhibitors (KIs): an update of recent medicinal chemistry efforts. Eur J Med Chem.

[11] Wang Q, Dai Y, Ji Y, Shi H, Guo Z, Chen D, Chen Y, Peng X, Gao Y, Wang X, Chen L, Jiang Y, Geng M, Shen J, Ai J, Xiong B (2019). Discovery and optimization of a series of 3-substituted indazole derivatives as multi-target kinase inhibitors for the treatment of lung squamous cell carcinoma. Eur J Med Chem.

[12] Dong J, Zhang Q, Wang Z, Huang G, Li S (2018). Recent advances in the development of indazole-based anticancer agents. Chem Med Chem.

[13] Janetka JW, Ashwell S, Zabludoff S, Lyne P (2007). Inhibitors of checkpoint kinases: from discovery to the clinic. Curr Opin Drug Discov Dev.

[14] Galal SA (2019). Checkpoint kinases inhibitors; synthesis and biological evaluation products. Acta Sci Cancer Biol.

[15] Toton E, Ignatowicz E, Bernard MK, Kujawski J, Rybczyńska M (2013). Evaluation of apoptotic activity of new condensed pyrazole derivatives. J Physiol Pharmacol.

[16] Lehmann TP, Kujawski J, Kruk J, Czaja K, Bernard MK, Jagodziński PP (2017). Cell-specific cytotoxic effect of pyrazole derivatives on breast cancer cell lines MCF7 and MDA-MB-231. J Physiol Pharmacol.

[17] Kujawski J, Doskocz M, Popielarska H, Myka A, Drabińska B, Kruk J, Bernard MK (2013). Interactions between indazole derivative and magnesium cations – NMR investigations and theoretical calculations. J Mol Struct.

[18] Czaja K, Kujawski J, Girreser U, Panek JJ, Doskocz M, Bernard MK (2016). Possible interactions between fused pyrazole derivative and magnesium ions - NMR experiments and theoretical calculations. ARKIVOC.

[19] Czaja K, Kujawski J, Jodłowska-Siewert E, Szulc P, Ratajczak T, Krygier D, Chmielewski MK, Bernard MK (2017) On the interactions of fused pyrazole derivative with selected amino acids: DFT calculations. J Chem 1–8

[20] Tong Y, Claiborne A, Stewart KD, Park C, Kovar P, Chen Z, Credo RB, Gu W-Z, Gwaltney SL, Judge RA, Zhang H, Rosenberg SH, Sham HL, Sowin TJ, Lin N-H (2007). Discovery of 1,4-dihydroindeno[1,2-c]pyrazoles as a novel class of potent and selective checkpoint kinase 1 inhibitors. Bioorg Med Chem.

[21] https://www.rcsb.org/structure/2e9n, login on 20^th^ of June 2019

[22] Allouche A-R (2011). Gabedit – a graphical user interface for computational chemistry softwares. J Comput Chem.

[23] Hünenberger PH (2005). Thermostat algorithms for molecular dynamics simulations. Adv Polym Sci.

[24] Spreiter Q, Walter M (1999). Classical molecular dynamics simulation with the Velocity Verlet algorithm at strong external magnetic fields. J Comput Phys.

[25] Cantrell JW (1969). Relation between the memory gradient method and the Fletcher-Reeves method. J Optimiz Theory App.

[26] http://openmopac.net/MOPAC2016.html, login on 20th of August 2019

[27] Stewart JPP (2013). Optimization of parameters for semiempirical methods VI: more modifications to the NDDO approximations and re-optimization of parameters. J Mol Model.

[28] Parr RG, Yang W (1994). Density-functional theory of atoms and molecules.

[29] Frisch MJ, Trucks GW, Schlegel HB, Scuseria GE, Robb MA, Cheeseman JR, Scalmani G, Barone V, Petersson GA, Nakatsuji H, Li X, Caricato M, Marenich AV, Bloino J, Janesko BG, Gomperts R, Mennucci B, Hratchian HP, Ortiz JV, Izmaylov AF, Sonnenberg JL, Williams-Young D, Ding F, Lipparini F, Egidi F, Goings J, Peng B, Petrone A, Henderson T, Ranasinghe D, Zakrzewski VG, Gao J, Rega N, Zheng G, Liang W, Hada M, Ehara M, Toyota K, Fukuda R, Hasegawa J, Ishida M, Nakajima T, Honda Y, Kitao O, Nakai H, Vreven T, Throssell K, Montgomery JJA, Peralta JE, Ogliaro F, Bearpark MJ, Heyd JJ, Brothers EN, Kudin KN, Staroverov VN, Keith TA, Kobayashi R, Normand J, Raghavachari K, Rendell AP, Burant JC, Iyengar SS, Tomasi J, Cossi M, Millam JM, Klene M, Adamo C, Cammi R, Ochterski JW, Martin RL, Morokuma K, Farkas O, Foresman JB, Fox DJ (2016). Gaussian 16, revision C.01.

[30] Trott O, Olson AJ (2010). AutoDock Vina: improving the speed and accuracy of docking with a new scoring function, efficient optimization and multithreading. J Comput Chem.

[31] Pettersen EF, Goddard TD, Huang CC, Couch GS, Greenblatt DM, Meng EC, Ferrin TE (2004). Chimera - a visualization system for exploratory research and analysis. J Comput Chem.

[32] AutoDockTools. Molecular Graphics Laboratory, Scripps Research Institute, La Jolla, California; http://www.scripps.edu/∼sanner/python/, login on 20th of June 2019

[33] http://www.chemcraftprog.com, login on 5^th^ of September 2019

[34] Raha K, Merz KM (2005). Large-scale validation of a quantum mechanics based scoring function: predicting the binding affinity and the binding mode of a diverse set of protein-ligand complexes. J Med Chem.

[35] van der Spoel D, Lindahl E, Hess B, Groenhof G, Mark AE, Berendsen HJC (2005). GROMACS: fast, flexible, and free. J Comput Chem.

[36] www.gromacs.com, login on 20th of August 2019

[37] Lindorff-Larsen K, Piana S, Palmo K, Maragakis P, Klepeis JL, Dror LO, Shaw DE (2010). Improved side-chain torsion potentials for the Amber ff99SB protein force field. Proteins.

[38] Wang J, Wolf RM, Caldwell JW, Kollman PA, Case DA (2004). Development and testing of a general amber force field. J Comput Chem.

[39] Jorgensen WL, Chandrasekhar J, Madura JD, Impey RW, Klein ML (1983). Comparison of simple potential functions for simulating liquid water. J Chem Phys.

[40] Gao X, Han L, Ren Y (2016). In silico exploration of 1,7-diazacarbazole analogs as checkpoint kinase 1 inhibitors by using 3D QSAR, molecular docking study, and molecular dynamics simulations. Molecules.

[41] Banerjee S, Norman DD, Lee SC, Parrill AL, Pham TCT, Baker DL, Tigyi GJ, Miller DD (2017). Highly potent non-carboxylic acid autotaxin inhibitors reduce melanoma metastasis and chemotherapeutic resistance of breast cancer stem cells. J Med Chem.

[42] Philp J, Lawhorn BG, Graves AP, Shewchuk L, Rivera KL, Jolivette LJ, Holt DA, Gatto JGJ, Kallander LS (2018). 4,6-Diaminopyrimidines as highly preferred troponin I-interacting kinase (TNNI3K) inhibitors. J Med Chem.

[43] Barton N, Convery M, Cooper AWJ, Down K, Hamblin JN, Inglis G, Peace S, Rowedder J, Rowland P, Taylor JA, Wellaway N (2018). Discovery of potent, efficient, and selective inhibitors of phosphoinositide 3-kinase δ through a deconstruction and regrowth approach. J Med Chem.

[44] Schnute ME, McReynolds MD, Carroll J, Chrencik J, Highkin MK, Iyanar K, Jerome G, Rains JW, Saabye M, Scholten JA, Yates M, Nagiec MM (2017). Discovery of a potent and selective sphingosine kinase 1 inhibitor through the molecular combination of chemotype-distinct screening hits. J Med Chem.

[45] Malvacio I, Cuzzolin A, Sturlese M, Vera DMA, Moyano EL, Moro S (2017). Synthesis and preliminary structure-activity relationship study of 2-aryl-2H-pyrazolo[4,3-c]quinolin-3-ones as potential checkpoint kinase 1 (Chk1) inhibitors. J Enzym Inhib Med Chem.

[46] Matthews TP, Jones AM, Collins I (2013). Structure-based design, discovery and development of checkpoint kinase inhibitors as potential anticancer therapies. Expert Opin Drug Discovery.

[47] Al-Sha’er MA, Khanfar MA, Taha MO (2015). Discovery of check point kinase 1 (Chk1) inhibitors as potential anticancer agents using ligand-based modelling and virtual screening. J Sil Vitro Pharmacol.

[48] Foloppe N, Fisher LM, Howes R, Kierstan P, Potter A, Robertson AGS, Surgenor AE (2005). Structure-based design of novel Chk1 inhibitors: insights into hydrogen bonding and protein−ligand affinity. J Med Chem.

[49] Cleland WW, Frey PA, Gerlt JA (1998). The low barrier hydrogen bond in enzymatic catalysis. J Biol Chem.

[50] Bondi A (1964). van der Waals volumes and radii. J Phys Chem.

[51] McDonald IK, Thornton JM (1994). Satisfying hydrogen bonding potential in proteins. J Mol Biol.

[52] Keränen H, Pérez-Benito L, Ciordia M, Delgado F, Steinbrecher TB, Oehlrich D, van Vlijmen HWT, Trabanco AA, Tresadern G (2017). Acylguanidine beta secretase 1 inhibitors: a combined experimental and free energy perturbation study. J Chem Theory Comput.

