# Supplementary material for: Selected arylsulphonyl pyrazole derivatives as potential Chk1 kinase ligands—computational investigations
Source: J Mol Model. 2020 May 18;26(6):144. doi: 10.1007/s00894-020-04407-3 (PMC7235069; doi:10.1007/s00894-020-04407-3)

**Supporting Information**

**Selected arylsulphonyl pyrazole derivatives as potential Check1-kinase ligands - computational investigations**

*^1^Kornelia Czaja*, ^1^Jacek Kujawski, ^2^Karol Kamel, ^1^Marek K. Bernard*

^1^Chair and Department of Organic Chemistry, Faculty of Pharmacy, Poznan University of Medical Sciences, ul. Grunwaldzka 6, 60-780 Poznań, Poland

^2^Institute of Bioorganic Chemistry, Polish Academy of Sciences, ul. Noskowskiego 12/14, 61-704 Poznań, Poland

*corresponding author: czaja.kornelia@gmail.com, phone 486185466701, fax 48618546680

Table of Contents:

[**Table S1**: Configuration file used for docking procedure of azole **1**. 3](#__RefHeading___Toc35529587)

[**Table S2**: Estimated binding affinity [kcal mol^-1^] for all poses for azoles **1**‑**7** generated during docking procedurę (results for the best poses are marked in bold). 3](#__RefHeading___Toc35529588)

[**Fig. S1** Electrostatic potential (ESP) map of docked azole **1** (1^st^ pose) calculated at the B3LYP/6-311++G(2d,3p)//B3LYP-631G(d,p) level of theory (gaseous phase); isovalue = 0.002 a.u. 4](#__RefHeading___Toc35529589)

[**Fig. S2** Electrostatic potential (ESP) map of docked azole **2** (1^st^ pose) calculated at the B3LYP/6-311++G(2d,3p)//B3LYP-631G(d,p) level of theory (gaseous phase); isovalue = 0.002 a.u. 4](#__RefHeading___Toc35529590)

[**Fig. S3** Electrostatic potential (ESP) map of docked azole **3** (1^st^ pose) calculated at the B3LYP/6-311++G(2d,3p)//B3LYP-631G(d,p) level of theory (gaseous phase); isovalue = 0.002 a.u. 5](#__RefHeading___Toc35529591)

[**Fig. S4** Electrostatic potential (ESP) map of docked azole **4** (1^st^ pose) calculated at the B3LYP/6-311++G(2d,3p)//B3LYP-631G(d,p) level of theory (gaseous phase); isovalue = 0.002 a.u. 5](#__RefHeading___Toc35529592)

[**Fig. S6** Electrostatic potential (ESP) map of docked azole **6** (1^st^ pose) calculated at the B3LYP/6-311++G(2d,3p)//B3LYP-631G(d,p) level of theory (gaseous phase); isovalue = 0.002 a.u. 6](#__RefHeading___Toc35529593)

[**Fig. S7** Electrostatic potential (ESP) map of docked azole **7** (1^st^ pose) calculated at the B3LYP/6-311++G(2d,3p)//B3LYP-631G(d,p) level of theory (gaseous phase); isovalue = 0.002 a.u. 7](#__RefHeading___Toc35529594)

[**Fig. S8**: Geometry of the first poses of azoles **1**–**7** after docking procedure to 2e9n.pdb protein and their Cartesian coordinates (charge=0, multiplicity=1): 8](#__RefHeading___Toc35529595)

[**Cartesian coordinates of azoles 1‑7 with corresponding residues around 4 Å after optimization with DFT formalism** (ligands optimization, B3LYP/6-31G(d,p) level of theory, results given in the Table 3, ***Gaussian G16.A01* software**) 17](#__RefHeading___Toc35529596)

[**Cartesian coordinates of azoles 1‑7 with corresponding residues around 4 Å after optimization with DFT formalism** (ligands and functionalities of amino acids optimization, B3LYP/6-31G(d,p) level of theory, results given in the Table 4, ***Gaussian G16.A01* software**) 71](#__RefHeading___Toc35529597)

[**Cartesian coordinates of azoles 1‑7 with corresponding residues around 4 Å after optimization with PM7 method (*Mopac 2016* software)** 125](#__RefHeading___Toc35529598)

[**Fig. S9** The first (best) pose of azole **1** docked to Chk1 binding site 178](#__RefHeading___Toc35529599)

[**Fig. S10** The first (best) pose of azole **2** docked to Chk1 binding site 178](#__RefHeading___Toc35529600)

[**Fig. S11** The first (best) pose of azole **3** docked to Chk1 binding site 179](#__RefHeading___Toc35529601)

[**Fig. S12** The first (best) pose of azole **4** docked to Chk1 binding site 179](#__RefHeading___Toc35529602)

[**Fig. S13** The first (best) pose of azole **5** docked to Chk1 binding site 180](#__RefHeading___Toc35529603)

[**Fig. S14** The first (best) pose of azole **6** docked to Chk1 binding site 180](#__RefHeading___Toc35529604)

[**Fig. S15** The first (best) pose of azole **7** docked to Chk1 binding site 181](#__RefHeading___Toc35529605)

[**Fig. S16** Close contacts within the **1**‑**2e9n** complex (HB marked with dashes, π‑cation contact with Lys38, π‑π stacking contact with Tyr86; *MGLTools 1-5-6* program) 181](#__RefHeading___Toc35529606)

[**Fig. S17** Close contacts within the **2**‑**2e9n** complex (HB marked with dashes, π‑cation contact with Lys38, π‑π stacking contact with Tyr86; *MGLTools 1-5-6* program) 182](#__RefHeading___Toc35529607)

[**Fig. S18** Close contacts within the **3**‑**2e9n** complex (HB marked with dashes, π‑cation contact with Lys38, π‑π stacking contact with Tyr86; *MGLTools 1-5-6* program) 182](#__RefHeading___Toc35529608)

[**Fig. S19** Close contacts within the **4**‑**2e9n** complex (HB marked with dashes, π‑cation contact with Lys38, π‑π stacking contact with Tyr86; *MGLTools 1-5-6* program) 183](#__RefHeading___Toc35529609)

[**Fig. S20** Close contacts within the **5**‑**2e9n** complex (*MGLTools 1-5-6* program) 183](#__RefHeading___Toc35529610)

[**Fig. S21** Close contacts within the **6**‑**2e9n** complex (HB marked with dashes, π‑cation contact with Lys38;*MGLTools 1-5-6* program) 184](#__RefHeading___Toc35529611)

[**Fig. S22** Close contacts within the **7**‑**2e9n** complex (HB marked with dashes, π‑cation contact with Lys38, π‑π stacking contact with Tyr86; *MGLTools 1-5-6* program) 184](#__RefHeading___Toc35529612)

[**Fig. S23** HBs lifetime for **1** (black), **2** (red), **3** (green), **4** (blue), or **7** (yellow) and Glu85 resulted from the MD simulation. 185](#__RefHeading___Toc35529613)

[**Fig. S24** HBs lifetime for the **4** (black) or **7** (red) and Ser147 resulted from the MD simulation. 186](#__RefHeading___Toc35529614)

[**Fig. S25** HBs lifetime for the **5** and Tyr20 (black) or Glu17 (red) obtained from the MD simulation. 187](#__RefHeading___Toc35529615)

# **Table S1**: Configuration file used for docking procedure of azole **1**.

receptor = 2e9n_clear.pdbqt <-- file name (*.pdbqt) generated for prepared structure of protein (2e9n.pdb)

ligand = Tos_chlor_conf_9_opt.pdbqt <-- file name (*.pdbqt) generated for optimized structure of ligand **1**

out=dock_Tos_chlor_conf_9_opt_5.pdbqt <-- output file name (*.pdbqt) for generated docked poses of ligand **1**

log=log_Tos_chlor_conf_9_opt_5.txt <-- file name (*.txt) for resulted data related with docked poses of ligand **1**

exhaustiveness = 9

energy_range = 5

center_x =-1.0

center_y =10.0

center_z =-19.0

size_x = 30

size_y = 30

size_z = 30

Ligand‑amino acid electrostatic contacts (HBs under *d*≤2.2 Å) [Å] for first poses of azoles **1**‑**7** generated during docking procedure.

| HB | Hydrogen bond length calculated for docked azoles **1**‑**7** | | | | | | |
| --- | --- | --- | --- | --- | --- | --- | --- |
|  | **1** | **2** | **3** | **4** | **5** | **6** | **7** |
| N-H…O=C_Glu85_ | 2.082 | 2.107 | 2.267 | 2.031 | ☓ | ☓ | 2.060 |
| SO_2_…H-O_Ser147_ | 2.585 | 2.249 | 2.586 | 2.583 | ☓ | ☓ | 2.587 |
| SO_2_…H-N_Lys38_ | ☓ | ☓ | ☓ | ☓ | 2.692 | ☓ | ☓ |
| N2_indol_…O-H_Tyr20_ | ☓ | ☓ | ☓ | ☓ | 1.862 | ☓ | ☓ |
| N-H…O=C_Asn135_ | ☓ | ☓ | ☓ | ☓ | ☓ | 2.182 | ☓ |
| N2…H-O_Ser147_ | ☓ | ☓ | ☓ | ☓ | ☓ | 2.847 | ☓ |

# **Table S2**: Estimated binding affinity [kcal mol^-1^] for all poses for azoles **1**‑**7** generated during docking procedurę (results for the best poses are marked in bold).

| ***Pose*** | **Estimated binding affinity of the docked azoles** | | | | | | |
| --- | --- | --- | --- | --- | --- | --- | --- |
|  | **1** | **2** | **3** | **4** | **5** | **6** | **7** |
| ***1*** | **-9.8** | **-9.7** | **-9.8** | **-10.5** | **-9.7** | **-8.0** | **-10.6** |
| ***2*** | -8.8 | -9.1 | -9.4 | -10.1 | -9.5 | -7.8 | -9.4 |
| ***3*** | -8.8 | -9.0 | -9.2 | -9.9 | -9.1 | -7.8 | -9.4 |
| ***4*** | -8.7 | -9.0 | -8.8 | -9.8 | -8.8 | -7.7 | -9.3 |
| ***5*** | -8.6 | -9.0 | -8.7 | -9.7 | -8.7 | -7.3 | -9.3 |
| ***6*** | -8.6 | -8.8 | -8.7 | -9.7 | -8.7 | -7.2 | -9.2 |
| ***7*** | -8.5 | -8.7 | -8.6 | -9.6 | -8.7 | -7.1 | -8.9 |
| ***8*** | -8.5 | -8.6 | -8.6 | -9.5 | -8.6 | -7.1 | -8.9 |
| ***9*** | -8.4 | -8.3 | -8.6 | -9.4 | -8.6 | -7.0 | -8.8 |

# **Fig. S1** Electrostatic potential (ESP) map of docked azole **1** (1^st^ pose) calculated at the B3LYP/6-311++G(2d,3p)//B3LYP-631G(d,p) level of theory (gaseous phase); isovalue = 0.002 a.u.


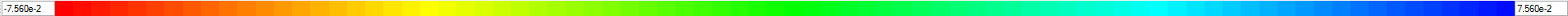


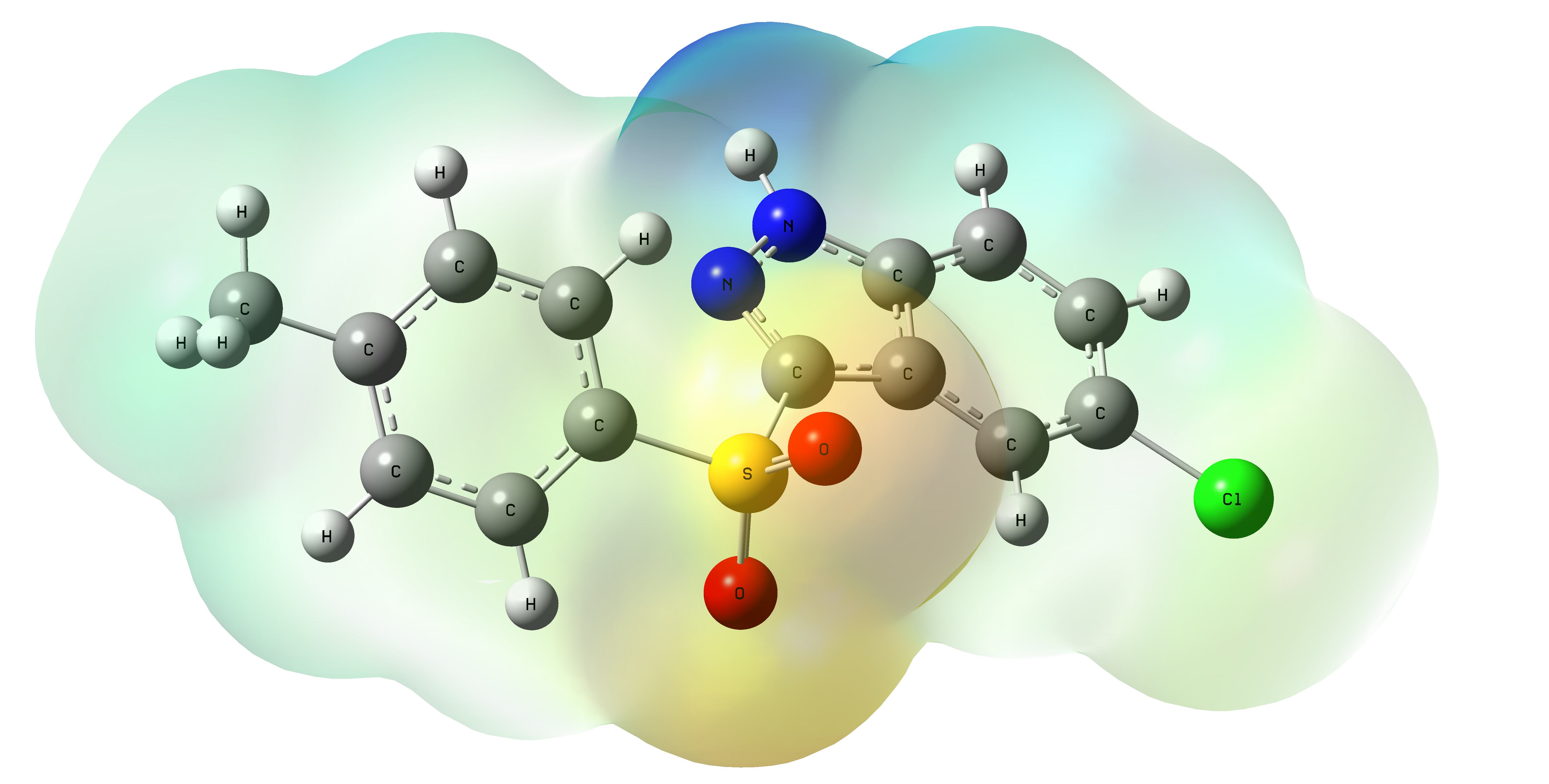


# **Fig. S2** Electrostatic potential (ESP) map of docked azole **2** (1^st^ pose) calculated at the B3LYP/6-311++G(2d,3p)//B3LYP-631G(d,p) level of theory (gaseous phase); isovalue = 0.002 a.u.


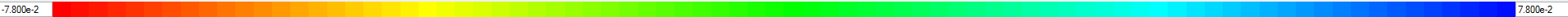


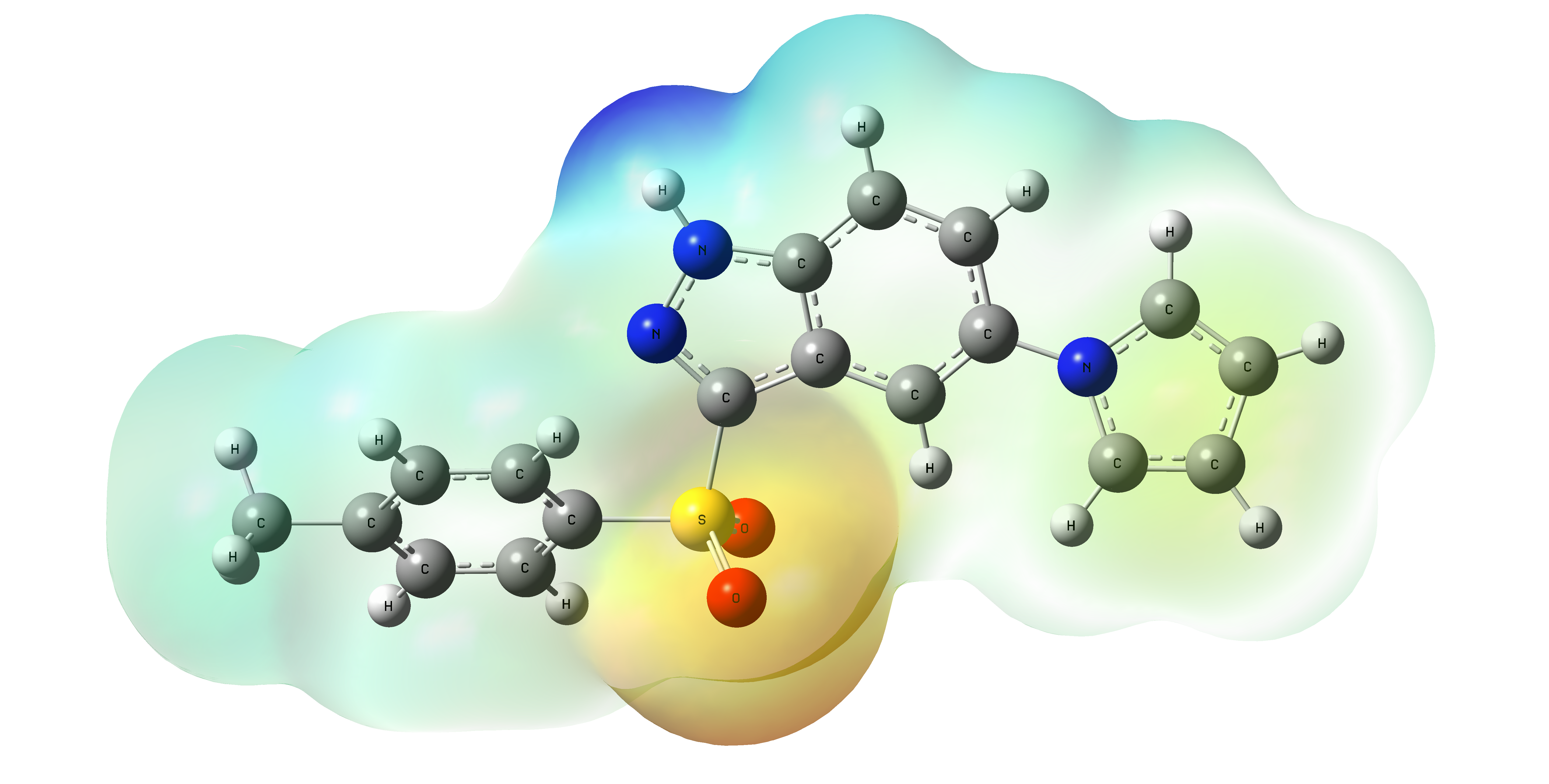


# **Fig. S3** Electrostatic potential (ESP) map of docked azole **3** (1^st^ pose) calculated at the B3LYP/6-311++G(2d,3p)//B3LYP-631G(d,p) level of theory (gaseous phase); isovalue = 0.002 a.u.


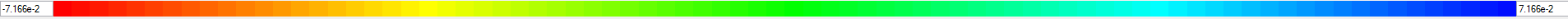


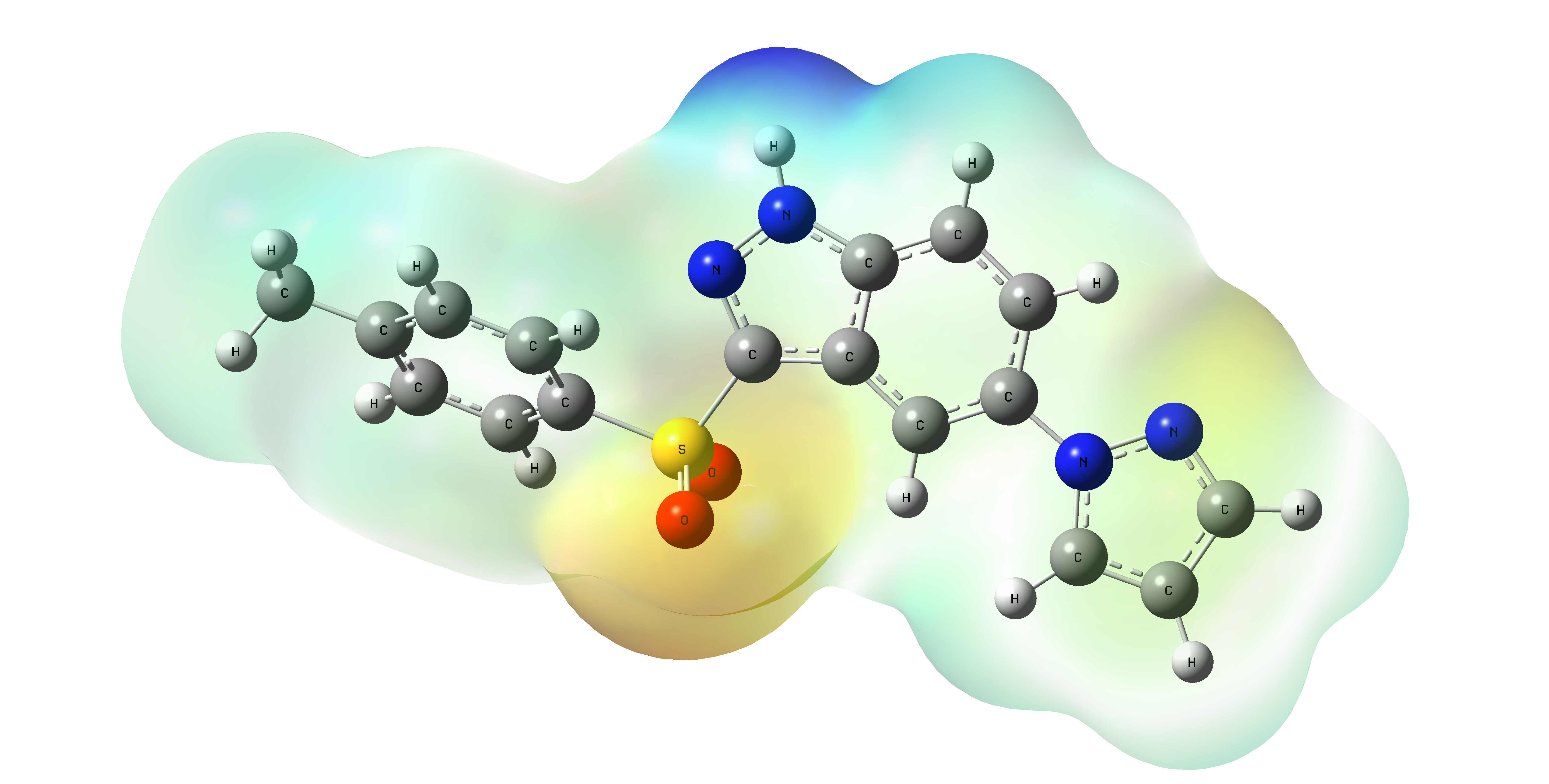


# **Fig. S4** Electrostatic potential (ESP) map of docked azole **4** (1^st^ pose) calculated at the B3LYP/6-311++G(2d,3p)//B3LYP-631G(d,p) level of theory (gaseous phase); isovalue = 0.002 a.u.


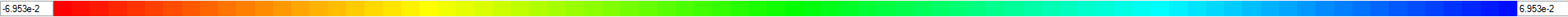


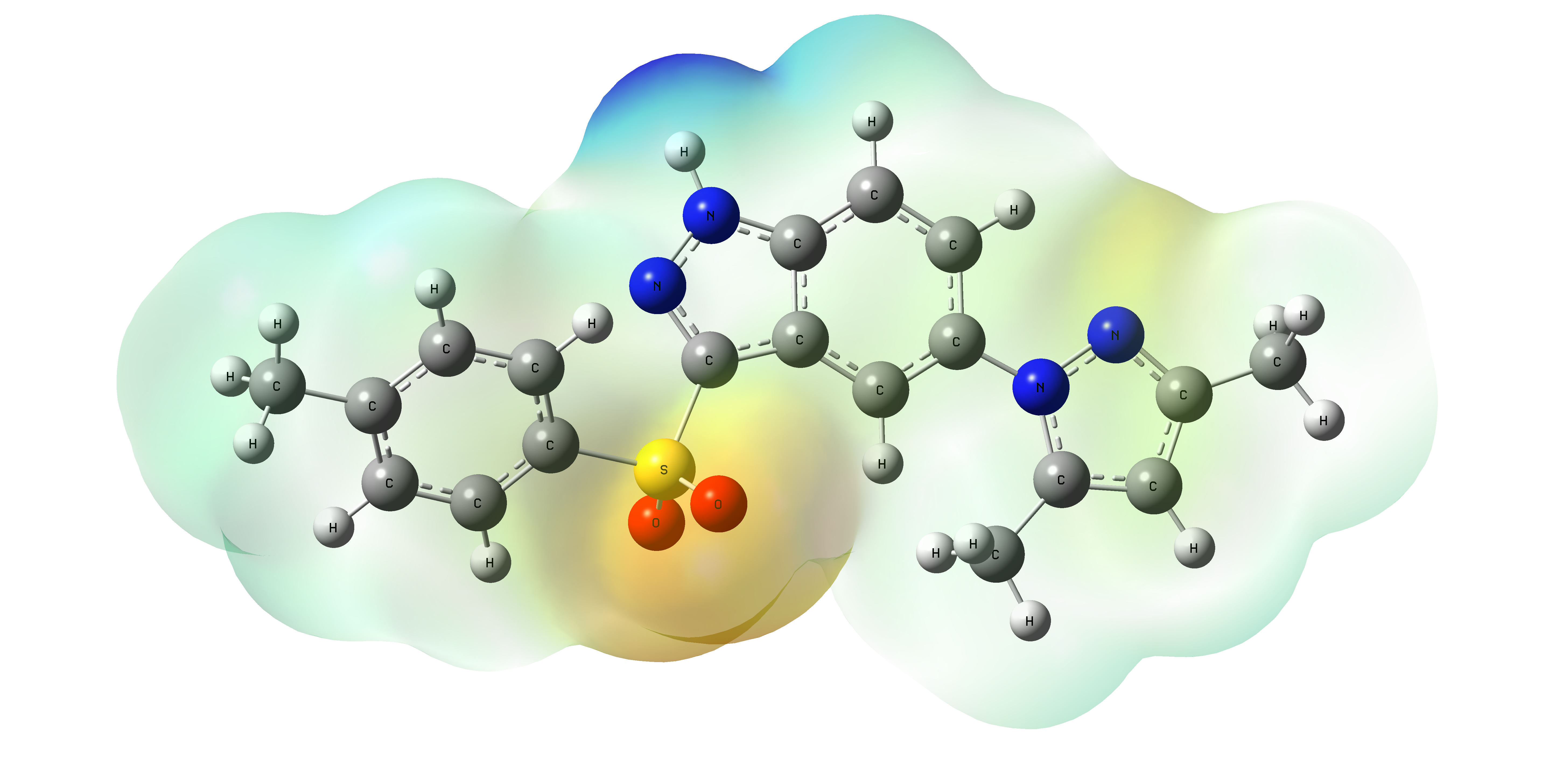


**Fig. S5** Electrostatic potential (ESP) map of docked azole **5** (1^st^ pose) calculated at the B3LYP/6-311++G(2d,3p)//B3LYP-631G(d,p) level of theory (gaseous phase); isovalue = 0.002 a.u.


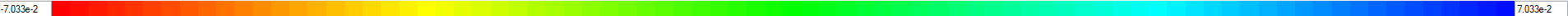


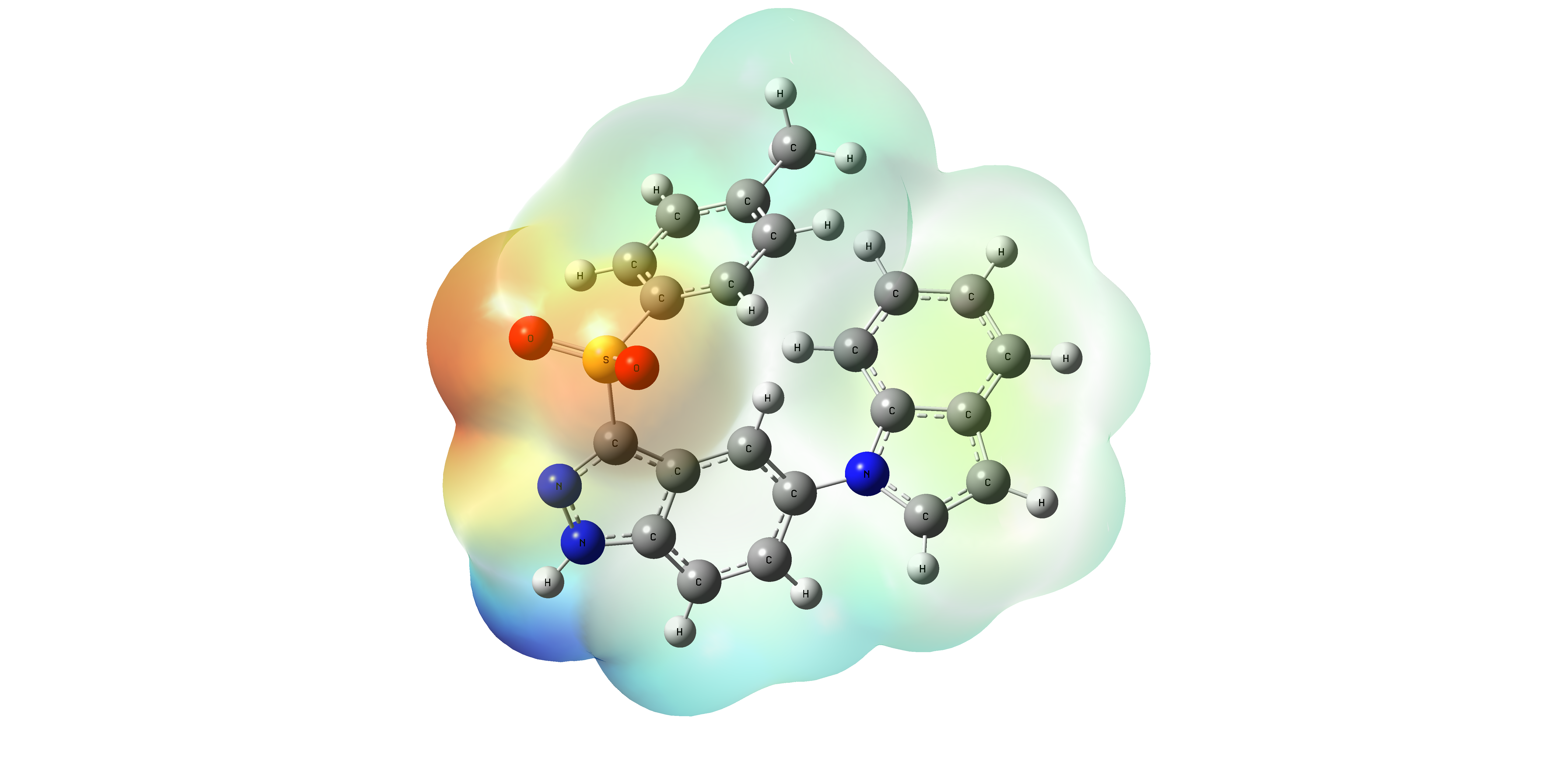


# **Fig. S6** Electrostatic potential (ESP) map of docked azole **6** (1^st^ pose) calculated at the B3LYP/6-311++G(2d,3p)//B3LYP-631G(d,p) level of theory (gaseous phase); isovalue = 0.002 a.u.


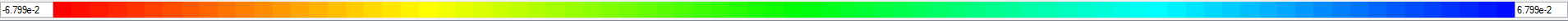


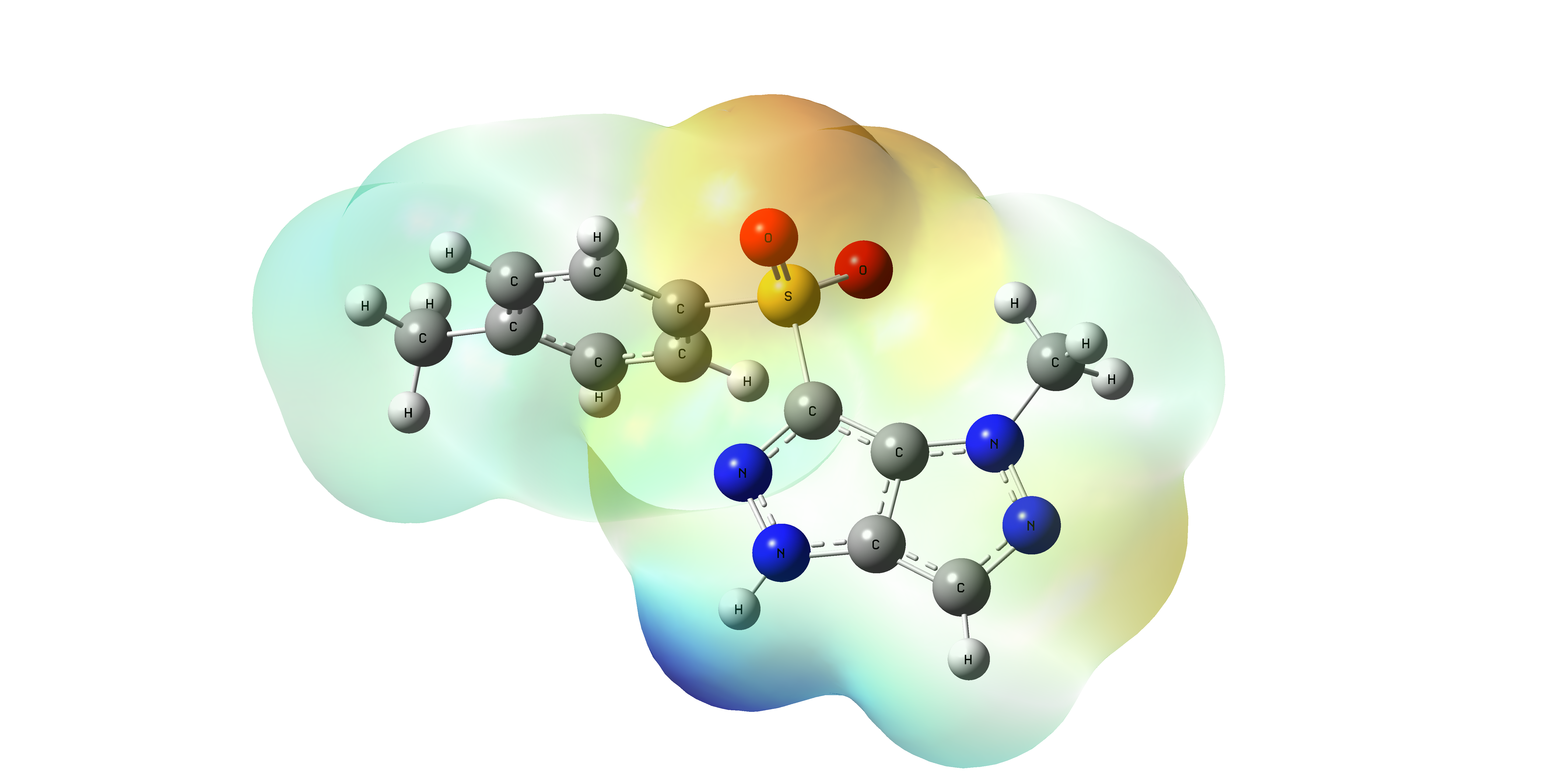


# **Fig. S7** Electrostatic potential (ESP) map of docked azole **7** (1^st^ pose) calculated at the B3LYP/6-311++G(2d,3p)//B3LYP-631G(d,p) level of theory (gaseous phase); isovalue = 0.002 a.u.


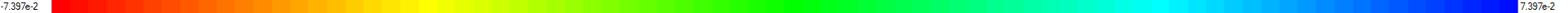


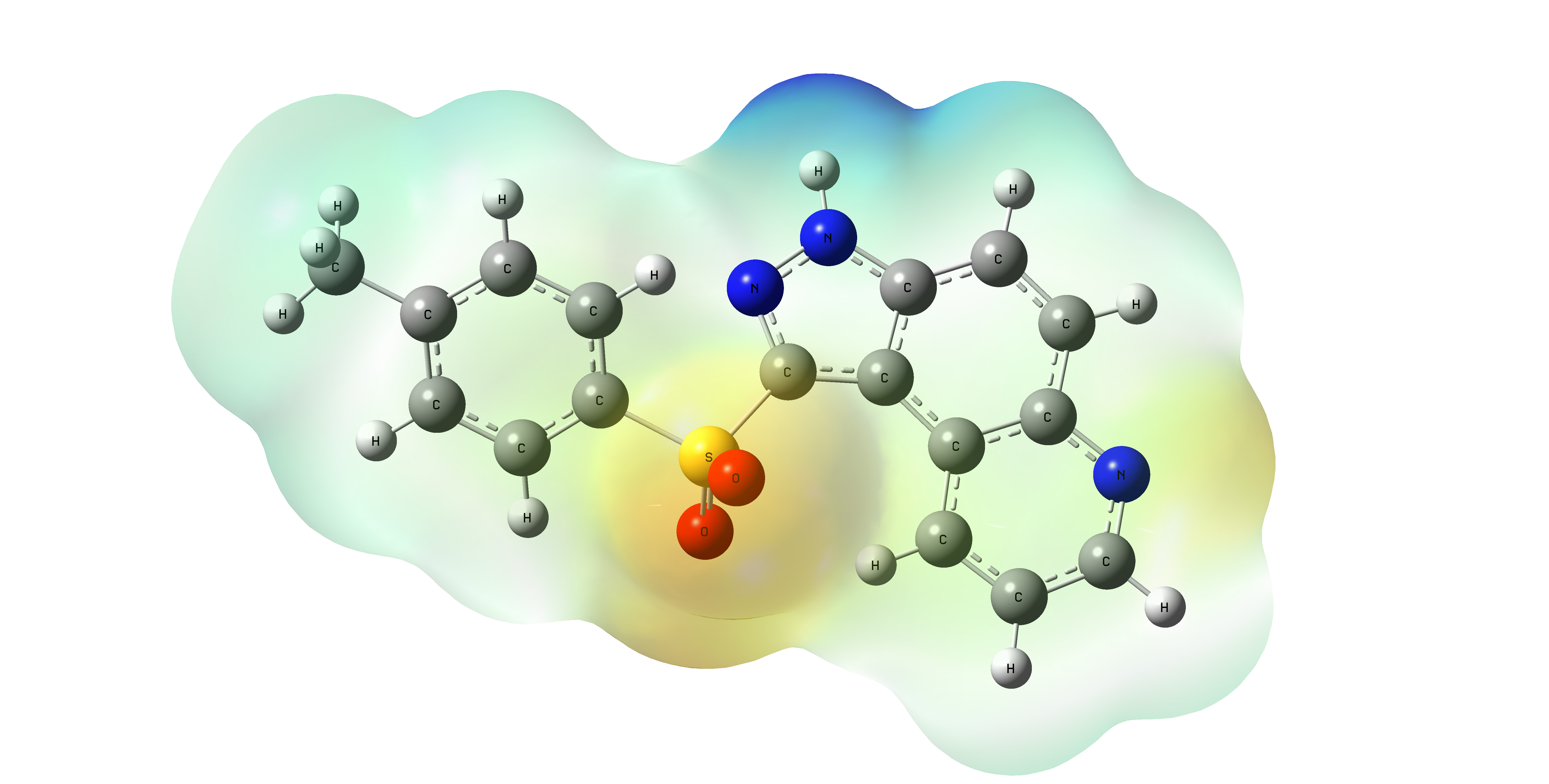


# **Fig. S8**: Geometry of the first poses of azoles **1**–**7** after docking procedure to 2e9n.pdb protein and their Cartesian coordinates (charge=0, multiplicity=1):

**Azole 1**


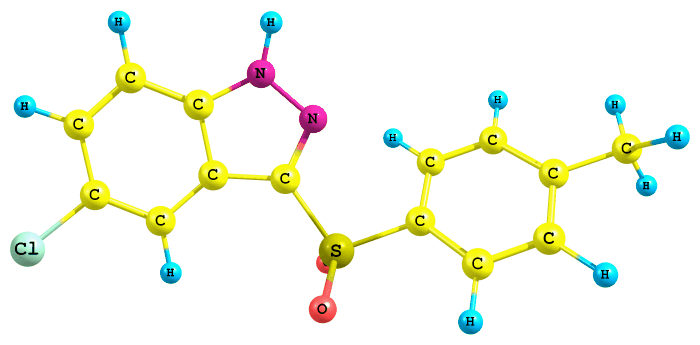


Cartesian coordinates:

C -6.323000000 9.395000000 -17.854000000

C -5.081000000 9.379000000 -18.568000000

C -5.195000000 10.480000000 -19.452000000

N -6.423000000 11.037000000 -19.203000000

N -7.108000000 10.383000000 -18.245000000

C -4.174000000 10.824000000 -20.351000000

C -3.036000000 10.037000000 -20.343000000

C -2.921000000 8.937000000 -19.460000000

C -3.920000000 8.584000000 -18.571000000

H -6.852000000 11.832000000 -19.649000000

Cl -1.441000000 7.988000000 -19.513000000

S -6.882000000 8.258000000 -16.588000000

O -6.023000000 8.532000000 -15.423000000

O -6.969000000 6.916000000 -17.176000000

C -8.545000000 8.801000000 -16.194000000

C -8.818000000 10.167000000 -16.123000000

C -10.111000000 10.581000000 -15.807000000

C -11.130000000 9.651000000 -15.561000000

C -12.519000000 10.108000000 -15.188000000

C -10.824000000 8.284000000 -15.640000000

C -9.538000000 7.850000000 -15.951000000

H -4.272000000 11.666000000 -21.020000000

H -2.225000000 10.265000000 -21.019000000

H -3.814000000 7.737000000 -17.909000000

H -8.040000000 10.892000000 -16.310000000

H -10.331000000 11.637000000 -15.751000000

H -11.600000000 7.555000000 -15.456000000

H -9.312000000 6.795000000 -16.003000000

H -12.554000000 11.197000000 -15.181000000

H -13.235000000 9.726000000 -15.916000000

H -12.772000000 9.730000000 -14.197000000

**Azole 2**


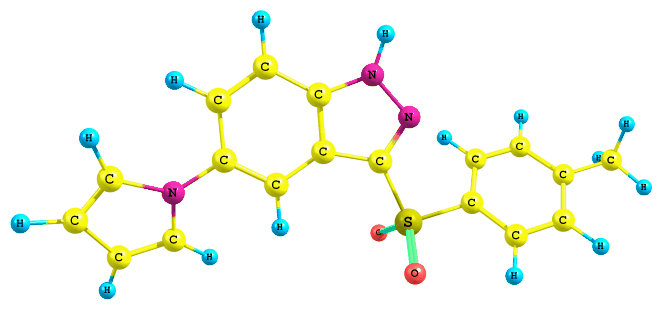


Cartesian coordinates:

C -3.737000000 8.703000000 -18.418000000

C -2.685000000 9.073000000 -19.219000000

C -2.829000000 10.120000000 -20.177000000

C -4.009000000 10.783000000 -20.346000000

C -5.084000000 10.404000000 -19.517000000

N -6.398000000 10.858000000 -19.428000000

H -6.845000000 11.615000000 -19.950000000

N -7.111000000 10.189000000 -18.440000000

C -6.255000000 9.318000000 -17.941000000

C -4.954000000 9.394000000 -18.568000000

N -1.406000000 8.410000000 -19.101000000

C -0.920000000 7.737000000 -17.976000000

C 0.316000000 7.276000000 -18.254000000

C 0.635000000 7.663000000 -19.597000000

C -0.416000000 8.349000000 -20.088000000

S -6.702000000 8.231000000 -16.585000000

O -6.872000000 6.549000000 -17.344000000

O -5.647000000 8.724000000 -15.131000000

C -8.374000000 8.800000000 -16.211000000

C -9.359000000 7.871000000 -15.912000000

C -10.635000000 8.312000000 -15.605000000

C -10.934000000 9.673000000 -15.588000000

C -9.926000000 10.589000000 -15.881000000

C -12.349000000 10.153000000 -15.270000000

C -8.645000000 10.159000000 -16.190000000

H -3.634000000 7.907000000 -17.696000000

H -1.980000000 10.395000000 -20.785000000

H -4.115000000 11.565000000 -21.083000000

H -1.446000000 7.608000000 -17.042000000

H 0.952000000 6.716000000 -17.585000000

H 1.553000000 7.444000000 -20.121000000

H -0.483000000 8.779000000 -21.076000000

H -9.133000000 6.815000000 -15.919000000

H -11.408000000 7.593000000 -15.376000000

H -10.145000000 11.646000000 -15.867000000

H -7.867000000 10.875000000 -16.412000000

H -12.988000000 9.293000000 -15.069000000

H -12.324000000 10.800000000 -14.393000000

H -12.744000000 10.709000000 -16.120000000

**Azole 3**


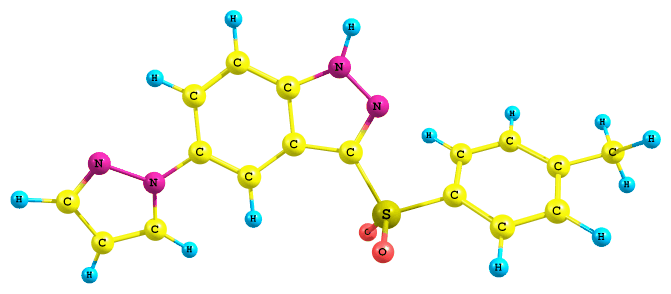


Cartesian coordinates:

C -6.306000000 9.259000000 -17.890000000

C -5.003000000 9.304000000 -18.482000000

C -5.137000000 10.283000000 -19.495000000

N -6.436000000 10.720000000 -19.427000000

N -7.146000000 10.101000000 -18.463000000

C -4.063000000 10.641000000 -20.325000000

C -2.862000000 9.989000000 -20.125000000

C -2.718000000 9.004000000 -19.111000000

C -3.770000000 8.661000000 -18.277000000

H -6.895000000 11.410000000 -20.001000000

S -6.870000000 8.200000000 -16.561000000

O -6.980000000 6.827000000 -17.069000000

O -5.997000000 8.523000000 -15.417000000

C -8.522000000 8.787000000 -16.181000000

C -8.765000000 10.159000000 -16.133000000

C -10.050000000 10.607000000 -15.830000000

C -11.090000000 9.704000000 -15.573000000

C -10.813000000 8.329000000 -15.628000000

C -12.471000000 10.196000000 -15.215000000

C -9.536000000 7.862000000 -15.927000000

N -1.457000000 8.368000000 -18.973000000

C -0.941000000 7.749000000 -17.868000000

C 0.310000000 7.288000000 -18.215000000

C 0.467000000 7.674000000 -19.566000000

N -0.597000000 8.323000000 -20.026000000

H -4.172000000 11.396000000 -21.089000000

H -2.016000000 10.232000000 -20.751000000

H -3.647000000 7.925000000 -17.496000000

H -7.970000000 10.864000000 -16.328000000

H -10.247000000 11.668000000 -15.793000000

H -11.605000000 7.620000000 -15.435000000

H -9.333000000 6.802000000 -15.962000000

H -1.422000000 7.642000000 -16.907000000

H 1.013000000 6.752000000 -17.594000000

H 1.348000000 7.466000000 -20.155000000

H -12.483000000 11.286000000 -15.226000000

H -13.191000000 9.817000000 -15.941000000

H -12.737000000 9.840000000 -14.220000000

**Azole 4**


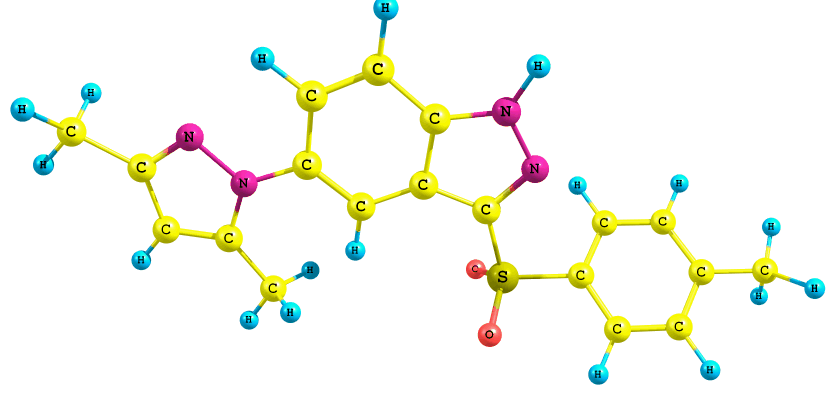


Cartesian coordinates:

C -4.008000000 8.849000000 -18.534000000

C -5.234000000 9.537000000 -18.589000000

C -2.989000000 9.277000000 -19.372000000

C -5.414000000 10.605000000 -19.498000000

C -3.184000000 10.351000000 -20.284000000

C -4.389000000 11.019000000 -20.366000000

C -6.498000000 9.449000000 -17.924000000

N -7.358000000 10.352000000 -18.363000000

N -6.700000000 11.043000000 -19.316000000

H -7.178000000 11.796000000 -19.785000000

S -6.975000000 8.330000000 -16.611000000

O -7.052000000 7.002000000 -17.247000000

O -6.107000000 8.569000000 -15.453000000

C -8.639000000 8.832000000 -16.170000000

C -8.944000000 10.190000000 -16.080000000

C -10.243000000 10.571000000 -15.751000000

C -11.238000000 9.615000000 -15.506000000

C -12.636000000 10.035000000 -15.120000000

C -10.903000000 8.256000000 -15.612000000

C -9.612000000 7.856000000 -15.943000000

N -1.717000000 8.648000000 -19.361000000

N -0.721000000 9.186000000 -20.129000000

C 0.347000000 8.429000000 -19.899000000

C 1.643000000 8.718000000 -20.592000000

C 0.051000000 7.401000000 -18.970000000

C -1.278000000 7.563000000 -18.633000000

C -2.105000000 6.794000000 -17.650000000

H -3.865000000 8.016000000 -17.862000000

H -2.370000000 10.651000000 -20.927000000

H -4.538000000 11.826000000 -21.068000000

H -8.184000000 10.935000000 -16.263000000

H -10.487000000 11.621000000 -15.684000000

H -11.661000000 7.507000000 -15.433000000

H -9.366000000 6.807000000 -16.023000000

H 0.727000000 6.644000000 -18.601000000

H -12.694000000 11.123000000 -15.092000000

H -13.346000000 9.652000000 -15.853000000

H -12.877000000 9.633000000 -14.136000000

H -1.517000000 5.971000000 -17.245000000

H -2.412000000 7.454000000 -16.839000000

H -2.989000000 6.397000000 -18.150000000

H 2.394000000 7.994000000 -20.276000000

H 1.976000000 9.723000000 -20.334000000

H 1.502000000 8.648000000 -21.671000000

**Azole 5**


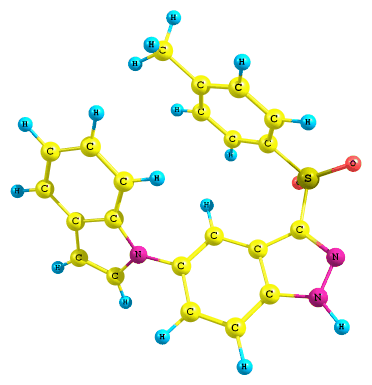


Cartesian coordinates:

C -5.001000000 5.256000000 -14.776000000

C -3.951000000 5.741000000 -15.621000000

C -3.080000000 4.629000000 -15.729000000

N -3.645000000 3.631000000 -14.977000000

N -4.807000000 4.003000000 -14.403000000

C -1.902000000 4.681000000 -16.489000000

C -1.617000000 5.875000000 -17.125000000

C -2.489000000 6.996000000 -17.041000000

C -3.662000000 6.937000000 -16.299000000

H -3.305000000 2.696000000 -14.821000000

S -6.479000000 6.122000000 -14.250000000

O -7.502000000 5.073000000 -14.095000000

O -6.126000000 7.014000000 -13.140000000

C -6.935000000 7.139000000 -15.656000000

C -7.681000000 6.576000000 -16.691000000

C -8.042000000 7.375000000 -17.774000000

C -7.667000000 8.724000000 -17.837000000

C -8.087000000 9.591000000 -18.999000000

C -6.917000000 9.261000000 -16.779000000

C -6.550000000 8.481000000 -15.687000000

N -2.135000000 8.176000000 -17.743000000

C -0.928000000 8.855000000 -17.592000000

C -0.902000000 9.958000000 -18.394000000

C -2.155000000 10.003000000 -19.094000000

C -2.717000000 10.892000000 -20.026000000

C -2.910000000 8.875000000 -18.671000000

C -4.000000000 10.650000000 -20.500000000

C -4.738000000 9.536000000 -20.054000000

C -4.209000000 8.637000000 -19.135000000

H -1.247000000 3.826000000 -16.573000000

H -0.708000000 5.959000000 -17.701000000

H -4.330000000 7.784000000 -16.245000000

H -0.127000000 8.551000000 -16.934000000

H -0.091000000 10.665000000 -18.485000000

H -2.159000000 11.751000000 -20.369000000

H -4.438000000 11.325000000 -21.220000000

H -5.736000000 9.377000000 -20.434000000

H -4.779000000 7.786000000 -18.792000000

H -7.974000000 5.537000000 -16.654000000

H -8.621000000 6.947000000 -18.579000000

H -6.619000000 10.299000000 -16.813000000

H -5.977000000 8.906000000 -14.877000000

H -9.103000000 9.951000000 -18.836000000

H -7.410000000 10.441000000 -19.081000000

H -8.051000000 9.008000000 -19.919000000

**Azole 6**


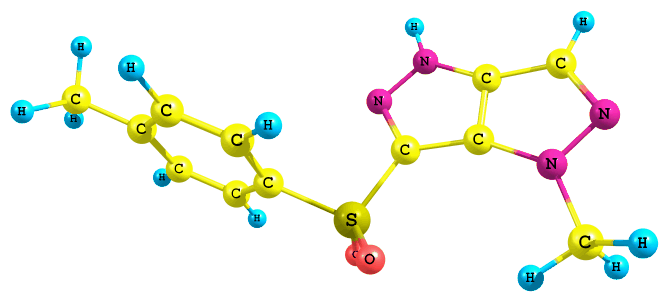


Cartesian coordinates:

C -6.213000000 7.717000000 -15.414000000

N -6.616000000 8.152000000 -14.221000000

C -4.977000000 6.646000000 -13.888000000

N -5.867000000 7.508000000 -13.305000000

C -3.956000000 5.686000000 -13.711000000

H -6.028000000 7.699000000 -12.330000000

N -4.296000000 5.886000000 -15.852000000

C -5.171000000 6.758000000 -15.269000000

N -3.560000000 5.248000000 -14.906000000

C -4.021000000 5.633000000 -17.259000000

S -7.021000000 8.331000000 -16.884000000

O -7.203000000 7.216000000 -17.821000000

O -6.257000000 9.532000000 -17.271000000

C -8.642000000 8.864000000 -16.330000000

C -9.601000000 7.904000000 -15.998000000

C -10.860000000 8.328000000 -15.583000000

C -11.176000000 9.693000000 -15.497000000

C -12.538000000 10.138000000 -15.022000000

C -10.196000000 10.630000000 -15.849000000

C -8.927000000 10.227000000 -16.264000000

H -3.560000000 5.360000000 -12.761000000

H -9.369000000 6.851000000 -16.062000000

H -11.608000000 7.593000000 -15.322000000

H -10.427000000 11.684000000 -15.798000000

H -8.178000000 10.958000000 -16.530000000

H -12.587000000 11.227000000 -15.026000000

H -13.303000000 9.738000000 -15.687000000

H -12.708000000 9.770000000 -14.010000000

H -4.700000000 6.224000000 -17.874000000

H -4.166000000 4.574000000 -17.473000000

H -2.991000000 5.912000000 -17.484000000

**Azole 7**


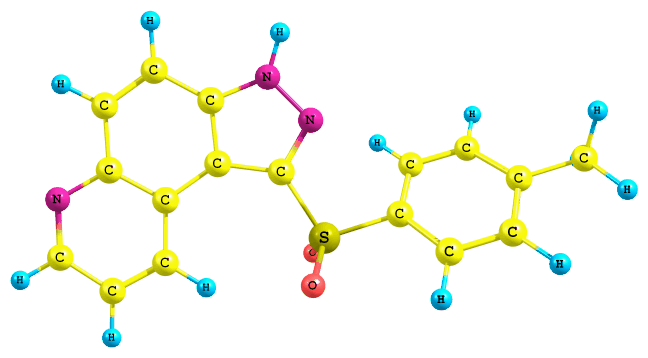


Cartesian coordinates:

C -3.983000000 8.723000000 -18.667000000

C -2.970000000 9.211000000 -19.555000000

C -3.687000000 7.563000000 -17.918000000

N -1.756000000 8.614000000 -19.682000000

C -2.448000000 6.973000000 -18.061000000

C -1.514000000 7.539000000 -18.953000000

C -5.219000000 9.457000000 -18.619000000

C -5.378000000 10.588000000 -19.444000000

C -3.200000000 10.372000000 -20.369000000

C -4.378000000 11.062000000 -20.332000000

C -6.466000000 9.393000000 -17.917000000

N -7.292000000 10.370000000 -18.264000000

N -6.625000000 11.082000000 -19.191000000

H -7.077000000 11.880000000 -19.611000000

S -6.990000000 8.296000000 -16.593000000

O -7.064000000 6.937000000 -17.162000000

O -6.134000000 8.556000000 -15.426000000

C -8.657000000 8.823000000 -16.205000000

C -9.638000000 7.858000000 -15.973000000

C -10.921000000 8.273000000 -15.629000000

C -11.237000000 9.635000000 -15.514000000

C -12.639000000 10.075000000 -15.169000000

C -10.225000000 10.580000000 -15.741000000

C -8.935000000 10.184000000 -16.087000000

H -4.419000000 7.144000000 -17.244000000

H -2.196000000 6.088000000 -17.496000000

H -0.547000000 7.067000000 -19.045000000

H -2.417000000 10.710000000 -21.031000000

H -4.541000000 11.931000000 -20.952000000

H -9.405000000 6.807000000 -16.059000000

H -11.687000000 7.534000000 -15.447000000

H -10.450000000 11.632000000 -15.646000000

H -8.163000000 10.919000000 -16.261000000

H -13.272000000 9.198000000 -15.032000000

H -12.620000000 10.657000000 -14.248000000

H -13.037000000 10.688000000 -15.978000000

# **Cartesian coordinates of azoles 1‑7 with corresponding residues around 4 Å after optimization with DFT formalism** (ligands optimization, B3LYP/6-31G(d,p) level of theory, results given in the Table 3, ***Gaussian G16.A01* software**)

Azole **1** with surrounded residues

N -2.564000000 3.856000000 -23.064000000

C -2.089000000 4.847000000 -22.104000000

C -1.721000000 4.207000000 -20.769000000

O -0.655000000 4.475000000 -20.213000000

C -3.161000000 5.910000000 -21.873000000

C -3.399000000 6.915000000 -22.996000000

C -4.670000000 7.698000000 -22.725000000

C -2.199000000 7.842000000 -23.096000000

H -2.738000000 2.956000000 -22.558000000

H -1.922000000 3.695000000 -23.837000000

H -1.157000000 5.341000000 -22.504000000

H -2.906000000 6.459000000 -20.934000000

H -4.119000000 5.407000000 -21.589000000

H -3.523000000 6.368000000 -23.964000000

H -4.799000000 8.506000000 -23.450000000

H -4.657000000 8.145000000 -21.720000000

H -5.548000000 7.038000000 -22.789000000

H -2.274000000 8.508000000 -23.963000000

H -1.256000000 7.293000000 -23.201000000

H -2.103000000 8.475000000 -22.203000000

H -3.546000000 4.121000000 -23.427000000

O -2.504000000 3.408000000 -20.237000000

N -6.914000000 2.232000000 -21.285000000

C -7.141000000 3.614000000 -21.675000000

C -6.212000000 3.896000000 -22.850000000

O -4.998000000 3.971000000 -22.680000000

C -6.812000000 4.580000000 -20.522000000

C -7.120000000 6.006000000 -20.938000000

C -7.614000000 4.206000000 -19.290000000

H -8.212000000 3.774000000 -22.024000000

H -5.709000000 4.511000000 -20.290000000

H -7.286000000 3.282000000 -18.809000000

H -8.692000000 4.161000000 -19.487000000

H -7.479000000 4.991000000 -18.510000000

H -8.192000000 6.155000000 -21.121000000

H -6.813000000 6.715000000 -20.156000000

H -6.592000000 6.272000000 -21.864000000

H -7.251000000 2.042000000 -20.320000000

H -7.398000000 1.581000000 -21.930000000

O -6.685000000 4.051000000 -23.984000000

N -7.810000000 11.109000000 -24.650000000

C -7.667000000 9.874000000 -23.900000000

C -9.024000000 9.198000000 -23.777000000

O -9.997000000 9.801000000 -23.325000000

C -7.092000000 10.166000000 -22.525000000

H -6.933000000 9.191000000 -24.444000000

H -7.640000000 10.962000000 -21.984000000

H -6.031000000 10.462000000 -22.547000000

H -7.142000000 9.286000000 -21.857000000

H -8.530000000 11.726000000 -24.161000000

H -8.125000000 10.968000000 -25.620000000

N -9.086000000 7.939000000 -24.187000000

H -8.264000000 7.347000000 -24.381000000

C -10.334000000 7.200000000 -24.132000000

C -10.241000000 5.992000000 -23.215000000

O -9.362000000 5.136000000 -23.368000000

C -10.757000000 6.739000000 -25.535000000

C -12.086000000 6.006000000 -25.467000000

C -10.854000000 7.941000000 -26.455000000

H -11.146000000 7.897000000 -23.752000000

H -9.979000000 6.025000000 -25.925000000

H -11.260000000 7.649000000 -27.435000000

H -11.525000000 8.711000000 -26.059000000

H -9.881000000 8.401000000 -26.646000000

H -12.385000000 5.622000000 -26.450000000

H -12.050000000 5.120000000 -24.800000000

H -12.904000000 6.634000000 -25.100000000

N -11.160000000 5.939000000 -22.257000000

C -11.208000000 4.850000000 -21.299000000

C -12.247000000 3.846000000 -21.763000000

O -13.295000000 4.220000000 -22.292000000

C -11.569000000 5.384000000 -19.908000000

C -11.561000000 4.333000000 -18.800000000

C -11.519000000 4.986000000 -17.417000000

C -11.276000000 3.955000000 -16.326000000

N -10.825000000 4.567000000 -15.042000000

H -10.594000000 3.862000000 -14.318000000

H -9.999000000 5.174000000 -15.162000000

H -10.209000000 4.335000000 -21.263000000

H -12.583000000 5.844000000 -19.953000000

H -10.870000000 6.206000000 -19.653000000

H -12.459000000 3.682000000 -18.912000000

H -10.706000000 3.645000000 -18.943000000

H -10.737000000 5.772000000 -17.408000000

H -12.483000000 5.526000000 -17.232000000

H -12.228000000 3.386000000 -16.135000000

H -10.521000000 3.204000000 -16.656000000

H -12.027000000 6.473000000 -22.315000000

O -12.039000000 2.635000000 -21.608000000

N -19.659000000 7.628000000 -11.972000000

C -18.418000000 8.207000000 -12.466000000

C -18.674000000 9.165000000 -13.615000000

O -17.964000000 10.150000000 -13.771000000

C -17.459000000 7.107000000 -12.934000000

C -16.274000000 7.626000000 -13.753000000

C -15.176000000 6.582000000 -13.982000000

O -14.245000000 6.852000000 -14.766000000

O -15.229000000 5.495000000 -13.377000000

H -17.874000000 8.770000000 -11.635000000

H -17.006000000 6.573000000 -12.055000000

H -17.984000000 6.342000000 -13.536000000

H -15.793000000 8.469000000 -13.213000000

H -16.603000000 8.014000000 -14.731000000

H -20.138000000 8.254000000 -11.314000000

H -19.485000000 6.722000000 -11.500000000

O -19.607000000 8.949000000 -14.400000000

N -18.408000000 13.436000000 -13.639000000

C -17.583000000 14.107000000 -14.633000000

C -18.350000000 15.230000000 -15.328000000

O -17.787000000 16.280000000 -15.632000000

C -17.068000000 13.094000000 -15.661000000

C -15.763000000 12.428000000 -15.228000000

O -14.713000000 13.071000000 -15.171000000

N -15.826000000 11.140000000 -14.919000000

H -15.033000000 10.642000000 -14.550000000

H -16.709000000 10.611000000 -14.872000000

H -16.681000000 14.591000000 -14.140000000

H -17.851000000 12.359000000 -15.939000000

H -16.858000000 13.643000000 -16.614000000

H -19.417000000 13.569000000 -13.906000000

H -18.271000000 13.809000000 -12.695000000

O -19.552000000 15.081000000 -15.588000000

N -8.842000000 16.763000000 -14.873000000

C -8.904000000 16.251000000 -16.233000000

C -10.236000000 16.661000000 -16.839000000

O -11.274000000 16.583000000 -16.184000000

C -8.757000000 14.730000000 -16.248000000

C -9.094000000 14.181000000 -17.629000000

C -7.333000000 14.369000000 -15.868000000

H -8.096000000 16.723000000 -16.883000000

H -9.484000000 14.281000000 -15.522000000

H -9.013000000 13.089000000 -17.656000000

H -8.410000000 14.582000000 -18.397000000

H -10.118000000 14.461000000 -17.922000000

H -6.602000000 14.949000000 -16.472000000

H -7.095000000 13.329000000 -16.133000000

H -7.098000000 14.538000000 -14.821000000

H -8.216000000 16.234000000 -14.264000000

H -8.540000000 17.759000000 -14.877000000

O -10.277000000 17.078000000 -18.005000000

N -11.852000000 11.728000000 -22.235000000

C -11.252000000 12.660000000 -21.292000000

C -10.018000000 13.331000000 -21.868000000

O -9.420000000 12.837000000 -22.819000000

C -10.880000000 11.902000000 -20.021000000

C -12.053000000 11.078000000 -19.490000000

C -11.581000000 9.694000000 -19.097000000

C -12.706000000 11.813000000 -18.329000000

H -12.024000000 13.456000000 -21.021000000

H -10.515000000 12.607000000 -19.236000000

H -9.991000000 11.254000000 -20.199000000

H -12.860000000 10.938000000 -20.265000000

H -13.604000000 11.290000000 -17.962000000

H -12.039000000 11.897000000 -17.458000000

H -13.021000000 12.833000000 -18.583000000

H -12.402000000 9.086000000 -18.685000000

H -11.148000000 9.133000000 -19.931000000

H -10.814000000 9.731000000 -18.305000000

H -12.616000000 11.163000000 -21.780000000

H -12.232000000 12.205000000 -23.067000000

N -9.636000000 14.455000000 -21.276000000

H -10.056000000 14.810000000 -20.402000000

C -8.474000000 15.205000000 -21.731000000

C -7.220000000 14.350000000 -21.754000000

O -7.030000000 13.486000000 -20.901000000

C -8.221000000 16.412000000 -20.820000000

C -7.118000000 17.331000000 -21.327000000

C -6.586000000 18.273000000 -20.262000000

O -7.384000000 19.025000000 -19.657000000

O -5.359000000 18.261000000 -20.035000000

H -8.702000000 15.579000000 -22.774000000

H -9.144000000 17.022000000 -20.668000000

H -7.955000000 16.076000000 -19.791000000

H -7.478000000 17.957000000 -22.162000000

H -6.227000000 16.779000000 -21.689000000

N -6.362000000 14.589000000 -22.735000000

H -6.339000000 15.508000000 -23.208000000

C -5.117000000 13.848000000 -22.810000000

C -4.047000000 14.649000000 -22.083000000

O -3.991000000 15.874000000 -22.210000000

C -4.678000000 13.637000000 -24.256000000

C -3.285000000 13.061000000 -24.334000000

C -2.980000000 11.863000000 -23.704000000

C -2.267000000 13.732000000 -24.994000000

C -1.696000000 11.347000000 -23.725000000

C -0.975000000 13.224000000 -25.022000000

C -0.697000000 12.030000000 -24.382000000

O 0.585000000 11.521000000 -24.383000000

H 1.062000000 11.931000000 -23.604000000

H -5.266000000 12.846000000 -22.315000000

H -5.388000000 12.988000000 -24.795000000

H -4.702000000 14.615000000 -24.794000000

H -3.755000000 11.310000000 -23.177000000

H -2.462000000 14.700000000 -25.461000000

H -1.452000000 10.428000000 -23.190000000

H -0.170000000 13.787000000 -25.497000000

N -3.202000000 13.962000000 -21.321000000

C -2.138000000 14.627000000 -20.578000000

C -0.785000000 14.060000000 -21.008000000

O -0.324000000 13.047000000 -20.490000000

C -2.346000000 14.435000000 -19.063000000

S -3.922000000 15.086000000 -18.375000000

H -2.131000000 15.732000000 -20.832000000

H -1.497000000 14.926000000 -18.533000000

H -2.289000000 13.362000000 -18.804000000

H -4.089000000 16.235000000 -19.092000000

H -3.193000000 12.954000000 -21.264000000

O -0.141000000 14.634000000 -21.897000000

N -2.154000000 13.882000000 -12.921000000

C -1.496000000 13.980000000 -14.208000000

C -0.940000000 15.358000000 -14.520000000

O -1.238000000 16.336000000 -13.849000000

C -2.456000000 13.539000000 -15.313000000

C -3.008000000 12.130000000 -15.078000000

C -3.818000000 11.709000000 -16.275000000

C -1.871000000 11.156000000 -14.838000000

H -0.592000000 13.295000000 -14.231000000

H -3.266000000 14.278000000 -15.459000000

H -1.898000000 13.586000000 -16.289000000

H -3.689000000 12.120000000 -14.188000000

H -4.282000000 10.722000000 -16.127000000

H -4.621000000 12.416000000 -16.512000000

H -3.199000000 11.627000000 -17.184000000

H -2.232000000 10.115000000 -14.863000000

H -1.107000000 11.228000000 -15.631000000

H -1.376000000 11.287000000 -13.876000000

H -1.580000000 13.379000000 -12.229000000

H -3.070000000 13.416000000 -12.971000000

O -0.166000000 15.495000000 -15.477000000

N -7.232000000 13.290000000 -11.662000000

C -7.919000000 12.392000000 -12.581000000

C -8.383000000 11.096000000 -11.941000000

O -7.817000000 10.651000000 -10.947000000

C -7.017000000 12.057000000 -13.774000000

O -6.332000000 10.827000000 -13.589000000

H -6.581000000 10.113000000 -14.236000000

H -8.838000000 12.935000000 -12.973000000

H -7.620000000 12.015000000 -14.706000000

H -6.199000000 12.792000000 -13.914000000

H -6.291000000 13.552000000 -11.993000000

H -7.774000000 14.143000000 -11.468000000

N -9.409000000 10.496000000 -12.542000000

H -9.888000000 10.926000000 -13.329000000

C -10.005000000 9.238000000 -12.095000000

C -11.043000000 9.433000000 -10.996000000

O -10.729000000 9.410000000 -9.804000000

C -8.935000000 8.242000000 -11.633000000

C -9.461000000 6.806000000 -11.586000000

O -8.656000000 5.868000000 -11.368000000

O -10.685000000 6.617000000 -11.771000000

H -10.506000000 8.781000000 -13.006000000

H -8.035000000 8.279000000 -12.262000000

H -8.595000000 8.480000000 -10.599000000

N -12.293000000 9.620000000 -11.407000000

C -13.367000000 9.829000000 -10.459000000

C -14.265000000 8.614000000 -10.320000000

O -15.444000000 8.727000000 -9.974000000

C -14.166000000 11.065000000 -10.864000000

C -13.355000000 12.320000000 -10.833000000

C -12.522000000 12.648000000 -11.889000000

C -13.352000000 13.128000000 -9.704000000

C -11.703000000 13.748000000 -11.816000000

C -12.526000000 14.234000000 -9.632000000

C -11.704000000 14.540000000 -10.684000000

H -12.932000000 9.996000000 -9.426000000

H -14.613000000 10.907000000 -11.869000000

H -15.051000000 11.124000000 -10.187000000

H -12.531000000 12.025000000 -12.788000000

H -13.993000000 12.872000000 -8.858000000

H -11.082000000 14.018000000 -12.666000000

H -12.532000000 14.857000000 -8.738000000

H -11.067000000 15.419000000 -10.636000000

H -12.610000000 9.110000000 -12.245000000

O -13.806000000 7.488000000 -10.558000000

C -5.462000000 8.980000000 -18.076000000

C -4.080000000 8.901000000 -18.438000000

C -3.946000000 9.879000000 -19.452000000

N -5.195000000 10.427000000 -19.615000000

N -6.109000000 9.891000000 -18.773000000

C -2.705000000 10.127000000 -20.058000000

C -1.619000000 9.387000000 -19.622000000

C -1.761000000 8.411000000 -18.611000000

C -2.975000000 8.150000000 -18.005000000

H -5.488000000 11.212000000 -20.178000000

Cl -0.335000000 7.509000000 -18.124000000

S -6.181000000 8.040000000 -16.737000000

O -5.354000000 8.359000000 -15.561000000

O -6.267000000 6.634000000 -17.154000000

C -7.855000000 8.658000000 -16.496000000

C -8.174000000 10.016000000 -16.479000000

C -9.458000000 10.404000000 -16.099000000

C -10.428000000 9.460000000 -15.736000000

C -11.835000000 9.876000000 -15.372000000

C -10.082000000 8.104000000 -15.773000000

C -8.808000000 7.696000000 -16.159000000

H -2.577000000 10.849000000 -20.853000000

H -0.642000000 9.545000000 -20.066000000

H -3.069000000 7.408000000 -17.222000000

H -7.439000000 10.759000000 -16.764000000

H -9.714000000 11.459000000 -16.086000000

H -10.828000000 7.367000000 -15.494000000

H -8.533000000 6.649000000 -16.206000000

H -11.887000000 10.923000000 -15.060000000

H -12.252000000 9.251000000 -14.578000000

H -12.510000000 9.758000000 -16.226000000

H -20.483000000 14.966000000 -15.789000000

H -13.450000000 6.616000000 -10.742000000

H 0.434000000 15.601000000 -16.219000000

H -3.111000000 2.789000000 -19.825000000

H -8.040000000 5.151000000 -11.201000000

H -11.878000000 1.696000000 -21.488000000

H 0.358000000 15.079000000 -22.586000000

H -4.415000000 18.252000000 -19.860000000

H -13.528000000 7.060000000 -15.370000000

H -10.309000000 17.401000000 -18.908000000

H -20.330000000 8.782000000 -15.009000000

Azole **2** with surrounded residues

N 0.594079000 8.830274000 -5.979801000

C 0.711027000 8.452502000 -4.580814000

C 1.948980000 9.157249000 -4.038602000

O 3.070904000 8.808903000 -4.396740000

C 0.882801000 6.930142000 -4.426799000

C 0.936908000 6.561805000 -2.956154000

C -0.264715000 6.209268000 -5.108307000

H -0.171963000 8.781858000 -4.033540000

H 1.818214000 6.631329000 -4.900012000

H -0.291489000 6.483285000 -6.163107000

H -0.122484000 5.132549000 -5.015855000

H -1.204267000 6.493769000 -4.634997000

H -0.054874000 6.259972000 -2.620200000

H 1.269726000 7.424263000 -2.377997000

H 1.635223000 5.737722000 -2.813641000

H 1.273541000 9.452124000 -6.393728000

H 0.585948000 7.980396000 -6.526012000

O 1.822350000 10.087412000 -3.230845000

N 2.104867000 7.045370000 3.264592000

C 1.838272000 7.055620000 1.837359000

C 0.403397000 7.498249000 1.594938000

O -0.539317000 6.933218000 2.148720000

C 2.073279000 5.672382000 1.256035000

H 2.514215000 7.762079000 1.355477000

H 1.563990000 4.929086000 1.868724000

H 1.682331000 5.634103000 0.239409000

H 3.142350000 5.461328000 1.242117000

H 1.379663000 7.309670000 3.915545000

H 3.016030000 6.774162000 3.606382000

N 0.241868000 8.518837000 0.764678000

H 1.058481000 8.945677000 0.325979000

C -1.082091000 9.036856000 0.472378000

C -1.435465000 8.900031000 -0.999087000

O -0.702647000 9.362577000 -1.881025000

C -1.193472000 10.512523000 0.884520000

C -2.596830000 11.027589000 0.613506000

C -0.844115000 10.655594000 2.353565000

H -1.807752000 8.465468000 1.051947000

H -0.485593000 11.095979000 0.295777000

H -2.664191000 12.074307000 0.909524000

H -3.316153000 10.441534000 1.186023000

H -2.818754000 10.935709000 -0.449723000

H -0.922864000 11.702999000 2.646288000

H 0.176032000 10.308014000 2.519777000

H -1.533523000 10.059098000 2.950719000

N -2.568856000 8.253510000 -1.249226000

C -3.047147000 8.038470000 -2.602365000

C -4.080667000 9.102507000 -2.922512000

O -4.875840000 9.489399000 -2.064588000

C -3.665668000 6.641470000 -2.729577000

C -4.123691000 6.273185000 -4.139155000

C -4.337197000 4.764363000 -4.276025000

C -4.564054000 4.367443000 -5.726295000

N -4.369690000 2.907105000 -5.963437000

H -4.954862000 2.354577000 -5.337390000

H -3.450942000 2.604088000 -5.641132000

H -2.212543000 8.126783000 -3.297588000

H -4.529830000 6.591313000 -2.067811000

H -2.932046000 5.905911000 -2.398970000

H -3.364721000 6.591448000 -4.853787000

H -5.059355000 6.788671000 -4.358135000

H -3.455857000 4.245807000 -3.899964000

H -5.204513000 4.471223000 -3.684920000

H -5.583729000 4.632386000 -6.004532000

H -3.870425000 4.923574000 -6.357372000

H -3.118787000 7.897674000 -0.466970000

O -4.125578000 9.588364000 -4.060816000

N -13.060499000 -0.075489000 -2.440919000

C -11.654863000 -0.140576000 -2.067681000

C -11.437344000 0.345415000 -0.646414000

O -10.557276000 -0.145257000 0.048843000

C -10.804171000 0.704008000 -3.022514000

C -9.376621000 0.950121000 -2.526657000

C -8.447083000 1.546132000 -3.589066000

O -7.311677000 1.930203000 -3.246053000

O -8.837304000 1.625736000 -4.768809000

H -11.325144000 -1.177153000 -2.134119000

H -10.750293000 0.188830000 -3.981065000

H -11.294545000 1.666692000 -3.169834000

H -9.420312000 1.638621000 -1.683170000

H -8.956324000 0.003706000 -2.185029000

H -13.352735000 -0.368948000 -3.361379000

H -13.194615000 -1.016031000 -2.098560000

N -12.245779000 1.305497000 -0.212432000

H -12.976295000 1.663538000 -0.827564000

C -12.104503000 1.855480000 1.129040000

C -12.707015000 0.967793000 2.212354000

O -12.100583000 0.772929000 3.271239000

C -12.728798000 3.269313000 1.218038000

C -11.951957000 4.233468000 0.322093000

C -12.688559000 3.777385000 2.651727000

C -12.597665000 5.585391000 0.196302000

H -11.039122000 1.951534000 1.335406000

H -13.764565000 3.225376000 0.882533000

H -11.874552000 3.793828000 -0.672299000

H -10.948564000 4.359982000 0.729026000

H -13.241261000 3.093247000 3.295363000

H -13.142259000 4.767175000 2.698660000

H -11.653833000 3.836016000 2.987216000

H -11.993044000 6.218353000 -0.453201000

H -12.673810000 6.043962000 1.182091000

H -13.594079000 5.473914000 -0.230102000

O -13.798869000 1.468953000 2.515379000

N -13.084047000 -2.149237000 2.035944000

H -13.113361000 -1.618295000 1.165450000

C -12.290659000 -3.367490000 2.115296000

C -11.085264000 -3.103221000 3.007033000

O -10.732194000 -3.927491000 3.845042000

C -11.813264000 -3.836091000 0.719711000

C -12.993537000 -4.430078000 -0.049795000

C -10.699525000 -4.872317000 0.854879000

C -12.594076000 -5.190484000 -1.291413000

H -12.896432000 -4.154554000 2.563980000

H -11.430638000 -2.975808000 0.170049000

H -13.527377000 -5.111225000 0.613645000

H -13.666879000 -3.621665000 -0.336224000

H -10.068351000 -4.618665000 1.705842000

H -11.137551000 -5.858706000 1.008542000

H -10.098005000 -4.879775000 -0.053561000

H -13.430117000 -5.210032000 -1.991049000

H -12.322950000 -6.210980000 -1.020246000

H -11.740030000 -4.699840000 -1.759399000

H -13.603910000 -1.830943000 2.840397000

N -10.465748000 -1.941941000 2.837374000

C -9.309184000 -1.581050000 3.644232000

C -9.668819000 -1.492190000 5.125856000

O -8.878860000 -1.873584000 5.987475000

C -8.719295000 -0.253846000 3.155623000

C -7.703390000 -0.440468000 2.029933000

O -6.612997000 -0.973910000 2.243663000

N -8.059091000 -0.005233000 0.828916000

H -7.381125000 -0.129532000 0.077186000

H -8.961540000 0.436023000 0.652076000

H -8.552874000 -2.355708000 3.522520000

H -9.531476000 0.375726000 2.792198000

H -8.231419000 0.245380000 3.992712000

H -10.805820000 -1.291103000 2.128970000

O -10.726193000 -2.116405000 5.289020000

N 0.037703000 -7.012176000 4.989466000

C -0.938914000 -6.277462000 4.203723000

C -0.725807000 -4.818882000 4.539733000

O -0.754383000 -4.447797000 5.709240000

C -2.381936000 -6.645403000 4.590408000

C -3.362779000 -5.867518000 3.725973000

C -2.602984000 -8.126268000 4.432007000

H -0.775894000 -6.448958000 3.139834000

H -2.544255000 -6.376085000 5.633698000

H -3.189755000 -4.798968000 3.851588000

H -3.218337000 -6.137836000 2.679613000

H -4.382522000 -6.109102000 4.026641000

H -1.893046000 -8.669029000 5.057032000

H -2.455666000 -8.405430000 3.389040000

H -3.620018000 -8.376164000 4.735238000

H 0.671959000 -6.515950000 5.598918000

H 0.594841000 -7.558969000 4.349147000

N -0.494583000 -3.990725000 3.529020000

H -0.465429000 -4.349629000 2.574345000

C -0.282593000 -2.573357000 3.777443000

C -1.321539000 -2.102329000 4.781576000

O -2.492826000 -2.466012000 4.690061000

C -0.392658000 -1.772229000 2.480773000

C -0.442736000 -0.279944000 2.785259000

C 0.801126000 -2.093038000 1.600740000

H 0.712188000 -2.431034000 4.201047000

H -1.306252000 -2.060252000 1.960805000

H -0.074544000 0.279403000 1.925354000

H 0.182377000 -0.064883000 3.652470000

H -1.471386000 0.013677000 2.996880000

H 1.711330000 -2.069835000 2.199900000

H 0.676210000 -3.085213000 1.167520000

H 0.873339000 -1.354192000 0.802035000

N -0.887569000 -1.309857000 5.753449000

C -1.792361000 -0.832749000 6.792332000

C -2.619361000 0.374445000 6.367007000

O -2.115392000 1.294958000 5.727964000

C -0.994999000 -0.509967000 8.061241000

C -1.843097000 -0.390391000 9.311410000

C -0.997774000 -0.025072000 10.522051000

C 0.028824000 -1.107023000 10.841752000

N 0.781895000 -0.788077000 12.097835000

H 1.468234000 -1.512007000 12.311706000

H 0.150572000 -0.627088000 12.882959000

H -2.482404000 -1.642214000 7.029230000

H -0.265124000 -1.304167000 8.217755000

H -0.462215000 0.428699000 7.909769000

H -2.336094000 -1.344029000 9.498293000

H -2.599811000 0.379382000 9.157894000

H -0.474844000 0.908892000 10.317760000

H -1.651013000 0.113008000 11.383620000

H 0.734632000 -1.183083000 10.014154000

H -0.483209000 -2.061807000 10.964283000

H 0.092852000 -1.030699000 5.773783000

O -3.818816000 0.428272000 6.670147000

N -2.266705000 5.251867000 3.739833000

C -1.787985000 3.885952000 3.891416000

C -0.352170000 3.841439000 4.382566000

O 0.383016000 4.816953000 4.264609000

C -1.893843000 3.175757000 2.545012000

C -3.285407000 3.330871000 1.931534000

C -3.170593000 3.673620000 0.461149000

C -4.087693000 2.059242000 2.163595000

H -2.422286000 3.369650000 4.611953000

H -1.687087000 2.115441000 2.687995000

H -1.154221000 3.594152000 1.862258000

H -3.793103000 4.153448000 2.436233000

H -4.167261000 3.781942000 0.033435000

H -2.623713000 4.609449000 0.348384000

H -2.638086000 2.876471000 -0.057543000

H -5.079470000 2.170554000 1.725974000

H -3.576958000 1.217525000 1.696611000

H -4.180371000 1.878923000 3.234951000

H -3.077228000 5.344138000 4.335099000

H -2.575070000 5.363038000 2.784253000

N 0.043326000 2.696665000 4.923713000

H -0.615721000 1.919690000 4.977677000

C 1.392177000 2.518888000 5.442138000

C 2.447552000 2.820857000 4.393774000

O 2.260534000 2.541315000 3.211791000

C 1.595318000 1.081055000 5.934225000

C 2.929903000 0.864964000 6.634514000

C 3.313689000 -0.598910000 6.754483000

O 2.522088000 -1.395023000 7.309376000

O 4.418523000 -0.950417000 6.292977000

H 1.533572000 3.197508000 6.283062000

H 1.543451000 0.411283000 5.074958000

H 0.790665000 0.828808000 6.625639000

H 3.705142000 1.380743000 6.068387000

H 2.877149000 1.297579000 7.633907000

N 3.560014000 3.396062000 4.826457000

H 3.657874000 3.638513000 5.812316000

C 4.639476000 3.682108000 3.900352000

C 5.599691000 2.501955000 3.916390000

O 5.890544000 1.949070000 4.979223000

C 5.400430000 4.942640000 4.300561000

C 6.648883000 5.123176000 3.471657000

C 6.573888000 5.191840000 2.087897000

C 7.901390000 5.178471000 4.063547000

C 7.713806000 5.307774000 1.311689000

C 9.052080000 5.294905000 3.295129000

C 8.949468000 5.356785000 1.917391000

O 10.083413000 5.452556000 1.137902000

H 10.935594000 5.486284000 1.555539000

H 4.233652000 3.806120000 2.896702000

H 5.680917000 4.867841000 5.351272000

H 4.753210000 5.809348000 4.164240000

H 5.606929000 5.153783000 1.607448000

H 7.983666000 5.129728000 5.139450000

H 7.634049000 5.359282000 0.235845000

H 10.021152000 5.336576000 3.770243000

N 6.088644000 2.114581000 2.742719000

H 5.809216000 2.611480000 1.897060000

C 7.016276000 0.994191000 2.639783000

C 8.320436000 1.469612000 1.999360000

O 8.453930000 1.512132000 0.779776000

C 6.389592000 -0.141387000 1.807268000

S 4.819877000 -0.846533000 2.455098000

H 7.230763000 0.620390000 3.641147000

H 6.192628000 0.247135000 0.808197000

H 7.118895000 -0.947591000 1.723772000

H 4.741732000 -0.626553000 3.770237000

N 9.281235000 1.833599000 2.836690000

H 9.113526000 1.773039000 3.840838000

C 10.571794000 2.317487000 2.360986000

C 11.330893000 1.235414000 1.603478000

O 12.269527000 1.526637000 0.854705000

C 11.418693000 2.778166000 3.544458000

C 11.786474000 1.670299000 4.349652000

O 12.473374000 2.044394000 5.309904000

H 10.406981000 3.164275000 1.694679000

H 12.319630000 3.266659000 3.172860000

H 10.845573000 3.486161000 4.144286000

O 11.465677000 0.495204000 4.124077000

N 10.919387000 -0.011112000 1.814054000

H 10.133824000 -0.179590000 2.443273000

C 11.565980000 -1.134800000 1.166911000

C 11.486972000 -1.092005000 -0.339794000

O 12.435273000 -1.462811000 -1.020670000

H 11.088502000 -2.052459000 1.511010000

H 12.614849000 -1.153676000 1.463175000

N 10.357553000 -0.637005000 -0.866891000

H 9.598578000 -0.335263000 -0.256234000

C 10.195786000 -0.565776000 -2.307449000

C 9.340886000 -1.708600000 -2.802489000

O 8.659178000 -2.350557000 -2.016803000

H 9.717903000 0.378208000 -2.568009000

H 11.175853000 -0.616632000 -2.782596000

N 9.371207000 -1.973603000 -4.100721000

C 8.582142000 -3.063303000 -4.659272000

C 9.409518000 -4.338081000 -4.769876000

O 10.620790000 -4.293229000 -4.966434000

C 8.046183000 -2.676908000 -6.038410000

C 7.362392000 -1.322344000 -6.069350000

C 6.642766000 -1.044989000 -7.375433000

O 7.128549000 -1.505823000 -8.430514000

O 5.597437000 -0.352919000 -7.341277000

H 7.736388000 -3.253914000 -3.998412000

H 8.881123000 -2.656428000 -6.738599000

H 7.334281000 -3.436195000 -6.363543000

H 8.117509000 -0.549837000 -5.919062000

H 6.641879000 -1.273132000 -5.253582000

H 9.955036000 -1.407412000 -4.716545000

O 8.850684000 -5.438365000 -4.660060000

N 3.445705000 -6.039423000 -4.460096000

C 3.989065000 -6.614316000 -3.239283000

C 5.111668000 -5.698219000 -2.774606000

O 6.159576000 -5.596684000 -3.405460000

C 4.496202000 -8.034123000 -3.498855000

C 3.362691000 -8.963982000 -3.954498000

C 3.900021000 -10.321177000 -4.367917000

C 2.347014000 -9.094818000 -2.826200000

H 3.209832000 -6.643506000 -2.477581000

H 5.259880000 -8.000540000 -4.276298000

H 4.936099000 -8.429258000 -2.582934000

H 2.868654000 -8.511308000 -4.814267000

H 4.622155000 -10.195567000 -5.174430000

H 4.386110000 -10.793953000 -3.514607000

H 3.077900000 -10.948513000 -4.710459000

H 1.537312000 -9.753636000 -3.139609000

H 1.942766000 -8.111249000 -2.586039000

H 2.834515000 -9.512339000 -1.945016000

H 3.832232000 -5.182617000 -4.829709000

H 3.545643000 -6.739523000 -5.181404000

N 4.870588000 -5.006898000 -1.669263000

H 3.985206000 -5.133468000 -1.178622000

C 5.849596000 -4.073567000 -1.150250000

C 6.689713000 -4.621718000 -0.010470000

O 6.393789000 -5.661688000 0.560864000

C 5.153033000 -2.783991000 -0.715623000

C 4.331722000 -2.155552000 -1.845102000

C 3.805182000 -0.820279000 -1.391284000

C 5.187126000 -1.994078000 -3.086575000

H 6.527451000 -3.821326000 -1.965532000

H 4.487439000 -3.009823000 0.117721000

H 5.905621000 -2.068431000 -0.384512000

H 3.489966000 -2.808253000 -2.077099000

H 3.219697000 -0.369059000 -2.192665000

H 3.173774000 -0.958161000 -0.513305000

H 4.640392000 -0.166386000 -1.139144000

H 4.798013000 -1.176334000 -3.692698000

H 6.213664000 -1.772607000 -2.794501000

H 5.166095000 -2.917929000 -3.665521000

N 7.749059000 -3.899797000 0.308851000

C 8.653905000 -4.295361000 1.364732000

C 8.879494000 -3.077037000 2.248920000

O 8.767777000 -1.946971000 1.782251000

C 9.980064000 -4.748732000 0.750174000

C 9.893250000 -5.864358000 -0.296050000

C 11.231839000 -6.038854000 -0.983821000

C 9.456874000 -7.151969000 0.370130000

H 8.215886000 -5.105160000 1.948215000

H 10.439944000 -3.882346000 0.274408000

H 10.633728000 -5.084592000 1.555126000

H 9.149383000 -5.586418000 -1.042893000

H 9.395450000 -7.944251000 -0.375381000

H 8.479269000 -7.009354000 0.830078000

H 10.181240000 -7.428821000 1.136039000

H 11.158461000 -6.834430000 -1.725427000

H 11.510984000 -5.107177000 -1.476609000

H 11.988611000 -6.299826000 -0.244361000

H 7.934164000 -3.037830000 -0.204867000

O 9.176413000 -3.226330000 3.442525000

N 1.228975000 -7.024015000 1.053672000

C 0.346799000 -7.031468000 -0.099846000

C -0.270090000 -5.626934000 -0.160402000

O -0.627221000 -5.065940000 0.875164000

C -0.758468000 -8.101710000 0.061909000

C -0.108566000 -9.487679000 0.146990000

C -1.726785000 -8.049626000 -1.116868000

C -1.080168000 -10.620885000 0.278906000

H 0.921198000 -7.224390000 -1.005987000

H -1.308565000 -7.909566000 0.983142000

H 0.551048000 -9.501558000 1.014821000

H 0.491472000 -9.646311000 -0.749569000

H -2.183501000 -7.061450000 -1.169118000

H -1.184749000 -8.248469000 -2.040773000

H -2.503998000 -8.802364000 -0.982696000

H -0.612231000 -11.440455000 0.824668000

H -1.963255000 -10.281197000 0.820772000

H -1.373203000 -10.965246000 -0.712528000

H 1.320160000 -6.189397000 1.614373000

H 2.152500000 -7.278047000 0.733162000

N -0.384872000 -5.048364000 -1.352958000

H -0.079239000 -5.543177000 -2.191278000

C -0.946905000 -3.708662000 -1.463642000

C -1.771833000 -3.490850000 -2.719256000

O -1.568497000 -4.162556000 -3.726173000

C 0.167175000 -2.657102000 -1.417533000

O 0.564641000 -2.251592000 -2.719045000

H 1.254919000 -1.599985000 -2.690958000

H -1.596977000 -3.545587000 -0.603564000

H 1.028836000 -3.079380000 -0.901197000

H -0.187260000 -1.786448000 -0.865868000

N -2.689707000 -2.528908000 -2.641463000

H -2.774706000 -2.021669000 -1.760025000

C -3.583198000 -2.151908000 -3.735598000

C -4.825277000 -3.031826000 -3.814687000

O -4.842959000 -4.058858000 -4.496509000

C -2.853317000 -2.155092000 -5.083791000

C -3.605160000 -1.358737000 -6.152216000

O -3.053566000 -1.152515000 -7.260642000

O -4.751676000 -0.935869000 -5.878709000

H -3.917425000 -1.131580000 -3.547387000

H -1.865431000 -1.714876000 -4.948573000

H -2.739226000 -3.184524000 -5.422687000

N -5.874571000 -2.616438000 -3.112672000

H -5.809302000 -1.751153000 -2.576504000

C -7.108970000 -3.372666000 -3.097236000

C -8.201652000 -2.719725000 -3.922864000

O -9.394143000 -2.907390000 -3.667376000

C -7.556154000 -3.581875000 -1.652805000

C -6.584037000 -4.386240000 -0.851748000

C -5.460842000 -3.792250000 -0.302006000

C -6.742208000 -5.759850000 -0.726478000

C -4.517981000 -4.550398000 0.347853000

C -5.791431000 -6.521312000 -0.073106000

C -4.683409000 -5.917459000 0.459505000

H -6.905463000 -4.352846000 -3.528538000

H -8.514973000 -4.100602000 -1.657466000

H -7.683834000 -2.608454000 -1.178438000

H -5.324899000 -2.724445000 -0.384995000

H -7.616277000 -6.238031000 -1.143720000

H -3.645691000 -4.076894000 0.772091000

H -5.922044000 -7.589071000 0.017272000

H -3.938338000 -6.511414000 0.967610000

N -7.773157000 -1.957945000 -4.927798000

C -8.707886000 -1.293189000 -5.816132000

C -9.458821000 -2.296057000 -6.678406000

O -10.451473000 -1.949878000 -7.302665000

H -9.426062000 -0.730301000 -5.219867000

H -8.161292000 -0.605266000 -6.460935000

H -6.770550000 -1.840047000 -5.075402000

O -9.063228000 -3.467479000 -6.748081000

C 4.469066000 2.432681000 -3.789124000

C 5.834850000 2.664135000 -3.691292000

C 6.427113000 2.934315000 -2.428005000

C 5.683758000 2.975403000 -1.261453000

C 4.298817000 2.761955000 -1.361577000

N 3.275747000 2.719559000 -0.441150000

H 3.302770000 2.780461000 0.565871000

N 2.080697000 2.432456000 -1.007077000

C 2.320352000 2.310951000 -2.297399000

C 3.701319000 2.501842000 -2.617375000

N 6.642922000 2.642422000 -4.852307000

C 6.222205000 2.971593000 -6.132339000

C 7.280774000 2.811761000 -6.991571000

C 8.394275000 2.365564000 -6.220344000

C 7.978487000 2.273147000 -4.914810000

S 1.108024000 1.797333000 -3.502013000

O 0.920971000 2.893003000 -4.462410000

O 1.590655000 0.499521000 -4.006155000

C -0.450598000 1.532046000 -2.642225000

C -1.595131000 1.903822000 -3.346804000

C -2.843499000 1.541069000 -2.849857000

C -2.971432000 0.832089000 -1.651057000

C -1.805996000 0.497007000 -0.945623000

C -4.349458000 0.511016000 -1.121635000

C -0.545630000 0.837350000 -1.435787000

H 4.009286000 2.178293000 -4.735853000

H 7.492006000 3.130027000 -2.375877000

H 6.186476000 3.176890000 -0.323485000

H 5.221847000 3.334567000 -6.308227000

H 7.262977000 3.007649000 -8.054268000

H 9.384880000 2.133636000 -6.585259000

H 8.501718000 1.946705000 -4.031034000

H -1.490548000 2.461209000 -4.270381000

H -3.743039000 1.809976000 -3.390218000

H -1.885869000 -0.020496000 0.007720000

H 0.349393000 0.590525000 -0.877888000

H -5.059446000 0.362641000 -1.939902000

H -4.354587000 -0.366810000 -0.468153000

H -4.751659000 1.343971000 -0.538021000

H -8.756689000 -4.375770000 -6.802382000

H -4.160621000 9.965287000 -4.943280000

H 1.723825000 10.808539000 -2.604747000

H 13.005960000 2.334155000 6.054458000

H 1.914871000 -2.005204000 7.734326000

H -11.545697000 -2.600408000 5.415901000

H -9.138163000 1.687469000 -5.678069000

H -2.631396000 -0.995274000 -8.108275000

H 4.797880000 0.176771000 -7.314756000

H 8.418136000 -6.291187000 -4.574917000

H 9.406769000 -3.342045000 4.367511000

H -14.686562000 1.124594000 2.387694000

H -4.748469000 0.470194000 6.905438000

Azole **3** with surrounded residues

C -2.024000000 4.919000000 -22.123000000

C -3.016000000 6.041000000 -21.881000000

C -3.077000000 7.058000000 -23.034000000

C -4.328000000 7.939000000 -22.876000000

C -1.822000000 7.936000000 -23.035000000

H -1.065000000 5.312000000 -22.538000000

H -4.023000000 5.627000000 -21.659000000

H -2.725000000 6.567000000 -20.936000000

H -3.151000000 6.515000000 -24.003000000

H -1.884000000 8.736000000 -23.779000000

H -0.917000000 7.359000000 -23.244000000

H -1.666000000 8.409000000 -22.051000000

H -4.378000000 8.688000000 -23.670000000

H -5.236000000 7.331000000 -22.915000000

H -4.308000000 8.462000000 -21.909000000

C -7.118000000 3.704000000 -21.617000000

C -6.629000000 4.571000000 -20.438000000

C -6.799000000 6.050000000 -20.796000000

C -7.403000000 4.273000000 -19.151000000

H -8.187000000 3.909000000 -21.874000000

H -5.529000000 4.379000000 -20.273000000

H -7.127000000 3.325000000 -18.688000000

H -8.486000000 4.313000000 -19.302000000

H -7.175000000 5.051000000 -18.392000000

H -7.851000000 6.359000000 -20.761000000

H -6.428000000 6.264000000 -21.806000000

H -6.242000000 6.684000000 -20.093000000

C -7.645000000 9.791000000 -23.909000000

C -9.042000000 9.145000000 -23.917000000

O -10.025000000 9.834000000 -23.665000000

C -7.202000000 10.034000000 -22.485000000

H -6.882000000 9.170000000 -24.459000000

H -7.800000000 10.807000000 -21.968000000

H -7.297000000 9.122000000 -21.862000000

H -6.142000000 10.334000000 -22.405000000

N -9.116000000 7.867000000 -24.345000000

H -8.296000000 7.239000000 -24.351000000

C -10.398000000 7.145000000 -24.225000000

C -10.238000000 6.317000000 -22.931000000

O -9.108000000 6.029000000 -22.566000000

C -10.587000000 6.189000000 -25.431000000

C -11.920000000 5.453000000 -25.313000000

C -10.544000000 6.982000000 -26.742000000

H -11.250000000 7.863000000 -24.163000000

H -9.753000000 5.436000000 -25.429000000

H -10.709000000 6.319000000 -27.601000000

H -11.320000000 7.753000000 -26.772000000

H -9.577000000 7.469000000 -26.892000000

H -12.104000000 4.823000000 -26.191000000

H -11.958000000 4.765000000 -24.447000000

H -12.772000000 6.132000000 -25.217000000

N -11.351000000 6.016000000 -22.222000000

C -11.260000000 4.928000000 -21.227000000

C -11.705000000 5.459000000 -19.859000000

C -11.509000000 4.384000000 -18.785000000

C -11.543000000 5.011000000 -17.388000000

C -11.281000000 3.933000000 -16.334000000

H -10.228000000 4.515000000 -21.177000000

H -12.772000000 5.762000000 -19.903000000

H -11.128000000 6.371000000 -19.614000000

H -12.296000000 3.607000000 -18.902000000

H -10.553000000 3.853000000 -18.951000000

H -12.533000000 5.498000000 -17.212000000

H -10.795000000 5.828000000 -17.333000000

H -12.217000000 3.353000000 -16.137000000

H -10.507000000 3.213000000 -16.675000000

H -12.299000000 6.126000000 -22.607000000

C -18.323000000 8.203000000 -12.467000000

C -17.426000000 7.076000000 -12.973000000

C -16.233000000 7.669000000 -13.726000000

H -17.808000000 8.744000000 -11.624000000

H -17.003000000 6.474000000 -12.127000000

H -17.962000000 6.375000000 -13.635000000

H -15.739000000 8.455000000 -13.115000000

H -16.533000000 8.123000000 -14.680000000

C -17.522000000 14.057000000 -14.711000000

C -17.284000000 13.091000000 -15.862000000

C -16.101000000 12.159000000 -15.683000000

O -14.951000000 12.567000000 -15.656000000

N -16.372000000 10.816000000 -15.699000000

H -15.633000000 10.154000000 -15.516000000

H -17.297000000 10.465000000 -15.448000000

H -16.571000000 14.427000000 -14.262000000

H -18.214000000 12.547000000 -16.129000000

H -17.067000000 13.705000000 -16.780000000

C -8.837000000 16.237000000 -16.292000000

C -8.578000000 14.731000000 -16.334000000

C -9.209000000 14.134000000 -17.597000000

C -7.069000000 14.472000000 -16.362000000

H -8.070000000 16.793000000 -16.903000000

H -9.042000000 14.232000000 -15.450000000

H -8.974000000 13.067000000 -17.691000000

H -8.834000000 14.643000000 -18.495000000

H -10.303000000 14.252000000 -17.583000000

H -6.597000000 14.961000000 -17.236000000

H -6.846000000 13.404000000 -16.487000000

H -6.546000000 14.843000000 -15.481000000

C -11.295000000 12.685000000 -21.196000000

C -10.183000000 13.496000000 -21.869000000

O -9.783000000 13.150000000 -22.978000000

C -10.769000000 11.839000000 -20.042000000

C -11.927000000 11.191000000 -19.244000000

C -11.475000000 9.826000000 -18.728000000

C -12.355000000 12.113000000 -18.105000000

H -12.125000000 13.364000000 -20.846000000

H -10.139000000 12.452000000 -19.356000000

H -10.048000000 11.073000000 -20.396000000

H -12.814000000 11.028000000 -19.905000000

H -12.188000000 9.417000000 -17.995000000

H -11.370000000 9.088000000 -19.527000000

H -10.506000000 9.891000000 -18.210000000

H -13.172000000 11.677000000 -17.504000000

H -12.719000000 13.085000000 -18.452000000

H -11.536000000 12.302000000 -17.399000000

N -9.771000000 14.621000000 -21.240000000

H -10.077000000 14.891000000 -20.300000000

C -8.517000000 15.240000000 -21.709000000

C -7.478000000 14.103000000 -21.816000000

O -7.696000000 13.027000000 -21.264000000

C -8.030000000 16.295000000 -20.700000000

C -7.118000000 17.309000000 -21.368000000

H -8.698000000 15.703000000 -22.715000000

H -7.509000000 15.818000000 -19.846000000

H -8.887000000 16.853000000 -20.234000000

H -7.644000000 17.922000000 -22.112000000

H -6.235000000 16.858000000 -21.850000000

N -6.410000000 14.318000000 -22.626000000

H -6.181000000 15.287000000 -22.929000000

C -5.235000000 13.430000000 -22.609000000

C -4.064000000 14.363000000 -22.168000000

O -4.087000000 15.520000000 -22.540000000

C -4.969000000 12.887000000 -24.036000000

C -3.501000000 12.704000000 -24.212000000

C -2.832000000 11.565000000 -23.743000000

C -2.753000000 13.775000000 -24.735000000

C -1.461000000 11.547000000 -23.643000000

C -1.386000000 13.792000000 -24.639000000

C -0.705000000 12.724000000 -23.963000000

O 0.503000000 12.851000000 -23.625000000

H -5.381000000 12.593000000 -21.889000000

H -5.516000000 11.935000000 -24.189000000

H -5.353000000 13.608000000 -24.787000000

H -3.408000000 10.697000000 -23.427000000

H -3.275000000 14.624000000 -25.174000000

H -0.916000000 10.696000000 -23.254000000

H -0.794000000 14.623000000 -24.995000000

N -3.162000000 13.853000000 -21.284000000

C -2.141000000 14.734000000 -20.704000000

C -2.377000000 14.901000000 -19.204000000

S -4.112000000 15.399000000 -18.837000000

H -2.164000000 15.741000000 -21.224000000

H -2.193000000 13.952000000 -18.662000000

H -1.670000000 15.641000000 -18.776000000

H -4.224000000 16.616000000 -19.460000000

H -3.069000000 12.864000000 -21.104000000

C 1.885000000 10.957000000 -14.037000000

C 1.764000000 9.507000000 -13.600000000

C 0.896000000 8.717000000 -14.564000000

H 0.909000000 11.486000000 -13.979000000

H 2.753000000 9.016000000 -13.473000000

H 1.342000000 9.458000000 -12.570000000

H 1.388000000 8.499000000 -15.524000000

H -0.067000000 9.208000000 -14.768000000

C -1.502000000 13.912000000 -14.290000000

C -2.489000000 13.612000000 -15.402000000

C -3.034000000 12.173000000 -15.419000000

C -4.052000000 12.040000000 -16.557000000

C -1.916000000 11.147000000 -15.618000000

H -0.641000000 13.200000000 -14.294000000

H -3.319000000 14.342000000 -15.405000000

H -1.965000000 13.819000000 -16.374000000

H -3.559000000 11.957000000 -14.459000000

H -2.326000000 10.132000000 -15.722000000

H -1.346000000 11.350000000 -16.534000000

H -1.223000000 11.120000000 -14.772000000

H -4.565000000 11.070000000 -16.517000000

H -3.568000000 12.109000000 -17.541000000

H -4.816000000 12.819000000 -16.521000000

C -7.842000000 12.412000000 -12.730000000

C -8.296000000 11.090000000 -12.004000000

O -7.786000000 10.894000000 -10.920000000

C -6.745000000 12.071000000 -13.761000000

O -5.511000000 12.016000000 -13.072000000

H -5.228000000 11.065000000 -12.982000000

H -8.716000000 12.916000000 -13.196000000

H -6.952000000 11.098000000 -14.277000000

H -6.611000000 12.859000000 -14.521000000

N -9.109000000 10.331000000 -12.766000000

H -9.530000000 10.724000000 -13.603000000

C -9.857000000 9.202000000 -12.173000000

C -10.924000000 9.876000000 -11.271000000

O -10.675000000 10.999000000 -10.840000000

C -8.936000000 8.246000000 -11.452000000

H -10.372000000 8.661000000 -13.023000000

H -7.904000000 8.280000000 -11.846000000

H -8.843000000 8.476000000 -10.374000000

N -12.114000000 9.250000000 -11.200000000

C -13.267000000 9.823000000 -10.490000000

C -13.978000000 10.935000000 -11.285000000

C -13.169000000 12.164000000 -11.564000000

C -13.199000000 12.752000000 -12.831000000

C -12.402000000 12.772000000 -10.559000000

C -12.464000000 13.906000000 -13.101000000

C -11.670000000 13.924000000 -10.827000000

C -11.694000000 14.495000000 -12.102000000

H -12.967000000 10.191000000 -9.484000000

H -14.378000000 10.497000000 -12.226000000

H -14.891000000 11.212000000 -10.708000000

H -13.809000000 12.319000000 -13.627000000

H -12.350000000 12.315000000 -9.571000000

H -12.514000000 14.356000000 -14.093000000

H -11.082000000 14.379000000 -10.039000000

H -11.149000000 15.407000000 -12.300000000

H -12.174000000 8.194000000 -11.369000000

C -5.653818000 8.934344000 -17.872450000

C -4.280329000 8.797959000 -18.247389000

C -4.133382000 9.727268000 -19.303875000

N -5.371355000 10.294111000 -19.481410000

N -6.287240000 9.827513000 -18.606458000

C -2.885466000 9.941682000 -19.910475000

C -1.814594000 9.200654000 -19.450044000

C -1.965952000 8.239875000 -18.418671000

C -3.188113000 8.041883000 -17.794800000

H -5.666530000 11.018875000 -20.115395000

S -6.389658000 8.215803000 -16.416933000

O -6.424763000 6.752874000 -16.579307000

O -5.646908000 8.779244000 -15.276325000

C -8.076934000 8.826075000 -16.345791000

C -8.364041000 10.190588000 -16.338729000

C -9.648541000 10.603252000 -15.998085000

C -10.649617000 9.677793000 -15.645858000

C -10.347184000 8.311072000 -15.730962000

C -12.005138000 10.146072000 -15.163335000

C -9.067311000 7.883356000 -16.078574000

N -0.834579000 7.465284000 -18.055315000

C -0.814893000 6.242409000 -17.443842000

C 0.506440000 5.869080000 -17.336720000

C 1.223789000 6.936029000 -17.924258000

N 0.417780000 7.898900000 -18.359468000

H -2.748708000 10.641459000 -20.725453000

H -0.828078000 9.328617000 -19.877066000

H -3.306160000 7.348089000 -16.971880000

H -7.600769000 10.919228000 -16.580409000

H -9.860965000 11.665804000 -15.941242000

H -11.111644000 7.577220000 -15.491214000

H -8.803878000 6.834656000 -16.127805000

H -1.722847000 5.731242000 -17.165134000

H 0.893456000 4.957759000 -16.906942000

H 2.293667000 7.044041000 -18.041673000

H -12.080454000 11.235132000 -15.187828000

H -12.822731000 9.734339000 -15.762838000

H -12.200297000 9.828165000 -14.133259000

N -2.564000000 3.856000000 -23.064000000

C -1.721000000 4.207000000 -20.769000000

O -0.655000000 4.475000000 -20.213000000

H -1.950000000 3.731000000 -23.863000000

H -2.684000000 2.946000000 -22.563000000

O -2.504000000 3.408000000 -20.237000000

N -6.914000000 2.232000000 -21.285000000

C -6.212000000 3.896000000 -22.850000000

O -4.998000000 3.971000000 -22.680000000

H -7.228000000 2.050000000 -20.312000000

H -7.412000000 1.580000000 -21.914000000

O -6.685000000 4.051000000 -23.984000000

N -7.810000000 11.109000000 -24.650000000

H -6.925000000 11.669000000 -24.650000000

H -8.118000000 10.962000000 -25.620000000

C -12.247000000 3.846000000 -21.763000000

O -13.295000000 4.220000000 -22.292000000

N -10.825000000 4.567000000 -15.042000000

H -10.614000000 3.860000000 -14.312000000

H -9.983000000 5.155000000 -15.157000000

O -12.039000000 2.635000000 -21.608000000

N -19.659000000 7.628000000 -11.972000000

C -18.674000000 9.165000000 -13.615000000

O -17.964000000 10.150000000 -13.771000000

C -15.176000000 6.582000000 -13.982000000

O -14.245000000 6.852000000 -14.766000000

O -15.229000000 5.495000000 -13.377000000

O -19.607000000 8.949000000 -14.400000000

H -20.125000000 8.238000000 -11.291000000

H -19.490000000 6.707000000 -11.529000000

N -18.408000000 13.436000000 -13.639000000

C -18.350000000 15.230000000 -15.328000000

O -17.787000000 16.280000000 -15.632000000

H -19.418000000 13.613000000 -13.873000000

H -18.229000000 13.768000000 -12.685000000

O -19.552000000 15.081000000 -15.588000000

N -8.842000000 16.763000000 -14.873000000

C -10.236000000 16.661000000 -16.839000000

O -11.274000000 16.583000000 -16.184000000

H -8.264000000 16.197000000 -14.250000000

H -8.485000000 17.738000000 -14.865000000

O -10.277000000 17.078000000 -18.005000000

N -11.852000000 11.728000000 -22.235000000

H -12.592000000 11.136000000 -21.772000000

H -12.264000000 12.202000000 -23.051000000

C -6.586000000 18.273000000 -20.262000000

O -7.384000000 19.025000000 -19.657000000

O -5.359000000 18.261000000 -20.035000000

C -0.785000000 14.060000000 -21.008000000

O -0.324000000 13.047000000 -20.490000000

O -0.141000000 14.634000000 -21.897000000

N 2.386000000 11.001000000 -15.462000000

C 2.933000000 11.709000000 -13.173000000

O 4.133000000 11.724000000 -13.433000000

C 0.578000000 7.333000000 -13.943000000

O 1.397000000 6.783000000 -13.176000000

O -0.520000000 6.819000000 -14.264000000

O 2.522000000 12.292000000 -12.160000000

H 1.922000000 10.271000000 -16.034000000

H 2.214000000 11.925000000 -15.898000000

N -2.154000000 13.882000000 -12.921000000

C -0.940000000 15.358000000 -14.520000000

O -1.238000000 16.336000000 -13.849000000

O -0.166000000 15.495000000 -15.477000000

H -1.547000000 13.400000000 -12.230000000

H -3.055000000 13.393000000 -12.956000000

N -7.232000000 13.290000000 -11.662000000

H -6.296000000 13.566000000 -12.000000000

H -7.780000000 14.132000000 -11.444000000

C -9.461000000 6.806000000 -11.586000000

O -8.656000000 5.868000000 -11.368000000

O -10.685000000 6.617000000 -11.771000000

C -14.265000000 8.614000000 -10.320000000

O -15.444000000 8.727000000 -9.974000000

O -13.806000000 7.488000000 -10.558000000

H 2.204000000 12.744000000 -11.375000000

H 2.026000000 6.360000000 -12.587000000

H 0.434000000 15.601000000 -16.219000000

H -7.996000000 19.601000000 -19.193000000

H -12.084000000 16.522000000 -15.673000000

H -15.270000000 4.657000000 -12.911000000

H -11.878000000 1.696000000 -21.488000000

H -7.052000000 4.171000000 -24.863000000

H -20.330000000 8.782000000 -15.009000000

H -14.131000000 6.598000000 -10.402000000

H -8.040000000 5.151000000 -11.201000000

H -0.147000000 15.589000000 -21.993000000

H 1.422000000 12.949000000 -23.368000000

H -3.111000000 2.789000000 -19.825000000

H -20.207000000 15.417000000 -16.204000000

Azole **4** with surrounded residues

N -2.564000000 3.856000000 -23.064000000

C -2.089000000 4.847000000 -22.104000000

C -1.721000000 4.207000000 -20.769000000

O -0.655000000 4.475000000 -20.213000000

C -3.161000000 5.910000000 -21.873000000

C -3.399000000 6.915000000 -22.996000000

C -4.670000000 7.698000000 -22.725000000

C -2.199000000 7.842000000 -23.096000000

H -1.202000000 5.331000000 -22.514000000

H -4.103000000 5.392000000 -21.692000000

H -2.900000000 6.464000000 -20.972000000

H -3.509000000 6.375000000 -23.937000000

H -4.583000000 8.694000000 -23.158000000

H -5.518000000 7.180000000 -23.173000000

H -4.823000000 7.782000000 -21.649000000

H -2.362000000 8.563000000 -23.897000000

H -2.069000000 8.371000000 -22.152000000

H -1.305000000 7.257000000 -23.311000000

H -2.020000000 3.018000000 -22.917000000

H -2.356000000 4.200000000 -23.991000000

N -2.611000000 3.366000000 -20.258000000

H -3.480000000 3.182000000 -20.760000000

C -2.358000000 2.706000000 -18.992000000

C -3.507000000 1.800000000 -18.602000000

O -4.587000000 2.270000000 -18.248000000

H -1.450000000 2.110000000 -19.078000000

H -2.218000000 3.460000000 -18.217000000

O -3.357000000 0.571000000 -18.640000000

N -6.914000000 2.232000000 -21.285000000

H -5.958000000 1.916000000 -21.121000000

C -7.141000000 3.614000000 -21.675000000

C -6.212000000 3.896000000 -22.850000000

O -4.998000000 3.971000000 -22.680000000

C -6.812000000 4.580000000 -20.522000000

C -7.120000000 6.006000000 -20.938000000

C -7.614000000 4.206000000 -19.290000000

H -8.178000000 3.746000000 -21.983000000

H -5.750000000 4.502000000 -20.289000000

H -8.115000000 6.280000000 -20.588000000

H -6.383000000 6.679000000 -20.500000000

H -7.083000000 6.084000000 -22.025000000

H -8.092000000 5.097000000 -18.882000000

H -6.949000000 3.776000000 -18.541000000

H -8.377000000 3.476000000 -19.561000000

H -7.296000000 1.651000000 -22.017000000

O -6.685000000 4.051000000 -23.984000000

N -7.810000000 11.109000000 -24.650000000

H -8.419000000 11.844000000 -24.292000000

C -7.667000000 9.874000000 -23.900000000

C -9.024000000 9.198000000 -23.777000000

O -9.997000000 9.801000000 -23.325000000

C -7.092000000 10.166000000 -22.525000000

H -6.987000000 9.211000000 -24.435000000

H -7.645000000 10.985000000 -22.066000000

H -7.175000000 9.277000000 -21.900000000

H -6.043000000 10.445000000 -22.622000000

H -6.888000000 11.505000000 -24.769000000

N -9.086000000 7.939000000 -24.187000000

H -8.246000000 7.484000000 -24.546000000

C -10.334000000 7.200000000 -24.132000000

C -10.241000000 5.992000000 -23.215000000

O -9.362000000 5.136000000 -23.368000000

C -10.757000000 6.739000000 -25.535000000

C -12.086000000 6.006000000 -25.467000000

C -10.854000000 7.941000000 -26.455000000

H -11.107000000 7.864000000 -23.744000000

H -10.000000000 6.058000000 -25.924000000

H -12.376000000 5.684000000 -26.467000000

H -12.849000000 6.673000000 -25.065000000

H -11.988000000 5.135000000 -24.819000000

H -11.154000000 7.614000000 -27.451000000

H -9.883000000 8.434000000 -26.512000000

H -11.594000000 8.639000000 -26.064000000

N -11.160000000 5.939000000 -22.257000000

H -11.852000000 6.686000000 -22.193000000

C -11.208000000 4.850000000 -21.299000000

C -12.247000000 3.846000000 -21.763000000

O -13.295000000 4.220000000 -22.292000000

C -11.569000000 5.384000000 -19.908000000

C -11.561000000 4.333000000 -18.800000000

C -11.519000000 4.986000000 -17.417000000

C -11.276000000 3.955000000 -16.326000000

N -10.825000000 4.567000000 -15.042000000

H -10.662000000 3.875000000 -14.310000000

H -10.000000000 5.149000000 -15.185000000

H -10.233000000 4.365000000 -21.256000000

H -10.850000000 6.160000000 -19.645000000

H -12.562000000 5.832000000 -19.955000000

H -12.463000000 3.726000000 -18.879000000

H -10.687000000 3.693000000 -18.920000000

H -10.715000000 5.721000000 -17.396000000

H -12.468000000 5.488000000 -17.230000000

H -10.509000000 3.260000000 -16.668000000

H -12.199000000 3.403000000 -16.149000000

O -12.039000000 2.635000000 -21.608000000

N -19.659000000 7.628000000 -11.972000000

C -18.418000000 8.207000000 -12.466000000

C -18.674000000 9.165000000 -13.615000000

O -17.964000000 10.150000000 -13.771000000

C -17.459000000 7.107000000 -12.934000000

C -16.274000000 7.626000000 -13.753000000

C -15.176000000 6.582000000 -13.982000000

O -14.245000000 6.852000000 -14.766000000

O -15.229000000 5.495000000 -13.377000000

H -17.944000000 8.758000000 -11.654000000

H -17.070000000 6.594000000 -12.055000000

H -18.016000000 6.391000000 -13.539000000

H -15.837000000 8.474000000 -13.226000000

H -16.640000000 7.967000000 -14.721000000

H -19.798000000 7.980000000 -11.036000000

H -20.413000000 7.975000000 -12.548000000

N -19.695000000 8.880000000 -14.415000000

H -20.267000000 8.057000000 -14.226000000

C -20.010000000 9.725000000 -15.559000000

C -20.763000000 10.996000000 -15.182000000

O -20.455000000 12.080000000 -15.689000000

C -20.822000000 8.943000000 -16.620000000

C -19.966000000 7.816000000 -17.197000000

C -21.253000000 9.870000000 -17.747000000

C -20.730000000 6.885000000 -18.097000000

H -19.068000000 10.023000000 -16.018000000

H -21.707000000 8.515000000 -16.150000000

H -19.555000000 7.237000000 -16.370000000

H -19.143000000 8.254000000 -17.762000000

H -21.864000000 10.676000000 -17.341000000

H -20.370000000 10.291000000 -18.229000000

H -21.833000000 9.307000000 -18.479000000

H -20.061000000 6.110000000 -18.471000000

H -21.543000000 6.424000000 -17.536000000

H -21.140000000 7.446000000 -18.936000000

N -21.749000000 10.869000000 -14.299000000

H -21.968000000 9.950000000 -13.914000000

C -22.518000000 12.031000000 -13.878000000

C -21.650000000 12.983000000 -13.077000000

O -21.880000000 14.191000000 -13.080000000

C -23.743000000 11.611000000 -13.061000000

S -25.103000000 11.007000000 -14.097000000

H -22.865000000 12.553000000 -14.770000000

H -24.094000000 12.472000000 -12.492000000

H -23.451000000 10.823000000 -12.366000000

H -25.072000000 9.672000000 -14.141000000

N -20.645000000 12.444000000 -12.393000000

H -20.503000000 11.434000000 -12.409000000

C -19.746000000 13.290000000 -11.621000000

C -18.885000000 14.091000000 -12.588000000

O -18.670000000 15.284000000 -12.396000000

C -18.839000000 12.460000000 -10.681000000

C -19.665000000 11.940000000 -9.504000000

C -17.676000000 13.309000000 -10.172000000

C -18.835000000 11.380000000 -8.374000000

H -20.339000000 13.980000000 -11.021000000

H -18.440000000 11.610000000 -11.235000000

H -20.264000000 12.764000000 -9.115000000

H -20.336000000 11.160000000 -9.865000000

H -17.348000000 13.986000000 -10.960000000

H -16.850000000 12.658000000 -9.885000000

H -18.000000000 13.888000000 -9.307000000

H -19.432000000 10.670000000 -7.801000000

H -18.513000000 12.193000000 -7.723000000

H -17.960000000 10.873000000 -8.782000000

N -18.408000000 13.436000000 -13.639000000

H -18.623000000 12.445000000 -13.752000000

C -17.583000000 14.107000000 -14.633000000

C -18.350000000 15.230000000 -15.328000000

O -17.787000000 16.280000000 -15.632000000

C -17.068000000 13.094000000 -15.661000000

C -15.763000000 12.428000000 -15.228000000

O -14.713000000 13.071000000 -15.171000000

N -15.826000000 11.140000000 -14.919000000

H -14.955000000 10.695000000 -14.630000000

H -16.695000000 10.608000000 -14.966000000

H -16.724000000 14.544000000 -14.125000000

H -17.825000000 12.322000000 -15.799000000

H -16.906000000 13.605000000 -16.610000000

O -19.552000000 15.081000000 -15.588000000

N -8.842000000 16.763000000 -14.873000000

C -8.904000000 16.251000000 -16.233000000

C -10.236000000 16.661000000 -16.839000000

O -11.274000000 16.583000000 -16.184000000

C -8.757000000 14.730000000 -16.248000000

C -9.094000000 14.181000000 -17.629000000

C -7.333000000 14.369000000 -15.868000000

H -8.096000000 16.693000000 -16.817000000

H -9.441000000 14.299000000 -15.517000000

H -8.581000000 13.231000000 -17.778000000

H -10.171000000 14.029000000 -17.706000000

H -8.772000000 14.891000000 -18.391000000

H -6.638000000 15.006000000 -16.415000000

H -7.144000000 13.325000000 -16.119000000

H -7.195000000 14.516000000 -14.797000000

O -10.277000000 17.078000000 -18.005000000

H -9.780000000 17.039000000 -14.622000000

H -8.271000000 17.596000000 -14.886000000

N -15.343000000 6.524000000 -22.559000000

H -14.625000000 5.821000000 -22.383000000

C -15.276000000 7.803000000 -21.865000000

C -14.182000000 8.625000000 -22.512000000

O -13.046000000 8.168000000 -22.634000000

C -14.959000000 7.609000000 -20.381000000

C -16.112000000 7.393000000 -19.396000000

C -16.908000000 6.156000000 -19.755000000

C -15.539000000 7.261000000 -17.996000000

H -16.229000000 8.322000000 -21.968000000

H -14.307000000 6.739000000 -20.305000000

H -14.393000000 8.478000000 -20.045000000

H -16.772000000 8.260000000 -19.428000000

H -16.350000000 7.107000000 -17.284000000

H -14.998000000 8.171000000 -17.738000000

H -14.858000000 6.411000000 -17.961000000

H -17.313000000 6.263000000 -20.761000000

H -17.726000000 6.032000000 -19.045000000

H -16.258000000 5.282000000 -19.717000000

H -16.239000000 6.107000000 -22.349000000

N -14.529000000 9.839000000 -22.923000000

H -15.487000000 10.158000000 -22.777000000

C -13.579000000 10.727000000 -23.576000000

C -13.120000000 11.815000000 -22.625000000

O -13.885000000 12.710000000 -22.267000000

C -14.217000000 11.331000000 -24.828000000

C -14.539000000 10.312000000 -25.883000000

C -15.367000000 9.237000000 -25.594000000

C -13.993000000 10.411000000 -27.153000000

C -15.645000000 8.276000000 -26.546000000

C -14.267000000 9.449000000 -28.119000000

C -15.095000000 8.379000000 -27.812000000

H -12.710000000 10.142000000 -23.878000000

H -15.141000000 11.832000000 -24.538000000

H -13.534000000 12.069000000 -25.249000000

H -15.801000000 9.150000000 -24.609000000

H -13.349000000 11.243000000 -27.395000000

H -16.291000000 7.445000000 -26.303000000

H -13.836000000 9.535000000 -29.106000000

H -15.310000000 7.629000000 -28.559000000

N -11.852000000 11.728000000 -22.235000000

H -11.275000000 10.976000000 -22.613000000

C -11.252000000 12.660000000 -21.292000000

C -10.018000000 13.331000000 -21.868000000

O -9.420000000 12.837000000 -22.819000000

C -10.880000000 11.902000000 -20.021000000

C -12.053000000 11.078000000 -19.490000000

C -11.581000000 9.694000000 -19.097000000

C -12.706000000 11.813000000 -18.329000000

H -11.985000000 13.427000000 -21.042000000

H -10.048000000 11.232000000 -20.240000000

H -10.571000000 12.616000000 -19.257000000

H -12.789000000 10.975000000 -20.288000000

H -12.424000000 9.115000000 -18.720000000

H -11.158000000 9.194000000 -19.968000000

H -10.821000000 9.776000000 -18.320000000

H -13.542000000 11.225000000 -17.951000000

H -11.975000000 11.958000000 -17.534000000

H -13.068000000 12.783000000 -18.671000000

N -9.636000000 14.455000000 -21.276000000

H -10.171000000 14.803000000 -20.480000000

C -8.474000000 15.205000000 -21.731000000

C -7.220000000 14.350000000 -21.754000000

O -7.030000000 13.486000000 -20.901000000

C -8.221000000 16.412000000 -20.820000000

C -7.118000000 17.331000000 -21.327000000

C -6.586000000 18.273000000 -20.262000000

O -7.384000000 19.025000000 -19.657000000

O -5.359000000 18.261000000 -20.035000000

H -8.667000000 15.566000000 -22.741000000

H -9.143000000 16.988000000 -20.745000000

H -7.950000000 16.052000000 -19.827000000

H -6.294000000 16.716000000 -21.688000000

H -7.506000000 17.922000000 -22.157000000

N -6.362000000 14.589000000 -22.735000000

H -6.577000000 15.295000000 -23.439000000

C -5.117000000 13.848000000 -22.810000000

C -4.047000000 14.649000000 -22.083000000

O -3.991000000 15.874000000 -22.210000000

C -4.678000000 13.637000000 -24.256000000

C -3.285000000 13.061000000 -24.334000000

C -2.980000000 11.863000000 -23.704000000

C -2.267000000 13.732000000 -24.994000000

C -1.696000000 11.347000000 -23.725000000

C -0.975000000 13.224000000 -25.022000000

C -0.697000000 12.030000000 -24.382000000

O 0.585000000 11.521000000 -24.383000000

H 1.274000000 11.992000000 -24.836000000

H -5.240000000 12.881000000 -22.323000000

H -5.372000000 12.950000000 -24.740000000

H -4.699000000 14.593000000 -24.779000000

H -3.760000000 11.323000000 -23.187000000

H -2.482000000 14.665000000 -25.494000000

H -1.479000000 10.413000000 -23.228000000

H -0.192000000 13.758000000 -25.540000000

N -3.202000000 13.962000000 -21.321000000

H -3.300000000 12.949000000 -21.255000000

C -2.138000000 14.627000000 -20.578000000

C -0.785000000 14.060000000 -21.008000000

O -0.324000000 13.047000000 -20.490000000

C -2.346000000 14.435000000 -19.063000000

S -3.922000000 15.086000000 -18.375000000

H -2.162000000 15.693000000 -20.805000000

H -1.526000000 14.935000000 -18.548000000

H -2.290000000 13.369000000 -18.842000000

H -4.393000000 16.048000000 -19.173000000

N -0.155000000 14.722000000 -21.968000000

H -0.586000000 15.560000000 -22.358000000

C 1.138000000 14.285000000 -22.481000000

C 2.219000000 14.358000000 -21.410000000

O 3.280000000 13.738000000 -21.538000000

C 1.550000000 15.164000000 -23.659000000

O 1.827000000 16.483000000 -23.218000000

H 2.083000000 17.030000000 -23.951000000

H 1.051000000 13.254000000 -22.824000000

H 2.443000000 14.746000000 -24.124000000

H 0.741000000 15.190000000 -24.390000000

N 1.940000000 15.127000000 -20.362000000

H 1.041000000 15.608000000 -20.319000000

C 2.889000000 15.295000000 -19.280000000

C 3.228000000 14.008000000 -18.568000000

O 4.370000000 13.798000000 -18.178000000

H 2.465000000 15.989000000 -18.554000000

H 3.806000000 15.726000000 -19.682000000

N 2.239000000 13.140000000 -18.398000000

H 1.306000000 13.360000000 -18.746000000

C 2.477000000 11.879000000 -17.720000000

C 1.959000000 11.933000000 -16.302000000

O 1.180000000 12.813000000 -15.966000000

H 1.966000000 11.081000000 -18.258000000

H 3.548000000 11.675000000 -17.705000000

N 2.386000000 11.001000000 -15.462000000

H 3.035000000 10.286000000 -15.791000000

C 1.941000000 10.985000000 -14.075000000

C 2.933000000 11.709000000 -13.173000000

O 4.133000000 11.724000000 -13.433000000

C 1.763000000 9.545000000 -13.592000000

C 0.936000000 8.685000000 -14.530000000

C 0.578000000 7.333000000 -13.943000000

O 1.397000000 6.783000000 -13.176000000

O -0.520000000 6.819000000 -14.264000000

H 0.979000000 11.494000000 -14.013000000

H 1.270000000 9.567000000 -12.620000000

H 2.747000000 9.090000000 -13.476000000

H 0.013000000 9.216000000 -14.764000000

H 1.496000000 8.531000000 -15.452000000

O 2.522000000 12.292000000 -12.160000000

N -2.154000000 13.882000000 -12.921000000

H -3.115000000 14.209000000 -12.820000000

C -1.496000000 13.980000000 -14.208000000

C -0.940000000 15.358000000 -14.520000000

O -1.238000000 16.336000000 -13.849000000

C -2.456000000 13.539000000 -15.313000000

C -3.008000000 12.130000000 -15.078000000

C -3.818000000 11.709000000 -16.275000000

C -1.871000000 11.156000000 -14.838000000

H -0.659000000 13.282000000 -14.203000000

H -3.290000000 14.240000000 -15.352000000

H -1.931000000 13.559000000 -16.268000000

H -3.654000000 12.144000000 -14.200000000

H -2.184000000 10.153000000 -15.127000000

H -1.604000000 11.163000000 -13.781000000

H -1.007000000 11.452000000 -15.433000000

H -4.213000000 10.706000000 -16.112000000

H -3.184000000 11.710000000 -17.161000000

H -4.644000000 12.406000000 -16.418000000

H -1.595000000 14.408000000 -12.265000000

O -0.166000000 15.495000000 -15.477000000

N -7.232000000 13.290000000 -11.662000000

C -7.919000000 12.392000000 -12.581000000

C -8.383000000 11.096000000 -11.941000000

O -7.817000000 10.651000000 -10.947000000

C -7.017000000 12.057000000 -13.774000000

O -6.332000000 10.827000000 -13.589000000

H -5.773000000 10.619000000 -14.328000000

H -8.799000000 12.911000000 -12.962000000

H -6.284000000 12.854000000 -13.896000000

H -7.627000000 11.993000000 -14.675000000

H -6.646000000 13.891000000 -12.224000000

H -7.932000000 13.871000000 -11.222000000

N -9.409000000 10.496000000 -12.542000000

H -9.805000000 10.944000000 -13.369000000

C -10.005000000 9.238000000 -12.095000000

C -11.043000000 9.433000000 -10.996000000

O -10.729000000 9.410000000 -9.804000000

C -8.935000000 8.242000000 -11.633000000

C -9.461000000 6.806000000 -11.586000000

O -8.656000000 5.868000000 -11.368000000

O -10.685000000 6.617000000 -11.771000000

H -10.515000000 8.796000000 -12.951000000

H -8.600000000 8.525000000 -10.635000000

H -8.088000000 8.288000000 -12.318000000

N -12.293000000 9.620000000 -11.407000000

H -12.499000000 9.616000000 -12.406000000

C -13.367000000 9.829000000 -10.459000000

C -14.265000000 8.614000000 -10.320000000

O -15.444000000 8.727000000 -9.974000000

C -14.166000000 11.065000000 -10.864000000

C -13.355000000 12.320000000 -10.833000000

C -12.522000000 12.648000000 -11.889000000

C -13.352000000 13.128000000 -9.704000000

C -11.703000000 13.748000000 -11.816000000

C -12.526000000 14.234000000 -9.632000000

C -11.704000000 14.540000000 -10.684000000

H -12.919000000 10.026000000 -9.485000000

H -15.005000000 11.177000000 -10.177000000

H -14.553000000 10.920000000 -11.873000000

H -12.516000000 12.034000000 -12.777000000

H -14.001000000 12.890000000 -8.874000000

H -11.057000000 13.994000000 -12.645000000

H -12.529000000 14.855000000 -8.749000000

H -11.056000000 15.402000000 -10.628000000

N -13.682000000 7.446000000 -10.584000000

H -12.704000000 7.428000000 -10.874000000

C -14.414000000 6.199000000 -10.466000000

C -14.750000000 5.891000000 -9.015000000

O -15.567000000 5.023000000 -8.743000000

H -13.804000000 5.390000000 -10.869000000

H -15.338000000 6.271000000 -11.040000000

O -14.194000000 6.522000000 -8.106000000

C -2.886638000 8.449432000 -17.939008000

C -4.024923000 9.081836000 -18.468423000

C -1.665207000 8.688290000 -18.554554000

C -3.911376000 9.988360000 -19.543348000

C -1.567265000 9.592697000 -19.645608000

C -2.670660000 10.250877000 -20.142227000

C -5.425816000 9.093110000 -18.168833000

N -6.101979000 9.911096000 -18.954673000

N -5.178678000 10.452287000 -19.783180000

H -5.462465000 11.187329000 -20.409258000

S -6.173095000 8.044264000 -16.929007000

O -6.318486000 6.692221000 -17.493038000

O -5.332388000 8.216371000 -15.729707000

C -7.826444000 8.672324000 -16.599210000

C -8.131575000 10.031233000 -16.521390000

C -9.409297000 10.417699000 -16.116420000

C -10.379593000 9.467868000 -15.766166000

C -11.781849000 9.865069000 -15.372577000

C -10.040857000 8.112789000 -15.849384000

C -8.780746000 7.705831000 -16.276590000

N -0.472415000 8.042501000 -18.144142000

N 0.725000000 8.619661000 -18.470761000

C 1.653850000 7.750389000 -18.092767000

C 3.107551000 8.064424000 -18.266963000

C 1.063275000 6.594255000 -17.524061000

C -0.298775000 6.801414000 -17.571071000

C -1.399113000 5.878182000 -17.165587000

H -2.986592000 7.826458000 -17.064150000

H -2.560852000 10.907340000 -20.991156000

H -0.590165000 9.752110000 -20.081589000

H 1.560865000 5.720372000 -17.128864000

H -8.522736000 6.656962000 -16.364883000

H -10.789003000 7.374421000 -15.579754000

H -9.657320000 11.474691000 -16.072937000

H -7.391125000 10.775894000 -16.787314000

H -0.962920000 4.907271000 -16.924736000

H -1.950151000 6.215181000 -16.283128000

H -2.134198000 5.755121000 -17.966855000

H 3.632109000 7.245883000 -18.770983000

H 3.228125000 8.972358000 -18.861164000

H 3.599752000 8.220076000 -17.300178000

H -11.822318000 10.868312000 -14.941275000

H -12.205564000 9.157475000 -14.656570000

H -12.450675000 9.850673000 -16.239032000

H -3.241000000 -0.381000000 -18.669000000

H -11.878000000 1.696000000 -21.488000000

H 2.026000000 6.360000000 -12.587000000

H 2.204000000 12.744000000 -11.375000000

H 0.434000000 15.601000000 -16.219000000

H -4.415000000 18.252000000 -19.860000000

H -20.483000000 14.966000000 -15.789000000

H -13.763000000 7.011000000 -7.401000000

H -8.040000000 5.151000000 -11.201000000

H -7.052000000 4.171000000 -24.863000000

H -10.309000000 17.401000000 -18.908000000

H -15.270000000 4.657000000 -12.911000000

Azole **5** with surrounded residues

N -2.564000000 3.856000000 -23.064000000

C -2.089000000 4.847000000 -22.104000000

C -1.721000000 4.207000000 -20.769000000

O -0.655000000 4.475000000 -20.213000000

C -3.161000000 5.910000000 -21.873000000

C -3.399000000 6.915000000 -22.996000000

C -4.670000000 7.698000000 -22.725000000

C -2.199000000 7.842000000 -23.096000000

H -1.202000000 5.331000000 -22.514000000

H -4.103000000 5.392000000 -21.692000000

H -2.900000000 6.464000000 -20.972000000

H -3.509000000 6.375000000 -23.937000000

H -4.583000000 8.694000000 -23.158000000

H -5.518000000 7.180000000 -23.173000000

H -4.823000000 7.782000000 -21.649000000

H -2.362000000 8.563000000 -23.897000000

H -2.069000000 8.371000000 -22.152000000

H -1.305000000 7.257000000 -23.311000000

H -2.020000000 3.018000000 -22.917000000

H -2.356000000 4.200000000 -23.991000000

N -2.611000000 3.366000000 -20.258000000

H -3.480000000 3.182000000 -20.760000000

C -2.358000000 2.706000000 -18.992000000

C -3.507000000 1.800000000 -18.602000000

O -4.587000000 2.270000000 -18.248000000

H -1.450000000 2.110000000 -19.078000000

H -2.218000000 3.460000000 -18.217000000

N -3.272000000 0.493000000 -18.666000000

H -2.351000000 0.172000000 -18.964000000

C -4.289000000 -0.498000000 -18.324000000

C -4.435000000 -0.697000000 -16.821000000

O -3.535000000 -0.369000000 -16.048000000

C -3.956000000 -1.837000000 -18.987000000

C -4.043000000 -1.816000000 -20.509000000

C -3.513000000 -3.092000000 -21.141000000

O -3.952000000 -4.185000000 -20.718000000

O -2.664000000 -3.002000000 -22.059000000

H -5.246000000 -0.153000000 -18.716000000

H -4.654000000 -2.586000000 -18.615000000

H -2.946000000 -2.128000000 -18.699000000

H -5.087000000 -1.691000000 -20.797000000

H -3.468000000 -0.970000000 -20.885000000

O -5.475000000 -1.196000000 -16.370000000

N -10.051000000 -1.941000000 -14.398000000

H -9.466000000 -1.212000000 -13.990000000

C -11.166000000 -1.542000000 -15.262000000

C -11.060000000 -2.110000000 -16.685000000

O -11.995000000 -2.725000000 -17.208000000

C -11.226000000 -0.013000000 -15.343000000

C -9.876000000 0.638000000 -15.590000000

C -8.922000000 0.709000000 -14.580000000

C -9.557000000 1.185000000 -16.830000000

C -7.693000000 1.311000000 -14.795000000

C -8.328000000 1.789000000 -17.057000000

C -7.398000000 1.853000000 -16.033000000

O -6.184000000 2.482000000 -16.228000000

H -5.546000000 2.526000000 -15.525000000

H -12.094000000 -1.900000000 -14.816000000

H -11.895000000 0.262000000 -16.158000000

H -11.635000000 0.372000000 -14.409000000

H -9.144000000 0.287000000 -13.611000000

H -10.280000000 1.138000000 -17.631000000

H -6.966000000 1.357000000 -13.997000000

H -8.099000000 2.206000000 -18.026000000

O -10.011000000 -1.947000000 -17.324000000

H -10.442000000 -2.472000000 -13.633000000

N -6.914000000 2.232000000 -21.285000000

H -5.958000000 1.916000000 -21.121000000

C -7.141000000 3.614000000 -21.675000000

C -6.212000000 3.896000000 -22.850000000

O -4.998000000 3.971000000 -22.680000000

C -6.812000000 4.580000000 -20.522000000

C -7.120000000 6.006000000 -20.938000000

C -7.614000000 4.206000000 -19.290000000

H -8.178000000 3.746000000 -21.983000000

H -5.750000000 4.502000000 -20.289000000

H -8.115000000 6.280000000 -20.588000000

H -6.383000000 6.679000000 -20.500000000

H -7.083000000 6.084000000 -22.025000000

H -8.092000000 5.097000000 -18.882000000

H -6.949000000 3.776000000 -18.541000000

H -8.377000000 3.476000000 -19.561000000

H -7.296000000 1.651000000 -22.017000000

O -6.685000000 4.051000000 -23.984000000

N -7.810000000 11.109000000 -24.650000000

C -7.667000000 9.874000000 -23.900000000

C -9.024000000 9.198000000 -23.777000000

O -9.997000000 9.801000000 -23.325000000

C -7.092000000 10.166000000 -22.525000000

H -6.987000000 9.211000000 -24.435000000

H -7.645000000 10.985000000 -22.066000000

H -7.175000000 9.277000000 -21.900000000

H -6.043000000 10.445000000 -22.622000000

H -8.157000000 10.859000000 -25.565000000

H -6.888000000 11.505000000 -24.769000000

N -9.086000000 7.939000000 -24.187000000

H -8.246000000 7.484000000 -24.546000000

C -10.334000000 7.200000000 -24.132000000

C -10.241000000 5.992000000 -23.215000000

O -9.362000000 5.136000000 -23.368000000

C -10.757000000 6.739000000 -25.535000000

C -12.086000000 6.006000000 -25.467000000

C -10.854000000 7.941000000 -26.455000000

H -11.107000000 7.864000000 -23.744000000

H -10.000000000 6.058000000 -25.924000000

H -12.376000000 5.684000000 -26.467000000

H -12.849000000 6.673000000 -25.065000000

H -11.988000000 5.135000000 -24.819000000

H -11.154000000 7.614000000 -27.451000000

H -9.883000000 8.434000000 -26.512000000

H -11.594000000 8.639000000 -26.064000000

N -11.160000000 5.939000000 -22.257000000

H -11.852000000 6.686000000 -22.193000000

C -11.208000000 4.850000000 -21.299000000

C -12.247000000 3.846000000 -21.763000000

O -13.295000000 4.220000000 -22.292000000

C -11.569000000 5.384000000 -19.908000000

C -11.561000000 4.333000000 -18.800000000

C -11.519000000 4.986000000 -17.417000000

C -11.276000000 3.955000000 -16.326000000

N -10.825000000 4.567000000 -15.042000000

H -10.662000000 3.875000000 -14.310000000

H -10.000000000 5.149000000 -15.185000000

H -10.233000000 4.365000000 -21.256000000

H -10.850000000 6.160000000 -19.645000000

H -12.562000000 5.832000000 -19.955000000

H -12.463000000 3.726000000 -18.879000000

H -10.687000000 3.693000000 -18.920000000

H -10.715000000 5.721000000 -17.396000000

H -12.468000000 5.488000000 -17.230000000

H -10.509000000 3.260000000 -16.668000000

H -12.199000000 3.403000000 -16.149000000

O -12.039000000 2.635000000 -21.608000000

N -8.842000000 16.763000000 -14.873000000

H -8.499000000 16.166000000 -14.120000000

C -8.904000000 16.251000000 -16.233000000

C -10.236000000 16.661000000 -16.839000000

O -11.274000000 16.583000000 -16.184000000

C -8.757000000 14.730000000 -16.248000000

C -9.094000000 14.181000000 -17.629000000

C -7.333000000 14.369000000 -15.868000000

H -8.096000000 16.693000000 -16.817000000

H -9.441000000 14.299000000 -15.517000000

H -8.581000000 13.231000000 -17.778000000

H -10.171000000 14.029000000 -17.706000000

H -8.772000000 14.891000000 -18.391000000

H -6.638000000 15.006000000 -16.415000000

H -7.144000000 13.325000000 -16.119000000

H -7.195000000 14.516000000 -14.797000000

H -8.271000000 17.596000000 -14.886000000

O -10.277000000 17.078000000 -18.005000000

N -11.852000000 11.728000000 -22.235000000

H -11.275000000 10.976000000 -22.613000000

C -11.252000000 12.660000000 -21.292000000

C -10.018000000 13.331000000 -21.868000000

O -9.420000000 12.837000000 -22.819000000

C -10.880000000 11.902000000 -20.021000000

C -12.053000000 11.078000000 -19.490000000

C -11.581000000 9.694000000 -19.097000000

C -12.706000000 11.813000000 -18.329000000

H -11.985000000 13.427000000 -21.042000000

H -10.048000000 11.232000000 -20.240000000

H -10.571000000 12.616000000 -19.257000000

H -12.789000000 10.975000000 -20.288000000

H -12.424000000 9.115000000 -18.720000000

H -11.158000000 9.194000000 -19.968000000

H -10.821000000 9.776000000 -18.320000000

H -13.542000000 11.225000000 -17.951000000

H -11.975000000 11.958000000 -17.534000000

H -13.068000000 12.783000000 -18.671000000

H -12.184000000 12.271000000 -23.019000000

N -9.636000000 14.455000000 -21.276000000

H -10.171000000 14.803000000 -20.480000000

C -8.474000000 15.205000000 -21.731000000

C -7.220000000 14.350000000 -21.754000000

O -7.030000000 13.486000000 -20.901000000

C -8.221000000 16.412000000 -20.820000000

C -7.118000000 17.331000000 -21.327000000

C -6.586000000 18.273000000 -20.262000000

O -7.384000000 19.025000000 -19.657000000

O -5.359000000 18.261000000 -20.035000000

H -8.667000000 15.566000000 -22.741000000

H -9.143000000 16.988000000 -20.745000000

H -7.950000000 16.052000000 -19.827000000

H -6.294000000 16.716000000 -21.688000000

H -7.506000000 17.922000000 -22.157000000

N -6.362000000 14.589000000 -22.735000000

H -6.577000000 15.295000000 -23.439000000

C -5.117000000 13.848000000 -22.810000000

C -4.047000000 14.649000000 -22.083000000

O -3.991000000 15.874000000 -22.210000000

C -4.678000000 13.637000000 -24.256000000

C -3.285000000 13.061000000 -24.334000000

C -2.980000000 11.863000000 -23.704000000

C -2.267000000 13.732000000 -24.994000000

C -1.696000000 11.347000000 -23.725000000

C -0.975000000 13.224000000 -25.022000000

C -0.697000000 12.030000000 -24.382000000

O 0.585000000 11.521000000 -24.383000000

H 1.274000000 11.992000000 -24.836000000

H -5.240000000 12.881000000 -22.323000000

H -5.372000000 12.950000000 -24.740000000

H -4.699000000 14.593000000 -24.779000000

H -3.760000000 11.323000000 -23.187000000

H -2.482000000 14.665000000 -25.494000000

H -1.479000000 10.413000000 -23.228000000

H -0.192000000 13.758000000 -25.540000000

N -3.202000000 13.962000000 -21.321000000

H -3.300000000 12.949000000 -21.255000000

C -2.138000000 14.627000000 -20.578000000

C -0.785000000 14.060000000 -21.008000000

O -0.324000000 13.047000000 -20.490000000

C -2.346000000 14.435000000 -19.063000000

S -3.922000000 15.086000000 -18.375000000

H -2.162000000 15.693000000 -20.805000000

H -1.526000000 14.935000000 -18.548000000

H -2.290000000 13.369000000 -18.842000000

H -4.393000000 16.048000000 -19.173000000

N -0.155000000 14.722000000 -21.968000000

H -0.586000000 15.560000000 -22.358000000

C 1.138000000 14.285000000 -22.481000000

C 2.219000000 14.358000000 -21.410000000

O 3.280000000 13.738000000 -21.538000000

C 1.550000000 15.164000000 -23.659000000

O 1.827000000 16.483000000 -23.218000000

H 2.083000000 17.030000000 -23.951000000

H 1.051000000 13.254000000 -22.824000000

H 2.443000000 14.746000000 -24.124000000

H 0.741000000 15.190000000 -24.390000000

N 1.940000000 15.127000000 -20.362000000

H 1.041000000 15.608000000 -20.319000000

C 2.889000000 15.295000000 -19.280000000

C 3.228000000 14.008000000 -18.568000000

O 4.370000000 13.798000000 -18.178000000

H 2.465000000 15.989000000 -18.554000000

H 3.806000000 15.726000000 -19.682000000

N 2.239000000 13.140000000 -18.398000000

H 1.306000000 13.360000000 -18.746000000

C 2.477000000 11.879000000 -17.720000000

C 1.959000000 11.933000000 -16.302000000

O 1.180000000 12.813000000 -15.966000000

H 1.966000000 11.081000000 -18.258000000

H 3.548000000 11.675000000 -17.705000000

N 2.386000000 11.001000000 -15.462000000

H 3.035000000 10.286000000 -15.791000000

C 1.941000000 10.985000000 -14.075000000

C 2.933000000 11.709000000 -13.173000000

O 4.133000000 11.724000000 -13.433000000

C 1.763000000 9.545000000 -13.592000000

C 0.936000000 8.685000000 -14.530000000

C 0.578000000 7.333000000 -13.943000000

O 1.397000000 6.783000000 -13.176000000

O -0.520000000 6.819000000 -14.264000000

H 0.979000000 11.494000000 -14.013000000

H 1.270000000 9.567000000 -12.620000000

H 2.747000000 9.090000000 -13.476000000

H 0.013000000 9.216000000 -14.764000000

H 1.496000000 8.531000000 -15.452000000

O 2.522000000 12.292000000 -12.160000000

N -2.651000000 9.314000000 -9.781000000

C -3.941000000 9.877000000 -10.174000000

C -3.811000000 11.368000000 -10.436000000

O -4.801000000 12.050000000 -10.695000000

C -4.998000000 9.655000000 -9.094000000

C -5.537000000 8.241000000 -9.091000000

O -5.763000000 7.652000000 -10.154000000

N -5.765000000 7.690000000 -7.897000000

H -5.579000000 8.176000000 -7.020000000

H -6.128000000 6.737000000 -7.895000000

H -4.271000000 9.389000000 -11.091000000

H -4.552000000 9.861000000 -8.121000000

H -5.823000000 10.348000000 -9.259000000

H -2.692000000 8.323000000 -9.973000000

H -1.946000000 9.714000000 -10.383000000

N -2.589000000 11.876000000 -10.338000000

H -1.813000000 11.266000000 -10.082000000

C -2.334000000 13.286000000 -10.589000000

C -1.522000000 13.372000000 -11.873000000

O -0.364000000 12.969000000 -11.928000000

C -1.585000000 13.910000000 -9.410000000

C -2.408000000 13.837000000 -8.116000000

C -1.596000000 14.305000000 -6.923000000

C -3.670000000 14.674000000 -8.285000000

H -3.283000000 13.804000000 -10.728000000

H -0.647000000 13.374000000 -9.263000000

H -1.368000000 14.954000000 -9.637000000

H -2.700000000 12.800000000 -7.950000000

H -0.702000000 13.689000000 -6.828000000

H -1.307000000 15.346000000 -7.067000000

H -2.196000000 14.217000000 -6.018000000

H -4.262000000 14.629000000 -7.371000000

H -4.256000000 14.283000000 -9.117000000

H -3.395000000 15.709000000 -8.489000000

N -2.154000000 13.882000000 -12.921000000

H -3.115000000 14.209000000 -12.820000000

C -1.496000000 13.980000000 -14.208000000

C -0.940000000 15.358000000 -14.520000000

O -1.238000000 16.336000000 -13.849000000

C -2.456000000 13.539000000 -15.313000000

C -3.008000000 12.130000000 -15.078000000

C -3.818000000 11.709000000 -16.275000000

C -1.871000000 11.156000000 -14.838000000

H -0.659000000 13.282000000 -14.203000000

H -3.290000000 14.240000000 -15.352000000

H -1.931000000 13.559000000 -16.268000000

H -3.654000000 12.144000000 -14.200000000

H -4.213000000 10.706000000 -16.112000000

H -3.184000000 11.710000000 -17.161000000

H -4.644000000 12.406000000 -16.418000000

H -2.184000000 10.153000000 -15.127000000

H -1.604000000 11.163000000 -13.781000000

H -1.007000000 11.452000000 -15.433000000

O -0.166000000 15.495000000 -15.477000000

N -7.232000000 13.290000000 -11.662000000

H -6.640000000 12.908000000 -10.924000000

C -7.919000000 12.392000000 -12.581000000

C -8.383000000 11.096000000 -11.941000000

O -7.817000000 10.651000000 -10.947000000

C -7.017000000 12.057000000 -13.774000000

O -6.332000000 10.827000000 -13.589000000

H -5.773000000 10.619000000 -14.328000000

H -8.799000000 12.911000000 -12.962000000

H -6.284000000 12.854000000 -13.896000000

H -7.627000000 11.993000000 -14.675000000

H -7.932000000 13.871000000 -11.222000000

N -9.409000000 10.496000000 -12.542000000

H -9.805000000 10.944000000 -13.369000000

C -10.005000000 9.238000000 -12.095000000

C -11.043000000 9.433000000 -10.996000000

O -10.729000000 9.410000000 -9.804000000

C -8.935000000 8.242000000 -11.633000000

C -9.461000000 6.806000000 -11.586000000

O -8.656000000 5.868000000 -11.368000000

O -10.685000000 6.617000000 -11.771000000

H -10.515000000 8.796000000 -12.951000000

H -8.600000000 8.525000000 -10.635000000

H -8.088000000 8.288000000 -12.318000000

O -12.228000000 9.619000000 -11.307000000

C -3.684474000 4.846635000 -14.267741000

C -2.822624000 5.284066000 -15.330525000

C -1.852046000 4.255421000 -15.409774000

N -2.214352000 3.321714000 -14.474316000

N -3.310450000 3.678199000 -13.776809000

C -0.750784000 4.342745000 -16.273924000

C -0.681828000 5.447498000 -17.103003000

C -1.663279000 6.476035000 -17.066791000

C -2.725799000 6.406598000 -16.171960000

H -1.760335000 2.450601000 -14.248379000

S -4.939648000 5.763356000 -13.365106000

O -5.753585000 4.801003000 -12.615933000

O -4.242623000 6.871344000 -12.693106000

C -5.953613000 6.482904000 -14.655125000

C -6.944245000 5.712190000 -15.261177000

C -7.703493000 6.279567000 -16.278160000

C -7.508636000 7.605145000 -16.688359000

C -8.326517000 8.197462000 -17.809092000

C -6.524895000 8.363984000 -16.041176000

C -5.743577000 7.809957000 -15.028138000

N -1.503297000 7.594844000 -17.920955000

C -0.267989000 8.147931000 -18.260941000

C -0.437757000 9.221734000 -19.083597000

C -1.848570000 9.382215000 -19.281737000

C -2.613718000 10.298111000 -20.017789000

C -2.498102000 8.352558000 -18.547173000

C -3.996273000 10.188248000 -20.006128000

C -4.623784000 9.151916000 -19.289863000

C -3.891517000 8.213485000 -18.569259000

H 0.004177000 3.564980000 -16.319122000

H 0.134901000 5.528019000 -17.810571000

H -3.427351000 7.224124000 -16.092419000

H 0.637247000 7.737943000 -17.840251000

H 0.345524000 9.837347000 -19.499819000

H -2.115347000 11.068401000 -20.594773000

H -4.602853000 10.896418000 -20.561501000

H -5.705843000 9.068758000 -19.316496000

H -4.396071000 7.405987000 -18.054085000

H -7.127269000 4.697811000 -14.925249000

H -8.467802000 5.681547000 -16.755814000

H -6.367916000 9.395167000 -16.341791000

H -4.982043000 8.387193000 -14.515445000

H -8.452786000 9.276006000 -17.680790000

H -7.838616000 8.041570000 -18.778217000

H -9.316733000 7.738924000 -17.869853000

H 2.204000000 12.744000000 -11.375000000

H -11.878000000 1.696000000 -21.488000000

H -2.014000000 -2.933000000 -22.762000000

H -12.722000000 -3.203000000 -17.614000000

H -13.146000000 9.763000000 -11.548000000

H -4.415000000 18.252000000 -19.860000000

H -10.309000000 17.401000000 -18.908000000

H -11.623000000 6.472000000 -11.913000000

H -6.281000000 -1.583000000 -16.020000000

H 2.026000000 6.360000000 -12.587000000

H 0.434000000 15.601000000 -16.219000000

H -7.052000000 4.171000000 -24.863000000

Azole **6** with surrounded residues

N -2.564000000 3.856000000 -23.064000000

H -1.790000000 3.209000000 -23.339000000

C -3.507000000 1.800000000 -18.602000000

O -4.587000000 2.270000000 -18.248000000

O -3.357000000 0.571000000 -18.640000000

N -10.051000000 -1.941000000 -14.398000000

H -10.037000000 -2.963000000 -14.235000000

H -10.104000000 -1.459000000 -13.475000000

C -7.299000000 -1.436000000 -24.371000000

O -6.417000000 -2.322000000 -24.395000000

O -7.680000000 -0.824000000 -25.394000000

C -6.212000000 3.896000000 -22.850000000

O -4.998000000 3.971000000 -22.680000000

O -6.685000000 4.051000000 -23.984000000

N -11.160000000 5.939000000 -22.257000000

C -12.247000000 3.846000000 -21.763000000

O -13.295000000 4.220000000 -22.292000000

N -10.825000000 4.567000000 -15.042000000

H -10.627000000 3.857000000 -14.316000000

H -9.964000000 5.133000000 -15.152000000

O -12.039000000 2.635000000 -21.608000000

H -10.890000000 6.840000000 -21.846000000

H -10.509000000 5.721000000 -23.032000000

N -19.659000000 7.628000000 -11.972000000

C -18.674000000 9.165000000 -13.615000000

O -17.964000000 10.150000000 -13.771000000

C -15.176000000 6.582000000 -13.982000000

O -14.245000000 6.852000000 -14.766000000

O -15.229000000 5.495000000 -13.377000000

O -19.607000000 8.949000000 -14.400000000

H -20.107000000 8.222000000 -11.265000000

H -19.498000000 6.692000000 -11.560000000

C -18.350000000 15.230000000 -15.328000000

O -17.787000000 16.280000000 -15.632000000

O -19.552000000 15.081000000 -15.588000000

N -8.842000000 16.763000000 -14.873000000

C -10.236000000 16.661000000 -16.839000000

O -11.274000000 16.583000000 -16.184000000

H -8.501000000 17.734000000 -14.843000000

H -8.265000000 16.189000000 -14.248000000

O -10.277000000 17.078000000 -18.005000000

N -11.852000000 11.728000000 -22.235000000

C -10.018000000 13.331000000 -21.868000000

O -9.420000000 12.837000000 -22.819000000

O -9.610000000 14.390000000 -21.372000000

H -11.899000000 10.760000000 -21.881000000

H -12.794000000 12.014000000 -22.531000000

N 2.386000000 11.001000000 -15.462000000

C 2.933000000 11.709000000 -13.173000000

O 4.133000000 11.724000000 -13.433000000

C 0.578000000 7.333000000 -13.943000000

O 1.397000000 6.783000000 -13.176000000

O -0.520000000 6.819000000 -14.264000000

O 2.522000000 12.292000000 -12.160000000

H 2.155000000 10.108000000 -15.947000000

H 1.990000000 11.781000000 -15.993000000

N -0.126000000 8.721000000 -8.903000000

C -0.073000000 6.184000000 -7.674000000

O -0.179000000 7.228000000 -6.994000000

O 0.805000000 5.319000000 -7.452000000

H -0.246000000 9.743000000 -8.897000000

H 0.883000000 8.531000000 -8.746000000

C -3.811000000 11.368000000 -10.436000000

O -4.801000000 12.050000000 -10.695000000

N -7.232000000 13.290000000 -11.662000000

C -9.461000000 6.806000000 -11.586000000

O -8.656000000 5.868000000 -11.368000000

O -10.685000000 6.617000000 -11.771000000

C -14.265000000 8.614000000 -10.320000000

O -15.444000000 8.727000000 -9.974000000

H -4.597000000 4.583000000 -23.396000000

H -6.916000000 14.150000000 -12.098000000

H -7.806000000 13.498000000 -10.831000000

O -13.806000000 7.488000000 -10.558000000

H -2.907000000 4.287000000 -23.941000000

O -2.695000000 11.903000000 -10.388000000

C -2.100000000 4.804000000 -21.944000000

C -1.815000000 3.756000000 -20.814000000

O -1.128000000 2.821000000 -21.212000000

C -3.117000000 5.867000000 -21.597000000

C -3.374000000 6.903000000 -22.708000000

C -4.427000000 7.897000000 -22.208000000

C -2.091000000 7.654000000 -23.075000000

H -1.127000000 5.265000000 -22.251000000

H -4.083000000 5.425000000 -21.260000000

H -2.755000000 6.422000000 -20.690000000

H -3.768000000 6.398000000 -23.621000000

H -4.674000000 8.645000000 -22.970000000

H -4.083000000 8.452000000 -21.322000000

H -5.365000000 7.407000000 -21.921000000

H -2.301000000 8.468000000 -23.782000000

H -1.635000000 8.124000000 -22.193000000

H -1.340000000 7.015000000 -23.541000000

N -2.417000000 3.821000000 -19.618000000

C -2.254000000 2.699000000 -18.691000000

H -2.054000000 3.094000000 -17.660000000

H -1.386000000 2.056000000 -18.972000000

H -2.885000000 4.637000000 -19.225000000

C -11.163000000 -1.464000000 -15.325000000

C -10.994000000 -2.117000000 -16.721000000

O -11.934000000 -2.699000000 -17.214000000

C -11.130000000 0.077000000 -15.431000000

C -9.758000000 0.610000000 -15.674000000

C -8.885000000 0.832000000 -14.592000000

C -9.310000000 0.896000000 -16.972000000

C -7.569000000 1.155000000 -14.805000000

C -7.990000000 1.223000000 -17.217000000

C -7.071000000 1.279000000 -16.140000000

O -5.797000000 1.398000000 -16.256000000

H -5.322000000 1.668000000 -17.259000000

H -12.148000000 -1.800000000 -14.906000000

H -11.826000000 0.379000000 -16.253000000

H -11.580000000 0.528000000 -14.519000000

H -9.264000000 0.776000000 -13.572000000

H -10.008000000 0.838000000 -17.813000000

H -6.872000000 1.338000000 -13.986000000

H -7.632000000 1.405000000 -18.228000000

N -9.752000000 -2.046000000 -17.275000000

H -9.015000000 -1.439000000 -16.901000000

C -9.528000000 -2.479000000 -18.663000000

C -8.216000000 -1.783000000 -19.089000000

O -7.214000000 -1.966000000 -18.425000000

H -9.390000000 -3.584000000 -18.706000000

H -10.401000000 -2.240000000 -19.310000000

N -8.306000000 -0.928000000 -20.136000000

H -9.161000000 -0.793000000 -20.679000000

C -7.120000000 -0.249000000 -20.682000000

C -7.573000000 1.185000000 -20.980000000

O -8.739000000 1.463000000 -21.160000000

C -6.714000000 -0.897000000 -22.023000000

C -7.879000000 -1.020000000 -22.999000000

H -6.271000000 -0.290000000 -19.946000000

H -6.278000000 -1.905000000 -21.846000000

H -5.891000000 -0.318000000 -22.486000000

H -8.602000000 -1.790000000 -22.699000000

H -8.430000000 -0.074000000 -23.131000000

N -6.587000000 2.161000000 -21.170000000

C -7.062000000 3.456000000 -21.644000000

C -7.027000000 4.553000000 -20.562000000

C -7.546000000 5.883000000 -21.111000000

C -7.863000000 4.115000000 -19.359000000

H -8.122000000 3.338000000 -22.043000000

H -5.964000000 4.680000000 -20.245000000

H -8.173000000 4.982000000 -18.748000000

H -7.291000000 3.452000000 -18.695000000

H -8.771000000 3.578000000 -19.656000000

H -8.583000000 5.809000000 -21.437000000

H -7.522000000 6.650000000 -20.308000000

H -6.949000000 6.259000000 -21.944000000

H -5.719000000 2.140000000 -20.650000000

C -11.182000000 4.847000000 -21.216000000

C -11.581000000 5.436000000 -19.875000000

C -11.562000000 4.349000000 -18.789000000

C -11.262000000 4.999000000 -17.438000000

C -11.272000000 3.941000000 -16.338000000

H -10.192000000 4.329000000 -21.169000000

H -12.600000000 5.878000000 -19.920000000

H -10.886000000 6.256000000 -19.589000000

H -12.534000000 3.813000000 -18.782000000

H -10.812000000 3.568000000 -19.031000000

H -10.273000000 5.515000000 -17.495000000

H -12.017000000 5.791000000 -17.221000000

H -12.300000000 3.526000000 -16.201000000

H -10.593000000 3.092000000 -16.589000000

C -18.322000000 8.201000000 -12.468000000

C -17.429000000 7.076000000 -12.981000000

C -16.224000000 7.672000000 -13.713000000

H -17.803000000 8.739000000 -11.625000000

H -17.018000000 6.459000000 -12.140000000

H -17.963000000 6.389000000 -13.659000000

H -15.724000000 8.439000000 -13.080000000

H -16.511000000 8.151000000 -14.659000000

C -17.519000000 14.065000000 -14.703000000

C -17.243000000 13.088000000 -15.836000000

C -16.023000000 12.209000000 -15.618000000

O -14.894000000 12.667000000 -15.586000000

N -16.235000000 10.856000000 -15.584000000

H -15.471000000 10.234000000 -15.368000000

H -17.153000000 10.470000000 -15.367000000

H -16.578000000 14.452000000 -14.243000000

H -17.040000000 13.689000000 -16.765000000

H -18.148000000 12.502000000 -16.097000000

C -8.843000000 16.216000000 -16.282000000

C -8.688000000 14.694000000 -16.294000000

C -9.033000000 14.185000000 -17.696000000

C -7.253000000 14.295000000 -15.939000000

H -8.047000000 16.706000000 -16.886000000

H -9.408000000 14.233000000 -15.571000000

H -8.850000000 13.111000000 -17.796000000

H -8.456000000 14.691000000 -18.485000000

H -10.088000000 14.391000000 -17.951000000

H -6.522000000 14.803000000 -16.580000000

H -6.982000000 14.487000000 -14.901000000

H -7.091000000 13.219000000 -16.125000000

C -11.284000000 12.691000000 -21.209000000

C -10.960000000 11.952000000 -19.930000000

C -12.239000000 11.554000000 -19.156000000

C -12.152000000 10.094000000 -18.699000000

C -12.443000000 12.487000000 -17.962000000

H -12.016000000 13.517000000 -21.022000000

H -10.322000000 12.623000000 -19.299000000

H -10.294000000 11.087000000 -20.106000000

H -13.131000000 11.654000000 -19.819000000

H -13.353000000 12.243000000 -17.391000000

H -12.544000000 13.537000000 -18.271000000

H -11.608000000 12.441000000 -17.254000000

H -13.003000000 9.834000000 -18.050000000

H -12.144000000 9.393000000 -19.531000000

H -11.250000000 9.915000000 -18.097000000

C 1.881000000 10.961000000 -14.030000000

C 1.760000000 9.513000000 -13.587000000

C 0.859000000 8.736000000 -14.531000000

H 0.901000000 11.491000000 -13.962000000

H 2.748000000 9.014000000 -13.482000000

H 1.353000000 9.481000000 -12.550000000

H -0.121000000 9.227000000 -14.665000000

H 1.296000000 8.567000000 -15.525000000

C -0.558000000 8.081000000 -10.197000000

C -1.993000000 8.413000000 -10.619000000

O -2.454000000 7.922000000 -11.624000000

C -0.404000000 6.557000000 -10.113000000

C -1.025000000 5.965000000 -8.855000000

H 0.108000000 8.455000000 -11.035000000

H -0.858000000 6.108000000 -11.036000000

H 0.665000000 6.262000000 -10.200000000

H -2.024000000 6.383000000 -8.646000000

H -1.173000000 4.874000000 -8.982000000

N -2.755000000 9.261000000 -9.827000000

H -2.325000000 9.855000000 -9.136000000

C -3.950000000 9.833000000 -10.459000000

C -5.227000000 9.304000000 -9.803000000

C -5.800000000 8.176000000 -10.638000000

O -5.653000000 8.148000000 -11.854000000

N -6.528000000 7.193000000 -10.030000000

H -6.782000000 7.239000000 -9.069000000

H -7.101000000 6.542000000 -10.610000000

H -3.968000000 9.564000000 -11.563000000

H -5.043000000 9.002000000 -8.758000000

H -5.992000000 10.121000000 -9.764000000

C -7.809000000 12.367000000 -12.697000000

C -8.400000000 11.128000000 -11.967000000

O -8.027000000 10.904000000 -10.840000000

C -6.609000000 11.826000000 -13.557000000

O -5.469000000 12.569000000 -13.247000000

H -4.987000000 12.193000000 -12.423000000

H -8.568000000 12.891000000 -13.308000000

H -6.437000000 10.740000000 -13.378000000

H -6.784000000 11.984000000 -14.634000000

N -9.156000000 10.349000000 -12.793000000

H -9.526000000 10.720000000 -13.657000000

C -9.900000000 9.213000000 -12.215000000

C -10.944000000 9.856000000 -11.271000000

O -10.704000000 10.944000000 -10.763000000

C -8.944000000 8.247000000 -11.539000000

H -10.429000000 8.679000000 -13.056000000

H -7.956000000 8.276000000 -12.048000000

H -8.769000000 8.530000000 -10.485000000

N -12.152000000 9.249000000 -11.254000000

C -13.285000000 9.821000000 -10.513000000

C -14.010000000 10.929000000 -11.305000000

C -13.190000000 12.147000000 -11.599000000

C -13.206000000 12.707000000 -12.878000000

C -12.416000000 12.763000000 -10.606000000

C -12.445000000 13.836000000 -13.176000000

C -11.654000000 13.889000000 -10.902000000

C -11.660000000 14.428000000 -12.190000000

H -12.962000000 10.198000000 -9.518000000

H -14.912000000 11.213000000 -10.717000000

H -14.418000000 10.485000000 -12.239000000

H -13.822000000 12.266000000 -13.665000000

H -12.376000000 12.330000000 -9.606000000

H -12.483000000 14.261000000 -14.180000000

H -11.050000000 14.345000000 -10.125000000

H -11.082000000 15.314000000 -12.412000000

H -12.221000000 8.479000000 -11.479000000

O -6.898946000 9.124373000 -19.362097000

S -6.637549000 8.647180000 -17.997053000

O -6.241044000 7.243451000 -17.769312000

C -8.087540000 8.993712000 -16.997092000

C -8.852428000 7.930554000 -16.523817000

C -10.071563000 8.203604000 -15.897606000

C -10.519288000 9.515980000 -15.711049000

C -11.864537000 9.805961000 -15.079388000

C -9.691286000 10.565349000 -16.151144000

C -8.498594000 10.315259000 -16.812554000

C -5.307292000 9.640868000 -17.325546000

N -5.485826000 10.941992000 -17.097113000

N -4.283255000 11.444951000 -16.754123000

H -4.217053000 12.423873000 -16.526692000

C -3.309352000 10.486028000 -16.777070000

C -3.940379000 9.291884000 -17.142821000

C -1.939228000 10.175450000 -16.631272000

N -2.963091000 8.341414000 -17.202772000

N -1.758656000 8.879560000 -16.885944000

C -3.063261000 6.912109000 -17.447638000

H -8.497140000 6.914933000 -16.656580000

H -10.690938000 7.385379000 -15.545728000

H -9.979901000 11.593819000 -15.960462000

H -7.872474000 11.122377000 -17.164514000

H -1.098254000 10.802545000 -16.374505000

H -3.155721000 6.361792000 -16.505928000

H -3.943435000 6.717131000 -18.058950000

H -2.153401000 6.598610000 -17.959733000

H -12.555045000 10.261928000 -15.794217000

H -12.334486000 8.894799000 -14.701970000

H -11.784909000 10.511087000 -14.246302000

N -18.249000000 13.397000000 -13.616000000

H -18.357000000 13.948000000 -12.775000000

H -17.934000000 12.459000000 -13.406000000

H 2.204000000 12.744000000 -11.375000000

H -10.309000000 17.401000000 -18.908000000

H -11.878000000 1.696000000 -21.488000000

H -7.972000000 -0.355000000 -26.179000000

H -20.483000000 14.966000000 -15.789000000

H -15.270000000 4.657000000 -12.911000000

H -13.450000000 6.616000000 -10.742000000

H -3.241000000 -0.381000000 -18.669000000

H -9.294000000 15.211000000 -20.988000000

H 2.026000000 6.360000000 -12.587000000

H 1.478000000 4.656000000 -7.282000000

H -8.040000000 5.151000000 -11.201000000

H -1.830000000 12.318000000 -10.351000000

H -20.330000000 8.782000000 -15.009000000

Azole **7** with surrounded residues

N -2.564000000 3.856000000 -23.064000000

C -2.089000000 4.847000000 -22.104000000

C -1.721000000 4.207000000 -20.769000000

O -0.655000000 4.475000000 -20.213000000

C -3.161000000 5.910000000 -21.873000000

C -3.399000000 6.915000000 -22.996000000

C -4.670000000 7.698000000 -22.725000000

C -2.199000000 7.842000000 -23.096000000

H -2.020000000 3.018000000 -22.917000000

H -2.356000000 4.200000000 -23.991000000

H -1.202000000 5.331000000 -22.514000000

H -2.878000000 6.473000000 -20.984000000

H -4.103000000 5.402000000 -21.667000000

H -3.509000000 6.375000000 -23.937000000

H -4.583000000 8.694000000 -23.158000000

H -5.518000000 7.180000000 -23.173000000

H -4.823000000 7.782000000 -21.649000000

H -2.362000000 8.563000000 -23.897000000

H -1.305000000 7.257000000 -23.311000000

H -2.069000000 8.371000000 -22.152000000

N -2.611000000 3.366000000 -20.258000000

C -2.358000000 2.706000000 -18.992000000

C -3.507000000 1.800000000 -18.602000000

O -4.587000000 2.270000000 -18.248000000

H -1.450000000 2.110000000 -19.078000000

H -2.218000000 3.460000000 -18.217000000

H -3.480000000 3.182000000 -20.760000000

O -3.357000000 0.571000000 -18.640000000

N -6.914000000 2.232000000 -21.285000000

C -7.141000000 3.614000000 -21.675000000

C -6.212000000 3.896000000 -22.850000000

O -4.998000000 3.971000000 -22.680000000

C -6.812000000 4.580000000 -20.522000000

C -7.120000000 6.006000000 -20.938000000

C -7.614000000 4.206000000 -19.290000000

H -8.178000000 3.746000000 -21.983000000

H -5.750000000 4.502000000 -20.289000000

H -7.383000000 3.180000000 -19.003000000

H -7.357000000 4.879000000 -18.472000000

H -8.678000000 4.291000000 -19.510000000

H -8.115000000 6.280000000 -20.588000000

H -6.383000000 6.679000000 -20.500000000

H -7.083000000 6.084000000 -22.025000000

H -6.238000000 1.671000000 -21.783000000

O -6.685000000 4.051000000 -23.984000000

H -6.625000000 2.246000000 -20.317000000

N -7.810000000 11.109000000 -24.650000000

C -7.667000000 9.874000000 -23.900000000

C -9.024000000 9.198000000 -23.777000000

O -9.997000000 9.801000000 -23.325000000

C -7.092000000 10.166000000 -22.525000000

H -6.987000000 9.211000000 -24.435000000

H -7.645000000 10.985000000 -22.066000000

H -7.175000000 9.277000000 -21.900000000

H -6.043000000 10.445000000 -22.622000000

H -8.717000000 11.388000000 -24.995000000

H -7.185000000 11.059000000 -25.442000000

N -9.086000000 7.939000000 -24.187000000

H -8.246000000 7.484000000 -24.546000000

C -10.334000000 7.200000000 -24.132000000

C -10.241000000 5.992000000 -23.215000000

O -9.362000000 5.136000000 -23.368000000

C -10.757000000 6.739000000 -25.535000000

C -12.086000000 6.006000000 -25.467000000

C -10.854000000 7.941000000 -26.455000000

H -11.107000000 7.864000000 -23.744000000

H -10.000000000 6.058000000 -25.924000000

H -12.376000000 5.684000000 -26.467000000

H -12.849000000 6.673000000 -25.065000000

H -11.988000000 5.135000000 -24.819000000

H -11.154000000 7.614000000 -27.451000000

H -9.883000000 8.434000000 -26.512000000

H -11.594000000 8.639000000 -26.064000000

N -11.160000000 5.939000000 -22.257000000

C -11.208000000 4.850000000 -21.299000000

C -12.247000000 3.846000000 -21.763000000

O -13.295000000 4.220000000 -22.292000000

C -11.569000000 5.384000000 -19.908000000

C -11.561000000 4.333000000 -18.800000000

C -11.519000000 4.986000000 -17.417000000

C -11.276002000 3.955002000 -16.326000000

N -10.824985000 4.567000000 -15.041916000

H -11.480008000 5.283005000 -14.729028000

H -9.999940000 5.149148000 -15.185136000

H -10.233000000 4.365000000 -21.256000000

H -12.568000000 5.816000000 -19.959000000

H -10.863000000 6.172000000 -19.645000000

H -12.463000000 3.726000000 -18.879000000

H -10.687000000 3.693000000 -18.920000000

H -12.471000000 5.483000000 -17.230000000

H -10.719000000 5.726000000 -17.395000000

H -10.509000000 3.260000000 -16.668000000

H -12.199000000 3.403000000 -16.149000000

H -11.852000000 6.686000000 -22.193000000

O -12.039000000 2.635000000 -21.608000000

N -19.659000000 7.628000000 -11.972000000

C -18.418000000 8.207000000 -12.466000000

C -18.674000000 9.165000000 -13.615000000

O -17.964000000 10.150000000 -13.771000000

C -17.459000000 7.107000000 -12.934000000

C -16.274016000 7.625988000 -13.753024000

C -15.175981000 6.581904000 -13.982010000

O -14.244936000 6.851765000 -14.766005000

O -15.228967000 5.495025000 -13.376952000

H -17.944000000 8.758000000 -11.654000000

H -18.019000000 6.402000000 -13.549000000

H -17.076000000 6.581000000 -12.059000000

H -16.645000000 7.953000000 -14.724000000

H -15.840000000 8.483000000 -13.237000000

H -20.539000000 7.884000000 -12.396000000

O -19.607000000 8.949000000 -14.400000000

H -19.572000000 6.624000000 -12.032000000

N -18.408000000 13.436000000 -13.639000000

C -17.583000000 14.107000000 -14.633000000

C -18.350000000 15.230000000 -15.328000000

O -17.787000000 16.280000000 -15.632000000

C -17.068000000 13.094000000 -15.661000000

C -15.763000000 12.428000000 -15.228000000

O -14.713000000 13.071000000 -15.171000000

N -15.826000000 11.140000000 -14.919000000

H -14.955000000 10.695000000 -14.630000000

H -16.695000000 10.608000000 -14.966000000

H -16.724000000 14.544000000 -14.125000000

H -17.825000000 12.322000000 -15.799000000

H -16.906000000 13.605000000 -16.610000000

H -19.360000000 13.736000000 -13.487000000

H -18.448000000 12.462000000 -13.903000000

O -19.552000000 15.081000000 -15.588000000

N -9.636000000 14.455000000 -21.276000000

H -10.171000000 14.803000000 -20.480000000

C -8.474000000 15.205000000 -21.731000000

C -7.220000000 14.350000000 -21.754000000

O -7.030000000 13.486000000 -20.901000000

C -8.221000000 16.412000000 -20.820000000

C -7.118000000 17.331000000 -21.327000000

C -6.586000000 18.273000000 -20.262000000

O -7.384000000 19.025000000 -19.657000000

O -5.359000000 18.261000000 -20.035000000

H -8.667000000 15.566000000 -22.741000000

H -9.143000000 16.988000000 -20.745000000

H -7.950000000 16.052000000 -19.827000000

H -7.515000000 17.927000000 -22.149000000

H -6.295000000 16.721000000 -21.700000000

H -10.274000000 14.396000000 -22.057000000

N -6.362000000 14.589000000 -22.735000000

H -6.577000000 15.295000000 -23.439000000

C -5.117000000 13.848000000 -22.810000000

C -4.047062000 14.649011000 -22.082921000

O -3.991000000 15.874000000 -22.210000000

C -4.678000000 13.637000000 -24.256000000

C -3.285000000 13.061000000 -24.334000000

C -2.980000000 11.863000000 -23.704000000

C -2.267000000 13.732000000 -24.994000000

C -1.696000000 11.347000000 -23.725000000

C -0.975000000 13.224000000 -25.022000000

C -0.697000000 12.030000000 -24.382000000

O 0.585000000 11.521000000 -24.383000000

H 1.274000000 11.992000000 -24.836000000

H -5.240000000 12.881000000 -22.323000000

H -5.372000000 12.950000000 -24.740000000

H -4.699000000 14.593000000 -24.779000000

H -3.760000000 11.323000000 -23.187000000

H -2.482000000 14.665000000 -25.494000000

H -1.479000000 10.413000000 -23.228000000

H -0.192000000 13.758000000 -25.540000000

N -3.201792000 13.961958000 -21.321325000

C -2.138050000 14.627009000 -20.577936000

C -0.785000000 14.060000000 -21.008000000

O -0.324000000 13.047000000 -20.490000000

C -2.346000000 14.435000000 -19.063000000

S -3.922000000 15.086000000 -18.375000000

H -2.162000000 15.693000000 -20.805000000

H -2.308000000 13.366000000 -18.854000000

H -1.519000000 14.916000000 -18.540000000

H -4.389000000 16.054000000 -19.169000000

H -3.300093000 12.949034000 -21.254811000

O -0.141000000 14.634000000 -21.897000000

N 2.386000000 11.001000000 -15.462000000

C 1.941000000 10.985000000 -14.075000000

C 2.933000000 11.709000000 -13.173000000

O 4.133000000 11.724000000 -13.433000000

C 1.763000000 9.545000000 -13.592000000

C 0.936000000 8.685000000 -14.530000000

C 0.578000000 7.333000000 -13.943000000

O 1.397000000 6.783000000 -13.176000000

O -0.520000000 6.819000000 -14.264000000

H 0.979000000 11.494000000 -14.013000000

H 1.270000000 9.567000000 -12.620000000

H 2.747000000 9.090000000 -13.476000000

H 0.013000000 9.216000000 -14.764000000

H 1.496000000 8.531000000 -15.452000000

H 3.253000000 11.456000000 -15.709000000

H 1.653000000 11.424000000 -16.013000000

O 2.522000000 12.292000000 -12.160000000

N -2.154000000 13.882000000 -12.921000000

C -1.496000000 13.980000000 -14.208000000

C -0.940000000 15.358000000 -14.520000000

O -1.238000000 16.336000000 -13.849000000

C -2.456000000 13.539000000 -15.313000000

C -3.008000000 12.130000000 -15.078000000

C -3.818000000 11.709000000 -16.275000000

C -1.871000000 11.156000000 -14.838000000

H -0.659000000 13.282000000 -14.203000000

H -1.924000000 13.552000000 -16.264000000

H -3.288000000 14.242000000 -15.362000000

H -3.654000000 12.144000000 -14.200000000

H -4.213000000 10.706000000 -16.112000000

H -4.644000000 12.406000000 -16.418000000

H -3.184000000 11.710000000 -17.162000000

H -2.184000000 10.153000000 -15.127000000

H -1.007000000 11.452000000 -15.433000000

H -1.603000000 11.163000000 -13.781000000

H -2.211000000 14.692000000 -12.320000000

H -3.096000000 13.556000000 -13.084000000

O -0.166000000 15.495000000 -15.477000000

N -7.232000000 13.290000000 -11.662000000

C -7.918999000 12.392010000 -12.581002000

C -8.383000000 11.096000000 -11.941000000

O -7.817000000 10.651000000 -10.947000000

C -7.016457000 12.058070000 -13.774449000

O -6.332123000 10.826945000 -13.588912000

H -5.773000000 10.619000000 -14.328000000

H -8.799000000 12.911000000 -12.962000000

H -6.283923000 12.853922000 -13.896046000

H -7.627702000 11.991681000 -14.674430000

H -7.104000000 13.023000000 -10.696000000

H -6.311000000 13.450000000 -12.045000000

N -9.409000000 10.496000000 -12.542000000

H -9.805000000 10.944000000 -13.369000000

C -10.005000000 9.238000000 -12.095000000

C -11.042998000 9.433015000 -10.996001000

O -10.729000000 9.410000000 -9.804000000

C -8.935000000 8.242000000 -11.633000000

C -9.461000000 6.806000000 -11.586000000

O -8.656000000 5.868000000 -11.368000000

O -10.685000000 6.617000000 -11.771000000

H -10.515000000 8.796000000 -12.951000000

H -8.094000000 8.285000000 -12.325000000

H -8.591000000 8.527000000 -10.639000000

N -12.293109000 9.619935000 -11.406977000

C -13.366997000 9.829021000 -10.459001000

C -14.265000000 8.614000000 -10.320000000

O -15.444000000 8.727000000 -9.974000000

C -14.166000000 11.065000000 -10.864000000

C -13.354999000 12.319999000 -10.833000000

C -12.522039000 12.648025000 -11.889017000

C -13.352000000 13.128000000 -9.704000000

C -11.702996000 13.747997000 -11.815999000

C -12.526000000 14.234000000 -9.632000000

C -11.704000000 14.540000000 -10.684000000

H -12.919000000 10.026000000 -9.485000000

H -14.542000000 10.919000000 -11.877000000

H -15.013000000 11.175000000 -10.186000000

H -12.515950000 12.033934000 -12.777039000

H -14.001000000 12.890000000 -8.874000000

H -11.057000000 13.994000000 -12.645000000

H -12.529000000 14.855000000 -8.749000000

H -11.056000000 15.402000000 -10.628000000

H -12.498856000 9.616052000 -12.406037000

O -13.806000000 7.488000000 -10.558000000

C -4.143866000 7.992606000 -18.209026000

C -2.730820000 8.006567000 -18.438344000

C -4.678284000 6.895283000 -17.497943000

N -1.897599000 7.054087000 -17.944389000

C -3.816233000 5.934875000 -17.009878000

C -2.432178000 6.073990000 -17.237203000

C -4.894924000 9.112012000 -18.702939000

C -4.224494000 10.120795000 -19.423512000

C -2.117091000 9.051566000 -19.209277000

C -2.837293000 10.095912000 -19.714537000

C -6.238273000 9.605767000 -18.629054000

N -6.371871000 10.783291000 -19.221161000

N -5.155465000 11.074223000 -19.709689000

H -5.018883000 11.965198000 -20.152588000

S -7.678755000 8.996975000 -17.742548000

O -7.766536000 7.543998000 -17.978435000

O -7.614458000 9.513430000 -16.367042000

C -9.049764000 9.781538000 -18.587208000

C -9.719915000 9.077289000 -19.585760000

C -10.788669000 9.683741000 -20.239592000

C -11.182786000 10.990612000 -19.921481000

C -12.344193000 11.639545000 -20.633687000

C -10.490916000 11.672783000 -18.909267000

C -9.431624000 11.075654000 -18.232058000

H -5.749584000 6.816429000 -17.350271000

H -4.191785000 5.082043000 -16.453773000

H -1.735650000 5.346248000 -16.824096000

H -1.047003000 8.972620000 -19.365560000

H -2.368694000 10.884262000 -20.293725000

H -9.407719000 8.072020000 -19.834151000

H -11.322855000 9.133449000 -21.009344000

H -10.787380000 12.685179000 -18.648516000

H -8.904651000 11.598717000 -17.442215000

H -12.440924000 11.269458000 -21.658364000

H -13.289025000 11.426252000 -20.118715000

H -12.232699000 12.726845000 -20.671856000

H 0.434000000 15.601000000 -16.219000000

H 2.204000000 12.744000000 -11.375000000

H -7.996000000 19.601000000 -19.193000000

H -20.483000000 14.966000000 -15.789000000

H -13.528020000 7.060546000 -15.370026000

H -8.040000000 5.151000000 -11.201000000

H -11.878000000 1.696000000 -21.488000000

H -13.450000000 6.616000000 -10.742000000

H 2.026000000 6.360000000 -12.587000000

H 0.358000000 15.079000000 -22.586000000

H -3.241000000 -0.381000000 -18.669000000

H -7.052000000 4.171000000 -24.863000000

H -20.330000000 8.782000000 -15.009000000

# **Cartesian coordinates of azoles 1‑7 with corresponding residues around 4 Å after optimization with DFT formalism** (ligands and functionalities of amino acids optimization, B3LYP/6-31G(d,p) level of theory, results given in the Table 4, ***Gaussian G16.A01* software**)

Azole **1** with surrounded residues

C -5.561682000 9.057591000 -18.076509000

C -4.184048000 8.899041000 -18.430264000

C -3.981240000 9.879864000 -19.427797000

N -5.191552000 10.501936000 -19.593709000

N -6.149677000 10.014355000 -18.767566000

C -2.723346000 10.078615000 -20.015681000

C -1.685111000 9.275853000 -19.577962000

C -1.894177000 8.288461000 -18.588065000

C -3.126753000 8.079472000 -18.000185000

H -5.421082000 11.274375000 -20.197814000

Cl -0.524489000 7.301654000 -18.101462000

S -6.311447000 8.151067000 -16.727864000

O -5.530518000 8.516788000 -15.535208000

O -6.358356000 6.735516000 -17.122648000

C -7.994904000 8.739504000 -16.524452000

C -8.318596000 10.095265000 -16.470104000

C -9.599280000 10.468215000 -16.067217000

C -10.563229000 9.509176000 -15.723646000

C -11.964640000 9.908158000 -15.330558000

C -10.213247000 8.156093000 -15.807039000

C -8.939788000 7.763417000 -16.208857000

H -2.553510000 10.816822000 -20.788126000

H -0.694835000 9.392161000 -20.003016000

H -3.271221000 7.326297000 -17.235863000

H -7.583987000 10.843100000 -16.741436000

H -9.857347000 11.521493000 -16.012424000

H -10.952777000 7.406207000 -15.544744000

H -8.654985000 6.720388000 -16.275458000

H -12.014166000 10.947917000 -14.998664000

H -12.353795000 9.271901000 -14.533609000

H -12.653564000 9.794998000 -16.174897000

C -7.273073000 14.348123000 -21.773334000

O -7.108535000 13.467585000 -20.941766000

O -6.338643000 10.836702000 -13.597367000

H -6.921992000 10.120815000 -13.960453000

N -2.564000000 3.856000000 -23.064000000

C -2.089000000 4.847000000 -22.104000000

C -1.721000000 4.207000000 -20.769000000

O -0.655000000 4.475000000 -20.213000000

C -3.161000000 5.910000000 -21.873000000

C -3.399000000 6.915000000 -22.996000000

C -4.670000000 7.698000000 -22.725000000

C -2.199000000 7.842000000 -23.096000000

H -2.738000000 2.956000000 -22.558000000

H -1.922000000 3.695000000 -23.837000000

H -1.157000000 5.341000000 -22.504000000

H -2.906000000 6.459000000 -20.934000000

H -4.119000000 5.407000000 -21.589000000

H -3.523000000 6.368000000 -23.964000000

H -4.799000000 8.506000000 -23.450000000

H -4.657000000 8.145000000 -21.720000000

H -5.548000000 7.038000000 -22.789000000

H -2.274000000 8.508000000 -23.963000000

H -1.256000000 7.293000000 -23.201000000

H -2.103000000 8.475000000 -22.203000000

H -3.546000000 4.121000000 -23.427000000

O -2.504000000 3.408000000 -20.237000000

N -6.914000000 2.232000000 -21.285000000

C -7.141000000 3.614000000 -21.675000000

C -6.212000000 3.896000000 -22.850000000

O -4.998000000 3.971000000 -22.680000000

C -6.812000000 4.580000000 -20.522000000

C -7.120000000 6.006000000 -20.938000000

C -7.614000000 4.206000000 -19.290000000

H -8.212000000 3.774000000 -22.024000000

H -5.709000000 4.511000000 -20.290000000

H -7.286000000 3.282000000 -18.809000000

H -8.692000000 4.161000000 -19.487000000

H -7.479000000 4.991000000 -18.510000000

H -8.192000000 6.155000000 -21.121000000

H -6.813000000 6.715000000 -20.156000000

H -6.592000000 6.272000000 -21.864000000

H -7.251000000 2.042000000 -20.320000000

H -7.398000000 1.581000000 -21.930000000

O -6.685000000 4.051000000 -23.984000000

N -7.810000000 11.109000000 -24.650000000

C -7.667000000 9.874000000 -23.900000000

C -9.024000000 9.198000000 -23.777000000

O -9.997000000 9.801000000 -23.325000000

C -7.092000000 10.166000000 -22.525000000

H -6.933000000 9.191000000 -24.444000000

H -7.640000000 10.962000000 -21.984000000

H -6.031000000 10.462000000 -22.547000000

H -7.142000000 9.286000000 -21.857000000

H -8.530000000 11.726000000 -24.161000000

H -8.125000000 10.968000000 -25.620000000

N -9.086000000 7.939000000 -24.187000000

H -8.264000000 7.347000000 -24.381000000

C -10.334000000 7.200000000 -24.132000000

C -10.241000000 5.992000000 -23.215000000

O -9.362000000 5.136000000 -23.368000000

C -10.757000000 6.739000000 -25.535000000

C -12.086000000 6.006000000 -25.467000000

C -10.854000000 7.941000000 -26.455000000

H -11.146000000 7.897000000 -23.752000000

H -9.979000000 6.025000000 -25.925000000

H -11.260000000 7.649000000 -27.435000000

H -11.525000000 8.711000000 -26.059000000

H -9.881000000 8.401000000 -26.646000000

H -12.385000000 5.622000000 -26.450000000

H -12.050000000 5.120000000 -24.800000000

H -12.904000000 6.634000000 -25.100000000

N -11.160000000 5.939000000 -22.257000000

C -11.208000000 4.850000000 -21.299000000

C -12.247000000 3.846000000 -21.763000000

O -13.295000000 4.220000000 -22.292000000

C -11.569000000 5.384000000 -19.908000000

C -11.561000000 4.333000000 -18.800000000

C -11.519000000 4.986000000 -17.417000000

C -11.276000000 3.955000000 -16.326000000

N -10.825000000 4.567000000 -15.042000000

H -10.594000000 3.862000000 -14.318000000

H -9.999000000 5.174000000 -15.162000000

H -10.209000000 4.335000000 -21.263000000

H -12.583000000 5.844000000 -19.953000000

H -10.870000000 6.206000000 -19.653000000

H -12.459000000 3.682000000 -18.912000000

H -10.706000000 3.645000000 -18.943000000

H -10.737000000 5.772000000 -17.408000000

H -12.483000000 5.526000000 -17.232000000

H -12.228000000 3.386000000 -16.135000000

H -10.521000000 3.204000000 -16.656000000

H -12.027000000 6.473000000 -22.315000000

O -12.039000000 2.635000000 -21.608000000

N -19.659000000 7.628000000 -11.972000000

C -18.418000000 8.207000000 -12.466000000

C -18.674000000 9.165000000 -13.615000000

O -17.964000000 10.150000000 -13.771000000

C -17.459000000 7.107000000 -12.934000000

C -16.274000000 7.626000000 -13.753000000

C -15.176000000 6.582000000 -13.982000000

O -14.245000000 6.852000000 -14.766000000

O -15.229000000 5.495000000 -13.377000000

H -17.874000000 8.770000000 -11.635000000

H -17.006000000 6.573000000 -12.055000000

H -17.984000000 6.342000000 -13.536000000

H -15.793000000 8.469000000 -13.213000000

H -16.603000000 8.014000000 -14.731000000

H -20.138000000 8.254000000 -11.314000000

H -19.485000000 6.722000000 -11.500000000

O -19.607000000 8.949000000 -14.400000000

N -18.408000000 13.436000000 -13.639000000

C -17.583000000 14.107000000 -14.633000000

C -18.350000000 15.230000000 -15.328000000

O -17.787000000 16.280000000 -15.632000000

C -17.068000000 13.094000000 -15.661000000

C -15.763000000 12.428000000 -15.228000000

O -14.713000000 13.071000000 -15.171000000

N -15.826000000 11.140000000 -14.919000000

H -15.033000000 10.642000000 -14.550000000

H -16.709000000 10.611000000 -14.872000000

H -16.681000000 14.591000000 -14.140000000

H -17.851000000 12.359000000 -15.939000000

H -16.858000000 13.643000000 -16.614000000

H -19.417000000 13.569000000 -13.906000000

H -18.271000000 13.809000000 -12.695000000

O -19.552000000 15.081000000 -15.588000000

N -8.842000000 16.763000000 -14.873000000

C -8.904000000 16.251000000 -16.233000000

C -10.236000000 16.661000000 -16.839000000

O -11.274000000 16.583000000 -16.184000000

C -8.757000000 14.730000000 -16.248000000

C -9.094000000 14.181000000 -17.629000000

C -7.333000000 14.369000000 -15.868000000

H -8.096000000 16.723000000 -16.883000000

H -9.484000000 14.281000000 -15.522000000

H -9.013000000 13.089000000 -17.656000000

H -8.410000000 14.582000000 -18.397000000

H -10.118000000 14.461000000 -17.922000000

H -6.602000000 14.949000000 -16.472000000

H -7.095000000 13.329000000 -16.133000000

H -7.098000000 14.538000000 -14.821000000

H -8.216000000 16.234000000 -14.264000000

H -8.540000000 17.759000000 -14.877000000

O -10.277000000 17.078000000 -18.005000000

N -11.852000000 11.728000000 -22.235000000

C -11.252000000 12.660000000 -21.292000000

C -10.018000000 13.331000000 -21.868000000

O -9.420000000 12.837000000 -22.819000000

C -10.880000000 11.902000000 -20.021000000

C -12.053000000 11.078000000 -19.490000000

C -11.581000000 9.694000000 -19.097000000

C -12.706000000 11.813000000 -18.329000000

H -12.024000000 13.456000000 -21.021000000

H -10.515000000 12.607000000 -19.236000000

H -9.991000000 11.254000000 -20.199000000

H -12.860000000 10.938000000 -20.265000000

H -13.604000000 11.290000000 -17.962000000

H -12.039000000 11.897000000 -17.458000000

H -13.021000000 12.833000000 -18.583000000

H -12.402000000 9.086000000 -18.685000000

H -11.148000000 9.133000000 -19.931000000

H -10.814000000 9.731000000 -18.305000000

H -12.616000000 11.163000000 -21.780000000

H -12.232000000 12.205000000 -23.067000000

N -9.636000000 14.455000000 -21.276000000

H -10.056000000 14.810000000 -20.402000000

C -8.474000000 15.205000000 -21.731000000

C -8.221000000 16.412000000 -20.820000000

C -7.118000000 17.331000000 -21.327000000

C -6.586000000 18.273000000 -20.262000000

O -7.384000000 19.025000000 -19.657000000

O -5.359000000 18.261000000 -20.035000000

H -8.702000000 15.579000000 -22.774000000

H -9.144000000 17.022000000 -20.668000000

H -7.955000000 16.076000000 -19.791000000

H -7.478000000 17.957000000 -22.162000000

H -6.227000000 16.779000000 -21.689000000

N -6.362000000 14.589000000 -22.735000000

H -6.339000000 15.508000000 -23.208000000

C -5.117000000 13.848000000 -22.810000000

C -4.047000000 14.649000000 -22.083000000

O -3.991000000 15.874000000 -22.210000000

C -4.678000000 13.637000000 -24.256000000

C -3.285000000 13.061000000 -24.334000000

C -2.980000000 11.863000000 -23.704000000

C -2.267000000 13.732000000 -24.994000000

C -1.696000000 11.347000000 -23.725000000

C -0.975000000 13.224000000 -25.022000000

C -0.697000000 12.030000000 -24.382000000

O 0.585000000 11.521000000 -24.383000000

H 1.062000000 11.931000000 -23.604000000

H -5.266000000 12.846000000 -22.315000000

H -5.388000000 12.988000000 -24.795000000

H -4.702000000 14.615000000 -24.794000000

H -3.755000000 11.310000000 -23.177000000

H -2.462000000 14.700000000 -25.461000000

H -1.452000000 10.428000000 -23.190000000

H -0.170000000 13.787000000 -25.497000000

N -3.202000000 13.962000000 -21.321000000

C -2.138000000 14.627000000 -20.578000000

C -0.785000000 14.060000000 -21.008000000

O -0.324000000 13.047000000 -20.490000000

C -2.346000000 14.435000000 -19.063000000

S -3.922000000 15.086000000 -18.375000000

H -2.131000000 15.732000000 -20.832000000

H -1.497000000 14.926000000 -18.533000000

H -2.289000000 13.362000000 -18.804000000

H -4.089000000 16.235000000 -19.092000000

H -3.193000000 12.954000000 -21.264000000

O -0.141000000 14.634000000 -21.897000000

N -2.154000000 13.882000000 -12.921000000

C -1.496000000 13.980000000 -14.208000000

C -0.940000000 15.358000000 -14.520000000

O -1.238000000 16.336000000 -13.849000000

C -2.456000000 13.539000000 -15.313000000

C -3.008000000 12.130000000 -15.078000000

C -3.818000000 11.709000000 -16.275000000

C -1.871000000 11.156000000 -14.838000000

H -0.592000000 13.295000000 -14.231000000

H -3.266000000 14.278000000 -15.459000000

H -1.898000000 13.586000000 -16.289000000

H -3.689000000 12.120000000 -14.188000000

H -4.282000000 10.722000000 -16.127000000

H -4.621000000 12.416000000 -16.512000000

H -3.199000000 11.627000000 -17.184000000

H -2.232000000 10.115000000 -14.863000000

H -1.107000000 11.228000000 -15.631000000

H -1.376000000 11.287000000 -13.876000000

H -1.580000000 13.379000000 -12.229000000

H -3.070000000 13.416000000 -12.971000000

O -0.166000000 15.495000000 -15.477000000

N -7.232000000 13.290000000 -11.662000000

C -7.919000000 12.392000000 -12.581000000

C -8.383000000 11.096000000 -11.941000000

O -7.817000000 10.651000000 -10.947000000

C -7.017000000 12.057000000 -13.774000000

H -8.838000000 12.935000000 -12.973000000

H -7.620000000 12.015000000 -14.706000000

H -6.199000000 12.792000000 -13.914000000

H -6.291000000 13.552000000 -11.993000000

H -7.774000000 14.143000000 -11.468000000

N -9.409000000 10.496000000 -12.542000000

H -9.888000000 10.926000000 -13.329000000

C -10.005000000 9.238000000 -12.095000000

C -11.043000000 9.433000000 -10.996000000

O -10.729000000 9.410000000 -9.804000000

C -8.935000000 8.242000000 -11.633000000

C -9.461000000 6.806000000 -11.586000000

O -8.656000000 5.868000000 -11.368000000

O -10.685000000 6.617000000 -11.771000000

H -10.506000000 8.781000000 -13.006000000

H -8.035000000 8.279000000 -12.262000000

H -8.595000000 8.480000000 -10.599000000

N -12.293000000 9.620000000 -11.407000000

C -13.367000000 9.829000000 -10.459000000

C -14.265000000 8.614000000 -10.320000000

O -15.444000000 8.727000000 -9.974000000

C -14.166000000 11.065000000 -10.864000000

C -13.355000000 12.320000000 -10.833000000

C -12.522000000 12.648000000 -11.889000000

C -13.352000000 13.128000000 -9.704000000

C -11.703000000 13.748000000 -11.816000000

C -12.526000000 14.234000000 -9.632000000

C -11.704000000 14.540000000 -10.684000000

H -12.932000000 9.996000000 -9.426000000

H -14.613000000 10.907000000 -11.869000000

H -15.051000000 11.124000000 -10.187000000

H -12.531000000 12.025000000 -12.788000000

H -13.993000000 12.872000000 -8.858000000

H -11.082000000 14.018000000 -12.666000000

H -12.532000000 14.857000000 -8.738000000

H -11.067000000 15.419000000 -10.636000000

H -12.610000000 9.110000000 -12.245000000

O -13.806000000 7.488000000 -10.558000000

H -20.483000000 14.966000000 -15.789000000

H -13.450000000 6.616000000 -10.742000000

H 0.434000000 15.601000000 -16.219000000

H -3.111000000 2.789000000 -19.825000000

H -8.040000000 5.151000000 -11.201000000

H -11.878000000 1.696000000 -21.488000000

H 0.358000000 15.079000000 -22.586000000

H -4.415000000 18.252000000 -19.860000000

H -13.528000000 7.060000000 -15.370000000

H -10.309000000 17.401000000 -18.908000000

H -20.330000000 8.782000000 -15.009000000

Azole **2** with surrounded residues

C -2.956714000 8.235354000 -17.942085000

C -1.730397000 8.393564000 -18.573925000

C -1.582402000 9.320769000 -19.641081000

C -2.634149000 10.099062000 -20.086768000

C -3.879137000 9.916662000 -19.466796000

N -5.109958000 10.492769000 -19.655800000

H -5.368276000 11.223930000 -20.298260000

N -6.053330000 10.000994000 -18.816985000

C -5.428599000 9.104963000 -18.076511000

C -4.043701000 8.984544000 -18.417843000

N -0.617647000 7.615710000 -18.175157000

C -0.677831000 6.338263000 -17.640791000

C 0.604191000 5.908387000 -17.400755000

C 1.487458000 6.954830000 -17.798386000

C 0.714079000 7.986982000 -18.269973000

S -6.184983000 8.129726000 -16.782388000

O -6.269426000 6.744116000 -17.266735000

O -5.386679000 8.393138000 -15.573354000

C -7.858767000 8.733131000 -16.538937000

C -8.807913000 7.759332000 -16.225302000

C -10.079853000 8.159349000 -15.829453000

C -10.430803000 9.511804000 -15.758077000

C -9.467536000 10.468210000 -16.109735000

C -11.836907000 9.897760000 -15.369614000

C -8.181815000 10.089308000 -16.493890000

H -3.073966000 7.573999000 -17.093006000

H -0.620430000 9.403014000 -20.133569000

H -2.490479000 10.798186000 -20.900218000

H -1.624230000 5.835167000 -17.520838000

H 0.878149000 4.945679000 -16.993348000

H 2.566061000 6.957631000 -17.729993000

H 0.988522000 8.966662000 -18.623251000

H -8.534593000 6.712752000 -16.290758000

H -10.826661000 7.417599000 -15.570254000

H -9.725378000 11.523200000 -16.078879000

H -7.445404000 10.835765000 -16.764742000

H -12.251410000 9.192536000 -14.646300000

H -11.887467000 10.904849000 -14.948702000

H -12.505944000 9.865677000 -16.235187000

C -7.256267000 14.364610000 -21.750557000

O -7.038136000 13.560753000 -20.856116000

O -6.309272000 10.861646000 -13.575051000

H -5.845139000 10.673516000 -14.428561000

N -6.914000000 2.232000000 -21.285000000

C -7.141000000 3.614000000 -21.675000000

C -6.212000000 3.896000000 -22.850000000

O -4.998000000 3.971000000 -22.680000000

C -6.812000000 4.580000000 -20.522000000

C -7.120000000 6.006000000 -20.938000000

C -7.614000000 4.206000000 -19.290000000

H -8.178000000 3.746000000 -21.983000000

H -5.750000000 4.502000000 -20.289000000

H -7.383000000 3.180000000 -19.003000000

H -7.357000000 4.879000000 -18.472000000

H -8.678000000 4.291000000 -19.510000000

H -8.115000000 6.280000000 -20.588000000

H -7.083000000 6.083000000 -22.025000000

H -6.383000000 6.679000000 -20.501000000

H -6.238000000 1.671000000 -21.783000000

H -6.653000000 2.229000000 -20.309000000

O -6.685000000 4.051000000 -23.984000000

N -7.810000000 11.109000000 -24.650000000

C -7.667000000 9.874000000 -23.900000000

C -9.024000000 9.198000000 -23.777000000

O -9.997000000 9.801000000 -23.325000000

C -7.092000000 10.166000000 -22.525000000

H -6.987000000 9.211000000 -24.435000000

H -7.645000000 10.985000000 -22.066000000

H -7.175000000 9.277000000 -21.900000000

H -6.043000000 10.445000000 -22.622000000

H -8.717000000 11.388000000 -24.995000000

H -7.002000000 11.689000000 -24.827000000

N -9.086000000 7.939000000 -24.187000000

H -8.246000000 7.484000000 -24.546000000

C -10.334000000 7.200000000 -24.132000000

C -10.241000000 5.992000000 -23.215000000

O -9.362000000 5.136000000 -23.368000000

C -10.757000000 6.739000000 -25.535000000

C -12.086000000 6.006000000 -25.467000000

C -10.854000000 7.941000000 -26.455000000

H -11.107000000 7.864000000 -23.744000000

H -10.000000000 6.058000000 -25.924000000

H -12.376000000 5.684000000 -26.467000000

H -12.849000000 6.673000000 -25.065000000

H -11.988000000 5.135000000 -24.819000000

H -11.154000000 7.614000000 -27.451000000

H -9.883000000 8.434000000 -26.512000000

H -11.594000000 8.639000000 -26.064000000

N -11.160000000 5.939000000 -22.257000000

C -11.208000000 4.850000000 -21.299000000

C -12.247000000 3.846000000 -21.763000000

O -13.295000000 4.220000000 -22.292000000

C -11.569000000 5.384000000 -19.908000000

C -11.561000000 4.333000000 -18.800000000

C -11.519000000 4.986000000 -17.417000000

C -11.276000000 3.955000000 -16.326000000

N -10.825000000 4.567000000 -15.042000000

H -11.480000000 5.283000000 -14.729000000

H -10.000000000 5.149000000 -15.185000000

H -10.233000000 4.365000000 -21.256000000

H -12.568000000 5.816000000 -19.959000000

H -10.863000000 6.172000000 -19.645000000

H -10.684000000 3.697000000 -18.920000000

H -12.460000000 3.721000000 -18.880000000

H -10.715000000 5.721000000 -17.396000000

H -12.468000000 5.488000000 -17.230000000

H -12.204000000 3.413000000 -16.146000000

H -10.517000000 3.251000000 -16.668000000

H -11.852000000 6.686000000 -22.193000000

O -12.039000000 2.635000000 -21.608000000

N -19.659000000 7.628000000 -11.972000000

C -18.418000000 8.207000000 -12.466000000

C -18.674000000 9.165000000 -13.615000000

O -17.964000000 10.150000000 -13.771000000

C -17.459000000 7.107000000 -12.934000000

C -16.274000000 7.626000000 -13.753000000

C -15.176000000 6.582000000 -13.982000000

O -14.245000000 6.852000000 -14.766000000

O -15.229000000 5.495000000 -13.377000000

H -17.944000000 8.758000000 -11.654000000

H -17.070000000 6.594000000 -12.055000000

H -18.016000000 6.391000000 -13.539000000

H -16.645000000 7.953000000 -14.724000000

H -15.840000000 8.483000000 -13.237000000

H -19.640000000 6.971000000 -11.206000000

H -19.753000000 8.390000000 -11.316000000

N -19.695000000 8.880000000 -14.415000000

H -20.267000000 8.057000000 -14.226000000

C -20.010000000 9.725000000 -15.559000000

C -20.763000000 10.996000000 -15.182000000

O -20.455000000 12.080000000 -15.689000000

C -20.822000000 8.943000000 -16.620000000

C -19.966000000 7.816000000 -17.197000000

C -21.253000000 9.870000000 -17.747000000

C -20.730000000 6.885000000 -18.097000000

H -19.068000000 10.023000000 -16.018000000

H -21.707000000 8.515000000 -16.150000000

H -19.555000000 7.237000000 -16.370000000

H -19.143000000 8.254000000 -17.762000000

H -21.864000000 10.676000000 -17.341000000

H -21.833000000 9.307000000 -18.478000000

H -20.371000000 10.290000000 -18.229000000

H -20.061000000 6.110000000 -18.471000000

H -21.140000000 7.447000000 -18.936000000

H -21.543000000 6.424000000 -17.537000000

O -21.954000000 10.799000000 -15.461000000

N -20.645000000 12.444000000 -12.393000000

H -20.503000000 11.434000000 -12.409000000

C -19.746000000 13.290000000 -11.621000000

C -18.885000000 14.091000000 -12.588000000

O -18.670000000 15.284000000 -12.396000000

C -18.839000000 12.460000000 -10.681000000

C -19.665000000 11.940000000 -9.504000000

C -17.676000000 13.309000000 -10.172000000

C -18.835000000 11.380000000 -8.374000000

H -20.339000000 13.980000000 -11.021000000

H -18.440000000 11.610000000 -11.235000000

H -20.264000000 12.764000000 -9.115000000

H -20.336000000 11.160000000 -9.865000000

H -17.348000000 13.986000000 -10.960000000

H -18.000000000 13.888000000 -9.307000000

H -16.850000000 12.659000000 -9.885000000

H -19.432000000 10.670000000 -7.801000000

H -18.513000000 12.193000000 -7.723000000

H -17.960000000 10.873000000 -8.782000000

H -21.407000000 12.856000000 -12.911000000

N -18.408000000 13.436000000 -13.639000000

C -17.583000000 14.107000000 -14.633000000

C -18.350000000 15.230000000 -15.328000000

O -17.787000000 16.280000000 -15.632000000

C -17.068000000 13.094000000 -15.661000000

C -15.763000000 12.428000000 -15.228000000

O -14.713000000 13.071000000 -15.171000000

N -15.826000000 11.140000000 -14.919000000

H -14.955000000 10.695000000 -14.630000000

H -16.695000000 10.608000000 -14.966000000

H -16.724000000 14.544000000 -14.125000000

H -17.825000000 12.322000000 -15.799000000

H -16.906000000 13.605000000 -16.610000000

H -18.623000000 12.445000000 -13.752000000

O -19.315000000 15.521000000 -14.608000000

N -8.329000000 19.671000000 -13.192000000

C -9.139000000 18.465000000 -13.171000000

C -9.230000000 18.004000000 -14.608000000

O -9.634000000 18.773000000 -15.475000000

C -10.568000000 18.741000000 -12.672000000

C -11.366000000 17.446000000 -12.648000000

C -10.531000000 19.360000000 -11.300000000

H -8.664000000 17.700000000 -12.557000000

H -11.050000000 19.437000000 -13.358000000

H -11.383000000 17.011000000 -13.647000000

H -10.900000000 16.745000000 -11.955000000

H -12.386000000 17.654000000 -12.324000000

H -9.956000000 20.286000000 -11.334000000

H -10.062000000 18.667000000 -10.602000000

H -11.548000000 19.575000000 -10.971000000

H -7.964000000 20.019000000 -14.067000000

H -7.546000000 19.523000000 -12.572000000

N -8.842000000 16.763000000 -14.873000000

H -8.499000000 16.166000000 -14.120000000

C -8.904000000 16.251000000 -16.233000000

C -10.236000000 16.661000000 -16.839000000

O -11.274000000 16.583000000 -16.184000000

C -8.757000000 14.730000000 -16.248000000

C -9.094000000 14.181000000 -17.629000000

C -7.333000000 14.369000000 -15.868000000

H -8.096000000 16.693000000 -16.817000000

H -9.441000000 14.299000000 -15.517000000

H -8.581000000 13.231000000 -17.778000000

H -8.771000000 14.891000000 -18.391000000

H -10.171000000 14.029000000 -17.707000000

H -6.638000000 15.006000000 -16.415000000

H -7.195000000 14.516000000 -14.797000000

H -7.143000000 13.325000000 -16.119000000

N -10.203000000 17.119000000 -18.084000000

C -11.418000000 17.577000000 -18.747000000

C -12.251000000 16.444000000 -19.334000000

O -11.720000000 15.508000000 -19.927000000

C -11.058000000 18.592000000 -19.838000000

C -12.229000000 19.425000000 -20.319000000

C -11.813000000 20.377000000 -21.430000000

C -10.778000000 21.389000000 -20.949000000

N -10.456000000 22.388000000 -22.019000000

H -9.764000000 23.065000000 -21.697000000

H -11.297000000 22.849000000 -22.367000000

H -12.029000000 18.089000000 -18.004000000

H -10.299000000 19.266000000 -19.441000000

H -10.638000000 18.056000000 -20.689000000

H -12.619000000 20.005000000 -19.483000000

H -13.011000000 18.762000000 -20.690000000

H -11.387000000 19.798000000 -22.249000000

H -12.693000000 20.910000000 -21.790000000

H -9.866000000 20.860000000 -20.671000000

H -11.168000000 21.913000000 -20.076000000

H -9.315000000 17.151000000 -18.584000000

O -13.483000000 16.467000000 -19.211000000

N -11.852000000 11.728000000 -22.235000000

C -11.252000000 12.660000000 -21.292000000

C -10.018000000 13.331000000 -21.868000000

O -9.420000000 12.837000000 -22.819000000

C -10.880000000 11.902000000 -20.021000000

C -12.053000000 11.078000000 -19.490000000

C -11.581000000 9.694000000 -19.097000000

C -12.706000000 11.813000000 -18.329000000

H -11.985000000 13.427000000 -21.042000000

H -10.578000000 12.619000000 -19.258000000

H -10.044000000 11.236000000 -20.235000000

H -12.789000000 10.975000000 -20.288000000

H -12.424000000 9.115000000 -18.720000000

H -11.158000000 9.194000000 -19.968000000

H -10.821000000 9.776000000 -18.320000000

H -13.542000000 11.225000000 -17.951000000

H -11.975000000 11.958000000 -17.534000000

H -13.068000000 12.783000000 -18.671000000

H -12.801000000 12.038000000 -22.387000000

H -11.894000000 10.824000000 -21.786000000

N -9.636000000 14.455000000 -21.276000000

H -10.171000000 14.803000000 -20.480000000

C -8.474000000 15.205000000 -21.731000000

C -8.221000000 16.412000000 -20.820000000

C -7.118000000 17.331000000 -21.327000000

C -6.586000000 18.273000000 -20.262000000

O -7.384000000 19.025000000 -19.657000000

O -5.359000000 18.261000000 -20.035000000

H -8.667000000 15.566000000 -22.741000000

H -7.939000000 16.046000000 -19.832000000

H -9.144000000 16.986000000 -20.732000000

H -6.294000000 16.716000000 -21.688000000

H -7.506000000 17.922000000 -22.157000000

N -6.362000000 14.589000000 -22.735000000

H -6.577000000 15.295000000 -23.439000000

C -5.117000000 13.848000000 -22.810000000

C -4.047000000 14.649000000 -22.083000000

O -3.991000000 15.874000000 -22.210000000

C -4.678000000 13.637000000 -24.256000000

C -3.285000000 13.061000000 -24.334000000

C -2.980000000 11.863000000 -23.704000000

C -2.267000000 13.732000000 -24.994000000

C -1.696000000 11.347000000 -23.725000000

C -0.975000000 13.224000000 -25.022000000

C -0.697000000 12.030000000 -24.382000000

O 0.585000000 11.521000000 -24.383000000

H 1.274000000 11.992000000 -24.836000000

H -5.240000000 12.881000000 -22.323000000

H -4.694000000 14.596000000 -24.774000000

H -5.374000000 12.955000000 -24.745000000

H -3.760000000 11.323000000 -23.187000000

H -2.482000000 14.665000000 -25.494000000

H -1.479000000 10.413000000 -23.228000000

H -0.192000000 13.758000000 -25.540000000

N -3.202000000 13.962000000 -21.321000000

H -3.300000000 12.949000000 -21.255000000

C -2.138000000 14.627000000 -20.578000000

C -0.785000000 14.060000000 -21.008000000

O -0.324000000 13.047000000 -20.490000000

C -2.346000000 14.435000000 -19.063000000

S -3.922000000 15.086000000 -18.375000000

H -2.162000000 15.693000000 -20.805000000

H -2.308000000 13.366000000 -18.854000000

H -1.519000000 14.916000000 -18.540000000

H -4.393000000 16.048000000 -19.173000000

N -0.155000000 14.722000000 -21.968000000

H -0.586000000 15.560000000 -22.358000000

C 1.138000000 14.285000000 -22.481000000

C 2.219000000 14.358000000 -21.410000000

O 3.280000000 13.738000000 -21.538000000

C 1.550000000 15.164000000 -23.659000000

C 1.827000000 16.483000000 -23.218000000

O 2.161000000 17.196000000 -24.174000000

H 1.051000000 13.254000000 -22.824000000

H 2.443000000 14.746000000 -24.124000000

H 0.741000000 15.190000000 -24.390000000

O 1.746000000 16.867000000 -22.043000000

N 1.940000000 15.127000000 -20.362000000

H 1.041000000 15.608000000 -20.319000000

C 2.889000000 15.295000000 -19.280000000

C 3.228000000 14.008000000 -18.568000000

O 4.370000000 13.798000000 -18.178000000

H 2.465000000 15.989000000 -18.554000000

H 3.806000000 15.726000000 -19.682000000

N 2.239000000 13.140000000 -18.398000000

H 1.306000000 13.360000000 -18.746000000

C 2.477000000 11.879000000 -17.720000000

C 1.959000000 11.933000000 -16.302000000

O 1.180000000 12.813000000 -15.966000000

H 1.966000000 11.081000000 -18.258000000

H 3.548000000 11.675000000 -17.705000000

N 2.386000000 11.001000000 -15.462000000

C 1.941000000 10.985000000 -14.075000000

C 2.933000000 11.709000000 -13.173000000

O 4.133000000 11.724000000 -13.433000000

C 1.763000000 9.545000000 -13.592000000

C 0.936000000 8.685000000 -14.530000000

C 0.578000000 7.333000000 -13.943000000

O 1.397000000 6.783000000 -13.176000000

O -0.520000000 6.819000000 -14.264000000

H 0.979000000 11.494000000 -14.013000000

H 2.749000000 9.092000000 -13.490000000

H 1.281000000 9.560000000 -12.614000000

H 1.506000000 8.525000000 -15.446000000

H 0.017000000 9.216000000 -14.776000000

H 3.035000000 10.286000000 -15.791000000

O 2.522000000 12.292000000 -12.160000000

N -2.589000000 11.876000000 -10.338000000

C -2.334000000 13.286000000 -10.589000000

C -1.522000000 13.372000000 -11.873000000

O -0.364000000 12.969000000 -11.928000000

C -1.585000000 13.910000000 -9.410000000

C -2.408000000 13.837000000 -8.116000000

C -1.596000000 14.305000000 -6.923000000

C -3.670000000 14.674000000 -8.285000000

H -3.283000000 13.804000000 -10.728000000

H -0.647000000 13.374000000 -9.263000000

H -1.368000000 14.954000000 -9.637000000

H -2.700000000 12.800000000 -7.950000000

H -0.702000000 13.689000000 -6.828000000

H -1.307000000 15.346000000 -7.067000000

H -2.196000000 14.217000000 -6.018000000

H -4.262000000 14.629000000 -7.371000000

H -4.256000000 14.283000000 -9.117000000

H -3.395000000 15.709000000 -8.489000000

H -2.236000000 11.178000000 -10.977000000

H -2.197000000 11.666000000 -9.431000000

N -2.154000000 13.882000000 -12.921000000

H -3.115000000 14.209000000 -12.820000000

C -1.496000000 13.980000000 -14.208000000

C -0.940000000 15.358000000 -14.520000000

O -1.238000000 16.336000000 -13.849000000

C -2.456000000 13.539000000 -15.313000000

C -3.008000000 12.130000000 -15.078000000

C -3.818000000 11.709000000 -16.275000000

C -1.871000000 11.156000000 -14.838000000

H -0.659000000 13.282000000 -14.203000000

H -3.290000000 14.240000000 -15.352000000

H -1.931000000 13.559000000 -16.268000000

H -3.654000000 12.144000000 -14.200000000

H -4.213000000 10.706000000 -16.112000000

H -4.644000000 12.406000000 -16.418000000

H -3.184000000 11.710000000 -17.162000000

H -2.184000000 10.153000000 -15.127000000

H -1.007000000 11.452000000 -15.433000000

H -1.603000000 11.163000000 -13.781000000

N -0.121000000 15.416000000 -15.555000000

C 0.499000000 16.654000000 -15.971000000

C 0.300000000 16.778000000 -17.475000000

O 0.169000000 15.771000000 -18.165000000

C 1.993000000 16.606000000 -15.643000000

C 2.355000000 16.316000000 -14.183000000

C 3.843000000 16.061000000 -14.056000000

C 1.931000000 17.481000000 -13.314000000

H 0.031000000 17.496000000 -15.461000000

H 2.444000000 15.827000000 -16.258000000

H 2.436000000 17.562000000 -15.922000000

H 1.818000000 15.424000000 -13.860000000

H 2.189000000 17.273000000 -12.276000000

H 0.854000000 17.624000000 -13.399000000

H 2.444000000 18.385000000 -13.642000000

H 4.089000000 15.856000000 -13.014000000

H 4.118000000 15.203000000 -14.670000000

H 4.392000000 16.940000000 -14.393000000

H 0.080000000 14.562000000 -16.076000000

O 0.270000000 17.898000000 -18.004000000

N -6.098000000 16.608000000 -11.595000000

C -6.614000000 15.507000000 -10.801000000

C -7.376000000 14.607000000 -11.784000000

O -8.081000000 15.109000000 -12.659000000

C -7.563000000 16.026000000 -9.695000000

C -6.779000000 16.942000000 -8.748000000

C -8.162000000 14.858000000 -8.916000000

C -7.584000000 17.492000000 -7.610000000

H -5.789000000 14.953000000 -10.353000000

H -8.369000000 16.597000000 -10.156000000

H -6.392000000 17.780000000 -9.328000000

H -5.937000000 16.382000000 -8.340000000

H -8.717000000 14.213000000 -9.597000000

H -7.362000000 14.287000000 -8.446000000

H -8.835000000 15.240000000 -8.148000000

H -7.179000000 18.459000000 -7.311000000

H -8.621000000 17.614000000 -7.924000000

H -7.539000000 16.803000000 -6.767000000

H -6.282000000 16.645000000 -12.587000000

H -5.096000000 16.631000000 -11.470000000

N -7.232000000 13.290000000 -11.662000000

H -6.640000000 12.908000000 -10.924000000

C -7.919000000 12.392000000 -12.581000000

C -8.383000000 11.096000000 -11.941000000

O -7.817000000 10.651000000 -10.947000000

C -7.017000000 12.057000000 -13.774000000

H -8.799000000 12.911000000 -12.962000000

H -6.284000000 12.854000000 -13.896000000

H -7.627000000 11.993000000 -14.675000000

N -9.409000000 10.496000000 -12.542000000

H -9.805000000 10.944000000 -13.369000000

C -10.005000000 9.238000000 -12.095000000

C -11.043000000 9.433000000 -10.996000000

O -10.729000000 9.410000000 -9.804000000

C -8.935000000 8.242000000 -11.633000000

C -9.461000000 6.806000000 -11.586000000

O -8.656000000 5.868000000 -11.368000000

O -10.685000000 6.617000000 -11.771000000

H -10.515000000 8.796000000 -12.951000000

H -8.094000000 8.285000000 -12.325000000

H -8.591000000 8.527000000 -10.639000000

N -12.293000000 9.620000000 -11.407000000

H -12.499000000 9.616000000 -12.406000000

C -13.367000000 9.829000000 -10.459000000

C -14.265000000 8.614000000 -10.320000000

O -15.444000000 8.727000000 -9.974000000

C -14.166000000 11.065000000 -10.864000000

C -13.355000000 12.320000000 -10.833000000

C -12.522000000 12.648000000 -11.889000000

C -13.352000000 13.128000000 -9.704000000

C -11.703000000 13.748000000 -11.816000000

C -12.526000000 14.234000000 -9.632000000

C -11.704000000 14.540000000 -10.684000000

H -12.919000000 10.026000000 -9.485000000

H -15.005000000 11.177000000 -10.177000000

H -14.553000000 10.920000000 -11.873000000

H -12.516000000 12.034000000 -12.777000000

H -14.001000000 12.890000000 -8.874000000

H -11.057000000 13.994000000 -12.645000000

H -12.529000000 14.855000000 -8.749000000

H -11.056000000 15.402000000 -10.628000000

N -13.682000000 7.446000000 -10.584000000

C -14.414000000 6.199000000 -10.466000000

C -14.750000000 5.891000000 -9.015000000

O -15.567000000 5.023000000 -8.743000000

H -15.340000000 6.275000000 -11.036000000

H -13.809000000 5.389000000 -10.873000000

H -12.704000000 7.428000000 -10.874000000

O -14.194000000 6.522000000 -8.106000000

H -13.763000000 7.011000000 -7.401000000

H -11.878000000 1.696000000 -21.488000000

H -7.052000000 4.171000000 -24.863000000

H 2.420000000 17.749000000 -24.915000000

H -7.996000000 19.601000000 -19.193000000

H -20.063000000 15.747000000 -14.050000000

H -15.270000000 4.657000000 -12.911000000

H -8.040000000 5.151000000 -11.201000000

H -1.360000000 6.426000000 -14.510000000

H 2.204000000 12.744000000 -11.375000000

H 0.247000000 18.766000000 -18.414000000

H -22.715000000 10.728000000 -14.879000000

H -14.438000000 16.485000000 -19.116000000

Azole **3** with surrounded residues

C -2.024000000 4.919000000 -22.123000000

C -3.016000000 6.041000000 -21.881000000

C -3.077000000 7.058000000 -23.034000000

C -4.328000000 7.939000000 -22.876000000

C -1.822000000 7.936000000 -23.035000000

H -1.065000000 5.312000000 -22.538000000

H -4.023000000 5.627000000 -21.659000000

H -2.725000000 6.567000000 -20.936000000

H -3.151000000 6.515000000 -24.003000000

H -1.884000000 8.736000000 -23.779000000

H -0.917000000 7.359000000 -23.244000000

H -1.666000000 8.409000000 -22.051000000

H -4.378000000 8.688000000 -23.670000000

H -5.236000000 7.331000000 -22.915000000

H -4.308000000 8.462000000 -21.909000000

C -7.118000000 3.704000000 -21.617000000

C -6.629000000 4.571000000 -20.438000000

C -6.799000000 6.050000000 -20.796000000

C -7.403000000 4.273000000 -19.151000000

H -8.187000000 3.909000000 -21.874000000

H -5.529000000 4.379000000 -20.273000000

H -7.127000000 3.325000000 -18.688000000

H -8.486000000 4.313000000 -19.302000000

H -7.175000000 5.051000000 -18.392000000

H -7.851000000 6.359000000 -20.761000000

H -6.428000000 6.264000000 -21.806000000

H -6.242000000 6.684000000 -20.093000000

C -7.645000000 9.791000000 -23.909000000

C -9.042000000 9.145000000 -23.917000000

O -10.025000000 9.834000000 -23.665000000

C -7.202000000 10.034000000 -22.485000000

H -6.882000000 9.170000000 -24.459000000

H -7.800000000 10.807000000 -21.968000000

H -7.297000000 9.122000000 -21.862000000

H -6.142000000 10.334000000 -22.405000000

N -9.116000000 7.867000000 -24.345000000

H -8.296000000 7.239000000 -24.351000000

C -10.398000000 7.145000000 -24.225000000

C -10.238000000 6.317000000 -22.931000000

O -9.108000000 6.029000000 -22.566000000

C -10.587000000 6.189000000 -25.431000000

C -11.920000000 5.453000000 -25.313000000

C -10.544000000 6.982000000 -26.742000000

H -11.250000000 7.863000000 -24.163000000

H -9.753000000 5.436000000 -25.429000000

H -10.709000000 6.319000000 -27.601000000

H -11.320000000 7.753000000 -26.772000000

H -9.577000000 7.469000000 -26.892000000

H -12.104000000 4.823000000 -26.191000000

H -11.958000000 4.765000000 -24.447000000

H -12.772000000 6.132000000 -25.217000000

N -11.351000000 6.016000000 -22.222000000

C -11.260000000 4.928000000 -21.227000000

C -11.705000000 5.459000000 -19.859000000

C -11.509000000 4.384000000 -18.785000000

C -11.543000000 5.011000000 -17.388000000

C -11.281000000 3.933000000 -16.334000000

H -10.228000000 4.515000000 -21.177000000

H -12.772000000 5.762000000 -19.903000000

H -11.128000000 6.371000000 -19.614000000

H -12.296000000 3.607000000 -18.902000000

H -10.553000000 3.853000000 -18.951000000

H -12.533000000 5.498000000 -17.212000000

H -10.795000000 5.828000000 -17.333000000

H -12.217000000 3.353000000 -16.137000000

H -10.507000000 3.213000000 -16.675000000

H -12.299000000 6.126000000 -22.607000000

C -18.323000000 8.203000000 -12.467000000

C -17.426000000 7.076000000 -12.973000000

C -16.233000000 7.669000000 -13.726000000

H -17.808000000 8.744000000 -11.624000000

H -17.003000000 6.474000000 -12.127000000

H -17.962000000 6.375000000 -13.635000000

H -15.739000000 8.455000000 -13.115000000

H -16.533000000 8.123000000 -14.680000000

C -17.522000000 14.057000000 -14.711000000

C -17.284000000 13.091000000 -15.862000000

C -16.101000000 12.159000000 -15.683000000

O -14.951000000 12.567000000 -15.656000000

N -16.372000000 10.816000000 -15.699000000

H -15.633000000 10.154000000 -15.516000000

H -17.297000000 10.465000000 -15.448000000

H -16.571000000 14.427000000 -14.262000000

H -18.214000000 12.547000000 -16.129000000

H -17.067000000 13.705000000 -16.780000000

C -8.837000000 16.237000000 -16.292000000

C -8.578000000 14.731000000 -16.334000000

C -9.209000000 14.134000000 -17.597000000

C -7.069000000 14.472000000 -16.362000000

H -8.070000000 16.793000000 -16.903000000

H -9.042000000 14.232000000 -15.450000000

H -8.974000000 13.067000000 -17.691000000

H -8.834000000 14.643000000 -18.495000000

H -10.303000000 14.252000000 -17.583000000

H -6.597000000 14.961000000 -17.236000000

H -6.846000000 13.404000000 -16.487000000

H -6.546000000 14.843000000 -15.481000000

C -11.295000000 12.685000000 -21.196000000

C -10.183000000 13.496000000 -21.869000000

O -9.783000000 13.150000000 -22.978000000

C -10.769000000 11.839000000 -20.042000000

C -11.927000000 11.191000000 -19.244000000

C -11.475000000 9.826000000 -18.728000000

C -12.355000000 12.113000000 -18.105000000

H -12.125000000 13.364000000 -20.846000000

H -10.139000000 12.452000000 -19.356000000

H -10.048000000 11.073000000 -20.396000000

H -12.814000000 11.028000000 -19.905000000

H -12.188000000 9.417000000 -17.995000000

H -11.370000000 9.088000000 -19.527000000

H -10.506000000 9.891000000 -18.210000000

H -13.172000000 11.677000000 -17.504000000

H -12.719000000 13.085000000 -18.452000000

H -11.536000000 12.302000000 -17.399000000

N -9.771000000 14.621000000 -21.240000000

H -10.077000000 14.891000000 -20.300000000

C -8.517000000 15.240000000 -21.709000000

C -7.438467000 14.173056000 -21.779653000

O -7.391377000 13.265242000 -20.901860000

C -8.030000000 16.295000000 -20.700000000

C -7.118000000 17.309000000 -21.368000000

H -8.698000000 15.703000000 -22.715000000

H -7.509000000 15.818000000 -19.846000000

H -8.887000000 16.853000000 -20.234000000

H -7.644000000 17.922000000 -22.112000000

H -6.235000000 16.858000000 -21.850000000

N -6.410000000 14.318000000 -22.626000000

H -6.181000000 15.287000000 -22.929000000

C -5.235000000 13.430000000 -22.609000000

C -4.064000000 14.363000000 -22.168000000

O -4.087000000 15.520000000 -22.540000000

C -4.969000000 12.887000000 -24.036000000

C -3.501000000 12.704000000 -24.212000000

C -2.832000000 11.565000000 -23.743000000

C -2.753000000 13.775000000 -24.735000000

C -1.461000000 11.547000000 -23.643000000

C -1.386000000 13.792000000 -24.639000000

C -0.705000000 12.724000000 -23.963000000

O 0.503000000 12.851000000 -23.625000000

H -5.381000000 12.593000000 -21.889000000

H -5.516000000 11.935000000 -24.189000000

H -5.353000000 13.608000000 -24.787000000

H -3.408000000 10.697000000 -23.427000000

H -3.275000000 14.624000000 -25.174000000

H -0.916000000 10.696000000 -23.254000000

H -0.794000000 14.623000000 -24.995000000

N -3.162000000 13.853000000 -21.284000000

C -2.141000000 14.734000000 -20.704000000

C -2.377000000 14.901000000 -19.204000000

S -4.112000000 15.399000000 -18.837000000

H -2.164000000 15.741000000 -21.224000000

H -2.193000000 13.952000000 -18.662000000

H -1.670000000 15.641000000 -18.776000000

H -4.224000000 16.616000000 -19.460000000

H -3.069000000 12.864000000 -21.104000000

C 1.885000000 10.957000000 -14.037000000

C 1.764000000 9.507000000 -13.600000000

C 0.896000000 8.717000000 -14.564000000

H 0.909000000 11.486000000 -13.979000000

H 2.753000000 9.016000000 -13.473000000

H 1.342000000 9.458000000 -12.570000000

H 1.388000000 8.499000000 -15.524000000

H -0.067000000 9.208000000 -14.768000000

C -1.502000000 13.912000000 -14.290000000

C -2.489000000 13.612000000 -15.402000000

C -3.034000000 12.173000000 -15.419000000

C -4.052000000 12.040000000 -16.557000000

C -1.916000000 11.147000000 -15.618000000

H -0.641000000 13.200000000 -14.294000000

H -3.319000000 14.342000000 -15.405000000

H -1.965000000 13.819000000 -16.374000000

H -3.559000000 11.957000000 -14.459000000

H -2.326000000 10.132000000 -15.722000000

H -1.346000000 11.350000000 -16.534000000

H -1.223000000 11.120000000 -14.772000000

H -4.565000000 11.070000000 -16.517000000

H -3.568000000 12.109000000 -17.541000000

H -4.816000000 12.819000000 -16.521000000

C -7.842000000 12.412000000 -12.730000000

C -8.296000000 11.090000000 -12.004000000

O -7.786000000 10.894000000 -10.920000000

C -6.745000000 12.071000000 -13.761000000

O -5.503792000 11.838071000 -13.141550000

H -5.569437000 10.981192000 -12.643006000

H -8.716000000 12.916000000 -13.196000000

H -6.952000000 11.098000000 -14.277000000

H -6.611000000 12.859000000 -14.521000000

N -9.109000000 10.331000000 -12.766000000

H -9.530000000 10.724000000 -13.603000000

C -9.857000000 9.202000000 -12.173000000

C -10.924000000 9.876000000 -11.271000000

O -10.675000000 10.999000000 -10.840000000

C -8.936000000 8.246000000 -11.452000000

H -10.372000000 8.661000000 -13.023000000

H -7.904000000 8.280000000 -11.846000000

H -8.843000000 8.476000000 -10.374000000

N -12.114000000 9.250000000 -11.200000000

C -13.267000000 9.823000000 -10.490000000

C -13.978000000 10.935000000 -11.285000000

C -13.169000000 12.164000000 -11.564000000

C -13.199000000 12.752000000 -12.831000000

C -12.402000000 12.772000000 -10.559000000

C -12.464000000 13.906000000 -13.101000000

C -11.670000000 13.924000000 -10.827000000

C -11.694000000 14.495000000 -12.102000000

H -12.967000000 10.191000000 -9.484000000

H -14.378000000 10.497000000 -12.226000000

H -14.891000000 11.212000000 -10.708000000

H -13.809000000 12.319000000 -13.627000000

H -12.350000000 12.315000000 -9.571000000

H -12.514000000 14.356000000 -14.093000000

H -11.082000000 14.379000000 -10.039000000

H -11.149000000 15.407000000 -12.300000000

H -12.174000000 8.194000000 -11.369000000

C -5.923348000 9.029264000 -17.837621000

C -4.565551000 8.900789000 -18.259307000

C -4.462855000 9.829336000 -19.320809000

N -5.708371000 10.391894000 -19.455492000

N -6.586929000 9.922945000 -18.544519000

C -3.237199000 10.042899000 -19.969718000

C -2.141317000 9.331039000 -19.525697000

C -2.247660000 8.383614000 -18.477322000

C -3.452044000 8.163942000 -17.828365000

H -6.015515000 11.156608000 -20.037852000

S -6.601613000 8.290085000 -16.369871000

O -6.634183000 6.828332000 -16.546923000

O -5.830400000 8.841736000 -15.242809000

C -8.287165000 8.888433000 -16.266401000

C -8.590997000 10.248543000 -16.248512000

C -9.888362000 10.637792000 -15.927986000

C -10.877139000 9.693200000 -15.594160000

C -10.555398000 8.331543000 -15.688229000

C -12.244988000 10.127937000 -15.125514000

C -9.265223000 7.929321000 -16.022428000

N -1.089818000 7.643330000 -18.127919000

C -1.024216000 6.431795000 -17.498101000

C 0.308788000 6.095240000 -17.415447000

C 0.984028000 7.171214000 -18.035373000

N 0.143039000 8.105194000 -18.466731000

H -3.140784000 10.705090000 -20.817837000

H -1.167983000 9.474369000 -19.976467000

H -3.540550000 7.474396000 -16.998229000

H -7.836659000 10.991032000 -16.473547000

H -10.103202000 11.697652000 -15.839450000

H -11.311094000 7.583764000 -15.464155000

H -8.976615000 6.889525000 -16.085988000

H -1.911968000 5.902435000 -17.189802000

H 0.729532000 5.201996000 -16.979630000

H 2.047584000 7.306161000 -18.178838000

H -12.356926000 11.211132000 -15.191390000

H -13.043250000 9.664094000 -15.713295000

H -12.422168000 9.838645000 -14.083395000

N -2.564000000 3.856000000 -23.064000000

C -1.721000000 4.207000000 -20.769000000

O -0.655000000 4.475000000 -20.213000000

H -1.950000000 3.731000000 -23.863000000

H -2.684000000 2.946000000 -22.563000000

O -2.504000000 3.408000000 -20.237000000

N -6.914000000 2.232000000 -21.285000000

C -6.212000000 3.896000000 -22.850000000

O -4.998000000 3.971000000 -22.680000000

H -7.228000000 2.050000000 -20.312000000

H -7.412000000 1.580000000 -21.914000000

O -6.685000000 4.051000000 -23.984000000

N -7.810000000 11.109000000 -24.650000000

H -6.925000000 11.669000000 -24.650000000

H -8.118000000 10.962000000 -25.620000000

C -12.247000000 3.846000000 -21.763000000

O -13.295000000 4.220000000 -22.292000000

N -10.825000000 4.567000000 -15.042000000

H -10.614000000 3.860000000 -14.312000000

H -9.983000000 5.155000000 -15.157000000

O -12.039000000 2.635000000 -21.608000000

N -19.659000000 7.628000000 -11.972000000

C -18.674000000 9.165000000 -13.615000000

O -17.964000000 10.150000000 -13.771000000

C -15.176000000 6.582000000 -13.982000000

O -14.245000000 6.852000000 -14.766000000

O -15.229000000 5.495000000 -13.377000000

O -19.607000000 8.949000000 -14.400000000

H -20.125000000 8.238000000 -11.291000000

H -19.490000000 6.707000000 -11.529000000

N -18.408000000 13.436000000 -13.639000000

C -18.350000000 15.230000000 -15.328000000

O -17.787000000 16.280000000 -15.632000000

H -19.418000000 13.613000000 -13.873000000

H -18.229000000 13.768000000 -12.685000000

O -19.552000000 15.081000000 -15.588000000

N -8.842000000 16.763000000 -14.873000000

C -10.236000000 16.661000000 -16.839000000

O -11.274000000 16.583000000 -16.184000000

H -8.264000000 16.197000000 -14.250000000

H -8.485000000 17.738000000 -14.865000000

O -10.277000000 17.078000000 -18.005000000

N -11.852000000 11.728000000 -22.235000000

H -12.592000000 11.136000000 -21.772000000

H -12.264000000 12.202000000 -23.051000000

C -6.586000000 18.273000000 -20.262000000

O -7.384000000 19.025000000 -19.657000000

O -5.359000000 18.261000000 -20.035000000

C -0.785000000 14.060000000 -21.008000000

O -0.324000000 13.047000000 -20.490000000

O -0.141000000 14.634000000 -21.897000000

N 2.386000000 11.001000000 -15.462000000

C 2.933000000 11.709000000 -13.173000000

O 4.133000000 11.724000000 -13.433000000

C 0.578000000 7.333000000 -13.943000000

O 1.397000000 6.783000000 -13.176000000

O -0.520000000 6.819000000 -14.264000000

O 2.522000000 12.292000000 -12.160000000

H 1.922000000 10.271000000 -16.034000000

H 2.214000000 11.925000000 -15.898000000

N -2.154000000 13.882000000 -12.921000000

C -0.940000000 15.358000000 -14.520000000

O -1.238000000 16.336000000 -13.849000000

O -0.166000000 15.495000000 -15.477000000

H -1.547000000 13.400000000 -12.230000000

H -3.055000000 13.393000000 -12.956000000

N -7.232000000 13.290000000 -11.662000000

H -6.296000000 13.566000000 -12.000000000

H -7.780000000 14.132000000 -11.444000000

C -9.461000000 6.806000000 -11.586000000

O -8.656000000 5.868000000 -11.368000000

O -10.685000000 6.617000000 -11.771000000

C -14.265000000 8.614000000 -10.320000000

O -15.444000000 8.727000000 -9.974000000

O -13.806000000 7.488000000 -10.558000000

H 2.204000000 12.744000000 -11.375000000

H 2.026000000 6.360000000 -12.587000000

H 0.434000000 15.601000000 -16.219000000

H -7.996000000 19.601000000 -19.193000000

H -12.084000000 16.522000000 -15.673000000

H -15.270000000 4.657000000 -12.911000000

H -11.878000000 1.696000000 -21.488000000

H -7.052000000 4.171000000 -24.863000000

H -20.330000000 8.782000000 -15.009000000

H -14.131000000 6.598000000 -10.402000000

H -8.040000000 5.151000000 -11.201000000

H -0.147000000 15.589000000 -21.993000000

H 1.422000000 12.949000000 -23.368000000

H -3.111000000 2.789000000 -19.825000000

H -20.207000000 15.417000000 -16.204000000

Azole **4** with surrounded residues

C -2.897485000 8.452322000 -17.942773000

C -4.033749000 9.087905000 -18.472610000

C -1.674943000 8.689280000 -18.556720000

C -3.917863000 9.995540000 -19.546265000

C -1.574521000 9.593644000 -19.647493000

C -2.676072000 10.254526000 -20.144589000

C -5.434594000 9.101546000 -18.173467000

N -6.108861000 9.922513000 -18.957855000

N -5.184184000 10.462800000 -19.785700000

H -5.466246000 11.201080000 -20.408936000

S -6.183008000 8.048951000 -16.937792000

O -6.330210000 6.699518000 -17.507496000

O -5.341350000 8.214237000 -15.738169000

C -7.835589000 8.677073000 -16.604628000

C -8.142345000 10.035689000 -16.527115000

C -9.419026000 10.420723000 -16.117595000

C -10.387445000 9.469814000 -15.764973000

C -11.789558000 9.865283000 -15.369478000

C -10.047394000 8.115059000 -15.848779000

C -8.787794000 7.709600000 -16.278633000

N -0.483452000 8.041840000 -18.145455000

N 0.714817000 8.616681000 -18.473089000

C 1.642317000 7.747075000 -18.092656000

C 3.096584000 8.058479000 -18.266876000

C 1.049988000 6.592972000 -17.521588000

C -0.311746000 6.801851000 -17.569393000

C -1.413309000 5.881614000 -17.160282000

H -3.000176000 7.828659000 -17.068798000

H -2.563720000 10.909711000 -20.994013000

H -0.596901000 9.750899000 -20.083133000

H 1.546259000 5.719368000 -17.124119000

H -8.528752000 6.660985000 -16.366940000

H -10.793955000 7.375583000 -15.577685000

H -9.667917000 11.477477000 -16.073528000

H -7.404217000 10.781367000 -16.796710000

H -0.978232000 4.911277000 -16.915126000

H -1.963552000 6.223201000 -16.279070000

H -2.148655000 5.755749000 -17.960869000

H 3.619538000 7.238975000 -18.771073000

H 3.218937000 8.966053000 -18.861251000

H 3.588987000 8.212927000 -17.300049000

H -11.830365000 10.867518000 -14.935871000

H -12.212412000 9.155603000 -14.655055000

H -12.458613000 9.852623000 -16.235803000

C -7.256211000 14.364764000 -21.750425000

O -7.037993000 13.561191000 -20.855822000

O -6.307379000 10.861039000 -13.576426000

H -5.909640000 10.624640000 -14.452588000

N -2.564000000 3.856000000 -23.064000000

C -2.089000000 4.847000000 -22.104000000

C -1.721000000 4.207000000 -20.769000000

O -0.655000000 4.475000000 -20.213000000

C -3.161000000 5.910000000 -21.873000000

C -3.399000000 6.915000000 -22.996000000

C -4.670000000 7.698000000 -22.725000000

C -2.199000000 7.842000000 -23.096000000

H -1.202000000 5.331000000 -22.514000000

H -4.103000000 5.392000000 -21.692000000

H -2.900000000 6.464000000 -20.972000000

H -3.509000000 6.375000000 -23.937000000

H -4.583000000 8.694000000 -23.158000000

H -5.518000000 7.180000000 -23.173000000

H -4.823000000 7.782000000 -21.649000000

H -2.362000000 8.563000000 -23.897000000

H -2.069000000 8.371000000 -22.152000000

H -1.305000000 7.257000000 -23.311000000

H -2.020000000 3.018000000 -22.917000000

H -2.356000000 4.200000000 -23.991000000

N -2.611000000 3.366000000 -20.258000000

H -3.480000000 3.182000000 -20.760000000

C -2.358000000 2.706000000 -18.992000000

C -3.507000000 1.800000000 -18.602000000

O -4.587000000 2.270000000 -18.248000000

H -1.450000000 2.110000000 -19.078000000

H -2.218000000 3.460000000 -18.217000000

O -3.357000000 0.571000000 -18.640000000

N -6.914000000 2.232000000 -21.285000000

H -5.958000000 1.916000000 -21.121000000

C -7.141000000 3.614000000 -21.675000000

C -6.212000000 3.896000000 -22.850000000

O -4.998000000 3.971000000 -22.680000000

C -6.812000000 4.580000000 -20.522000000

C -7.120000000 6.006000000 -20.938000000

C -7.614000000 4.206000000 -19.290000000

H -8.178000000 3.746000000 -21.983000000

H -5.750000000 4.502000000 -20.289000000

H -8.115000000 6.280000000 -20.588000000

H -6.383000000 6.679000000 -20.500000000

H -7.083000000 6.084000000 -22.025000000

H -8.092000000 5.097000000 -18.882000000

H -6.949000000 3.776000000 -18.541000000

H -8.377000000 3.476000000 -19.561000000

H -7.296000000 1.651000000 -22.017000000

O -6.685000000 4.051000000 -23.984000000

N -7.810000000 11.109000000 -24.650000000

H -8.419000000 11.844000000 -24.292000000

C -7.667000000 9.874000000 -23.900000000

C -9.024000000 9.198000000 -23.777000000

O -9.997000000 9.801000000 -23.325000000

C -7.092000000 10.166000000 -22.525000000

H -6.987000000 9.211000000 -24.435000000

H -7.645000000 10.985000000 -22.066000000

H -7.175000000 9.277000000 -21.900000000

H -6.043000000 10.445000000 -22.622000000

H -6.888000000 11.505000000 -24.769000000

N -9.086000000 7.939000000 -24.187000000

H -8.246000000 7.484000000 -24.546000000

C -10.334000000 7.200000000 -24.132000000

C -10.241000000 5.992000000 -23.215000000

O -9.362000000 5.136000000 -23.368000000

C -10.757000000 6.739000000 -25.535000000

C -12.086000000 6.006000000 -25.467000000

C -10.854000000 7.941000000 -26.455000000

H -11.107000000 7.864000000 -23.744000000

H -10.000000000 6.058000000 -25.924000000

H -12.376000000 5.684000000 -26.467000000

H -12.849000000 6.673000000 -25.065000000

H -11.988000000 5.135000000 -24.819000000

H -11.154000000 7.614000000 -27.451000000

H -9.883000000 8.434000000 -26.512000000

H -11.594000000 8.639000000 -26.064000000

N -11.160000000 5.939000000 -22.257000000

H -11.852000000 6.686000000 -22.193000000

C -11.208000000 4.850000000 -21.299000000

C -12.247000000 3.846000000 -21.763000000

O -13.295000000 4.220000000 -22.292000000

C -11.569000000 5.384000000 -19.908000000

C -11.561000000 4.333000000 -18.800000000

C -11.519000000 4.986000000 -17.417000000

C -11.276000000 3.955000000 -16.326000000

N -10.825000000 4.567000000 -15.042000000

H -10.662000000 3.875000000 -14.310000000

H -10.000000000 5.149000000 -15.185000000

H -10.233000000 4.365000000 -21.256000000

H -10.850000000 6.160000000 -19.645000000

H -12.562000000 5.832000000 -19.955000000

H -12.463000000 3.726000000 -18.879000000

H -10.687000000 3.693000000 -18.920000000

H -10.715000000 5.721000000 -17.396000000

H -12.468000000 5.488000000 -17.230000000

H -10.509000000 3.260000000 -16.668000000

H -12.199000000 3.403000000 -16.149000000

O -12.039000000 2.635000000 -21.608000000

N -19.659000000 7.628000000 -11.972000000

C -18.418000000 8.207000000 -12.466000000

C -18.674000000 9.165000000 -13.615000000

O -17.964000000 10.150000000 -13.771000000

C -17.459000000 7.107000000 -12.934000000

C -16.274000000 7.626000000 -13.753000000

C -15.176000000 6.582000000 -13.982000000

O -14.245000000 6.852000000 -14.766000000

O -15.229000000 5.495000000 -13.377000000

H -17.944000000 8.758000000 -11.654000000

H -17.070000000 6.594000000 -12.055000000

H -18.016000000 6.391000000 -13.539000000

H -15.837000000 8.474000000 -13.226000000

H -16.640000000 7.967000000 -14.721000000

H -19.798000000 7.980000000 -11.036000000

H -20.413000000 7.975000000 -12.548000000

N -19.695000000 8.880000000 -14.415000000

H -20.267000000 8.057000000 -14.226000000

C -20.010000000 9.725000000 -15.559000000

C -20.763000000 10.996000000 -15.182000000

O -20.455000000 12.080000000 -15.689000000

C -20.822000000 8.943000000 -16.620000000

C -19.966000000 7.816000000 -17.197000000

C -21.253000000 9.870000000 -17.747000000

C -20.730000000 6.885000000 -18.097000000

H -19.068000000 10.023000000 -16.018000000

H -21.707000000 8.515000000 -16.150000000

H -19.555000000 7.237000000 -16.370000000

H -19.143000000 8.254000000 -17.762000000

H -21.864000000 10.676000000 -17.341000000

H -20.370000000 10.291000000 -18.229000000

H -21.833000000 9.307000000 -18.479000000

H -20.061000000 6.110000000 -18.471000000

H -21.543000000 6.424000000 -17.536000000

H -21.140000000 7.446000000 -18.936000000

N -21.749000000 10.869000000 -14.299000000

H -21.968000000 9.950000000 -13.914000000

C -22.518000000 12.031000000 -13.878000000

C -21.650000000 12.983000000 -13.077000000

O -21.880000000 14.191000000 -13.080000000

C -23.743000000 11.611000000 -13.061000000

S -25.103000000 11.007000000 -14.097000000

H -22.865000000 12.553000000 -14.770000000

H -24.094000000 12.472000000 -12.492000000

H -23.451000000 10.823000000 -12.366000000

H -25.072000000 9.672000000 -14.141000000

N -20.645000000 12.444000000 -12.393000000

H -20.503000000 11.434000000 -12.409000000

C -19.746000000 13.290000000 -11.621000000

C -18.885000000 14.091000000 -12.588000000

O -18.670000000 15.284000000 -12.396000000

C -18.839000000 12.460000000 -10.681000000

C -19.665000000 11.940000000 -9.504000000

C -17.676000000 13.309000000 -10.172000000

C -18.835000000 11.380000000 -8.374000000

H -20.339000000 13.980000000 -11.021000000

H -18.440000000 11.610000000 -11.235000000

H -20.264000000 12.764000000 -9.115000000

H -20.336000000 11.160000000 -9.865000000

H -17.348000000 13.986000000 -10.960000000

H -16.850000000 12.658000000 -9.885000000

H -18.000000000 13.888000000 -9.307000000

H -19.432000000 10.670000000 -7.801000000

H -18.513000000 12.193000000 -7.723000000

H -17.960000000 10.873000000 -8.782000000

N -18.408000000 13.436000000 -13.639000000

H -18.623000000 12.445000000 -13.752000000

C -17.583000000 14.107000000 -14.633000000

C -18.350000000 15.230000000 -15.328000000

O -17.787000000 16.280000000 -15.632000000

C -17.068000000 13.094000000 -15.661000000

C -15.763000000 12.428000000 -15.228000000

O -14.713000000 13.071000000 -15.171000000

N -15.826000000 11.140000000 -14.919000000

H -14.955000000 10.695000000 -14.630000000

H -16.695000000 10.608000000 -14.966000000

H -16.724000000 14.544000000 -14.125000000

H -17.825000000 12.322000000 -15.799000000

H -16.906000000 13.605000000 -16.610000000

O -19.552000000 15.081000000 -15.588000000

N -8.842000000 16.763000000 -14.873000000

C -8.904000000 16.251000000 -16.233000000

C -10.236000000 16.661000000 -16.839000000

O -11.274000000 16.583000000 -16.184000000

C -8.757000000 14.730000000 -16.248000000

C -9.094000000 14.181000000 -17.629000000

C -7.333000000 14.369000000 -15.868000000

H -8.096000000 16.693000000 -16.817000000

H -9.441000000 14.299000000 -15.517000000

H -8.581000000 13.231000000 -17.778000000

H -10.171000000 14.029000000 -17.706000000

H -8.772000000 14.891000000 -18.391000000

H -6.638000000 15.006000000 -16.415000000

H -7.144000000 13.325000000 -16.119000000

H -7.195000000 14.516000000 -14.797000000

O -10.277000000 17.078000000 -18.005000000

H -9.780000000 17.039000000 -14.622000000

H -8.271000000 17.596000000 -14.886000000

N -15.343000000 6.524000000 -22.559000000

H -14.625000000 5.821000000 -22.383000000

C -15.276000000 7.803000000 -21.865000000

C -14.182000000 8.625000000 -22.512000000

O -13.046000000 8.168000000 -22.634000000

C -14.959000000 7.609000000 -20.381000000

C -16.112000000 7.393000000 -19.396000000

C -16.908000000 6.156000000 -19.755000000

C -15.539000000 7.261000000 -17.996000000

H -16.229000000 8.322000000 -21.968000000

H -14.307000000 6.739000000 -20.305000000

H -14.393000000 8.478000000 -20.045000000

H -16.772000000 8.260000000 -19.428000000

H -16.350000000 7.107000000 -17.284000000

H -14.998000000 8.171000000 -17.738000000

H -14.858000000 6.411000000 -17.961000000

H -17.313000000 6.263000000 -20.761000000

H -17.726000000 6.032000000 -19.045000000

H -16.258000000 5.282000000 -19.717000000

H -16.239000000 6.107000000 -22.349000000

N -14.529000000 9.839000000 -22.923000000

H -15.487000000 10.158000000 -22.777000000

C -13.579000000 10.727000000 -23.576000000

C -13.120000000 11.815000000 -22.625000000

O -13.885000000 12.710000000 -22.267000000

C -14.217000000 11.331000000 -24.828000000

C -14.539000000 10.312000000 -25.883000000

C -15.367000000 9.237000000 -25.594000000

C -13.993000000 10.411000000 -27.153000000

C -15.645000000 8.276000000 -26.546000000

C -14.267000000 9.449000000 -28.119000000

C -15.095000000 8.379000000 -27.812000000

H -12.710000000 10.142000000 -23.878000000

H -15.141000000 11.832000000 -24.538000000

H -13.534000000 12.069000000 -25.249000000

H -15.801000000 9.150000000 -24.609000000

H -13.349000000 11.243000000 -27.395000000

H -16.291000000 7.445000000 -26.303000000

H -13.836000000 9.535000000 -29.106000000

H -15.310000000 7.629000000 -28.559000000

N -11.852000000 11.728000000 -22.235000000

H -11.275000000 10.976000000 -22.613000000

C -11.252000000 12.660000000 -21.292000000

C -10.018000000 13.331000000 -21.868000000

O -9.420000000 12.837000000 -22.819000000

C -10.880000000 11.902000000 -20.021000000

C -12.053000000 11.078000000 -19.490000000

C -11.581000000 9.694000000 -19.097000000

C -12.706000000 11.813000000 -18.329000000

H -11.985000000 13.427000000 -21.042000000

H -10.048000000 11.232000000 -20.240000000

H -10.571000000 12.616000000 -19.257000000

H -12.789000000 10.975000000 -20.288000000

H -12.424000000 9.115000000 -18.720000000

H -11.158000000 9.194000000 -19.968000000

H -10.821000000 9.776000000 -18.320000000

H -13.542000000 11.225000000 -17.951000000

H -11.975000000 11.958000000 -17.534000000

H -13.068000000 12.783000000 -18.671000000

N -9.636000000 14.455000000 -21.276000000

H -10.171000000 14.803000000 -20.480000000

C -8.474000000 15.205000000 -21.731000000

C -8.221000000 16.412000000 -20.820000000

C -7.118000000 17.331000000 -21.327000000

C -6.586000000 18.273000000 -20.262000000

O -7.384000000 19.025000000 -19.657000000

O -5.359000000 18.261000000 -20.035000000

H -8.667000000 15.566000000 -22.741000000

H -9.143000000 16.988000000 -20.745000000

H -7.950000000 16.052000000 -19.827000000

H -6.294000000 16.716000000 -21.688000000

H -7.506000000 17.922000000 -22.157000000

N -6.362000000 14.589000000 -22.735000000

H -6.577000000 15.295000000 -23.439000000

C -5.117000000 13.848000000 -22.810000000

C -4.047000000 14.649000000 -22.083000000

O -3.991000000 15.874000000 -22.210000000

C -4.678000000 13.637000000 -24.256000000

C -3.285000000 13.061000000 -24.334000000

C -2.980000000 11.863000000 -23.704000000

C -2.267000000 13.732000000 -24.994000000

C -1.696000000 11.347000000 -23.725000000

C -0.975000000 13.224000000 -25.022000000

C -0.697000000 12.030000000 -24.382000000

O 0.585000000 11.521000000 -24.383000000

H 1.274000000 11.992000000 -24.836000000

H -5.240000000 12.881000000 -22.323000000

H -5.372000000 12.950000000 -24.740000000

H -4.699000000 14.593000000 -24.779000000

H -3.760000000 11.323000000 -23.187000000

H -2.482000000 14.665000000 -25.494000000

H -1.479000000 10.413000000 -23.228000000

H -0.192000000 13.758000000 -25.540000000

N -3.202000000 13.962000000 -21.321000000

H -3.300000000 12.949000000 -21.255000000

C -2.138000000 14.627000000 -20.578000000

C -0.785000000 14.060000000 -21.008000000

O -0.324000000 13.047000000 -20.490000000

C -2.346000000 14.435000000 -19.063000000

S -3.922000000 15.086000000 -18.375000000

H -2.162000000 15.693000000 -20.805000000

H -1.526000000 14.935000000 -18.548000000

H -2.290000000 13.369000000 -18.842000000

H -4.393000000 16.048000000 -19.173000000

N -0.155000000 14.722000000 -21.968000000

H -0.586000000 15.560000000 -22.358000000

C 1.138000000 14.285000000 -22.481000000

C 2.219000000 14.358000000 -21.410000000

O 3.280000000 13.738000000 -21.538000000

C 1.550000000 15.164000000 -23.659000000

O 1.827000000 16.483000000 -23.218000000

H 2.083000000 17.030000000 -23.951000000

H 1.051000000 13.254000000 -22.824000000

H 2.443000000 14.746000000 -24.124000000

H 0.741000000 15.190000000 -24.390000000

N 1.940000000 15.127000000 -20.362000000

H 1.041000000 15.608000000 -20.319000000

C 2.889000000 15.295000000 -19.280000000

C 3.228000000 14.008000000 -18.568000000

O 4.370000000 13.798000000 -18.178000000

H 2.465000000 15.989000000 -18.554000000

H 3.806000000 15.726000000 -19.682000000

N 2.239000000 13.140000000 -18.398000000

H 1.306000000 13.360000000 -18.746000000

C 2.477000000 11.879000000 -17.720000000

C 1.959000000 11.933000000 -16.302000000

O 1.180000000 12.813000000 -15.966000000

H 1.966000000 11.081000000 -18.258000000

H 3.548000000 11.675000000 -17.705000000

N 2.386000000 11.001000000 -15.462000000

H 3.035000000 10.286000000 -15.791000000

C 1.941000000 10.985000000 -14.075000000

C 2.933000000 11.709000000 -13.173000000

O 4.133000000 11.724000000 -13.433000000

C 1.763000000 9.545000000 -13.592000000

C 0.936000000 8.685000000 -14.530000000

C 0.578000000 7.333000000 -13.943000000

O 1.397000000 6.783000000 -13.176000000

O -0.520000000 6.819000000 -14.264000000

H 0.979000000 11.494000000 -14.013000000

H 1.270000000 9.567000000 -12.620000000

H 2.747000000 9.090000000 -13.476000000

H 0.013000000 9.216000000 -14.764000000

H 1.496000000 8.531000000 -15.452000000

O 2.522000000 12.292000000 -12.160000000

N -2.154000000 13.882000000 -12.921000000

H -3.115000000 14.209000000 -12.820000000

C -1.496000000 13.980000000 -14.208000000

C -0.940000000 15.358000000 -14.520000000

O -1.238000000 16.336000000 -13.849000000

C -2.456000000 13.539000000 -15.313000000

C -3.008000000 12.130000000 -15.078000000

C -3.818000000 11.709000000 -16.275000000

C -1.871000000 11.156000000 -14.838000000

H -0.659000000 13.282000000 -14.203000000

H -3.290000000 14.240000000 -15.352000000

H -1.931000000 13.559000000 -16.268000000

H -3.654000000 12.144000000 -14.200000000

H -2.184000000 10.153000000 -15.127000000

H -1.604000000 11.163000000 -13.781000000

H -1.007000000 11.452000000 -15.433000000

H -4.213000000 10.706000000 -16.112000000

H -3.184000000 11.710000000 -17.161000000

H -4.644000000 12.406000000 -16.418000000

H -1.595000000 14.408000000 -12.265000000

O -0.166000000 15.495000000 -15.477000000

N -7.232000000 13.290000000 -11.662000000

C -7.919000000 12.392000000 -12.581000000

C -8.383000000 11.096000000 -11.941000000

O -7.817000000 10.651000000 -10.947000000

C -7.017000000 12.057000000 -13.774000000

H -8.799000000 12.911000000 -12.962000000

H -6.284000000 12.854000000 -13.896000000

H -7.627000000 11.993000000 -14.675000000

H -6.646000000 13.891000000 -12.224000000

H -7.932000000 13.871000000 -11.222000000

N -9.409000000 10.496000000 -12.542000000

H -9.805000000 10.944000000 -13.369000000

C -10.005000000 9.238000000 -12.095000000

C -11.043000000 9.433000000 -10.996000000

O -10.729000000 9.410000000 -9.804000000

C -8.935000000 8.242000000 -11.633000000

C -9.461000000 6.806000000 -11.586000000

O -8.656000000 5.868000000 -11.368000000

O -10.685000000 6.617000000 -11.771000000

H -10.515000000 8.796000000 -12.951000000

H -8.600000000 8.525000000 -10.635000000

H -8.088000000 8.288000000 -12.318000000

N -12.293000000 9.620000000 -11.407000000

H -12.499000000 9.616000000 -12.406000000

C -13.367000000 9.829000000 -10.459000000

C -14.265000000 8.614000000 -10.320000000

O -15.444000000 8.727000000 -9.974000000

C -14.166000000 11.065000000 -10.864000000

C -13.355000000 12.320000000 -10.833000000

C -12.522000000 12.648000000 -11.889000000

C -13.352000000 13.128000000 -9.704000000

C -11.703000000 13.748000000 -11.816000000

C -12.526000000 14.234000000 -9.632000000

C -11.704000000 14.540000000 -10.684000000

H -12.919000000 10.026000000 -9.485000000

H -15.005000000 11.177000000 -10.177000000

H -14.553000000 10.920000000 -11.873000000

H -12.516000000 12.034000000 -12.777000000

H -14.001000000 12.890000000 -8.874000000

H -11.057000000 13.994000000 -12.645000000

H -12.529000000 14.855000000 -8.749000000

H -11.056000000 15.402000000 -10.628000000

N -13.682000000 7.446000000 -10.584000000

H -12.704000000 7.428000000 -10.874000000

C -14.414000000 6.199000000 -10.466000000

C -14.750000000 5.891000000 -9.015000000

O -15.567000000 5.023000000 -8.743000000

H -13.804000000 5.390000000 -10.869000000

H -15.338000000 6.271000000 -11.040000000

O -14.194000000 6.522000000 -8.106000000

H -3.241000000 -0.381000000 -18.669000000

H -11.878000000 1.696000000 -21.488000000

H 2.026000000 6.360000000 -12.587000000

H 2.204000000 12.744000000 -11.375000000

H 0.434000000 15.601000000 -16.219000000

H -4.415000000 18.252000000 -19.860000000

H -20.483000000 14.966000000 -15.789000000

H -13.763000000 7.011000000 -7.401000000

H -8.040000000 5.151000000 -11.201000000

H -7.052000000 4.171000000 -24.863000000

H -10.309000000 17.401000000 -18.908000000

H -15.270000000 4.657000000 -12.911000000

Azole **5** with surrounded residues

C -5.044000000 4.881000000 -14.144000000

C -4.111000000 5.393000000 -15.079000000

C -3.182000000 4.351000000 -15.254000000

N -3.611000000 3.343000000 -14.424000000

N -4.748000000 3.660000000 -13.753000000

C -2.099000000 4.482000000 -16.139000000

C -1.995000000 5.675000000 -16.840000000

C -2.948000000 6.716000000 -16.668000000

C -4.000000000 6.582000000 -15.791000000

H -3.199000000 2.434000000 -14.281000000

S -6.485000000 5.785000000 -13.608000000

O -7.557000000 4.807000000 -13.351000000

O -6.072000000 6.744000000 -12.579000000

C -6.809000000 6.721000000 -15.106000000

C -7.553000000 6.265000000 -16.198000000

C -7.695000000 7.123000000 -17.303000000

C -7.100000000 8.398000000 -17.325000000

C -7.240000000 9.335000000 -18.500000000

C -6.377000000 8.810000000 -16.207000000

C -6.239000000 7.982000000 -15.108000000

N -2.861000000 7.914000000 -17.404000000

C -1.890000000 8.899000000 -17.271000000

C -2.147000000 9.922000000 -18.137000000

C -3.348000000 9.584000000 -18.846000000

C -4.114000000 10.230000000 -19.821000000

C -3.771000000 8.321000000 -18.371000000

C -5.279000000 9.625000000 -20.279000000

C -5.691000000 8.380000000 -19.776000000

C -4.947000000 7.709000000 -18.818000000

H -1.386000000 3.681000000 -16.281000000

H -1.175000000 5.818000000 -17.528000000

H -4.731000000 7.369000000 -15.679000000

H -1.059000000 8.854000000 -16.587000000

H -1.562000000 10.820000000 -18.262000000

H -3.798000000 11.186000000 -20.200000000

H -5.881000000 10.111000000 -21.029000000

H -6.476000000 7.718000000 -19.928000000

H -5.269000000 6.754000000 -18.429000000

H -8.001000000 5.281000000 -16.197000000

H -8.262000000 6.800000000 -18.162000000

H -5.911000000 9.786000000 -16.201000000

H -5.676000000 8.289000000 -14.251000000

H -8.172000000 9.873000000 -18.435000000

H -6.418000000 10.051000000 -18.491000000

H -7.345000000 9.034000000 -19.521000000

O -6.226000000 2.388000000 -16.287000000

H -5.561000000 2.275000000 -15.539000000

N -10.901000000 4.594000000 -15.067000000

H -10.741000000 3.837000000 -14.363000000

H -9.987000000 5.079000000 -15.190000000

N -2.564000000 3.856000000 -23.064000000

C -2.089000000 4.847000000 -22.104000000

C -1.721000000 4.207000000 -20.769000000

O -0.655000000 4.475000000 -20.213000000

C -3.161000000 5.910000000 -21.873000000

C -3.399000000 6.915000000 -22.996000000

C -4.670000000 7.698000000 -22.725000000

C -2.199000000 7.842000000 -23.096000000

H -1.202000000 5.331000000 -22.514000000

H -4.103000000 5.392000000 -21.692000000

H -2.900000000 6.464000000 -20.972000000

H -3.509000000 6.375000000 -23.937000000

H -4.583000000 8.694000000 -23.158000000

H -5.518000000 7.180000000 -23.173000000

H -4.823000000 7.782000000 -21.649000000

H -2.362000000 8.563000000 -23.897000000

H -2.069000000 8.371000000 -22.152000000

H -1.305000000 7.257000000 -23.311000000

H -2.020000000 3.018000000 -22.917000000

H -2.356000000 4.200000000 -23.991000000

N -2.611000000 3.366000000 -20.258000000

H -3.480000000 3.182000000 -20.760000000

C -2.358000000 2.706000000 -18.992000000

C -3.507000000 1.800000000 -18.602000000

O -4.587000000 2.270000000 -18.248000000

H -1.450000000 2.110000000 -19.078000000

H -2.218000000 3.460000000 -18.217000000

N -3.272000000 0.493000000 -18.666000000

H -2.351000000 0.172000000 -18.964000000

C -4.289000000 -0.498000000 -18.324000000

C -4.435000000 -0.697000000 -16.821000000

O -3.535000000 -0.369000000 -16.048000000

C -3.956000000 -1.837000000 -18.987000000

C -4.043000000 -1.816000000 -20.509000000

C -3.513000000 -3.092000000 -21.141000000

O -3.952000000 -4.185000000 -20.718000000

O -2.664000000 -3.002000000 -22.059000000

H -5.246000000 -0.153000000 -18.716000000

H -4.654000000 -2.586000000 -18.615000000

H -2.946000000 -2.128000000 -18.699000000

H -5.087000000 -1.691000000 -20.797000000

H -3.468000000 -0.970000000 -20.885000000

O -5.475000000 -1.196000000 -16.370000000

N -10.051000000 -1.941000000 -14.398000000

H -9.466000000 -1.212000000 -13.990000000

C -11.166000000 -1.542000000 -15.262000000

C -11.060000000 -2.110000000 -16.685000000

O -11.995000000 -2.725000000 -17.208000000

C -11.226000000 -0.013000000 -15.343000000

C -9.876000000 0.638000000 -15.590000000

C -8.922000000 0.709000000 -14.580000000

C -9.557000000 1.185000000 -16.830000000

C -7.693000000 1.311000000 -14.795000000

C -8.328000000 1.789000000 -17.057000000

C -7.398000000 1.853000000 -16.033000000

H -12.094000000 -1.900000000 -14.816000000

H -11.895000000 0.262000000 -16.158000000

H -11.635000000 0.372000000 -14.409000000

H -9.144000000 0.287000000 -13.611000000

H -10.280000000 1.138000000 -17.631000000

H -6.966000000 1.357000000 -13.997000000

H -8.099000000 2.206000000 -18.026000000

O -10.011000000 -1.947000000 -17.324000000

H -10.442000000 -2.472000000 -13.633000000

N -6.914000000 2.232000000 -21.285000000

H -5.958000000 1.916000000 -21.121000000

C -7.141000000 3.614000000 -21.675000000

C -6.212000000 3.896000000 -22.850000000

O -4.998000000 3.971000000 -22.680000000

C -6.812000000 4.580000000 -20.522000000

C -7.120000000 6.006000000 -20.938000000

C -7.614000000 4.206000000 -19.290000000

H -8.178000000 3.746000000 -21.983000000

H -5.750000000 4.502000000 -20.289000000

H -8.115000000 6.280000000 -20.588000000

H -6.383000000 6.679000000 -20.500000000

H -7.083000000 6.084000000 -22.025000000

H -8.092000000 5.097000000 -18.882000000

H -6.949000000 3.776000000 -18.541000000

H -8.377000000 3.476000000 -19.561000000

H -7.296000000 1.651000000 -22.017000000

O -6.685000000 4.051000000 -23.984000000

N -7.810000000 11.109000000 -24.650000000

C -7.667000000 9.874000000 -23.900000000

C -9.024000000 9.198000000 -23.777000000

O -9.997000000 9.801000000 -23.325000000

C -7.092000000 10.166000000 -22.525000000

H -6.987000000 9.211000000 -24.435000000

H -7.645000000 10.985000000 -22.066000000

H -7.175000000 9.277000000 -21.900000000

H -6.043000000 10.445000000 -22.622000000

H -8.157000000 10.859000000 -25.565000000

H -6.888000000 11.505000000 -24.769000000

N -9.086000000 7.939000000 -24.187000000

H -8.246000000 7.484000000 -24.546000000

C -10.334000000 7.200000000 -24.132000000

C -10.241000000 5.992000000 -23.215000000

O -9.362000000 5.136000000 -23.368000000

C -10.757000000 6.739000000 -25.535000000

C -12.086000000 6.006000000 -25.467000000

C -10.854000000 7.941000000 -26.455000000

H -11.107000000 7.864000000 -23.744000000

H -10.000000000 6.058000000 -25.924000000

H -12.376000000 5.684000000 -26.467000000

H -12.849000000 6.673000000 -25.065000000

H -11.988000000 5.135000000 -24.819000000

H -11.154000000 7.614000000 -27.451000000

H -9.883000000 8.434000000 -26.512000000

H -11.594000000 8.639000000 -26.064000000

N -11.160000000 5.939000000 -22.257000000

H -11.852000000 6.686000000 -22.193000000

C -11.208000000 4.850000000 -21.299000000

C -12.247000000 3.846000000 -21.763000000

O -13.295000000 4.220000000 -22.292000000

C -11.569000000 5.384000000 -19.908000000

C -11.561000000 4.333000000 -18.800000000

C -11.519000000 4.986000000 -17.417000000

C -11.276000000 3.955000000 -16.326000000

H -10.233000000 4.365000000 -21.256000000

H -10.850000000 6.160000000 -19.645000000

H -12.562000000 5.832000000 -19.955000000

H -12.463000000 3.726000000 -18.879000000

H -10.687000000 3.693000000 -18.920000000

H -10.715000000 5.721000000 -17.396000000

H -12.468000000 5.488000000 -17.230000000

H -10.509000000 3.260000000 -16.668000000

H -12.199000000 3.403000000 -16.149000000

O -12.039000000 2.635000000 -21.608000000

N -8.842000000 16.763000000 -14.873000000

H -8.499000000 16.166000000 -14.120000000

C -8.904000000 16.251000000 -16.233000000

C -10.236000000 16.661000000 -16.839000000

O -11.274000000 16.583000000 -16.184000000

C -8.757000000 14.730000000 -16.248000000

C -9.094000000 14.181000000 -17.629000000

C -7.333000000 14.369000000 -15.868000000

H -8.096000000 16.693000000 -16.817000000

H -9.441000000 14.299000000 -15.517000000

H -8.581000000 13.231000000 -17.778000000

H -10.171000000 14.029000000 -17.706000000

H -8.772000000 14.891000000 -18.391000000

H -6.638000000 15.006000000 -16.415000000

H -7.144000000 13.325000000 -16.119000000

H -7.195000000 14.516000000 -14.797000000

H -8.271000000 17.596000000 -14.886000000

O -10.277000000 17.078000000 -18.005000000

N -11.852000000 11.728000000 -22.235000000

H -11.275000000 10.976000000 -22.613000000

C -11.252000000 12.660000000 -21.292000000

C -10.018000000 13.331000000 -21.868000000

O -9.420000000 12.837000000 -22.819000000

C -10.880000000 11.902000000 -20.021000000

C -12.053000000 11.078000000 -19.490000000

C -11.581000000 9.694000000 -19.097000000

C -12.706000000 11.813000000 -18.329000000

H -11.985000000 13.427000000 -21.042000000

H -10.048000000 11.232000000 -20.240000000

H -10.571000000 12.616000000 -19.257000000

H -12.789000000 10.975000000 -20.288000000

H -12.424000000 9.115000000 -18.720000000

H -11.158000000 9.194000000 -19.968000000

H -10.821000000 9.776000000 -18.320000000

H -13.542000000 11.225000000 -17.951000000

H -11.975000000 11.958000000 -17.534000000

H -13.068000000 12.783000000 -18.671000000

H -12.184000000 12.271000000 -23.019000000

N -9.636000000 14.455000000 -21.276000000

H -10.171000000 14.803000000 -20.480000000

C -8.474000000 15.205000000 -21.731000000

C -7.220000000 14.350000000 -21.754000000

O -7.030000000 13.486000000 -20.901000000

C -8.221000000 16.412000000 -20.820000000

C -7.118000000 17.331000000 -21.327000000

C -6.586000000 18.273000000 -20.262000000

O -7.384000000 19.025000000 -19.657000000

O -5.359000000 18.261000000 -20.035000000

H -8.667000000 15.566000000 -22.741000000

H -9.143000000 16.988000000 -20.745000000

H -7.950000000 16.052000000 -19.827000000

H -6.294000000 16.716000000 -21.688000000

H -7.506000000 17.922000000 -22.157000000

N -6.362000000 14.589000000 -22.735000000

H -6.577000000 15.295000000 -23.439000000

C -5.117000000 13.848000000 -22.810000000

C -4.047000000 14.649000000 -22.083000000

O -3.991000000 15.874000000 -22.210000000

C -4.678000000 13.637000000 -24.256000000

C -3.285000000 13.061000000 -24.334000000

C -2.980000000 11.863000000 -23.704000000

C -2.267000000 13.732000000 -24.994000000

C -1.696000000 11.347000000 -23.725000000

C -0.975000000 13.224000000 -25.022000000

C -0.697000000 12.030000000 -24.382000000

O 0.585000000 11.521000000 -24.383000000

H 1.274000000 11.992000000 -24.836000000

H -5.240000000 12.881000000 -22.323000000

H -5.372000000 12.950000000 -24.740000000

H -4.699000000 14.593000000 -24.779000000

H -3.760000000 11.323000000 -23.187000000

H -2.482000000 14.665000000 -25.494000000

H -1.479000000 10.413000000 -23.228000000

H -0.192000000 13.758000000 -25.540000000

N -3.202000000 13.962000000 -21.321000000

H -3.300000000 12.949000000 -21.255000000

C -2.138000000 14.627000000 -20.578000000

C -0.785000000 14.060000000 -21.008000000

O -0.324000000 13.047000000 -20.490000000

C -2.346000000 14.435000000 -19.063000000

S -3.922000000 15.086000000 -18.375000000

H -2.162000000 15.693000000 -20.805000000

H -1.526000000 14.935000000 -18.548000000

H -2.290000000 13.369000000 -18.842000000

H -4.393000000 16.048000000 -19.173000000

N -0.155000000 14.722000000 -21.968000000

H -0.586000000 15.560000000 -22.358000000

C 1.138000000 14.285000000 -22.481000000

C 2.219000000 14.358000000 -21.410000000

O 3.280000000 13.738000000 -21.538000000

C 1.550000000 15.164000000 -23.659000000

O 1.827000000 16.483000000 -23.218000000

H 2.083000000 17.030000000 -23.951000000

H 1.051000000 13.254000000 -22.824000000

H 2.443000000 14.746000000 -24.124000000

H 0.741000000 15.190000000 -24.390000000

N 1.940000000 15.127000000 -20.362000000

H 1.041000000 15.608000000 -20.319000000

C 2.889000000 15.295000000 -19.280000000

C 3.228000000 14.008000000 -18.568000000

O 4.370000000 13.798000000 -18.178000000

H 2.465000000 15.989000000 -18.554000000

H 3.806000000 15.726000000 -19.682000000

N 2.239000000 13.140000000 -18.398000000

H 1.306000000 13.360000000 -18.746000000

C 2.477000000 11.879000000 -17.720000000

C 1.959000000 11.933000000 -16.302000000

O 1.180000000 12.813000000 -15.966000000

H 1.966000000 11.081000000 -18.258000000

H 3.548000000 11.675000000 -17.705000000

N 2.386000000 11.001000000 -15.462000000

H 3.035000000 10.286000000 -15.791000000

C 1.941000000 10.985000000 -14.075000000

C 2.933000000 11.709000000 -13.173000000

O 4.133000000 11.724000000 -13.433000000

C 1.763000000 9.545000000 -13.592000000

C 0.936000000 8.685000000 -14.530000000

C 0.578000000 7.333000000 -13.943000000

O 1.397000000 6.783000000 -13.176000000

O -0.520000000 6.819000000 -14.264000000

H 0.979000000 11.494000000 -14.013000000

H 1.270000000 9.567000000 -12.620000000

H 2.747000000 9.090000000 -13.476000000

H 0.013000000 9.216000000 -14.764000000

H 1.496000000 8.531000000 -15.452000000

O 2.522000000 12.292000000 -12.160000000

N -2.651000000 9.314000000 -9.781000000

C -3.941000000 9.877000000 -10.174000000

C -3.811000000 11.368000000 -10.436000000

O -4.801000000 12.050000000 -10.695000000

C -4.998000000 9.655000000 -9.094000000

C -5.537000000 8.241000000 -9.091000000

O -5.763000000 7.652000000 -10.154000000

N -5.765000000 7.690000000 -7.897000000

H -5.579000000 8.176000000 -7.020000000

H -6.128000000 6.737000000 -7.895000000

H -4.271000000 9.389000000 -11.091000000

H -4.552000000 9.861000000 -8.121000000

H -5.823000000 10.348000000 -9.259000000

H -2.692000000 8.323000000 -9.973000000

H -1.946000000 9.714000000 -10.383000000

N -2.589000000 11.876000000 -10.338000000

H -1.813000000 11.266000000 -10.082000000

C -2.334000000 13.286000000 -10.589000000

C -1.522000000 13.372000000 -11.873000000

O -0.364000000 12.969000000 -11.928000000

C -1.585000000 13.910000000 -9.410000000

C -2.408000000 13.837000000 -8.116000000

C -1.596000000 14.305000000 -6.923000000

C -3.670000000 14.674000000 -8.285000000

H -3.283000000 13.804000000 -10.728000000

H -0.647000000 13.374000000 -9.263000000

H -1.368000000 14.954000000 -9.637000000

H -2.700000000 12.800000000 -7.950000000

H -0.702000000 13.689000000 -6.828000000

H -1.307000000 15.346000000 -7.067000000

H -2.196000000 14.217000000 -6.018000000

H -4.262000000 14.629000000 -7.371000000

H -4.256000000 14.283000000 -9.117000000

H -3.395000000 15.709000000 -8.489000000

N -2.154000000 13.882000000 -12.921000000

H -3.115000000 14.209000000 -12.820000000

C -1.496000000 13.980000000 -14.208000000

C -0.940000000 15.358000000 -14.520000000

O -1.238000000 16.336000000 -13.849000000

C -2.456000000 13.539000000 -15.313000000

C -3.008000000 12.130000000 -15.078000000

C -3.818000000 11.709000000 -16.275000000

C -1.871000000 11.156000000 -14.838000000

H -0.659000000 13.282000000 -14.203000000

H -3.290000000 14.240000000 -15.352000000

H -1.931000000 13.559000000 -16.268000000

H -3.654000000 12.144000000 -14.200000000

H -4.213000000 10.706000000 -16.112000000

H -3.184000000 11.710000000 -17.161000000

H -4.644000000 12.406000000 -16.418000000

H -2.184000000 10.153000000 -15.127000000

H -1.604000000 11.163000000 -13.781000000

H -1.007000000 11.452000000 -15.433000000

O -0.166000000 15.495000000 -15.477000000

N -7.232000000 13.290000000 -11.662000000

H -6.640000000 12.908000000 -10.924000000

C -7.919000000 12.392000000 -12.581000000

C -8.383000000 11.096000000 -11.941000000

O -7.817000000 10.651000000 -10.947000000

C -7.017000000 12.057000000 -13.774000000

O -6.332000000 10.827000000 -13.589000000

H -5.773000000 10.619000000 -14.328000000

H -8.799000000 12.911000000 -12.962000000

H -6.284000000 12.854000000 -13.896000000

H -7.627000000 11.993000000 -14.675000000

H -7.932000000 13.871000000 -11.222000000

N -9.409000000 10.496000000 -12.542000000

H -9.805000000 10.944000000 -13.369000000

C -10.005000000 9.238000000 -12.095000000

C -11.043000000 9.433000000 -10.996000000

O -10.729000000 9.410000000 -9.804000000

C -8.935000000 8.242000000 -11.633000000

C -9.461000000 6.806000000 -11.586000000

O -8.656000000 5.868000000 -11.368000000

O -10.685000000 6.617000000 -11.771000000

H -10.515000000 8.796000000 -12.951000000

H -8.600000000 8.525000000 -10.635000000

H -8.088000000 8.288000000 -12.318000000

O -12.228000000 9.619000000 -11.307000000

H 2.204000000 12.744000000 -11.375000000

H -11.878000000 1.696000000 -21.488000000

H -2.014000000 -2.933000000 -22.762000000

H -12.722000000 -3.203000000 -17.614000000

H -13.146000000 9.763000000 -11.548000000

H -4.415000000 18.252000000 -19.860000000

H -10.309000000 17.401000000 -18.908000000

H -11.623000000 6.472000000 -11.913000000

H -6.281000000 -1.583000000 -16.020000000

H 2.026000000 6.360000000 -12.587000000

H 0.434000000 15.601000000 -16.219000000

H -7.052000000 4.171000000 -24.863000000

Azole **6** with surrounded residues

O -6.908532000 9.123176000 -19.364976000

S -6.643518000 8.647600000 -18.000119000

O -6.245779000 7.244402000 -17.771309000

C -8.091256000 8.994324000 -16.997218000

C -8.855666000 7.931146000 -16.523291000

C -10.074343000 8.204082000 -15.896204000

C -10.522098000 9.516441000 -15.709679000

C -11.866943000 9.806327000 -15.077230000

C -9.694268000 10.565902000 -16.149977000

C -8.501864000 10.315886000 -16.811908000

C -5.312407000 9.642724000 -17.332863000

N -5.488717000 10.945688000 -17.113437000

N -4.286128000 11.447910000 -16.769405000

H -4.217944000 12.428422000 -16.549807000

C -3.314454000 10.486544000 -16.782638000

C -3.947019000 9.291582000 -17.142920000

C -1.945659000 10.173712000 -16.629804000

N -2.971869000 8.338423000 -17.193280000

N -1.767275000 8.875772000 -16.875496000

C -3.074421000 6.907801000 -17.429930000

H -8.500171000 6.915645000 -16.656271000

H -10.693253000 7.385835000 -15.543562000

H -9.982632000 11.594317000 -15.958636000

H -7.875690000 11.122934000 -17.164015000

H -1.104223000 10.800420000 -16.373755000

H -3.176772000 6.363928000 -16.485504000

H -3.949829000 6.711436000 -18.047535000

H -2.161025000 6.588391000 -17.931894000

H -12.558169000 10.261418000 -15.791945000

H -12.336177000 8.895315000 -14.698638000

H -11.786753000 10.512101000 -14.244731000

C -5.773323000 8.134513000 -10.611205000

O -5.588202000 8.057750000 -11.815924000

O -5.389532000 12.453335000 -13.249213000

H -5.345691000 13.300150000 -13.763893000

N -2.564000000 3.856000000 -23.064000000

H -1.790000000 3.209000000 -23.339000000

C -3.507000000 1.800000000 -18.602000000

O -4.587000000 2.270000000 -18.248000000

O -3.357000000 0.571000000 -18.640000000

N -10.051000000 -1.941000000 -14.398000000

H -10.037000000 -2.963000000 -14.235000000

H -10.104000000 -1.459000000 -13.475000000

C -7.299000000 -1.436000000 -24.371000000

O -6.417000000 -2.322000000 -24.395000000

O -7.680000000 -0.824000000 -25.394000000

C -6.212000000 3.896000000 -22.850000000

O -4.998000000 3.971000000 -22.680000000

O -6.685000000 4.051000000 -23.984000000

N -11.160000000 5.939000000 -22.257000000

C -12.247000000 3.846000000 -21.763000000

O -13.295000000 4.220000000 -22.292000000

N -10.825000000 4.567000000 -15.042000000

H -10.627000000 3.857000000 -14.316000000

H -9.964000000 5.133000000 -15.152000000

O -12.039000000 2.635000000 -21.608000000

H -10.890000000 6.840000000 -21.846000000

H -10.509000000 5.721000000 -23.032000000

N -19.659000000 7.628000000 -11.972000000

C -18.674000000 9.165000000 -13.615000000

O -17.964000000 10.150000000 -13.771000000

C -15.176000000 6.582000000 -13.982000000

O -14.245000000 6.852000000 -14.766000000

O -15.229000000 5.495000000 -13.377000000

O -19.607000000 8.949000000 -14.400000000

H -20.107000000 8.222000000 -11.265000000

H -19.498000000 6.692000000 -11.560000000

C -18.350000000 15.230000000 -15.328000000

O -17.787000000 16.280000000 -15.632000000

O -19.552000000 15.081000000 -15.588000000

N -8.842000000 16.763000000 -14.873000000

C -10.236000000 16.661000000 -16.839000000

O -11.274000000 16.583000000 -16.184000000

H -8.501000000 17.734000000 -14.843000000

H -8.265000000 16.189000000 -14.248000000

O -10.277000000 17.078000000 -18.005000000

N -11.852000000 11.728000000 -22.235000000

C -10.018000000 13.331000000 -21.868000000

O -9.420000000 12.837000000 -22.819000000

O -9.610000000 14.390000000 -21.372000000

H -11.899000000 10.760000000 -21.881000000

H -12.794000000 12.014000000 -22.531000000

N 2.386000000 11.001000000 -15.462000000

C 2.933000000 11.709000000 -13.173000000

O 4.133000000 11.724000000 -13.433000000

C 0.578000000 7.333000000 -13.943000000

O 1.397000000 6.783000000 -13.176000000

O -0.520000000 6.819000000 -14.264000000

O 2.522000000 12.292000000 -12.160000000

H 2.155000000 10.108000000 -15.947000000

H 1.990000000 11.781000000 -15.993000000

N -0.126000000 8.721000000 -8.903000000

C -0.073000000 6.184000000 -7.674000000

O -0.179000000 7.228000000 -6.994000000

O 0.805000000 5.319000000 -7.452000000

H -0.246000000 9.743000000 -8.897000000

H 0.883000000 8.531000000 -8.746000000

C -3.811000000 11.368000000 -10.436000000

O -4.801000000 12.050000000 -10.695000000

N -7.232000000 13.290000000 -11.662000000

C -9.461000000 6.806000000 -11.586000000

O -8.656000000 5.868000000 -11.368000000

O -10.685000000 6.617000000 -11.771000000

C -14.265000000 8.614000000 -10.320000000

O -15.444000000 8.727000000 -9.974000000

H -4.597000000 4.583000000 -23.396000000

H -6.916000000 14.150000000 -12.098000000

H -7.806000000 13.498000000 -10.831000000

O -13.806000000 7.488000000 -10.558000000

H -2.907000000 4.287000000 -23.941000000

O -2.695000000 11.903000000 -10.388000000

C -2.100000000 4.804000000 -21.944000000

C -1.815000000 3.756000000 -20.814000000

O -1.128000000 2.821000000 -21.212000000

C -3.117000000 5.867000000 -21.597000000

C -3.374000000 6.903000000 -22.708000000

C -4.427000000 7.897000000 -22.208000000

C -2.091000000 7.654000000 -23.075000000

H -1.127000000 5.265000000 -22.251000000

H -4.083000000 5.425000000 -21.260000000

H -2.755000000 6.422000000 -20.690000000

H -3.768000000 6.398000000 -23.621000000

H -4.674000000 8.645000000 -22.970000000

H -4.083000000 8.452000000 -21.322000000

H -5.365000000 7.407000000 -21.921000000

H -2.301000000 8.468000000 -23.782000000

H -1.635000000 8.124000000 -22.193000000

H -1.340000000 7.015000000 -23.541000000

N -2.417000000 3.821000000 -19.618000000

C -2.254000000 2.699000000 -18.691000000

H -2.054000000 3.094000000 -17.660000000

H -1.386000000 2.056000000 -18.972000000

H -2.885000000 4.637000000 -19.225000000

C -11.163000000 -1.464000000 -15.325000000

C -10.994000000 -2.117000000 -16.721000000

O -11.934000000 -2.699000000 -17.214000000

C -11.130000000 0.077000000 -15.431000000

C -9.758000000 0.610000000 -15.674000000

C -8.885000000 0.832000000 -14.592000000

C -9.310000000 0.896000000 -16.972000000

C -7.569000000 1.155000000 -14.805000000

C -7.990000000 1.223000000 -17.217000000

C -7.071000000 1.279000000 -16.140000000

O -5.797000000 1.398000000 -16.256000000

H -5.322000000 1.668000000 -17.259000000

H -12.148000000 -1.800000000 -14.906000000

H -11.826000000 0.379000000 -16.253000000

H -11.580000000 0.528000000 -14.519000000

H -9.264000000 0.776000000 -13.572000000

H -10.008000000 0.838000000 -17.813000000

H -6.872000000 1.338000000 -13.986000000

H -7.632000000 1.405000000 -18.228000000

N -9.752000000 -2.046000000 -17.275000000

H -9.015000000 -1.439000000 -16.901000000

C -9.528000000 -2.479000000 -18.663000000

C -8.216000000 -1.783000000 -19.089000000

O -7.214000000 -1.966000000 -18.425000000

H -9.390000000 -3.584000000 -18.706000000

H -10.401000000 -2.240000000 -19.310000000

N -8.306000000 -0.928000000 -20.136000000

H -9.161000000 -0.793000000 -20.679000000

C -7.120000000 -0.249000000 -20.682000000

C -7.573000000 1.185000000 -20.980000000

O -8.739000000 1.463000000 -21.160000000

C -6.714000000 -0.897000000 -22.023000000

C -7.879000000 -1.020000000 -22.999000000

H -6.271000000 -0.290000000 -19.946000000

H -6.278000000 -1.905000000 -21.846000000

H -5.891000000 -0.318000000 -22.486000000

H -8.602000000 -1.790000000 -22.699000000

H -8.430000000 -0.074000000 -23.131000000

N -6.587000000 2.161000000 -21.170000000

C -7.062000000 3.456000000 -21.644000000

C -7.027000000 4.553000000 -20.562000000

C -7.546000000 5.883000000 -21.111000000

C -7.863000000 4.115000000 -19.359000000

H -8.122000000 3.338000000 -22.043000000

H -5.964000000 4.680000000 -20.245000000

H -8.173000000 4.982000000 -18.748000000

H -7.291000000 3.452000000 -18.695000000

H -8.771000000 3.578000000 -19.656000000

H -8.583000000 5.809000000 -21.437000000

H -7.522000000 6.650000000 -20.308000000

H -6.949000000 6.259000000 -21.944000000

H -5.719000000 2.140000000 -20.650000000

C -11.182000000 4.847000000 -21.216000000

C -11.581000000 5.436000000 -19.875000000

C -11.562000000 4.349000000 -18.789000000

C -11.262000000 4.999000000 -17.438000000

C -11.272000000 3.941000000 -16.338000000

H -10.192000000 4.329000000 -21.169000000

H -12.600000000 5.878000000 -19.920000000

H -10.886000000 6.256000000 -19.589000000

H -12.534000000 3.813000000 -18.782000000

H -10.812000000 3.568000000 -19.031000000

H -10.273000000 5.515000000 -17.495000000

H -12.017000000 5.791000000 -17.221000000

H -12.300000000 3.526000000 -16.201000000

H -10.593000000 3.092000000 -16.589000000

C -18.322000000 8.201000000 -12.468000000

C -17.429000000 7.076000000 -12.981000000

C -16.224000000 7.672000000 -13.713000000

H -17.803000000 8.739000000 -11.625000000

H -17.018000000 6.459000000 -12.140000000

H -17.963000000 6.389000000 -13.659000000

H -15.724000000 8.439000000 -13.080000000

H -16.511000000 8.151000000 -14.659000000

C -17.519000000 14.065000000 -14.703000000

C -17.243000000 13.088000000 -15.836000000

C -16.023000000 12.209000000 -15.618000000

O -14.894000000 12.667000000 -15.586000000

N -16.235000000 10.856000000 -15.584000000

H -15.471000000 10.234000000 -15.368000000

H -17.153000000 10.470000000 -15.367000000

H -16.578000000 14.452000000 -14.243000000

H -17.040000000 13.689000000 -16.765000000

H -18.148000000 12.502000000 -16.097000000

C -8.843000000 16.216000000 -16.282000000

C -8.688000000 14.694000000 -16.294000000

C -9.033000000 14.185000000 -17.696000000

C -7.253000000 14.295000000 -15.939000000

H -8.047000000 16.706000000 -16.886000000

H -9.408000000 14.233000000 -15.571000000

H -8.850000000 13.111000000 -17.796000000

H -8.456000000 14.691000000 -18.485000000

H -10.088000000 14.391000000 -17.951000000

H -6.522000000 14.803000000 -16.580000000

H -6.982000000 14.487000000 -14.901000000

H -7.091000000 13.219000000 -16.125000000

C -11.284000000 12.691000000 -21.209000000

C -10.960000000 11.952000000 -19.930000000

C -12.239000000 11.554000000 -19.156000000

C -12.152000000 10.094000000 -18.699000000

C -12.443000000 12.487000000 -17.962000000

H -12.016000000 13.517000000 -21.022000000

H -10.322000000 12.623000000 -19.299000000

H -10.294000000 11.087000000 -20.106000000

H -13.131000000 11.654000000 -19.819000000

H -13.353000000 12.243000000 -17.391000000

H -12.544000000 13.537000000 -18.271000000

H -11.608000000 12.441000000 -17.254000000

H -13.003000000 9.834000000 -18.050000000

H -12.144000000 9.393000000 -19.531000000

H -11.250000000 9.915000000 -18.097000000

C 1.881000000 10.961000000 -14.030000000

C 1.760000000 9.513000000 -13.587000000

C 0.859000000 8.736000000 -14.531000000

H 0.901000000 11.491000000 -13.962000000

H 2.748000000 9.014000000 -13.482000000

H 1.353000000 9.481000000 -12.550000000

H -0.121000000 9.227000000 -14.665000000

H 1.296000000 8.567000000 -15.525000000

C -0.558000000 8.081000000 -10.197000000

C -1.993000000 8.413000000 -10.619000000

O -2.454000000 7.922000000 -11.624000000

C -0.404000000 6.557000000 -10.113000000

C -1.025000000 5.965000000 -8.855000000

H 0.108000000 8.455000000 -11.035000000

H -0.858000000 6.108000000 -11.036000000

H 0.665000000 6.262000000 -10.200000000

H -2.024000000 6.383000000 -8.646000000

H -1.173000000 4.874000000 -8.982000000

N -2.755000000 9.261000000 -9.827000000

H -2.325000000 9.855000000 -9.136000000

C -3.950000000 9.833000000 -10.459000000

C -5.227000000 9.304000000 -9.803000000

N -6.528000000 7.193000000 -10.030000000

H -6.782000000 7.239000000 -9.069000000

H -7.101000000 6.542000000 -10.610000000

H -3.968000000 9.564000000 -11.563000000

H -5.043000000 9.002000000 -8.758000000

H -5.992000000 10.121000000 -9.764000000

C -7.809000000 12.367000000 -12.697000000

C -8.400000000 11.128000000 -11.967000000

O -8.027000000 10.904000000 -10.840000000

C -6.609000000 11.826000000 -13.557000000

H -8.568000000 12.891000000 -13.308000000

H -6.437000000 10.740000000 -13.378000000

H -6.784000000 11.984000000 -14.634000000

N -9.156000000 10.349000000 -12.793000000

H -9.526000000 10.720000000 -13.657000000

C -9.900000000 9.213000000 -12.215000000

C -10.944000000 9.856000000 -11.271000000

O -10.704000000 10.944000000 -10.763000000

C -8.944000000 8.247000000 -11.539000000

H -10.429000000 8.679000000 -13.056000000

H -7.956000000 8.276000000 -12.048000000

H -8.769000000 8.530000000 -10.485000000

N -12.152000000 9.249000000 -11.254000000

C -13.285000000 9.821000000 -10.513000000

C -14.010000000 10.929000000 -11.305000000

C -13.190000000 12.147000000 -11.599000000

C -13.206000000 12.707000000 -12.878000000

C -12.416000000 12.763000000 -10.606000000

C -12.445000000 13.836000000 -13.176000000

C -11.654000000 13.889000000 -10.902000000

C -11.660000000 14.428000000 -12.190000000

H -12.962000000 10.198000000 -9.518000000

H -14.912000000 11.213000000 -10.717000000

H -14.418000000 10.485000000 -12.239000000

H -13.822000000 12.266000000 -13.665000000

H -12.376000000 12.330000000 -9.606000000

H -12.483000000 14.261000000 -14.180000000

H -11.050000000 14.345000000 -10.125000000

H -11.082000000 15.314000000 -12.412000000

H -12.221000000 8.479000000 -11.479000000

N -18.249000000 13.397000000 -13.616000000

H -18.357000000 13.948000000 -12.775000000

H -17.934000000 12.459000000 -13.406000000

H 2.204000000 12.744000000 -11.375000000

H -10.309000000 17.401000000 -18.908000000

H -11.878000000 1.696000000 -21.488000000

H -7.972000000 -0.355000000 -26.179000000

H -20.483000000 14.966000000 -15.789000000

H -15.270000000 4.657000000 -12.911000000

H -13.450000000 6.616000000 -10.742000000

H -3.241000000 -0.381000000 -18.669000000

H -9.294000000 15.211000000 -20.988000000

H 2.026000000 6.360000000 -12.587000000

H 1.478000000 4.656000000 -7.282000000

H -8.040000000 5.151000000 -11.201000000

H -1.830000000 12.318000000 -10.351000000

H -20.330000000 8.782000000 -15.009000000

Azole **7** with surrounded residues

C -4.180137000 8.011533000 -18.232607000

C -2.765947000 8.027785000 -18.454268000

C -4.717473000 6.908197000 -17.533054000

N -1.934587000 7.072634000 -17.962397000

C -3.857353000 5.944987000 -17.047069000

C -2.472115000 6.087347000 -17.264782000

C -4.928767000 9.135798000 -18.719671000

C -4.254589000 10.152404000 -19.425846000

C -2.148779000 9.078889000 -19.213719000

C -2.866222000 10.128358000 -19.712020000

C -6.272257000 9.629322000 -18.646372000

N -6.402355000 10.814230000 -19.224314000

N -5.183656000 11.110383000 -19.704354000

H -5.044161000 12.008759000 -20.131862000

S -7.717994000 9.009177000 -17.776074000

O -7.798040000 7.557471000 -18.022145000

O -7.667703000 9.516568000 -16.396709000

C -9.086829000 9.792588000 -18.625032000

C -9.754318000 9.087453000 -19.624565000

C -10.827651000 9.690135000 -20.274822000

C -11.227231000 10.994421000 -19.953863000

C -12.392734000 11.640116000 -20.662275000

C -10.536528000 11.678250000 -18.941763000

C -9.474281000 11.084406000 -18.266784000

H -5.789412000 6.827600000 -17.391587000

H -4.235389000 5.087928000 -16.499203000

H -1.776840000 5.358947000 -16.850885000

H -1.078093000 9.000945000 -19.366361000

H -2.393388000 10.920904000 -20.281558000

H -9.436980000 8.084567000 -19.876653000

H -11.361162000 9.138688000 -21.044149000

H -10.837051000 12.688894000 -18.678801000

H -8.949503000 11.607907000 -17.475814000

H -12.512812000 11.245129000 -21.674997000

H -13.330739000 11.453912000 -20.124822000

H -12.267385000 12.724556000 -20.730773000

C -7.283158000 14.338181000 -21.778147000

O -7.102995000 13.486299000 -20.922163000

O -6.306470000 10.861103000 -13.575044000

H -5.908779000 10.621901000 -14.450743000

N -2.564000000 3.856000000 -23.064000000

C -2.089000000 4.847000000 -22.104000000

C -1.721000000 4.207000000 -20.769000000

O -0.655000000 4.475000000 -20.213000000

C -3.161000000 5.910000000 -21.873000000

C -3.399000000 6.915000000 -22.996000000

C -4.670000000 7.698000000 -22.725000000

C -2.199000000 7.842000000 -23.096000000

H -2.020000000 3.018000000 -22.917000000

H -2.356000000 4.200000000 -23.991000000

H -1.202000000 5.331000000 -22.514000000

H -2.878000000 6.473000000 -20.984000000

H -4.103000000 5.402000000 -21.667000000

H -3.509000000 6.375000000 -23.937000000

H -4.583000000 8.694000000 -23.158000000

H -5.518000000 7.180000000 -23.173000000

H -4.823000000 7.782000000 -21.649000000

H -2.362000000 8.563000000 -23.897000000

H -1.305000000 7.257000000 -23.311000000

H -2.069000000 8.371000000 -22.152000000

N -2.611000000 3.366000000 -20.258000000

C -2.358000000 2.706000000 -18.992000000

C -3.507000000 1.800000000 -18.602000000

O -4.587000000 2.270000000 -18.248000000

H -1.450000000 2.110000000 -19.078000000

H -2.218000000 3.460000000 -18.217000000

H -3.480000000 3.182000000 -20.760000000

O -3.357000000 0.571000000 -18.640000000

N -6.914000000 2.232000000 -21.285000000

C -7.141000000 3.614000000 -21.675000000

C -6.212000000 3.896000000 -22.850000000

O -4.998000000 3.971000000 -22.680000000

C -6.812000000 4.580000000 -20.522000000

C -7.120000000 6.006000000 -20.938000000

C -7.614000000 4.206000000 -19.290000000

H -8.178000000 3.746000000 -21.983000000

H -5.750000000 4.502000000 -20.289000000

H -7.383000000 3.180000000 -19.003000000

H -7.357000000 4.879000000 -18.472000000

H -8.678000000 4.291000000 -19.510000000

H -8.115000000 6.280000000 -20.588000000

H -6.383000000 6.679000000 -20.500000000

H -7.083000000 6.084000000 -22.025000000

H -6.238000000 1.671000000 -21.783000000

O -6.685000000 4.051000000 -23.984000000

H -6.625000000 2.246000000 -20.317000000

N -7.810000000 11.109000000 -24.650000000

C -7.667000000 9.874000000 -23.900000000

C -9.024000000 9.198000000 -23.777000000

O -9.997000000 9.801000000 -23.325000000

C -7.092000000 10.166000000 -22.525000000

H -6.987000000 9.211000000 -24.435000000

H -7.645000000 10.985000000 -22.066000000

H -7.175000000 9.277000000 -21.900000000

H -6.043000000 10.445000000 -22.622000000

H -8.717000000 11.388000000 -24.995000000

H -7.185000000 11.059000000 -25.442000000

N -9.086000000 7.939000000 -24.187000000

H -8.246000000 7.484000000 -24.546000000

C -10.334000000 7.200000000 -24.132000000

C -10.241000000 5.992000000 -23.215000000

O -9.362000000 5.136000000 -23.368000000

C -10.757000000 6.739000000 -25.535000000

C -12.086000000 6.006000000 -25.467000000

C -10.854000000 7.941000000 -26.455000000

H -11.107000000 7.864000000 -23.744000000

H -10.000000000 6.058000000 -25.924000000

H -12.376000000 5.684000000 -26.467000000

H -12.849000000 6.673000000 -25.065000000

H -11.988000000 5.135000000 -24.819000000

H -11.154000000 7.614000000 -27.451000000

H -9.883000000 8.434000000 -26.512000000

H -11.594000000 8.639000000 -26.064000000

N -11.160000000 5.939000000 -22.257000000

C -11.208000000 4.850000000 -21.299000000

C -12.247000000 3.846000000 -21.763000000

O -13.295000000 4.220000000 -22.292000000

C -11.569000000 5.384000000 -19.908000000

C -11.561000000 4.333000000 -18.800000000

C -11.519000000 4.986000000 -17.417000000

C -11.276000000 3.955000000 -16.326000000

N -10.825000000 4.567000000 -15.042000000

H -11.480000000 5.283000000 -14.729000000

H -10.000000000 5.149000000 -15.185000000

H -10.233000000 4.365000000 -21.256000000

H -12.568000000 5.816000000 -19.959000000

H -10.863000000 6.172000000 -19.645000000

H -12.463000000 3.726000000 -18.879000000

H -10.687000000 3.693000000 -18.920000000

H -12.471000000 5.483000000 -17.230000000

H -10.719000000 5.726000000 -17.395000000

H -10.509000000 3.260000000 -16.668000000

H -12.199000000 3.403000000 -16.149000000

H -11.852000000 6.686000000 -22.193000000

O -12.039000000 2.635000000 -21.608000000

N -19.659000000 7.628000000 -11.972000000

C -18.418000000 8.207000000 -12.466000000

C -18.674000000 9.165000000 -13.615000000

O -17.964000000 10.150000000 -13.771000000

C -17.459000000 7.107000000 -12.934000000

C -16.274000000 7.626000000 -13.753000000

C -15.176000000 6.582000000 -13.982000000

O -14.245000000 6.852000000 -14.766000000

O -15.229000000 5.495000000 -13.377000000

H -17.944000000 8.758000000 -11.654000000

H -18.019000000 6.402000000 -13.549000000

H -17.076000000 6.581000000 -12.059000000

H -16.645000000 7.953000000 -14.724000000

H -15.840000000 8.483000000 -13.237000000

H -20.539000000 7.884000000 -12.396000000

O -19.607000000 8.949000000 -14.400000000

H -19.572000000 6.624000000 -12.032000000

N -18.408000000 13.436000000 -13.639000000

C -17.583000000 14.107000000 -14.633000000

C -18.350000000 15.230000000 -15.328000000

O -17.787000000 16.280000000 -15.632000000

C -17.068000000 13.094000000 -15.661000000

C -15.763000000 12.428000000 -15.228000000

O -14.713000000 13.071000000 -15.171000000

N -15.826000000 11.140000000 -14.919000000

H -14.955000000 10.695000000 -14.630000000

H -16.695000000 10.608000000 -14.966000000

H -16.724000000 14.544000000 -14.125000000

H -17.825000000 12.322000000 -15.799000000

H -16.906000000 13.605000000 -16.610000000

H -19.360000000 13.736000000 -13.487000000

H -18.448000000 12.462000000 -13.903000000

O -19.552000000 15.081000000 -15.588000000

N -9.636000000 14.455000000 -21.276000000

H -10.171000000 14.803000000 -20.480000000

C -8.474000000 15.205000000 -21.731000000

C -8.221000000 16.412000000 -20.820000000

C -7.118000000 17.331000000 -21.327000000

C -6.586000000 18.273000000 -20.262000000

O -7.384000000 19.025000000 -19.657000000

O -5.359000000 18.261000000 -20.035000000

H -8.667000000 15.566000000 -22.741000000

H -9.143000000 16.988000000 -20.745000000

H -7.950000000 16.052000000 -19.827000000

H -7.515000000 17.927000000 -22.149000000

H -6.295000000 16.721000000 -21.700000000

H -10.274000000 14.396000000 -22.057000000

N -6.362000000 14.589000000 -22.735000000

H -6.577000000 15.295000000 -23.439000000

C -5.117000000 13.848000000 -22.810000000

C -4.047000000 14.649000000 -22.083000000

O -3.991000000 15.874000000 -22.210000000

C -4.678000000 13.637000000 -24.256000000

C -3.285000000 13.061000000 -24.334000000

C -2.980000000 11.863000000 -23.704000000

C -2.267000000 13.732000000 -24.994000000

C -1.696000000 11.347000000 -23.725000000

C -0.975000000 13.224000000 -25.022000000

C -0.697000000 12.030000000 -24.382000000

O 0.585000000 11.521000000 -24.383000000

H 1.274000000 11.992000000 -24.836000000

H -5.240000000 12.881000000 -22.323000000

H -5.372000000 12.950000000 -24.740000000

H -4.699000000 14.593000000 -24.779000000

H -3.760000000 11.323000000 -23.187000000

H -2.482000000 14.665000000 -25.494000000

H -1.479000000 10.413000000 -23.228000000

H -0.192000000 13.758000000 -25.540000000

N -3.202000000 13.962000000 -21.321000000

C -2.138000000 14.627000000 -20.578000000

C -0.785000000 14.060000000 -21.008000000

O -0.324000000 13.047000000 -20.490000000

C -2.346000000 14.435000000 -19.063000000

S -3.922000000 15.086000000 -18.375000000

H -2.162000000 15.693000000 -20.805000000

H -2.308000000 13.366000000 -18.854000000

H -1.519000000 14.916000000 -18.540000000

H -4.389000000 16.054000000 -19.169000000

H -3.300000000 12.949000000 -21.255000000

O -0.141000000 14.634000000 -21.897000000

N 2.386000000 11.001000000 -15.462000000

C 1.941000000 10.985000000 -14.075000000

C 2.933000000 11.709000000 -13.173000000

O 4.133000000 11.724000000 -13.433000000

C 1.763000000 9.545000000 -13.592000000

C 0.936000000 8.685000000 -14.530000000

C 0.578000000 7.333000000 -13.943000000

O 1.397000000 6.783000000 -13.176000000

O -0.520000000 6.819000000 -14.264000000

H 0.979000000 11.494000000 -14.013000000

H 1.270000000 9.567000000 -12.620000000

H 2.747000000 9.090000000 -13.476000000

H 0.013000000 9.216000000 -14.764000000

H 1.496000000 8.531000000 -15.452000000

H 3.253000000 11.456000000 -15.709000000

H 1.653000000 11.424000000 -16.013000000

O 2.522000000 12.292000000 -12.160000000

N -2.154000000 13.882000000 -12.921000000

C -1.496000000 13.980000000 -14.208000000

C -0.940000000 15.358000000 -14.520000000

O -1.238000000 16.336000000 -13.849000000

C -2.456000000 13.539000000 -15.313000000

C -3.008000000 12.130000000 -15.078000000

C -3.818000000 11.709000000 -16.275000000

C -1.871000000 11.156000000 -14.838000000

H -0.659000000 13.282000000 -14.203000000

H -1.924000000 13.552000000 -16.264000000

H -3.288000000 14.242000000 -15.362000000

H -3.654000000 12.144000000 -14.200000000

H -4.213000000 10.706000000 -16.112000000

H -4.644000000 12.406000000 -16.418000000

H -3.184000000 11.710000000 -17.162000000

H -2.184000000 10.153000000 -15.127000000

H -1.007000000 11.452000000 -15.433000000

H -1.603000000 11.163000000 -13.781000000

H -2.211000000 14.692000000 -12.320000000

H -3.096000000 13.556000000 -13.084000000

O -0.166000000 15.495000000 -15.477000000

N -7.232000000 13.290000000 -11.662000000

C -7.919000000 12.392000000 -12.581000000

C -8.383000000 11.096000000 -11.941000000

O -7.817000000 10.651000000 -10.947000000

C -7.017000000 12.057000000 -13.774000000

H -8.799000000 12.911000000 -12.962000000

H -6.284000000 12.854000000 -13.896000000

H -7.627000000 11.993000000 -14.675000000

H -7.104000000 13.023000000 -10.696000000

H -6.311000000 13.450000000 -12.045000000

N -9.409000000 10.496000000 -12.542000000

H -9.805000000 10.944000000 -13.369000000

C -10.005000000 9.238000000 -12.095000000

C -11.043000000 9.433000000 -10.996000000

O -10.729000000 9.410000000 -9.804000000

C -8.935000000 8.242000000 -11.633000000

C -9.461000000 6.806000000 -11.586000000

O -8.656000000 5.868000000 -11.368000000

O -10.685000000 6.617000000 -11.771000000

H -10.515000000 8.796000000 -12.951000000

H -8.094000000 8.285000000 -12.325000000

H -8.591000000 8.527000000 -10.639000000

N -12.293000000 9.620000000 -11.407000000

C -13.367000000 9.829000000 -10.459000000

C -14.265000000 8.614000000 -10.320000000

O -15.444000000 8.727000000 -9.974000000

C -14.166000000 11.065000000 -10.864000000

C -13.355000000 12.320000000 -10.833000000

C -12.522000000 12.648000000 -11.889000000

C -13.352000000 13.128000000 -9.704000000

C -11.703000000 13.748000000 -11.816000000

C -12.526000000 14.234000000 -9.632000000

C -11.704000000 14.540000000 -10.684000000

H -12.919000000 10.026000000 -9.485000000

H -14.542000000 10.919000000 -11.877000000

H -15.013000000 11.175000000 -10.186000000

H -12.516000000 12.034000000 -12.777000000

H -14.001000000 12.890000000 -8.874000000

H -11.057000000 13.994000000 -12.645000000

H -12.529000000 14.855000000 -8.749000000

H -11.056000000 15.402000000 -10.628000000

H -12.499000000 9.616000000 -12.406000000

O -13.806000000 7.488000000 -10.558000000

H 0.434000000 15.601000000 -16.219000000

H 2.204000000 12.744000000 -11.375000000

H -7.996000000 19.601000000 -19.193000000

H -20.483000000 14.966000000 -15.789000000

H -13.528000000 7.060000000 -15.370000000

H -8.040000000 5.151000000 -11.201000000

H -11.878000000 1.696000000 -21.488000000

H -13.450000000 6.616000000 -10.742000000

H 2.026000000 6.360000000 -12.587000000

H 0.358000000 15.079000000 -22.586000000

H -3.241000000 -0.381000000 -18.669000000

H -7.052000000 4.171000000 -24.863000000

H -20.330000000 8.782000000 -15.009000000

# **Cartesian coordinates of azoles 1‑7 with corresponding residues around 4 Å after optimization with PM7 method (*Mopac 2016* software)**

Azole **1** with surrounded residues

N -2.564000000 3.856000000 -23.064000000

C -1.721000000 4.207000000 -20.769000000

O -0.655000000 4.475000000 -20.213000000

H -2.738000000 2.956000000 -22.557000000

H -1.922000000 3.693000000 -23.837000000

H -3.546000000 4.119000000 -23.428000000

O -2.504000000 3.408000000 -20.237000000

N -6.914000000 2.232000000 -21.285000000

C -6.212000000 3.896000000 -22.850000000

O -4.998000000 3.971000000 -22.680000000

H -5.887000000 2.032000000 -21.299000000

H -7.256000000 2.041000000 -20.324000000

H -7.393000000 1.581000000 -21.932000000

O -6.685000000 4.051000000 -23.984000000

N -7.810000000 11.109000000 -24.650000000

H -8.532000000 11.726000000 -24.162000000

H -6.925000000 11.672000000 -24.651000000

H -8.120000000 10.966000000 -25.621000000

C -12.247000000 3.846000000 -21.763000000

O -13.295000000 4.220000000 -22.292000000

N -10.825000000 4.567000000 -15.042000000

H -10.594000000 3.861000000 -14.318000000

H -11.601000000 5.165000000 -14.615000000

H -9.999000000 5.174000000 -15.161000000

O -12.039000000 2.635000000 -21.608000000

N -19.659000000 7.628000000 -11.972000000

C -18.674000000 9.165000000 -13.615000000

O -17.964000000 10.150000000 -13.771000000

C -15.176000000 6.582000000 -13.982000000

O -14.245000000 6.852000000 -14.766000000

O -15.229000000 5.495000000 -13.377000000

H -20.302000000 7.472000000 -12.788000000

H -20.139000000 8.254000000 -11.314000000

H -19.486000000 6.721000000 -11.500000000

O -19.607000000 8.949000000 -14.400000000

N -18.408000000 13.436000000 -13.639000000

C -18.350000000 15.230000000 -15.328000000

O -17.787000000 16.280000000 -15.632000000

H -19.418000000 13.568000000 -13.907000000

H -18.271000000 13.810000000 -12.693000000

H -18.218000000 12.396000000 -13.624000000

O -19.552000000 15.081000000 -15.588000000

N -8.842000000 16.763000000 -14.873000000

C -10.236000000 16.661000000 -16.839000000

O -11.274000000 16.583000000 -16.184000000

H -9.828000000 16.742000000 -14.479000000

H -8.218000000 16.234000000 -14.264000000

H -8.541000000 17.758000000 -14.875000000

O -10.277000000 17.078000000 -18.005000000

N -11.852000000 11.728000000 -22.235000000

H -11.118000000 11.049000000 -22.585000000

H -12.617000000 11.160000000 -21.781000000

H -12.234000000 12.204000000 -23.067000000

C -6.586000000 18.273000000 -20.262000000

O -7.384000000 19.025000000 -19.657000000

O -5.359000000 18.261000000 -20.035000000

C -0.785000000 14.060000000 -21.008000000

O -0.324000000 13.047000000 -20.490000000

O -0.141000000 14.634000000 -21.897000000

N -2.154000000 13.882000000 -12.921000000

C -0.940000000 15.358000000 -14.520000000

O -1.238000000 16.336000000 -13.849000000

H -2.285000000 14.874000000 -12.566000000

H -1.581000000 13.380000000 -12.228000000

H -3.071000000 13.417000000 -12.970000000

O -0.166000000 15.495000000 -15.477000000

N -7.232000000 13.290000000 -11.662000000

H -7.102000000 12.771000000 -10.738000000

H -6.290000000 13.551000000 -11.992000000

H -7.773000000 14.143000000 -11.466000000

C -9.461000000 6.806000000 -11.586000000

O -8.656000000 5.868000000 -11.368000000

O -10.685000000 6.617000000 -11.771000000

C -14.265000000 8.614000000 -10.320000000

O -15.444000000 8.727000000 -9.974000000

O -13.806000000 7.488000000 -10.558000000

C -2.026372904 4.916381404 -22.124666993

C -3.025978288 6.035788570 -21.889059348

C -3.078865729 7.055395884 -23.040180380

C -4.359094764 7.899870908 -22.925016871

C -1.851952373 7.970106033 -23.008281908

H -1.067288172 5.311917402 -22.536635029

H -2.759898842 6.550017749 -20.940607203

H -4.036521520 5.612419986 -21.688725763

H -3.106567027 6.511871414 -24.012317504

H -4.386878302 8.668694324 -23.701710935

H -4.410756877 8.395982168 -21.947954019

H -5.247452688 7.268661048 -23.032834007

H -1.863839705 8.691827319 -23.833832015

H -0.916528412 7.405109092 -23.080907280

H -1.798815095 8.544234543 -22.073684040

C -7.118219529 3.703126541 -21.617118719

C -6.629444442 4.569523848 -20.438401138

C -6.796656564 6.048772742 -20.797131627

C -7.406497610 4.274653185 -19.152723250

H -8.187693012 3.909581179 -21.874494912

H -5.530851967 4.375063564 -20.272300738

H -7.133184527 3.326420035 -18.688779065

H -8.490106694 4.313556558 -19.310428578

H -7.183963408 5.053540032 -18.396019236

H -7.849846433 6.358295329 -20.757699403

H -6.236412082 6.684096684 -20.099500301

H -6.434390891 6.260973013 -21.810587082

C -7.641942018 9.793364982 -23.907703609

C -9.037693503 9.144075193 -23.905271591

O -10.018681618 9.826903306 -23.629157108

C -7.193068339 10.044261969 -22.485985566

H -6.881516782 9.171108371 -24.458919183

H -7.792950492 10.816987106 -21.969163916

H -6.136070070 10.354475722 -22.415608789

H -7.280216210 9.134402489 -21.861700780

N -9.114429196 7.870986043 -24.348449537

H -8.294786913 7.244724545 -24.369055268

C -10.394508824 7.145455281 -24.223562718

C -10.234179455 6.326951830 -22.924898333

O -9.103445889 6.054192338 -22.548593353

C -10.579734732 6.182597819 -25.424850408

C -11.913847329 5.448419603 -25.307024054

C -10.534131585 6.968307857 -26.739658251

H -11.248199387 7.862507889 -24.166627424

H -9.746604353 5.430365072 -25.416061613

H -10.698856347 6.300752983 -27.595466255

H -11.309186724 7.740131159 -26.776885849

H -9.566702527 7.453880253 -26.893255179

H -12.098978041 4.817915193 -26.186032799

H -11.953005000 4.758782236 -24.442698344

H -12.765932328 6.128039912 -25.212259032

N -11.349197641 6.013153268 -22.225004763

C -11.257443206 4.928407425 -21.227749909

C -11.704897336 5.459371722 -19.860165651

C -11.510252212 4.384281430 -18.785751595

C -11.559501581 5.007764117 -17.387007159

C -11.281567331 3.932176319 -16.334304058

H -10.226212230 4.513913689 -21.177233382

H -12.773034306 5.760857147 -19.907550356

H -11.131077146 6.372328299 -19.614347498

H -12.293696631 3.603810293 -18.911386422

H -10.551667809 3.857886604 -18.947016113

H -10.828285124 5.838412618 -17.328063488

H -12.559368138 5.475925754 -17.214052859

H -12.213265558 3.344507902 -16.133196504

H -10.504260037 3.217867349 -16.680059155

H -12.296103432 6.115312316 -22.615618887

C -18.324276407 8.201389927 -12.467800910

C -17.426813046 7.075201386 -12.975175103

C -16.234273223 7.667339394 -13.729028188

H -17.808529698 8.741708368 -11.624947208

H -17.005336782 6.472012236 -12.128780840

H -17.963578284 6.373607062 -13.637001713

H -15.743996334 8.457430914 -13.121931731

H -16.536132718 8.117208996 -14.685584100

C -17.519677612 14.057911343 -14.707063079

C -17.271879373 13.092895615 -15.857174567

C -16.094570218 12.154196959 -15.676105836

O -14.939468733 12.550206333 -15.654306901

N -16.372943818 10.812947443 -15.688254831

H -15.637972142 10.147084568 -15.506010475

H -17.301457289 10.466494599 -15.436205272

H -16.572698096 14.431852914 -14.255187126

H -18.202748741 12.551643535 -16.132636984

H -17.047239764 13.708835338 -16.772044659

C -8.834162734 16.242720861 -16.292599281

C -8.562285872 14.738980178 -16.347592344

C -9.274559739 14.129073063 -17.559142823

C -7.056056115 14.499144323 -16.484351114

H -8.073509687 16.810073264 -16.901575362

H -8.953797060 14.238479933 -15.430524533

H -9.031667051 13.065967944 -17.669236492

H -8.976168397 14.646105686 -18.481313995

H -10.367976137 14.232221716 -17.467723570

H -6.658594439 14.985571761 -17.394911168

H -6.825796016 13.433406235 -16.610895479

H -6.474448152 14.891202008 -15.650104002

C -11.310537842 12.693017679 -21.195409261

C -10.184548647 13.493251835 -21.858008155

O -9.776321841 13.140218838 -22.961703722

C -10.827685504 11.862043634 -20.011125639

C -12.019991239 11.266113690 -19.219968075

C -11.637420730 9.881898696 -18.697068283

C -12.416023730 12.203926455 -18.082365681

H -12.144580400 13.383986547 -20.876539724

H -10.195928804 12.476566796 -19.329285564

H -10.123885883 11.067476096 -20.334000490

H -12.907603993 11.144308374 -19.887555811

H -13.269338826 11.812474854 -17.498912955

H -11.602817850 12.342615572 -17.359353659

H -12.719956092 13.197086950 -18.429791846

H -12.397609530 9.495351319 -17.999475070

H -11.527420347 9.145807375 -19.497448154

H -10.692149326 9.906642848 -18.137329943

N -9.778814206 14.623069550 -21.234362557

H -10.091642723 14.901817888 -20.299511823

C -8.535512648 15.255856659 -21.712081067

C -7.484168994 14.132567083 -21.830348550

O -7.685867142 13.049512449 -21.283078188

C -8.049851971 16.312706296 -20.704234418

C -7.120290300 17.312548705 -21.368248018

H -8.730151795 15.720165444 -22.714779523

H -8.907047493 16.882189818 -20.252211148

H -7.546038210 15.836363389 -19.840024780

H -7.634189007 17.930594758 -22.117785877

H -6.238548922 16.851694770 -21.842542105

N -6.422909865 14.363227207 -22.644742091

H -6.204934173 15.337108154 -22.943229333

C -5.245664438 13.481159146 -22.651379650

C -4.074776900 14.404466728 -22.190875264

O -4.105524415 15.572979174 -22.519841341

C -4.983162826 12.966417214 -24.089268255

C -3.517807519 12.758599253 -24.254600208

C -2.875378858 11.594850112 -23.813645613

C -2.744940021 13.833419508 -24.736106187

C -1.505150409 11.552109159 -23.692224755

C -1.380974630 13.826256155 -24.615059509

C -0.726700181 12.727191784 -23.960943690

O 0.475053138 12.827428731 -23.598488117

H 0.438303437 13.975867074 -22.567139727

H -5.388945612 12.626981520 -21.950633171

H -5.551492483 12.030686867 -24.270264593

H -5.348655992 13.711591814 -24.826730500

H -3.469281095 10.726742838 -23.538668086

H -3.248010641 14.700799263 -25.162645448

H -0.978229163 10.679207661 -23.333686348

H -0.768964305 14.658012421 -24.938026403

N -3.163545922 13.868472940 -21.327985774

C -2.145051418 14.736062879 -20.721021035

C -2.402658560 14.893098457 -19.224311475

S -4.141316309 15.388098804 -18.872697758

H -2.153780548 15.748711544 -21.232154372

H -1.701597927 15.633062309 -18.785725681

H -2.223772849 13.942036657 -18.686192543

H -4.252558734 16.611221009 -19.490530721

H -3.042605359 12.873406802 -21.216726707

C -1.503483403 13.910851248 -14.293632569

C -2.499792689 13.626733223 -15.402055649

C -3.015493942 12.178239795 -15.462347516

C -4.068845881 12.070873230 -16.571767613

C -1.881580332 11.190439555 -15.751676214

H -0.651796657 13.193057643 -14.312723919

H -3.343962642 14.342138541 -15.376034441

H -1.987595852 13.873732617 -16.375030283

H -3.499506761 11.907618580 -14.495902203

H -4.570130311 11.095015158 -16.547521992

H -4.840785996 12.839322068 -16.481418452

H -3.616437561 12.180872711 -17.567147379

H -2.271978428 10.178609073 -15.918063561

H -1.330743784 11.470132814 -16.663654548

H -1.158225627 11.132371164 -14.936167810

C -7.882360159 12.439140325 -12.730783306

C -8.263922415 11.074917239 -12.041234903

O -7.680918853 10.843165244 -11.001792791

C -6.848110635 12.198270012 -13.844225380

O -5.593774724 11.999043135 -13.222782400

H -5.405294161 11.021150087 -13.194066267

H -8.803972283 12.940295971 -13.109239631

H -7.120488681 11.316521012 -14.475892015

H -6.703250800 13.077217245 -14.495663765

N -9.122848695 10.339826813 -12.775881158

H -9.597749110 10.766009288 -13.568926129

C -9.851556866 9.198072223 -12.178371588

C -10.910014582 9.855835069 -11.250794444

O -10.637895721 10.961511173 -10.788935695

C -8.922503859 8.242822182 -11.470231362

H -10.372358612 8.661411858 -13.027029843

H -7.899403837 8.268414758 -11.883915467

H -8.806399264 8.478702367 -10.395771368

N -12.107812596 9.244754995 -11.199526361

C -13.261600740 9.822477263 -10.492198016

C -13.958716908 10.936198340 -11.297610600

C -13.120232253 12.138252843 -11.609337627

C -13.130446241 12.691037349 -12.891859597

C -12.337685792 12.752849316 -10.620985332

C -12.354460114 13.809540899 -13.195840424

C -11.562112025 13.867480548 -10.923494694

C -11.559210796 14.396797463 -12.216366078

H -12.964445434 10.191377793 -9.486805644

H -14.381719955 10.492322179 -12.225482959

H -14.856051020 11.245890757 -10.713157569

H -13.761680553 12.261262971 -13.674210638

H -12.308992328 12.329120520 -9.616233812

H -12.397706197 14.238307442 -14.198267889

H -10.963202095 14.327600053 -10.146331121

H -10.982245727 15.281926823 -12.441175049

H -12.179292086 8.191265030 -11.378115880

C -7.002439298 9.680787257 -17.798508494

C -5.720528176 9.480883929 -18.412256437

C -5.630671381 10.517765450 -19.397286781

N -6.829349568 11.223612988 -19.335013382

N -7.631093197 10.727219055 -18.370232278

C -4.477910957 10.710364140 -20.176742938

C -3.410044956 9.871965026 -19.947407838

C -3.515451698 8.837825907 -18.987928856

C -4.639125510 8.606993818 -18.227992732

H -7.095309273 12.037806752 -19.879707363

Cl -2.156878313 7.842744212 -18.761300140

S -7.553851424 8.843910593 -16.354785282

O -6.785779073 9.502090285 -15.303015286

O -7.402466458 7.422699556 -16.610439124

C -9.238463173 9.158523549 -16.052256996

C -9.737148346 10.453500505 -15.920166392

C -11.037791618 10.653876883 -15.465766069

C -11.862840527 9.559345311 -15.171717759

C -13.214970309 9.735942925 -14.622935197

C -11.373577857 8.256975031 -15.366547727

C -10.073139792 8.062850257 -15.797509664

H -4.429513770 11.511612941 -20.908009398

H -2.467493014 10.011777816 -20.496534210

H -4.701766825 7.790770006 -17.501401341

H -9.121674409 11.320743096 -16.164751808

H -11.428963620 11.662329731 -15.327357373

H -12.055781883 7.421180066 -15.133785440

H -9.686460158 7.049537458 -15.920104280

H -13.699241897 10.672780550 -14.923521756

H -13.191069081 9.704838173 -13.508574431

H -13.886035645 8.870004733 -14.868161198

Azole **2** with surrounded residues

N -6.914000000 2.232000000 -21.285000000

C -6.212000000 3.896000000 -22.850000000

O -4.998000000 3.971000000 -22.680000000

H -6.055000000 1.859000000 -21.750000000

H -7.713000000 1.636000000 -21.558000000

H -6.772000000 2.132000000 -20.264000000

O -6.685000000 4.051000000 -23.984000000

N -7.810000000 11.109000000 -24.650000000

H -8.521000000 11.045000000 -25.396000000

H -6.913000000 11.416000000 -25.075000000

H -8.118000000 11.915000000 -24.006000000

C -12.247000000 3.846000000 -21.763000000

O -13.295000000 4.220000000 -22.292000000

N -10.825000000 4.567000000 -15.042000000

H -10.596000000 3.859000000 -14.319000000

H -11.588000000 5.169000000 -14.621000000

H -9.990000000 5.164000000 -15.165000000

O -12.039000000 2.635000000 -21.608000000

N -19.659000000 7.628000000 -11.972000000

C -15.176000000 6.582000000 -13.982000000

O -14.245000000 6.852000000 -14.766000000

O -15.229000000 5.495000000 -13.377000000

H -20.006000000 6.864000000 -12.583000000

H -19.491000000 7.192000000 -11.031000000

H -20.410000000 8.328000000 -11.867000000

C -20.763000000 10.996000000 -15.182000000

O -20.455000000 12.080000000 -15.689000000

O -21.954000000 10.799000000 -15.461000000

N -20.645000000 12.444000000 -12.393000000

H -20.228000000 11.526000000 -12.626000000

H -20.855000000 12.892000000 -13.359000000

H -21.543000000 12.278000000 -11.929000000

C -18.350000000 15.230000000 -15.328000000

O -17.787000000 16.280000000 -15.632000000

O -19.315000000 15.521000000 -14.608000000

N -8.329000000 19.671000000 -13.192000000

H -8.495000000 20.179000000 -14.116000000

H -8.579000000 20.333000000 -12.434000000

H -7.320000000 19.479000000 -13.142000000

C -12.251000000 16.444000000 -19.334000000

O -11.720000000 15.508000000 -19.927000000

N -10.456000000 22.388000000 -22.019000000

H -9.729000000 23.052000000 -21.699000000

H -11.290000000 22.923000000 -22.296000000

H -10.089000000 21.905000000 -22.861000000

O -13.483000000 16.467000000 -19.211000000

N -11.852000000 11.728000000 -22.235000000

H -11.093000000 11.143000000 -22.678000000

H -12.351000000 12.212000000 -22.991000000

H -12.521000000 11.070000000 -21.752000000

C -6.586000000 18.273000000 -20.262000000

O -7.384000000 19.025000000 -19.657000000

O -5.359000000 18.261000000 -20.035000000

C 1.827000000 16.483000000 -23.218000000

O 2.161000000 17.196000000 -24.174000000

O 1.746000000 16.867000000 -22.043000000

C 2.933000000 11.709000000 -13.173000000

O 4.133000000 11.724000000 -13.433000000

C 0.578000000 7.333000000 -13.943000000

O 1.397000000 6.783000000 -13.176000000

O -0.520000000 6.819000000 -14.264000000

O 2.522000000 12.292000000 -12.160000000

N -2.589000000 11.876000000 -10.338000000

H -1.684000000 11.323000000 -10.477000000

H -2.930000000 11.684000000 -9.383000000

H -3.279000000 11.477000000 -11.013000000

C 0.300000000 16.778000000 -17.475000000

O 0.169000000 15.771000000 -18.165000000

O 0.270000000 17.898000000 -18.004000000

N -6.098000000 16.608000000 -11.595000000

H -6.814000000 16.925000000 -12.290000000

H -5.805000000 17.415000000 -11.008000000

H -5.256000000 16.317000000 -12.158000000

C -9.461000000 6.806000000 -11.586000000

O -8.656000000 5.868000000 -11.368000000

O -10.685000000 6.617000000 -11.771000000

C -14.750000000 5.891000000 -9.015000000

O -15.567000000 5.023000000 -8.743000000

O -14.194000000 6.522000000 -8.106000000

C -7.127900307 3.711474994 -21.611474572

C -6.657539793 4.567423594 -20.423035056

C -6.409480399 6.017281209 -20.853928323

C -7.693664079 4.546237740 -19.293928240

H -8.192002321 3.905526188 -21.876861004

H -5.674739000 4.171973796 -20.052059634

H -7.888395899 3.550836948 -18.900789405

H -7.355559631 5.178126966 -18.453241654

H -8.645751317 4.984329438 -19.628423861

H -7.321552028 6.496604737 -21.220113588

H -5.653026500 6.066765741 -21.653844175

H -6.032925358 6.613330526 -20.012659813

C -7.670745804 9.817512845 -23.855251468

C -9.030628697 9.107230318 -23.986424792

O -10.062180369 9.768935717 -23.929272052

C -7.405909750 10.121461556 -22.398414406

H -6.830019791 9.215521483 -24.300637377

H -8.142488785 10.822596007 -21.963672082

H -7.444261712 9.208519740 -21.773087306

H -6.411999477 10.567147133 -22.211424362

N -8.989060200 7.795037662 -24.296071009

H -8.138416310 7.218702968 -24.172383491

C -10.232666480 7.004011733 -24.250034967

C -10.173463544 6.312237928 -22.873404344

O -9.079457580 6.111528033 -22.370134001

C -10.207764972 5.921882252 -25.361703649

C -11.492146577 5.096704637 -25.320906735

C -10.057269632 6.583489984 -26.735651564

H -11.126587812 7.661669172 -24.361605017

H -9.328194889 5.241464555 -25.183606822

H -11.519028489 4.367731253 -26.140101844

H -12.393383486 5.711082680 -25.400889478

H -11.588948703 4.500249070 -24.395852226

H -10.059967358 5.826963624 -27.530338258

H -9.114479188 7.132026399 -26.821002271

H -10.874259323 7.280369716 -26.942313691

N -11.333784008 5.993346488 -22.247722281

C -11.265811390 4.928377290 -21.226473124

C -11.719301265 5.473878738 -19.867035037

C -11.561856740 4.392731334 -18.790470586

C -11.519644510 5.023807651 -17.394573729

C -11.282595674 3.939872813 -16.340724655

H -10.235946096 4.506173362 -21.155219860

H -12.776961586 5.803541018 -19.928364314

H -11.124800187 6.373591036 -19.618389347

H -10.645443418 3.802347458 -18.980396352

H -12.396767648 3.665438168 -18.884160212

H -10.721216625 5.794818081 -17.371082515

H -12.474734950 5.564123429 -17.195916085

H -12.226931375 3.372363442 -16.151456901

H -10.515134910 3.210125738 -16.678630441

H -12.251421431 6.056209754 -22.704853673

C -18.331844714 8.248923820 -12.477222395

C -18.963179822 9.360127687 -13.326464108

O -19.915618804 9.931856060 -12.756609121

C -17.506788785 7.157695517 -13.149564917

C -16.230474998 7.704447233 -13.767965119

H -17.746191930 8.680433741 -11.607435471

H -17.179150893 6.389151842 -12.389058636

H -18.077980852 6.588911797 -13.907301501

H -16.389834336 8.185562511 -14.743694235

H -15.729887811 8.435056969 -13.090268969

N -18.711737938 9.452252606 -14.623218736

H -17.824708031 9.111088817 -15.026347696

C -19.737836644 9.854438856 -15.569119687

C -20.556371422 8.618073903 -16.053460638

C -19.746471043 7.308313784 -16.104147667

C -21.093207455 8.932399027 -17.456362290

C -20.662299889 6.096629453 -16.287786418

H -19.196072402 10.303278219 -16.457663496

H -21.423780353 8.490090876 -15.362317932

H -19.152256149 7.159817244 -15.185574009

H -19.013114536 7.352941929 -16.934621588

H -21.630900994 9.899933490 -17.456954267

H -21.812221319 8.179957701 -17.787376479

H -20.293518134 8.999589274 -18.199164643

H -20.090253691 5.161714595 -16.269342005

H -21.184068223 6.135321876 -17.251902869

H -21.428747445 6.042780661 -15.510720428

C -19.552692359 13.256219678 -11.724737316

C -18.473286681 13.735211411 -12.798484148

O -17.616934806 14.461935467 -12.359469898

C -18.876146544 12.361723696 -10.660960532

C -19.848853632 12.076585209 -9.495425445

C -17.598058769 13.001607153 -10.119420567

C -19.472890628 10.775867649 -8.784911398

H -19.981338501 14.183838548 -11.282031102

H -18.593791501 11.387542099 -11.142505591

H -19.825355886 12.918418952 -8.776649275

H -20.894668467 12.015897686 -9.837139515

H -16.770478224 12.946863519 -10.846470508

H -17.730886901 14.064826671 -9.886796129

H -17.246382973 12.497121052 -9.212572126

H -20.084182747 10.614809001 -7.891867454

H -18.421944591 10.784603788 -8.455776493

H -19.596865171 9.906490425 -9.436263136

N -18.441878979 13.153781399 -14.053176274

C -17.634066488 13.872831573 -15.090071461

C -17.487351016 13.009356617 -16.336074578

C -16.529614189 11.854885805 -16.190411565

O -15.863050883 11.411365131 -17.110423634

N -16.444838393 11.236356178 -14.952812058

H -15.767504895 10.529203033 -14.765448665

H -16.942116433 11.619691190 -14.171900553

H -16.632268023 14.134259290 -14.656997627

H -18.489260135 12.630579148 -16.666136089

H -17.148046192 13.660789844 -17.177912535

H -19.305529020 12.786932491 -14.504517586

C -9.176123786 18.395870921 -13.195472142

C -9.015886787 17.915691251 -14.670018107

O -9.017323472 18.826606643 -15.488667277

C -10.656492884 18.758221126 -12.943358966

C -11.538620252 17.538260119 -13.177142087

C -10.846496497 19.268633776 -11.514596079

H -8.806592008 17.683695976 -12.435224789

H -10.966857548 19.550221329 -13.676213753

H -11.494818448 17.160199319 -14.219252012

H -11.307459542 16.702802767 -12.507674995

H -12.605751035 17.772655904 -13.022545705

H -10.293560702 20.177681929 -11.284672175

H -10.599585018 18.500935951 -10.769390330

H -11.913359665 19.503350618 -11.341674123

N -8.785044011 16.620775827 -14.925223503

H -8.779235981 15.891586279 -14.214735601

C -8.834416904 16.168363344 -16.337263382

C -10.179721376 16.664536549 -16.908646257

O -11.198994700 16.551733323 -16.254896661

C -8.815038689 14.621477183 -16.386225782

C -9.245100934 14.136928159 -17.769143574

C -7.413370207 14.109010636 -16.083214905

H -7.956217157 16.604263702 -16.886993176

H -9.540687137 14.225264937 -15.629194802

H -9.206917573 13.038454689 -17.809872881

H -8.593217507 14.521394478 -18.561589455

H -10.279222300 14.435053944 -18.014477078

H -6.677074292 14.446404972 -16.830278226

H -7.042751207 14.428925167 -15.106304426

H -7.392951389 13.009649594 -16.103383615

N -10.149224058 17.177241188 -18.170560784

C -11.401834406 17.630556891 -18.782884630

C -11.062119651 18.563096862 -19.956252398

C -12.247255157 19.480410709 -20.246577701

C -11.921736651 20.467655629 -21.380158184

C -10.779157372 21.367512065 -20.926346674

H -12.029458395 18.146370127 -18.007050596

H -10.140962556 19.139417818 -19.716408476

H -10.805589585 17.957624643 -20.850049427

H -12.561900486 20.019448030 -19.332549943

H -13.132784287 18.855361800 -20.518674041

H -11.653228918 19.891643519 -22.288697480

H -12.830790630 21.045500563 -21.629049190

H -9.845466359 20.765146167 -20.708425555

H -11.030083814 21.909009944 -19.988040466

H -9.275579568 17.459062297 -18.622654155

C -11.328090371 12.714846591 -21.195184235

C -10.108058725 13.396545325 -21.816080684

O -9.578720830 12.820929873 -22.775316992

C -10.984007376 11.908926963 -19.950534700

C -12.269370425 11.400675745 -19.248893216

C -12.021395494 9.994601600 -18.704783527

C -12.681975111 12.374194991 -18.147838288

H -12.130115052 13.486892690 -20.945594485

H -10.409181867 12.542886600 -19.236417264

H -10.290357308 11.075758349 -20.176643242

H -13.116565964 11.344273601 -19.976430627

H -12.882009780 9.643505456 -18.116175343

H -11.848426117 9.260596422 -19.495045870

H -11.151268098 9.971911487 -18.032351521

H -13.657974459 12.099401414 -17.713183721

H -11.957265425 12.406603176 -17.328714163

H -12.795892635 13.404837117 -18.524401849

N -9.714353716 14.557389882 -21.274224904

H -10.351448101 15.103308507 -20.551742720

C -8.518694643 15.229920153 -21.795936858

C -7.442946883 14.174778695 -22.076821051

O -7.357074734 13.138157705 -21.440728855

C -7.963747353 16.220299386 -20.745447523

C -7.122181046 17.320157043 -21.372705887

H -8.788812821 15.775808072 -22.736699932

H -7.360930415 15.676439787 -19.983524598

H -8.810972154 16.682361092 -20.199457169

H -6.228599292 16.916685961 -21.893011462

H -7.686327047 17.919454213 -22.094977225

N -6.570744891 14.415705276 -23.123134800

H -6.495530206 15.356139234 -23.519802177

C -5.292772560 13.686231533 -23.086806027

C -4.227816854 14.697298340 -22.556511426

O -4.213620317 15.813348921 -23.040496298

C -4.872038957 13.194589268 -24.488697815

C -3.675196686 12.323052525 -24.260549862

C -3.820148765 11.064746269 -23.668838987

C -2.399171475 12.862727096 -24.455378356

C -2.724625600 10.424604739 -23.105037220

C -1.290543542 12.231686215 -23.916188512

C -1.480968028 11.072038176 -23.150270690

O -0.478062653 10.511877305 -22.443237967

H 0.114608416 11.226637757 -22.040796442

H -5.385525254 12.798183928 -22.402892001

H -4.629374088 14.062198550 -25.139235405

H -5.694170030 12.669413734 -24.996083102

H -4.798781671 10.603690174 -23.601732574

H -2.281197361 13.811303634 -24.981282561

H -2.817015309 9.467186690 -22.597783408

H -0.293419506 12.660286459 -24.028773212

N -3.449863390 14.247829274 -21.546090027

H -3.451294117 13.269207443 -21.265744645

C -2.353983629 15.035763669 -20.954277372

C -1.087469639 14.206333409 -21.293280426

O -1.178157822 12.979056216 -21.343550513

C -2.491075969 15.137221829 -19.439425237

S -4.205849965 15.526779390 -18.919727854

H -2.317628122 16.056790709 -21.427850569

H -2.197008846 14.197950773 -18.927899167

H -1.764045578 15.904605089 -19.041960837

H -4.330304832 16.802154261 -19.383181827

N 0.007068956 14.883400927 -21.689660564

H 0.202147537 15.885905886 -21.468075732

C 1.123392128 14.170379062 -22.318407925

C 2.262920640 13.868746887 -21.321181322

O 2.779466982 12.762678300 -21.361132274

C 1.614705563 14.970455637 -23.520789682

H 0.768443931 13.158064846 -22.667756813

H 2.579749787 14.580198029 -23.885135953

H 0.908782714 14.902031384 -24.361798053

N 2.586844681 14.818849614 -20.405061348

H 2.216054659 15.781037916 -20.490766349

C 3.515011960 14.510488566 -19.322649252

C 2.986170167 13.353085314 -18.457820353

O 3.689798520 12.406610463 -18.157971398

H 3.671744263 15.417349317 -18.697203733

H 4.506579868 14.200054696 -19.731216131

N 1.683272477 13.446399606 -18.039979094

H 1.084941633 14.271502540 -18.244302596

C 1.027393901 12.246703299 -17.552545374

C 1.010371408 12.183073282 -16.015961025

O 0.094887340 12.728586854 -15.399101941

H -0.029989401 12.224276811 -17.910343437

H 1.513900762 11.328558393 -17.967910233

N 1.936103995 11.386834075 -15.433488946

C 1.841792855 11.001965369 -14.021661973

C 1.991107552 9.478732016 -13.853979170

C 0.867987648 8.721483044 -14.553470717

H 0.845630257 11.329345016 -13.625375336

H 2.977966017 9.136707586 -14.217914653

H 1.999683634 9.228794490 -12.773171619

H 1.111407494 8.573949376 -15.618399432

H -0.076346553 9.292238537 -14.524984037

H 2.790979528 11.137111996 -15.923402379

C -2.249393492 13.308838806 -10.732747025

C -2.578472707 13.317646027 -12.253405403

O -2.754493953 12.225447548 -12.764290014

C -0.736354209 13.544415903 -10.669429998

C -0.106731229 13.464049480 -9.273880856

C 1.408341831 13.598780215 -9.439829287

C -0.649396675 14.572656905 -8.359261083

H -2.827543328 14.022645035 -10.128536059

H -0.173236307 12.841394093 -11.359922696

H -0.488658594 14.532529332 -11.122331800

H -0.324834190 12.475368821 -8.808674262

H 1.814931921 12.907517820 -10.229251577

H 1.707858331 14.595757454 -9.777634589

H 1.948706609 13.373299486 -8.520782687

H -0.112570287 14.578142672 -7.403100037

H -1.709188637 14.456136575 -8.138714696

H -0.496286842 15.561670213 -8.805973623

N -2.636506666 14.479810533 -12.942588381

H -2.404451754 15.385289636 -12.541015515

C -2.317503900 14.348027010 -14.399667953

C -1.599055018 15.672724795 -14.737982705

O -1.959645224 16.686980072 -14.126165110

C -3.543711512 14.061611536 -15.262978298

C -3.513759773 12.584311285 -15.726217483

C -4.913385542 12.090324660 -16.087340456

C -2.576189215 12.405363189 -16.920486880

H -1.574985459 13.479484356 -14.521740364

H -4.473306574 14.285868324 -14.723120102

H -3.554864538 14.729720694 -16.151813740

H -3.125196481 11.948300759 -14.887443891

H -4.906664624 11.000458940 -16.236827108

H -5.639123350 12.294877417 -15.301544290

H -5.268733218 12.545408481 -17.018260760

H -2.254185144 11.358791772 -17.005783319

H -3.049666647 12.690943673 -17.863060800

H -1.657302359 13.002039193 -16.802463539

N -0.582353598 15.555847114 -15.592441506

C 0.359802014 16.631169720 -15.920604056

C 1.784526315 16.211701857 -15.512954246

C 1.879342952 15.585681468 -14.108505633

C 3.298480135 15.072260961 -13.860628025

C 1.473870264 16.592560844 -13.019693117

H 0.056707872 17.586437442 -15.442129201

H 2.169569744 15.472280012 -16.251361433

H 2.447587576 17.090652757 -15.588882331

H 1.192297989 14.700929022 -14.054744140

H 1.562786859 16.143502921 -12.027941629

H 0.439726821 16.924139577 -13.157194684

H 2.112479099 17.478701796 -13.045650542

H 3.337324016 14.444456748 -12.950576315

H 3.629391104 14.391052426 -14.658235452

H 4.028940372 15.871495741 -13.761393996

H -0.314867421 14.637369168 -16.000177533

C -6.619591175 15.398281818 -10.817190247

C -6.954522718 14.362536019 -11.945727452

O -7.486436004 14.844109953 -12.935664556

C -7.917632803 15.705818064 -10.054864116

C -7.667526623 16.724503608 -8.927696215

C -8.463069519 14.397288866 -9.482320644

C -8.971198703 17.400993798 -8.509846519

H -5.818082688 15.053581789 -10.126103417

H -8.683156380 16.109329840 -10.766526502

H -6.930524337 17.491028792 -9.213690344

H -7.228097180 16.207049464 -8.047257134

H -8.793311650 13.674680250 -10.261455180

H -7.754777973 13.869758895 -8.837745149

H -9.375143245 14.562447292 -8.887802004

H -8.824013847 18.053506793 -7.641059251

H -9.402294681 18.009159480 -9.309621293

H -9.733165221 16.659379225 -8.218440399

N -6.514265266 13.113481945 -11.763625249

H -6.225040177 12.770114808 -10.842018044

C -6.783894036 12.010643019 -12.704310144

C -7.853822089 11.091062584 -12.031718693

O -8.064469987 11.241714502 -10.843977241

C -5.481669475 11.191888315 -12.836510714

O -5.038679431 10.899519826 -11.518321313

H -5.499259546 10.100355682 -11.175223342

H -7.137226404 12.395849086 -13.693363569

H -4.658162724 11.771947134 -13.302984497

H -5.629087079 10.257473751 -13.427935242

N -8.389912076 10.183415528 -12.879693816

H -7.974965251 10.032639158 -13.812746093

C -9.418023975 9.213472375 -12.480882568

C -10.642401259 9.980232950 -11.937214044

O -10.613713128 11.183306555 -11.769282002

C -8.887727173 8.230139935 -11.447202949

H -9.730587045 8.662469920 -13.415393267

H -7.786176015 8.143331488 -11.513543135

H -9.088280383 8.571874010 -10.413443379

N -11.739534987 9.192723340 -11.721937948

H -11.619645656 8.146814258 -11.827863618

C -12.920933628 9.695769238 -11.012620441

C -14.010465893 8.580809596 -10.947702421

O -15.188798630 8.894528878 -11.140610604

C -13.559077850 10.930818960 -11.688229380

C -12.841051686 12.214459109 -11.414832579

C -12.321222528 12.965525317 -12.477763848

C -12.708651586 12.696538300 -10.109602846

C -11.632536504 14.147676155 -12.231349359

C -12.039893620 13.895190753 -9.868836480

C -11.492815572 14.620563773 -10.925386725

H -12.635428197 9.955594279 -9.955592034

H -14.616127687 11.021681741 -11.337093011

H -13.633046777 10.733815061 -12.777663304

H -12.423704372 12.604368517 -13.499547955

H -13.115169588 12.127171124 -9.275705119

H -11.198730574 14.694212631 -13.063595045

H -11.940605067 14.260797808 -8.852390537

H -10.963213696 15.541865767 -10.729621180

N -13.598755360 7.331721237 -10.680059298

C -14.540528760 6.231032416 -10.514303639

H -15.523251523 6.465554344 -10.967036798

H -14.167500706 5.334357600 -11.057794373

H -12.624420107 7.088579751 -10.487480425

C -4.710935940 9.337725829 -18.192837381

C -3.604358785 9.820097486 -18.892867460

C -3.746006183 10.884839578 -19.846957440

C -4.963223396 11.467422259 -20.086002071

C -6.067500894 11.002462341 -19.356611811

N -7.392425187 11.437201783 -19.315489647

H -7.796184753 12.204002095 -19.829867620

N -8.086262649 10.768976341 -18.383366062

C -7.252449672 9.868958409 -17.802613245

C -5.948388799 9.952570410 -18.393992568

N -2.341179657 9.290971738 -18.650916907

C -2.016160609 8.411284084 -17.589094158

C -0.652088032 8.229408157 -17.587195606

C -0.096945615 9.023869127 -18.648532424

C -1.135208164 9.674974784 -19.280448210

S -7.720920952 8.959031112 -16.401922281

O -7.565221533 7.540550242 -16.657181140

O -7.019722687 9.599326762 -15.279945380

C -9.406624121 9.262333398 -16.080844937

C -10.297214055 8.188439082 -16.034753520

C -11.627034674 8.402159092 -15.710965862

C -12.078723205 9.702059213 -15.445324980

C -11.179920530 10.773408902 -15.465685280

C -13.515156913 9.925090460 -15.182064225

C -9.845896861 10.552811554 -15.789089297

H -4.619809990 8.517574967 -17.478973511

H -2.848639694 11.247639916 -20.373044664

H -5.095785948 12.297001188 -20.778253923

H -2.754550231 8.004346057 -16.928465556

H -0.100944755 7.612469023 -16.890256203

H 0.945450610 9.086897212 -18.889695279

H -1.115084210 10.348407990 -20.117558783

H -9.948137497 7.178077157 -16.253920423

H -12.346698074 7.575903061 -15.641262054

H -11.524204982 11.777276191 -15.224436968

H -9.160594009 11.404236997 -15.807444394

H -13.978020553 9.019866472 -14.721356953

H -13.698224937 10.789035053 -14.527730407

H -14.063502898 10.100444461 -16.128336763

Azole **3** with surrounded residues

C -2.023647278 4.918781407 -22.123008320

C -3.015870129 6.040979971 -21.880871150

C -3.076649051 7.057972529 -23.033500533

C -4.327914234 7.939455496 -22.875912807

C -1.822417596 7.935851949 -23.035059596

H -1.065182778 5.311609052 -22.537510208

H -4.023391235 5.627387555 -21.658795575

H -2.725499574 6.566988677 -20.936054963

H -3.150976653 6.515428119 -24.002603214

H -1.883935357 8.736458023 -23.779037375

H -0.917187102 7.358689651 -23.243532606

H -1.665782256 8.409479765 -22.051091796

H -4.377846225 8.687699742 -23.669868565

H -5.236299466 7.330749252 -22.914580132

H -4.307820556 8.461768051 -21.909481503

C -7.118131482 3.703871111 -21.617324971

C -6.629107819 4.570812884 -20.437655856

C -6.798805877 6.049635218 -20.796148254

C -7.402603836 4.273179598 -19.151031337

H -8.186801857 3.909283225 -21.873710110

H -5.528651671 4.378557745 -20.273238093

H -7.126692878 3.324861476 -18.688272266

H -8.485985846 4.313059011 -19.302327744

H -7.174891453 5.050985605 -18.391972829

H -7.851096449 6.358577477 -20.760621660

H -6.428077020 6.263930107 -21.805982345

H -6.241898813 6.684383067 -20.092926836

C -7.644775943 9.790690922 -23.908648680

C -9.041619607 9.145238301 -23.917447162

O -10.025040715 9.834395748 -23.664847344

C -7.201773296 10.033582027 -22.484777045

H -6.882388073 9.169868826 -24.459404064

H -7.800344059 10.806881111 -21.968488492

H -7.296547718 9.121923287 -21.861937551

H -6.141625393 10.333658755 -22.405211833

N -9.116078337 7.866918478 -24.345021048

H -8.296274873 7.238550009 -24.350567677

C -10.398195836 7.144683246 -24.224974038

C -10.237793558 6.317358802 -22.931091121

O -9.108072900 6.029337990 -22.566165981

C -10.586646643 6.188817618 -25.430599119

C -11.920235971 5.453242371 -25.313053043

C -10.544309875 6.981733902 -26.741538448

H -11.250028924 7.863131274 -24.163460771

H -9.753295534 5.436495086 -25.428627835

H -10.709313348 6.319188701 -27.600541929

H -11.319722146 7.753203978 -26.772425755

H -9.577409115 7.469404992 -26.892493782

H -12.104419584 4.822602349 -26.191460065

H -11.958179137 4.764601558 -24.447367871

H -12.772408188 6.132254581 -25.217059182

N -11.351481778 6.016196666 -22.222051648

C -11.260000873 4.928246674 -21.227279117

C -11.705226006 5.458841878 -19.858885798

C -11.508692969 4.384110811 -18.784582226

C -11.543378429 5.011490956 -17.387677573

C -11.280817987 3.933455473 -16.333715289

H -10.227567508 4.514839452 -21.177260336

H -12.772322125 5.762499531 -19.902695146

H -11.128477900 6.370979126 -19.613806816

H -12.295745805 3.607267630 -18.901935089

H -10.553176129 3.853149428 -18.951444003

H -12.532995221 5.498065961 -17.211647215

H -10.794971640 5.828346770 -17.333218279

H -12.217199238 3.353496678 -16.137151988

H -10.507034502 3.212833386 -16.675491776

H -12.298536333 6.126001639 -22.606574573

C -18.322986931 8.202862068 -12.467201598

C -17.425785021 7.076476223 -12.973369321

C -16.232634498 7.669064924 -13.725858137

H -17.807520293 8.743711157 -11.624257487

H -17.003348415 6.474145510 -12.126572621

H -17.962192719 6.375193312 -13.635249140

H -15.739199915 8.454722377 -13.114760770

H -16.533009378 8.123295615 -14.680356380

C -17.522432407 14.057190464 -14.710560363

C -17.283743350 13.091353658 -15.861934312

C -16.100697164 12.158859384 -15.682981692

O -14.950857961 12.566654324 -15.656295498

N -16.372143009 10.815977729 -15.699400110

H -15.632535435 10.153925027 -15.516176229

H -17.297488750 10.464767287 -15.447985221

H -16.571345989 14.427080623 -14.262207956

H -18.214461285 12.547294429 -16.129218604

H -17.066842427 13.704854916 -16.779809755

C -8.837179621 16.237412320 -16.292118077

C -8.577763849 14.730503371 -16.334409553

C -9.208667189 14.133504934 -17.596641493

C -7.068721175 14.472158498 -16.362325866

H -8.070237794 16.793287898 -16.902628909

H -9.042053523 14.232249040 -15.449980756

H -8.973916263 13.066522488 -17.690568154

H -8.834278989 14.642797162 -18.495129703

H -10.303350854 14.252212337 -17.583392412

H -6.597477219 14.960950670 -17.235608805

H -6.845623084 13.403787305 -16.486557313

H -6.545831255 14.842770692 -15.481471393

C -11.294912316 12.685439114 -21.196364945

C -10.182676759 13.496413745 -21.868783931

O -9.782874266 13.149584136 -22.977786181

C -10.768632949 11.838935130 -20.041521570

C -11.927387850 11.190877207 -19.244323161

C -11.474694485 9.825916317 -18.727502167

C -12.355166552 12.112821709 -18.105174414

H -12.124641825 13.363563144 -20.845933782

H -10.138791533 12.452298213 -19.355587801

H -10.047767651 11.072694799 -20.396054493

H -12.813552764 11.027842596 -19.904958700

H -12.188290176 9.417206211 -17.994851328

H -11.369585815 9.088252438 -19.527198753

H -10.506110215 9.890922569 -18.210401700

H -13.172425023 11.676989130 -17.503889957

H -12.719173552 13.085105005 -18.452354565

H -11.536215683 12.302158716 -17.399195919

N -9.770520008 14.620911317 -21.239829394

H -10.076769544 14.891319909 -20.299910720

C -8.516953395 15.240420607 -21.709271174

C -7.478317338 14.102949226 -21.816059051

O -7.695674732 13.026889862 -21.263616938

C -8.029570600 16.295444443 -20.700182454

C -7.117888490 17.309285441 -21.367675841

H -8.698452740 15.702973169 -22.714501166

H -7.508616393 15.818421717 -19.845723749

H -8.886558943 16.853107586 -20.234163853

H -7.644498707 17.921835814 -22.112066157

H -6.235111787 16.857967564 -21.849573432

N -6.409508774 14.317697805 -22.626066087

H -6.180948750 15.287042944 -22.929374817

C -5.234798335 13.430208116 -22.608868316

C -4.064330548 14.362649868 -22.168439085

O -4.086717788 15.519754147 -22.540445994

C -4.968887097 12.886527214 -24.035789713

C -3.500857468 12.703802428 -24.212013218

C -2.832288222 11.564703583 -23.742787448

C -2.752984461 13.775307274 -24.734747983

C -1.460624801 11.547028414 -23.642833957

C -1.385897410 13.791552709 -24.638647293

C -0.704682608 12.723685528 -23.962783039

O 0.502585962 12.851350886 -23.625289364

H 0.438357836 13.984372740 -22.570312896

H -5.381077787 12.593035923 -21.888988387

H -5.516469211 11.934510353 -24.189181451

H -5.353478441 13.607564415 -24.786756721

H -3.408023930 10.697455149 -23.426812893

H -3.275233731 14.624475145 -25.173690007

H -0.916256003 10.695749831 -23.254094340

H -0.793542059 14.622826335 -24.995066187

N -3.162493244 13.852724789 -21.284175219

C -2.140849196 14.734309292 -20.703562636

C -2.377219512 14.900715256 -19.204217260

S -4.111775561 15.398921660 -18.836989671

H -2.163658905 15.741368128 -21.223614861

H -2.193300671 13.951887867 -18.662153187

H -1.670059077 15.640980404 -18.776470238

H -4.224259268 16.615778593 -19.460249883

H -3.069027345 12.863861078 -21.104010111

C 1.885161179 10.957446254 -14.036656404

C 1.763806973 9.507345680 -13.599568014

C 0.896195932 8.716878391 -14.563932785

H 0.908701325 11.486314922 -13.979240589

H 2.752961725 9.015769220 -13.473150950

H 1.342180414 9.458010874 -12.569668944

H 1.388430798 8.499197848 -15.523665763

H -0.066991347 9.208441074 -14.767643858

C -1.501695819 13.911932179 -14.289669701

C -2.488545976 13.611731929 -15.401848360

C -3.034256465 12.172620172 -15.418873364

C -4.052412480 12.039897584 -16.557483396

C -1.915532833 11.147028425 -15.618061611

H -0.641329459 13.200455268 -14.293655210

H -3.319331274 14.342397297 -15.405483167

H -1.964562518 13.819253223 -16.374022395

H -3.558667401 11.957010744 -14.459026894

H -2.326361392 10.131523904 -15.721816701

H -1.346124575 11.349815155 -16.534092247

H -1.222896988 11.119987218 -14.771641266

H -4.565273228 11.069795787 -16.517165366

H -3.568185451 12.109233050 -17.540952317

H -4.816377688 12.819280991 -16.521319247

C -7.842241048 12.411617430 -12.729675345

C -8.295888641 11.090028330 -12.003694756

O -7.785511461 10.894453559 -10.919888799

C -6.744682583 12.071366281 -13.760752825

O -5.510515814 12.016252927 -13.071502278

H -5.228256560 11.065496362 -12.982412656

H -8.716221017 12.916493236 -13.195695231

H -6.951875517 11.097549731 -14.277012540

H -6.610969963 12.859033277 -14.521292281

N -9.108856809 10.330952372 -12.766244467

H -9.530307867 10.724218945 -13.602513217

C -9.856910159 9.201747898 -12.173452048

C -10.924499787 9.875535882 -11.270549431

O -10.675394027 10.998927720 -10.839620499

C -8.936202436 8.245803308 -11.451727345

H -10.371787160 8.661467953 -13.023457311

H -7.904387553 8.279974838 -11.846221072

H -8.842890691 8.476168679 -10.374209018

N -12.114229784 9.249629038 -11.199627589

C -13.267142782 9.823111837 -10.489682820

C -13.978183013 10.935014810 -11.284974112

C -13.169063980 12.164296296 -11.563664468

C -13.198744770 12.751717183 -12.830584799

C -12.402269731 12.771985806 -10.559200551

C -12.463736908 13.905867945 -13.100558265

C -11.670169024 13.924200468 -10.827370849

C -11.693536956 14.494727623 -12.102222298

H -12.967175560 10.190999742 -9.484325607

H -14.377781087 10.497155023 -12.225990910

H -14.891009755 11.211750228 -10.707882771

H -13.808658081 12.318700418 -13.627300436

H -12.349952058 12.314678420 -9.570739257

H -12.514123969 14.356179109 -14.093009482

H -11.081946523 14.379298491 -10.039018904

H -11.149276036 15.406963151 -12.300351256

H -12.174221272 8.194262516 -11.369013015

C -7.030795370 9.613530605 -17.765784981

C -5.704475926 9.443215677 -18.289289807

C -5.609369201 10.403176591 -19.343618169

N -6.845092388 11.051306637 -19.397565548

N -7.674879859 10.589373966 -18.438025847

C -4.429818251 10.561894269 -20.091153984

C -3.353276215 9.778939197 -19.752669585

C -3.436561643 8.820663202 -18.690016846

C -4.611415340 8.632180936 -17.971857750

H -7.093731492 11.855615192 -19.956478351

S -7.611687061 8.780084671 -16.339610445

O -7.530833219 7.354925870 -16.607333369

O -6.844193729 9.403444420 -15.270215824

C -9.291032595 9.143255535 -16.039788463

C -9.761349628 10.448100710 -15.913437172

C -11.056709175 10.676194213 -15.454449374

C -11.900501485 9.598633106 -15.155499799

C -11.440496896 8.286160708 -15.344969272

C -13.248274726 9.800678055 -14.600757740

C -10.145089210 8.065514428 -15.777961309

N -2.302960861 8.067387058 -18.366679651

C -2.011379786 7.364242197 -17.181995510

C -0.850205620 6.657809802 -17.418576469

C -0.487434313 6.939448692 -18.785586663

N -1.362668168 7.794530274 -19.338538931

H -4.369326233 11.291611715 -20.891568899

H -2.393539865 9.877578901 -20.284461026

H -4.688613259 7.877436076 -17.184821099

H -9.128034080 11.300457548 -16.162812030

H -11.425284430 11.691942693 -15.314144611

H -12.135667225 7.462556467 -15.103722399

H -9.779075527 7.043948001 -15.894003803

H -2.601612993 7.444042073 -16.287311780

H -0.324154150 6.027866667 -16.720277401

H 0.329311353 6.509155974 -19.344801405

H -13.715750618 10.744614926 -14.904701228

H -13.930022492 8.945520761 -14.838559893

H -13.212492704 9.780110852 -13.486463329

N -2.564000000 3.856000000 -23.064000000

C -1.721000000 4.207000000 -20.769000000

O -0.655000000 4.475000000 -20.213000000

H -1.950000000 3.731000000 -23.863000000

H -2.684000000 2.946000000 -22.563000000

H -3.563000000 4.095000000 -23.392000000

O -2.504000000 3.408000000 -20.237000000

N -6.914000000 2.232000000 -21.285000000

C -6.212000000 3.896000000 -22.850000000

O -4.998000000 3.971000000 -22.680000000

H -5.887000000 2.027000000 -21.324000000

H -7.228000000 2.050000000 -20.312000000

H -7.412000000 1.580000000 -21.914000000

O -6.685000000 4.051000000 -23.984000000

N -7.810000000 11.109000000 -24.650000000

H -8.530000000 11.727000000 -24.166000000

H -6.925000000 11.669000000 -24.650000000

H -8.118000000 10.962000000 -25.620000000

C -12.247000000 3.846000000 -21.763000000

O -13.295000000 4.220000000 -22.292000000

N -10.825000000 4.567000000 -15.042000000

H -10.614000000 3.860000000 -14.312000000

H -11.591000000 5.182000000 -14.624000000

H -9.983000000 5.155000000 -15.157000000

O -12.039000000 2.635000000 -21.608000000

N -19.659000000 7.628000000 -11.972000000

C -18.674000000 9.165000000 -13.615000000

O -17.964000000 10.150000000 -13.771000000

C -15.176000000 6.582000000 -13.982000000

O -14.245000000 6.852000000 -14.766000000

O -15.229000000 5.495000000 -13.377000000

H -20.310000000 7.500000000 -12.786000000

O -19.607000000 8.949000000 -14.400000000

H -20.125000000 8.238000000 -11.291000000

H -19.490000000 6.707000000 -11.529000000

N -18.408000000 13.436000000 -13.639000000

C -18.350000000 15.230000000 -15.328000000

O -17.787000000 16.280000000 -15.632000000

H -19.418000000 13.613000000 -13.873000000

H -18.229000000 13.768000000 -12.685000000

H -18.256000000 12.388000000 -13.660000000

O -19.552000000 15.081000000 -15.588000000

N -8.842000000 16.763000000 -14.873000000

C -10.236000000 16.661000000 -16.839000000

O -11.274000000 16.583000000 -16.184000000

H -9.837000000 16.792000000 -14.500000000

H -8.264000000 16.197000000 -14.250000000

H -8.485000000 17.738000000 -14.865000000

O -10.277000000 17.078000000 -18.005000000

N -11.852000000 11.728000000 -22.235000000

H -11.112000000 11.068000000 -22.606000000

H -12.592000000 11.136000000 -21.772000000

H -12.264000000 12.202000000 -23.051000000

C -6.586000000 18.273000000 -20.262000000

O -7.384000000 19.025000000 -19.657000000

O -5.359000000 18.261000000 -20.035000000

C -0.785000000 14.060000000 -21.008000000

O -0.324000000 13.047000000 -20.490000000

O -0.141000000 14.634000000 -21.897000000

N 2.386000000 11.001000000 -15.462000000

C 2.933000000 11.709000000 -13.173000000

O 4.133000000 11.724000000 -13.433000000

C 0.578000000 7.333000000 -13.943000000

O 1.397000000 6.783000000 -13.176000000

O -0.520000000 6.819000000 -14.264000000

H 3.422000000 10.843000000 -15.477000000

O 2.522000000 12.292000000 -12.160000000

H 1.922000000 10.271000000 -16.034000000

H 2.214000000 11.925000000 -15.898000000

N -2.154000000 13.882000000 -12.921000000

C -0.940000000 15.358000000 -14.520000000

O -1.238000000 16.336000000 -13.849000000

H -2.300000000 14.869000000 -12.570000000

O -0.166000000 15.495000000 -15.477000000

H -1.547000000 13.400000000 -12.230000000

H -3.055000000 13.393000000 -12.956000000

N -7.232000000 13.290000000 -11.662000000

H -7.081000000 12.757000000 -10.747000000

H -6.296000000 13.566000000 -12.000000000

H -7.780000000 14.132000000 -11.444000000

C -9.461000000 6.806000000 -11.586000000

O -8.656000000 5.868000000 -11.368000000

O -10.685000000 6.617000000 -11.771000000

C -14.265000000 8.614000000 -10.320000000

O -15.444000000 8.727000000 -9.974000000

O -13.806000000 7.488000000 -10.558000000

Azole **4** with surrounded residues

N -2.564000000 3.856000000 -23.064000000

H -3.691000000 3.907000000 -23.160000000

H -2.321000000 2.892000000 -22.784000000

H -2.184000000 4.018000000 -24.002000000

C -3.507000000 1.800000000 -18.602000000

O -4.587000000 2.270000000 -18.248000000

O -3.357000000 0.571000000 -18.640000000

N -6.914000000 2.232000000 -21.285000000

H -5.933000000 2.096000000 -20.933000000

C -6.212000000 3.896000000 -22.850000000

O -4.998000000 3.971000000 -22.680000000

H -7.073000000 1.583000000 -22.073000000

H -7.542000000 1.951000000 -20.502000000

O -6.685000000 4.051000000 -23.984000000

N -7.810000000 11.109000000 -24.650000000

H -8.594000000 11.695000000 -24.187000000

H -8.049000000 10.967000000 -25.638000000

H -6.960000000 11.709000000 -24.583000000

C -12.247000000 3.846000000 -21.763000000

O -13.295000000 4.220000000 -22.292000000

N -10.825000000 4.567000000 -15.042000000

H -10.593000000 3.855000000 -14.319000000

H -11.583000000 5.170000000 -14.615000000

H -9.986000000 5.165000000 -15.165000000

O -12.039000000 2.635000000 -21.608000000

N -19.659000000 7.628000000 -11.972000000

H -20.002000000 6.852000000 -12.562000000

C -15.176000000 6.582000000 -13.982000000

O -14.245000000 6.852000000 -14.766000000

O -15.229000000 5.495000000 -13.377000000

H -19.486000000 7.214000000 -11.016000000

H -20.408000000 8.332000000 -11.878000000

C -18.350000000 15.230000000 -15.328000000

O -17.787000000 16.280000000 -15.632000000

O -19.552000000 15.081000000 -15.588000000

N -8.842000000 16.763000000 -14.873000000

H -8.249000000 16.211000000 -14.253000000

C -10.236000000 16.661000000 -16.839000000

O -11.274000000 16.583000000 -16.184000000

O -10.277000000 17.078000000 -18.005000000

H -9.837000000 16.774000000 -14.495000000

H -8.506000000 17.746000000 -14.867000000

N -15.343000000 6.524000000 -22.559000000

H -14.419000000 5.937000000 -22.454000000

H -15.513000000 6.620000000 -23.572000000

H -16.078000000 5.907000000 -22.155000000

C -6.586000000 18.273000000 -20.262000000

O -7.384000000 19.025000000 -19.657000000

O -5.359000000 18.261000000 -20.035000000

C 2.933000000 11.709000000 -13.173000000

O 4.133000000 11.724000000 -13.433000000

C 0.578000000 7.333000000 -13.943000000

O 1.397000000 6.783000000 -13.176000000

O -0.520000000 6.819000000 -14.264000000

O 2.522000000 12.292000000 -12.160000000

N -2.154000000 13.882000000 -12.921000000

H -3.106000000 14.248000000 -12.952000000

C -0.940000000 15.358000000 -14.520000000

O -1.238000000 16.336000000 -13.849000000

H -1.621000000 14.468000000 -12.231000000

H -2.180000000 12.903000000 -12.582000000

O -0.166000000 15.495000000 -15.477000000

N -7.232000000 13.290000000 -11.662000000

H -6.608000000 12.705000000 -11.032000000

H -6.644000000 13.981000000 -12.146000000

H -7.898000000 13.778000000 -11.034000000

C -9.461000000 6.806000000 -11.586000000

O -8.656000000 5.868000000 -11.368000000

O -10.685000000 6.617000000 -11.771000000

C -14.750000000 5.891000000 -9.015000000

O -15.567000000 5.023000000 -8.743000000

O -14.194000000 6.522000000 -8.106000000

C -2.051529782 4.793169026 -21.966385085

C -1.769846969 3.777708880 -20.814152662

O -0.994410915 2.881322139 -21.140009018

C -3.035790528 5.903251488 -21.659219354

C -3.077053219 6.991777743 -22.749567905

C -4.294795678 7.897558179 -22.505075299

C -1.797939351 7.832625765 -22.718674256

H -1.064568551 5.220246006 -22.282415011

H -4.060319156 5.499588525 -21.496517789

H -2.755986859 6.391697347 -20.690760648

H -3.187850815 6.513714887 -23.748476598

H -4.349526230 8.695513665 -23.247213190

H -5.224805197 7.322129936 -22.544387021

H -4.231515992 8.365273246 -21.510232960

H -1.860364194 8.694447993 -23.388904093

H -1.613879669 8.228494499 -21.702261784

H -0.913991111 7.258101473 -23.003658682

N -2.426415476 3.812103488 -19.644343247

H -3.039150268 4.562109398 -19.360698178

C -2.245791876 2.709889977 -18.694892521

H -1.375376336 2.073872722 -18.971332988

H -2.057997973 3.120798605 -17.675449123

C -7.109354739 3.691316418 -21.621961839

C -6.653654846 4.575094825 -20.436297086

C -7.109898209 6.014591828 -20.685973130

C -7.194435855 4.072026568 -19.098987770

H -8.180509249 3.882463962 -21.892164636

H -5.529256420 4.558245913 -20.389316004

H -8.197446984 6.118287492 -20.581474176

H -6.643415050 6.701475349 -19.966513700

H -6.847947263 6.350282293 -21.694755991

H -7.138514265 4.868671009 -18.336128764

H -6.561581881 3.265611898 -18.672346135

H -8.235961182 3.758625480 -19.141201964

C -7.619718284 9.773389964 -23.956091835

C -8.951365062 9.039499346 -24.188833700

O -9.972392779 9.707399249 -24.310252392

C -7.384971748 9.977934487 -22.475767753

H -6.753452353 9.236168520 -24.427633166

H -8.204620739 10.530961673 -21.983896470

H -7.285909510 9.020317087 -21.933520621

H -6.465048133 10.550200445 -22.255206486

N -8.934588339 7.701569298 -24.349006327

H -8.120011841 7.101090457 -24.161936030

C -10.226326219 6.988574680 -24.313414372

C -10.110294188 6.117875450 -23.052819678

O -9.023692474 5.652701522 -22.750872984

C -10.413549689 6.073121177 -25.546520478

C -11.776787613 5.385198397 -25.448726077

C -10.328581007 6.898170174 -26.833579243

H -11.082556767 7.715157651 -24.250111356

H -9.604558938 5.296243386 -25.551496426

H -11.983654624 4.785608120 -26.341681189

H -12.592633734 6.106515471 -25.329631002

H -11.833358747 4.689102852 -24.594168746

H -10.531939916 6.274429802 -27.711626358

H -9.332088201 7.331139162 -26.966826385

H -11.052417740 7.719777919 -26.836074384

N -11.213789835 5.975637442 -22.268367278

H -12.125459103 6.288489015 -22.580974055

C -11.205099838 4.887088475 -21.279587817

C -11.509572806 5.460343663 -19.891438497

C -11.401062083 4.376322225 -18.814806005

C -11.274572737 5.026663362 -17.434448494

C -11.265246312 3.950983665 -16.346715862

H -10.200291199 4.385517031 -21.261711591

H -10.794818986 6.285048610 -19.681997036

H -12.517567249 5.915058082 -19.868937509

H -12.282681807 3.705977990 -18.872441457

H -10.530967009 3.721818555 -19.019609388

H -10.340471244 5.638772255 -17.404212358

H -12.114859441 5.736539224 -17.268412599

H -10.572348895 3.120057430 -16.610889225

H -12.282556451 3.510043049 -16.215713732

C -18.348328197 8.194290742 -12.551345221

C -18.743517297 9.066725614 -13.749279892

O -18.649811571 10.265918630 -13.687421870

C -17.394512505 7.072209807 -12.930838574

C -16.290200739 7.628681021 -13.823583155

H -17.849534514 8.852269447 -11.764683201

H -16.876938018 6.642754150 -12.016940799

H -17.882283562 6.212524028 -13.416394358

H -15.802913968 8.522478734 -13.369017348

H -16.632826862 7.937538589 -14.820072504

N -19.371946908 8.418196095 -14.810614701

H -19.190067356 7.428999564 -14.962412061

C -19.587600094 9.196946221 -16.050136605

C -20.885111764 10.007829998 -15.981326628

O -21.192050803 10.805422965 -16.838841410

C -19.577389770 8.241483669 -17.266272496

C -18.184861714 7.589634618 -17.402688036

C -19.917972022 8.999407534 -18.550828916

C -18.297576769 6.124369977 -17.812227521

H -18.743369495 9.968475746 -16.172833611

H -20.347747049 7.449845279 -17.105844193

H -17.590494341 7.673674409 -16.466442082

H -17.573988744 8.157405672 -18.131985825

H -20.977467786 9.284807852 -18.591903696

H -19.346927052 9.937216629 -18.629050413

H -19.708587497 8.396022741 -19.437105475

H -17.305426781 5.656861250 -17.875798963

H -18.871288245 5.541585561 -17.083179389

H -18.786674337 6.011530808 -18.783614519

N -21.696706703 9.859079594 -14.862702976

H -21.492430149 9.167770612 -14.170072620

C -22.659961699 10.901405628 -14.507661449

C -22.204484897 11.603461306 -13.192810439

O -23.008171917 11.945799754 -12.350860287

C -24.053056287 10.318817033 -14.292438770

S -24.685015320 9.426869200 -15.771060669

H -22.676929245 11.696819420 -15.317219683

H -24.763352593 11.133337267 -14.029467041

H -24.076514147 9.618284290 -13.438931407

H -23.775227843 9.819964531 -16.680179647

N -20.855490243 11.765791093 -13.076108735

H -20.225657760 11.453746601 -13.816072193

C -20.198396459 12.447691667 -11.953275048

C -18.866483396 13.054408957 -12.498000312

O -17.854921275 13.037915738 -11.813069251

C -19.840721333 11.465938787 -10.797741397

C -20.970772678 10.453493435 -10.531171566

C -19.550949974 12.292085859 -9.541854397

C -20.608803741 9.475516300 -9.413637964

H -20.861685554 13.264969199 -11.570288552

H -18.909924986 10.913907376 -11.085013913

H -21.899308608 11.006709626 -10.271916405

H -21.228043993 9.905605571 -11.454632284

H -18.768055555 13.044882544 -9.742402423

H -19.168243613 11.666261163 -8.728992740

H -20.437913048 12.816366416 -9.178285849

H -21.281992826 8.617324525 -9.382321825

H -20.697163933 9.966544183 -8.431660011

H -19.567171109 9.137000281 -9.472801957

N -18.893958338 13.499355855 -13.775391467

H -19.779381138 13.689439249 -14.289928815

C -17.724767829 14.053230119 -14.480596629

C -17.173215639 13.036987334 -15.489883274

C -16.811738016 11.675139615 -14.956267146

O -16.880432931 10.680295161 -15.678244619

N -16.281086437 11.566041665 -13.696908369

H -16.092307431 10.659892230 -13.300612703

H -16.422731078 12.305114589 -13.017063072

H -16.953736915 14.429265596 -13.777250426

H -17.921144313 12.901445719 -16.307622039

H -16.284538799 13.484019598 -15.990388309

C -8.855732180 16.200317071 -16.279571724

C -8.744828381 14.672233949 -16.304935078

C -9.410713633 14.138969662 -17.582341930

C -7.276211758 14.245041473 -16.299917763

H -8.050454141 16.681500475 -16.891320036

H -9.280306210 14.233679084 -15.428210947

H -9.213820765 13.072531922 -17.725832273

H -10.502843041 14.284093749 -17.550160109

H -9.047715248 14.677815176 -18.468009155

H -6.770619376 14.566450751 -17.229273787

H -7.177647409 13.153443577 -16.280993210

H -6.703508055 14.673760478 -15.477462714

C -15.140221766 7.871866355 -21.899611887

C -14.145845615 8.566400838 -22.859958548

O -13.327443859 7.863511924 -23.434328206

C -14.432355694 7.759435388 -20.550333214

C -15.225433803 7.040943883 -19.440598068

C -15.260577270 5.524535361 -19.655448184

C -14.571299239 7.382300905 -18.098765788

H -16.120596173 8.397026268 -21.814100575

H -13.437320708 7.269005130 -20.669410966

H -14.186148909 8.791128086 -20.197771341

H -16.272991536 7.431425049 -19.428604006

H -15.043874244 6.859743324 -17.245724708

H -14.647784166 8.449754755 -17.852413348

H -13.512711656 7.107176659 -18.061982092

H -16.159217550 5.188573421 -20.174844331

H -15.248835765 4.988935502 -18.691882984

H -14.385013664 5.134728121 -20.198022967

N -14.234691713 9.901602373 -23.049036222

H -14.897712698 10.510872053 -22.577394674

C -13.337899974 10.545719834 -24.029214766

C -12.830089657 11.788983491 -23.273623452

O -13.612568640 12.620034508 -22.861026308

C -14.118827256 10.924358938 -25.298973864

C -14.845913240 9.712362291 -25.798228955

C -16.236686684 9.638114459 -25.699818341

C -14.127836622 8.619895032 -26.293185479

C -16.900260235 8.468584552 -26.064970190

C -14.793599160 7.452274646 -26.655130948

C -16.180167604 7.371360013 -26.532137761

H -12.493441325 9.845585252 -24.297370941

H -14.813572201 11.767637717 -25.087282613

H -13.420478721 11.317872649 -26.066929433

H -16.806156821 10.493789882 -25.339735841

H -13.042237375 8.673005943 -26.376810619

H -17.985247142 8.417527281 -25.994730411

H -14.228836552 6.599982801 -27.034129657

H -16.699527588 6.460169538 -26.821096969

N -11.489952188 11.809646145 -22.995242954

H -10.852321955 11.104290414 -23.348910412

C -11.019276911 12.576548310 -21.842041151

C -9.750196907 13.320326836 -22.261905696

O -9.052143350 12.959756828 -23.213907526

C -10.675479153 11.623741835 -20.674855135

C -11.875073587 10.741826477 -20.265540801

C -11.366383870 9.529732634 -19.481229236

C -12.875402564 11.544787592 -19.433033924

H -11.820470565 13.309598303 -21.522292994

H -9.822468978 10.975136621 -20.951305769

H -10.327441930 12.204980866 -19.795366818

H -12.386706240 10.365892186 -21.181201291

H -12.188080347 8.940788913 -19.061004956

H -10.759304835 8.866395828 -20.103522598

H -10.742460534 9.848088368 -18.631124739

H -13.734733731 10.935227643 -19.134973703

H -12.417621739 11.919502470 -18.504912239

H -13.264725122 12.410348438 -19.983275863

N -9.393423562 14.414040099 -21.546407440

H -9.891472478 14.736617620 -20.716260800

C -8.119061497 15.080111649 -21.855051837

C -7.001582471 14.023874953 -21.827318070

O -7.058523404 13.055315857 -21.087910412

C -7.800377979 16.147182279 -20.782726572

C -7.085873353 17.340772989 -21.395545739

H -8.199067613 15.548060985 -22.872149022

H -8.730475110 16.519596999 -20.280288195

H -7.193271175 15.713975855 -19.961035390

H -6.205881222 17.047700573 -21.994290965

H -7.754122383 17.929959834 -22.043362977

N -5.905066518 14.187149225 -22.655656042

H -5.826900412 15.052361605 -23.208004802

C -4.597119023 13.744941275 -22.112943371

C -3.946114435 15.080924439 -21.658188402

O -4.014087828 16.031839499 -22.416202936

C -3.719374677 13.074473355 -23.183056051

C -2.405658075 12.724178398 -22.548382622

C -2.171524564 11.449554220 -22.030496867

C -1.428758259 13.720690055 -22.415806575

C -1.006613303 11.176413220 -21.322379492

C -0.267109142 13.476075034 -21.703792575

C -0.072212714 12.206527493 -21.139978931

O 1.017160808 11.921979596 -20.414903189

H 1.477568297 12.774839650 -20.051006236

H -4.750390161 13.038894664 -21.255876406

H -4.226930587 12.185842902 -23.592795784

H -3.570817883 13.761114502 -24.041881245

H -2.910465380 10.663399952 -22.167826450

H -1.595707642 14.713818798 -22.843021633

H -0.810319680 10.194952167 -20.894919846

H 0.474499085 14.262238098 -21.555901863

N -3.510111213 15.159941237 -20.369707921

H -3.237672745 14.344589274 -19.841066500

C -3.119157630 16.471560925 -19.848029024

C -1.749695450 16.868017740 -20.403654408

O -1.010372156 16.071748130 -20.959992105

C -3.053474628 16.440073670 -18.320890273

S -4.705441342 16.653766646 -17.543671224

H -3.894128746 17.241819718 -20.239211395

H -2.388480557 17.239703280 -17.927825241

H -2.618919020 15.494076962 -17.939197873

H -5.194593301 17.653142366 -18.380499392

N -1.365702116 18.173549452 -20.218959088

H -1.955567108 18.875411613 -19.780098222

C -0.004419160 18.577576399 -20.566595791

C 0.927615106 18.137741554 -19.402797036

O 1.428150041 18.936555524 -18.628606337

C 0.056459273 20.107907277 -20.753135806

O -0.282490237 20.779572208 -19.566997494

H 0.298241109 20.438089693 -18.831821395

H 0.314737792 18.068337856 -21.516447877

H 1.064833421 20.421076310 -21.076116963

H -0.697916038 20.460213288 -21.482189423

N 1.096636185 16.793246246 -19.306689725

H 0.676616527 16.157155270 -19.990261236

C 1.717160766 16.176344174 -18.133903730

C 1.656978452 14.667544027 -18.349070280

O 2.045587218 14.179587297 -19.417111083

H 1.187357878 16.498787240 -17.190875105

H 2.777598273 16.502533940 -18.022346232

N 1.181816547 13.909286497 -17.340099624

H 0.707447808 14.328384460 -16.499635145

C 1.173987864 12.452695852 -17.494136294

C 0.958197920 11.842143852 -16.106918102

O -0.191224777 11.774499340 -15.681679362

H 0.333835641 12.125981178 -18.156282227

H 2.111733768 12.097402688 -17.976616225

N 2.032973019 11.425779367 -15.411912254

H 2.988844621 11.534492577 -15.726451316

C 1.872351004 10.951354817 -14.027185481

C 2.041909069 9.432695840 -13.916848750

C 0.887473583 8.695847325 -14.592827974

H 0.860804717 11.239378678 -13.644664726

H 2.091329529 9.148505354 -12.841718962

H 3.013989076 9.108052921 -14.327045172

H -0.038799010 9.296936792 -14.565298613

H 1.100232354 8.520155349 -15.658275408

C -1.492740206 13.926581285 -14.286136292

C -2.442774952 13.598940587 -15.429757805

C -3.409511996 12.421020771 -15.215242365

C -4.269966538 12.265816650 -16.481876183

C -2.694415695 11.100478237 -14.939636711

H -0.620808220 13.197821942 -14.268611837

H -3.020011133 14.497887964 -15.720797629

H -1.792236383 13.364252191 -16.315864345

H -4.078935744 12.661700418 -14.361432947

H -3.390054078 10.305284388 -14.664682539

H -1.920969189 11.174306706 -14.165217680

H -2.141014837 10.750958736 -15.831570849

H -5.018629059 11.477460520 -16.361756940

H -3.646460071 11.978234944 -17.340436998

H -4.782981677 13.192095758 -16.749191791

C -7.762555001 12.400151170 -12.769729879

C -8.266406161 11.100577877 -12.090880605

O -8.419801102 11.146337459 -10.887380261

C -6.558106168 12.140646122 -13.694536635

O -5.471386548 11.866951003 -12.835109886

H -5.245288564 10.899286603 -12.851488490

H -8.635463143 12.868724269 -13.288551517

H -6.251584502 13.035373104 -14.270925993

H -6.729571864 11.313316467 -14.422252935

N -8.433014550 10.068498925 -12.944350890

H -8.142881321 10.084576962 -13.926106391

C -9.426494926 9.049683897 -12.603385441

C -10.731020443 9.829314589 -12.292846494

O -10.711918238 11.044894593 -12.438988879

C -8.944086110 8.229556877 -11.427079879

H -9.591922311 8.399224398 -13.503326277

H -9.270496333 8.636565498 -10.450904767

H -7.837308524 8.202284846 -11.370830980

N -11.772715116 9.084177792 -11.870329925

H -11.620157889 8.018052499 -11.820475211

C -12.961341216 9.680303050 -11.252635482

C -14.071420622 8.589418814 -11.128180763

O -15.234567102 8.881749510 -11.410378995

C -13.539053615 10.879063905 -12.029801767

C -12.759877599 12.159404054 -11.955451432

C -12.686029394 12.978584115 -13.089840618

C -12.139400147 12.588928292 -10.774876266

C -11.973587967 14.174040074 -13.062663003

C -11.421116922 13.780533585 -10.752339177

C -11.325199803 14.575257437 -11.895899904

H -12.695345386 10.006615915 -10.206662227

H -14.570487545 11.076322244 -11.640670777

H -13.673544295 10.569313231 -13.089400191

H -13.183545864 12.675774448 -14.009560069

H -12.194908894 11.977831646 -9.875651656

H -11.945068309 14.804181259 -13.952924808

H -10.940222264 14.096631376 -9.832044991

H -10.794549191 15.515407672 -11.860387964

N -13.673752837 7.380089507 -10.696036980

H -12.711170042 7.170833327 -10.425834548

C -14.593627304 6.264489350 -10.515383418

H -14.229278336 5.382091215 -11.088317794

H -15.597658102 6.493683320 -10.924990337

C -5.191526249 8.431234752 -17.940428240

C -6.262576169 9.268420086 -18.274742513

C -3.956637180 8.693992701 -18.521386728

C -6.076015361 10.317285993 -19.230935403

C -3.780544313 9.779507119 -19.441590024

C -4.828527162 10.579799930 -19.817699976

C -7.627288688 9.416476896 -17.856175607

N -8.210945589 10.440124327 -18.522347821

N -7.302531637 10.970033854 -19.354931285

H -7.497415502 11.819196275 -19.865131968

S -8.365465669 8.741162079 -16.432231523

O -8.471039322 7.293937317 -16.531141263

O -7.559486929 9.381042888 -15.386949295

C -9.976943011 9.362784653 -16.249297566

C -10.227517694 10.735410474 -16.276381484

C -11.526463519 11.213351331 -16.140580389

C -12.584506380 10.317961412 -15.951214068

C -13.981920077 10.798692256 -15.874188507

C -12.319995553 8.948344362 -15.846153717

C -11.027904566 8.474244862 -16.003976805

N -2.854957490 7.869368290 -18.238028587

N -1.819092579 7.862821840 -19.163998217

C -0.753770187 7.317816168 -18.541203554

C 0.536734872 7.097450469 -19.218765417

C -1.069688031 7.017191302 -17.174654514

C -2.381194265 7.406139217 -16.977211694

C -3.171275162 7.352566079 -15.748389763

H -5.327932576 7.604302222 -17.239658004

H -4.702208699 11.406763626 -20.513630008

H -2.767149281 9.967848461 -19.828700375

H -0.412961234 6.609769458 -16.406517042

H -10.848178429 7.401580891 -15.920953403

H -13.142639386 8.252017102 -15.593038463

H -11.717904461 12.285765323 -16.157193891

H -9.416249163 11.454901731 -16.409717556

H -2.449586088 7.231046440 -14.873690409

H -3.760391835 8.252021017 -15.547462009

H -3.828431205 6.473094788 -15.692642844

H 0.854720408 6.045599719 -19.141951835

H 0.516157937 7.372122217 -20.280577396

H 1.328221959 7.705751966 -18.739040931

H -14.090679483 11.712251974 -15.265840945

H -14.662404704 10.044345047 -15.427957105

H -14.400630315 11.021966204 -16.865263589

Azole **5** with surrounded residues

N -2.564000000 3.856000000 -23.064000000

H -3.693000000 3.924000000 -23.187000000

H -2.359000000 2.885000000 -22.764000000

H -2.156000000 3.990000000 -23.994000000

C -4.435000000 -0.697000000 -16.821000000

O -3.535000000 -0.369000000 -16.048000000

C -3.513000000 -3.092000000 -21.141000000

O -3.952000000 -4.185000000 -20.718000000

O -2.664000000 -3.002000000 -22.059000000

O -5.475000000 -1.196000000 -16.370000000

N -10.051000000 -1.941000000 -14.398000000

H -9.985000000 -1.384000000 -13.537000000

C -11.060000000 -2.110000000 -16.685000000

O -11.995000000 -2.725000000 -17.208000000

O -10.011000000 -1.947000000 -17.324000000

H -10.092000000 -2.943000000 -14.165000000

H -9.156000000 -1.794000000 -14.975000000

N -6.914000000 2.232000000 -21.285000000

H -5.940000000 2.083000000 -20.942000000

C -6.212000000 3.896000000 -22.850000000

O -4.998000000 3.971000000 -22.680000000

H -7.075000000 1.581000000 -22.082000000

H -7.553000000 1.943000000 -20.509000000

O -6.685000000 4.051000000 -23.984000000

N -7.810000000 11.109000000 -24.650000000

H -8.575000000 11.697000000 -24.200000000

H -8.048000000 10.966000000 -25.640000000

H -6.949000000 11.703000000 -24.587000000

C -12.247000000 3.846000000 -21.763000000

O -13.295000000 4.220000000 -22.292000000

N -10.825000000 4.567000000 -15.042000000

H -10.997000000 3.935000000 -14.232000000

H -11.298000000 5.461000000 -14.813000000

H -9.783000000 4.753000000 -15.002000000

O -12.039000000 2.635000000 -21.608000000

N -8.842000000 16.763000000 -14.873000000

H -8.221000000 16.217000000 -14.265000000

C -10.236000000 16.661000000 -16.839000000

O -11.274000000 16.583000000 -16.184000000

H -9.818000000 16.754000000 -14.474000000

H -8.520000000 17.752000000 -14.871000000

O -10.277000000 17.078000000 -18.005000000

N -11.852000000 11.728000000 -22.235000000

H -11.096000000 11.101000000 -22.642000000

H -12.298000000 12.208000000 -23.031000000

H -12.560000000 11.100000000 -21.771000000

C -6.586000000 18.273000000 -20.262000000

O -7.384000000 19.025000000 -19.657000000

O -5.359000000 18.261000000 -20.035000000

C 2.933000000 11.709000000 -13.173000000

O 4.133000000 11.724000000 -13.433000000

C 0.578000000 7.333000000 -13.943000000

O 1.397000000 6.783000000 -13.176000000

O -0.520000000 6.819000000 -14.264000000

O 2.522000000 12.292000000 -12.160000000

N -2.651000000 9.314000000 -9.781000000

H -2.258000000 9.753000000 -8.932000000

H -2.774000000 8.291000000 -9.568000000

H -1.938000000 9.353000000 -10.559000000

C -0.940000000 15.358000000 -14.520000000

O -1.238000000 16.336000000 -13.849000000

O -0.166000000 15.495000000 -15.477000000

N -7.232000000 13.290000000 -11.662000000

H -6.398000000 12.778000000 -11.231000000

H -6.878000000 14.138000000 -12.117000000

H -7.838000000 13.545000000 -10.860000000

C -11.043000000 9.433000000 -10.996000000

O -10.729000000 9.410000000 -9.804000000

C -9.461000000 6.806000000 -11.586000000

O -8.656000000 5.868000000 -11.368000000

O -10.685000000 6.617000000 -11.771000000

O -12.228000000 9.619000000 -11.307000000

C -2.028175772 4.867177623 -22.069445304

C -1.973922428 4.216186891 -20.667125064

O -0.903637980 4.079661968 -20.117323961

C -2.880966799 6.130309148 -22.045267171

C -2.430071621 7.165268338 -23.091779000

C -3.565449607 8.169176573 -23.334921553

C -1.179350047 7.900034286 -22.602757708

H -0.967354762 5.119507079 -22.352604441

H -3.958771698 5.883589022 -22.182459484

H -2.842960242 6.601122117 -21.032411829

H -2.204249954 6.650590080 -24.053878255

H -3.270730124 8.925157628 -24.068310057

H -4.466843671 7.669666272 -23.698151117

H -3.818042479 8.696082941 -22.400698074

H -0.816520573 8.617156623 -23.346660103

H -1.377262989 8.460863665 -21.674986166

H -0.352922651 7.215515731 -22.381948637

N -3.164890646 3.830353410 -20.129588366

H -4.047873757 3.903223862 -20.629982697

C -3.182842345 3.193813512 -18.812359550

C -4.072674092 1.949745137 -18.884686552

O -5.255898294 2.138633861 -19.259642729

H -2.143315176 2.972133226 -18.461387892

H -3.621197246 3.898720002 -18.056029405

N -3.567444039 0.766469667 -18.552913878

H -2.611772172 0.672203407 -18.192678588

C -4.377033963 -0.454164861 -18.372907515

C -3.746550073 -1.673144962 -19.056281027

C -4.065488430 -1.760662255 -20.545381150

H -5.416860606 -0.294511014 -18.737911362

H -4.140908916 -2.597413523 -18.560053943

H -2.654958585 -1.709288975 -18.889042815

H -5.152212320 -1.762998893 -20.717594856

H -3.627874613 -0.924099249 -21.103298784

C -11.245264870 -1.514556095 -15.230116584

C -11.358321865 0.010097951 -15.299070830

C -10.104275773 0.693648576 -15.756839175

C -9.294588615 1.381358327 -14.841289007

C -9.727091682 0.630950546 -17.101304282

C -8.117351411 1.982776352 -15.251307083

C -8.527672724 1.191846767 -17.530098904

C -7.725992388 1.854402860 -16.596664416

O -6.585036475 2.469392406 -16.942676074

H -6.201456935 2.161340177 -17.818182688

H -12.165958114 -1.960663054 -14.795551269

H -12.187577287 0.239002716 -16.013243530

H -11.700441938 0.413841004 -14.326509196

H -9.583218464 1.443019928 -13.794356383

H -10.362037446 0.113012395 -17.834236271

H -7.469683041 2.526406977 -14.558964529

H -8.194427038 1.045115226 -18.549176404

C -7.124406101 3.680529026 -21.636647395

C -6.752138556 4.551019935 -20.417398722

C -6.671004058 6.023364286 -20.814956124

C -7.776404381 4.372922355 -19.295828322

H -8.189377931 3.867269632 -21.944882638

H -5.747047480 4.230580317 -20.028960924

H -7.641982278 6.403151512 -21.152911647

H -6.360409230 6.636759044 -19.958774270

H -5.952303415 6.189856049 -21.624561530

H -7.427354799 4.869329535 -18.375985614

H -7.920188573 3.325911586 -19.002656449

H -8.750626981 4.797384329 -19.559005857

C -7.677744350 9.784070423 -23.914660290

C -9.090597653 9.164602239 -23.954621978

O -10.051847101 9.871755221 -23.661306686

C -7.273636262 10.021033969 -22.480274626

H -6.915402422 9.152782168 -24.448587226

H -7.871405457 10.800557097 -21.975075957

H -7.391049052 9.102290394 -21.869358834

H -6.214041408 10.318249807 -22.371428842

N -9.193683551 7.911358796 -24.435950791

H -8.378130918 7.276279855 -24.479328670

C -10.473694484 7.181996568 -24.295844226

C -10.279605738 6.370894232 -22.992942261

O -9.131201194 6.156860172 -22.629724589

C -10.663180682 6.209662287 -25.488253694

C -11.988540954 5.462757331 -25.353647814

C -10.635346269 6.985783018 -26.809071487

H -11.327444109 7.893974192 -24.230462952

H -9.824170906 5.462315266 -25.482061922

H -12.179542201 4.833321314 -26.233197635

H -12.845713725 6.131694998 -25.242223812

H -12.007485805 4.764373904 -24.493467810

H -10.791262244 6.306827501 -27.658528737

H -9.678213448 7.487422502 -26.971262884

H -11.424363834 7.742453620 -26.850621730

N -11.369246251 6.001158625 -22.289194017

H -12.326607533 6.060272628 -22.668102643

C -11.217510267 4.921328052 -21.288974267

C -11.544158399 5.469898682 -19.896810217

C -11.408142477 4.377078857 -18.828718849

C -11.109165729 5.008624897 -17.464356164

C -11.257320368 3.968937170 -16.352765263

H -10.188438160 4.491046715 -21.312745183

H -10.872623380 6.320660755 -19.677766035

H -12.573878212 5.877803210 -19.889943115

H -12.336570559 3.768384678 -18.808475602

H -10.616876896 3.650683986 -19.107584401

H -10.075112068 5.420957323 -17.463312385

H -11.784423756 5.867092443 -17.290085905

H -10.613754368 3.071228887 -16.564668769

H -12.304254157 3.607480070 -16.277360756

C -8.844401173 16.239041931 -16.289652402

C -8.623277048 14.726613247 -16.328771565

C -8.784570424 14.249874379 -17.776266940

C -7.214491991 14.385605929 -15.838377587

H -8.059087977 16.775196142 -16.894901802

H -9.391709759 14.214764711 -15.700596234

H -8.722772070 13.157700052 -17.839815387

H -9.748731248 14.570912331 -18.190713958

H -7.994446593 14.667783529 -18.420247218

H -6.445562952 15.006439949 -16.336249352

H -6.940151517 13.354648608 -16.106351983

H -7.090223185 14.506452647 -14.764290681

C -11.239257520 12.667475471 -21.209576991

C -10.221177093 13.544043535 -21.957724658

O -9.882563748 13.213679946 -23.090500000

C -10.507241395 11.827989734 -20.159992600

C -11.456892265 11.232281532 -19.099910491

C -10.774169180 10.040400734 -18.425323263

C -11.828701351 12.286495171 -18.056767950

H -12.052826673 13.287158950 -20.744931461

H -9.908884131 11.025216610 -20.641622525

H -9.725156338 12.442821224 -19.650638133

H -12.389663963 10.867855355 -19.591199132

H -11.385014015 9.642142398 -17.602751356

H -10.596168928 9.213229543 -19.121044543

H -9.801810278 10.310438455 -17.987206013

H -12.573993876 11.898647597 -17.346755314

H -10.963798742 12.591241905 -17.451644974

H -12.251759048 13.195728985 -18.493609573

N -9.789146071 14.664628751 -21.336853394

H -10.057801007 14.925757816 -20.382707521

C -8.577003420 15.326996133 -21.860303990

C -7.548310812 14.216629427 -22.140282314

O -7.634388414 13.111605323 -21.628011706

C -8.016090244 16.292794049 -20.795753246

C -7.107433302 17.345257282 -21.404486437

H -8.842043861 15.875609467 -22.800846294

H -8.845710319 16.826345038 -20.254557550

H -7.472994365 15.731488444 -20.007389374

H -6.226420579 16.918908228 -21.907364955

H -7.637377865 17.988795370 -22.122681057

N -6.538698492 14.497372702 -23.034542351

H -6.415549142 15.450466275 -23.393500837

C -5.301192672 13.707022002 -22.952930827

C -4.246744452 14.684596697 -22.371199295

O -3.862305127 15.624557225 -23.037557802

C -4.853840927 13.250981400 -24.354848799

C -3.436232709 12.762798771 -24.316232744

C -3.055323460 11.760016531 -23.415694217

C -2.480244201 13.352891303 -25.142088082

C -1.728715240 11.382674944 -23.295561002

C -1.145963845 12.956053674 -25.077213661

C -0.787403749 11.987389669 -24.139605660

O 0.486574973 11.555742978 -23.981070927

H 1.131329768 12.057562197 -24.538602628

H -5.451562081 12.816689834 -22.287616162

H -5.528787125 12.465130911 -24.743175330

H -4.945106862 14.108087676 -25.060884705

H -3.800182680 11.283436264 -22.778491948

H -2.767987704 14.147536214 -25.830113356

H -1.406453663 10.651407249 -22.548854229

H -0.408035494 13.403591680 -25.733975306

N -3.858741785 14.446275620 -21.090636270

H -4.191436198 13.662199878 -20.545446572

C -2.910291368 15.363964299 -20.441193492

C -1.527635155 14.819970006 -20.851662628

O -1.088485443 13.774755275 -20.406909751

C -3.049823389 15.323027504 -18.923245752

S -4.752251846 15.767318041 -18.411883520

H -3.076548913 16.414088048 -20.827929970

H -2.319329616 16.013649713 -18.441218726

H -2.814368032 14.317925059 -18.505501907

H -4.901182189 17.020516417 -19.035336715

N -0.847528605 15.541271489 -21.797499076

H -1.212683724 16.390155937 -22.216371654

C 0.383469717 15.004865821 -22.369607887

C 1.593440343 15.371798294 -21.471312142

O 2.462144984 16.135750170 -21.861411066

C 0.595827826 15.586618429 -23.783966430

O 0.737111912 16.983859757 -23.738291022

H 1.498397811 17.199785300 -23.130912785

H 0.315938864 13.880203031 -22.433449147

H 1.490273307 15.145036925 -24.258900804

H -0.292094670 15.432325987 -24.425220200

N 1.611613669 14.756607498 -20.261589132

H 0.844615244 14.144216912 -19.970125961

C 2.738929618 14.892382861 -19.343008639

C 3.259164104 13.481196629 -19.024204542

O 4.303509671 13.057974420 -19.479310050

H 2.419758326 15.431516550 -18.415207147

H 3.562165481 15.493204442 -19.799218608

N 2.434980780 12.731759421 -18.236470264

H 1.589900402 13.107745092 -17.808725294

C 2.800081153 11.359466906 -17.875029194

C 1.992387903 11.027332286 -16.604180514

O 0.788388688 10.836508530 -16.714530031

H 2.519497791 10.656816557 -18.690123415

H 3.898903548 11.264511291 -17.737492817

N 2.667332591 11.026296353 -15.444486220

H 3.675270475 11.204193899 -15.361298739

C 1.980876010 10.949129371 -14.140768408

C 1.836303418 9.504172214 -13.651251539

C 0.881008211 8.713448639 -14.535943667

H 0.989698430 11.454249859 -14.198718252

H 1.483457847 9.504820260 -12.602808295

H 2.821702224 8.997050370 -13.609133332

H -0.068745878 9.260857513 -14.674202986

H 1.316217920 8.566204041 -15.539048549

C -3.926406364 9.872116183 -10.400692320

C -4.010701594 11.404277250 -10.348725478

O -5.051323123 11.927083112 -10.711424567

C -5.188682441 9.167312911 -9.910630572

C -5.023984116 7.652857806 -10.017111137

O -3.966160170 7.179452308 -9.552301791

N -5.948014740 6.854339655 -10.556967645

H -6.835872343 7.127932997 -10.999794055

H -5.787795439 5.855800158 -10.617895543

H -3.834132650 9.642667144 -11.528491123

H -5.451933245 9.427874534 -8.864949985

H -6.080047813 9.534544271 -10.493899539

N -2.953551664 12.181129751 -9.912906759

H -2.029011973 11.768538214 -9.802811675

C -2.873928417 13.519202048 -10.555834643

C -2.683578281 13.199607200 -12.086858349

O -2.936438511 12.037923431 -12.404712005

C -1.676320099 14.292337671 -10.002425528

C -1.818123794 14.627084135 -8.508344253

C -0.493670994 15.211799349 -8.003856566

C -2.956646515 15.629599964 -8.274462345

H -3.824836984 14.069457192 -10.393276864

H -0.728487922 13.730816949 -10.182942270

H -1.532304143 15.236030973 -10.575137515

H -2.043295058 13.694647373 -7.939220851

H 0.339624365 14.512140167 -8.157164462

H -0.236616427 16.137838653 -8.534477865

H -0.537243631 15.443296918 -6.937116416

H -2.994886845 15.944315492 -7.227012846

H -3.930475105 15.196846714 -8.516739861

H -2.824323232 16.532332652 -8.882152846

N -2.250319311 14.175637915 -12.885157449

H -2.109881376 15.155199690 -12.556888738

C -1.627324145 13.972056441 -14.212979623

C -2.598350636 13.684475595 -15.355188567

C -3.187366085 12.260918325 -15.391800123

C -3.890118343 12.062160281 -16.749569157

C -2.118370549 11.182466198 -15.222041607

H -0.842461599 13.176014341 -14.142086794

H -3.413491427 14.427403760 -15.355542238

H -2.028203721 13.861385784 -16.300151732

H -3.935069785 12.154562882 -14.573923834

H -4.299860848 11.051267427 -16.831017998

H -3.166765424 12.159511180 -17.572005152

H -4.688811932 12.784182116 -16.909232737

H -2.518831721 10.180692174 -15.402064602

H -1.704260943 11.180987952 -14.205308702

H -1.275381600 11.328516965 -15.918004996

C -7.911201753 12.413387137 -12.685347803

C -8.414775048 11.107579827 -11.930491805

O -7.812697373 10.854349913 -10.910496229

C -6.872321538 11.942723136 -13.723456426

O -6.180599311 10.820212947 -13.268993123

H -5.493298202 11.062880910 -12.598312080

H -8.749557956 12.966313806 -13.149584646

H -6.157858959 12.736527262 -14.013032228

H -7.396365858 11.564814822 -14.630183927

N -9.400769261 10.464152816 -12.572370887

H -9.874114495 10.881426098 -13.355913049

C -9.977774013 9.174466226 -12.109131547

C -8.910492757 8.239614548 -11.583704510

H -10.509185397 8.727454034 -12.987358928

H -8.664116997 8.498657812 -10.530507501

H -7.986964433 8.286600995 -12.184167344

C -5.694054057 5.409167561 -15.198889731

C -4.457522890 5.836088274 -15.793251613

C -3.544610560 4.759112990 -15.547297337

N -4.284080595 3.763013929 -14.891822273

N -5.545992611 4.158395939 -14.702931337

C -2.177221739 4.869301374 -15.847240749

C -1.749983346 6.010532971 -16.492654186

C -2.686998714 7.011044193 -16.879446330

C -4.017293204 6.964622835 -16.485238902

H -4.022467010 2.805341404 -14.692932114

S -7.131688043 6.301501600 -14.874003245

O -8.304547611 5.478858687 -15.221319285

O -6.998510149 6.936928143 -13.590050591

C -7.174989926 7.637532436 -16.040612142

C -7.705925209 7.487985272 -17.316131152

C -7.716231610 8.563204621 -18.205702800

C -7.191975376 9.796714156 -17.812417098

C -7.136770332 10.956077868 -18.745072015

C -6.676145121 9.946655178 -16.522966569

C -6.672873344 8.875010904 -15.636787778

N -2.263207367 8.084967673 -17.702659283

C -1.134911605 8.905587403 -17.504974713

C -1.193758973 9.982045471 -18.367303362

C -2.415300512 9.887447854 -19.106496178

C -3.051336438 10.721791407 -20.023286617

C -3.066188498 8.675612852 -18.680720908

C -4.290169061 10.344671049 -20.518441607

C -4.876733551 9.115505245 -20.157165493

C -4.274953683 8.265150239 -19.247802091

H -1.461024263 4.114511023 -15.516737977

H -0.679955171 6.175518425 -16.665594459

H -4.687791682 7.792584805 -16.693674382

H -0.395808075 8.662752158 -16.740193476

H -0.475308840 10.781798347 -18.445835608

H -2.573281735 11.659294493 -20.315552481

H -4.833348209 11.008921926 -21.179290149

H -5.835552312 8.836835687 -20.578971148

H -4.730033675 7.319151437 -18.967419843

H -8.117577221 6.528069026 -17.643859156

H -8.114420558 8.431717612 -19.204288731

H -6.232623456 10.896170417 -16.224812359

H -6.263151961 9.021115084 -14.620642802

H -7.541220239 11.873285570 -18.296342818

H -6.079198834 11.184489179 -18.998058493

H -7.656232982 10.788178320 -19.690775356

Azole **6** with surrounded residues

N -2.564000000 3.856000000 -23.064000000

H -3.312000000 3.228000000 -22.660000000

H -1.790000000 3.209000000 -23.339000000

C -3.507000000 1.800000000 -18.602000000

O -4.587000000 2.270000000 -18.248000000

O -3.357000000 0.571000000 -18.640000000

N -10.051000000 -1.941000000 -14.398000000

H -9.130000000 -1.676000000 -14.831000000

H -10.037000000 -2.963000000 -14.235000000

H -10.104000000 -1.459000000 -13.475000000

C -7.299000000 -1.436000000 -24.371000000

O -6.417000000 -2.322000000 -24.395000000

O -7.680000000 -0.824000000 -25.394000000

C -6.212000000 3.896000000 -22.850000000

O -4.998000000 3.971000000 -22.680000000

O -6.685000000 4.051000000 -23.984000000

N -11.160000000 5.939000000 -22.257000000

C -12.247000000 3.846000000 -21.763000000

O -13.295000000 4.220000000 -22.292000000

N -10.825000000 4.567000000 -15.042000000

H -10.627000000 3.857000000 -14.316000000

H -11.578000000 5.201000000 -14.629000000

H -9.964000000 5.133000000 -15.152000000

H -12.134000000 6.025000000 -22.681000000

O -12.039000000 2.635000000 -21.608000000

H -10.890000000 6.840000000 -21.846000000

H -10.509000000 5.721000000 -23.032000000

N -19.659000000 7.628000000 -11.972000000

C -18.674000000 9.165000000 -13.615000000

O -17.964000000 10.150000000 -13.771000000

C -15.176000000 6.582000000 -13.982000000

O -14.245000000 6.852000000 -14.766000000

O -15.229000000 5.495000000 -13.377000000

H -20.323000000 7.533000000 -12.781000000

O -19.607000000 8.949000000 -14.400000000

H -20.107000000 8.222000000 -11.265000000

H -19.498000000 6.692000000 -11.560000000

N -18.408000000 13.436000000 -13.639000000

C -18.350000000 15.230000000 -15.328000000

O -17.787000000 16.280000000 -15.632000000

H -19.416000000 13.555000000 -13.913000000

H -18.206000000 12.398000000 -13.616000000

H -18.281000000 13.818000000 -12.696000000

O -19.552000000 15.081000000 -15.588000000

N -8.842000000 16.763000000 -14.873000000

C -10.236000000 16.661000000 -16.839000000

O -11.274000000 16.583000000 -16.184000000

H -9.845000000 16.772000000 -14.503000000

H -8.501000000 17.734000000 -14.843000000

H -8.265000000 16.189000000 -14.248000000

O -10.277000000 17.078000000 -18.005000000

N -11.852000000 11.728000000 -22.235000000

C -10.018000000 13.331000000 -21.868000000

O -9.420000000 12.837000000 -22.819000000

H -11.222000000 11.713000000 -23.094000000

O -9.610000000 14.390000000 -21.372000000

H -11.899000000 10.760000000 -21.881000000

H -12.794000000 12.014000000 -22.531000000

N 2.386000000 11.001000000 -15.462000000

C 2.933000000 11.709000000 -13.173000000

O 4.133000000 11.724000000 -13.433000000

C 0.578000000 7.333000000 -13.943000000

O 1.397000000 6.783000000 -13.176000000

O -0.520000000 6.819000000 -14.264000000

H 3.435000000 11.113000000 -15.455000000

O 2.522000000 12.292000000 -12.160000000

H 2.155000000 10.108000000 -15.947000000

H 1.990000000 11.781000000 -15.993000000

N -0.126000000 8.721000000 -8.903000000

C -0.073000000 6.184000000 -7.674000000

O -0.179000000 7.228000000 -6.994000000

O 0.805000000 5.319000000 -7.452000000

H -0.246000000 9.743000000 -8.897000000

H 0.883000000 8.531000000 -8.746000000

C -3.811000000 11.368000000 -10.436000000

O -4.801000000 12.050000000 -10.695000000

N -7.232000000 13.290000000 -11.662000000

H -6.317000000 12.738000000 -11.263000000

C -9.461000000 6.806000000 -11.586000000

O -8.656000000 5.868000000 -11.368000000

O -10.685000000 6.617000000 -11.771000000

C -14.265000000 8.614000000 -10.320000000

O -15.444000000 8.727000000 -9.974000000

H -4.597000000 4.583000000 -23.396000000

H -6.916000000 14.150000000 -12.098000000

H -7.806000000 13.498000000 -10.831000000

O -13.806000000 7.488000000 -10.558000000

H -2.907000000 4.287000000 -23.941000000

H -0.575000000 8.205000000 -7.921000000

O -2.695000000 11.903000000 -10.388000000

C -2.106336223 4.804215038 -21.942933879

C -1.815755939 3.756423518 -20.813511367

O -1.132158325 2.821023449 -21.213453940

C -3.132983485 5.856861998 -21.588151276

C -3.475169125 6.848862294 -22.716027463

C -4.390531160 7.933710580 -22.139466095

C -2.218093801 7.493746655 -23.305209854

H -1.137456704 5.273870857 -22.248124519

H -4.073336963 5.403489424 -21.196713105

H -2.742025634 6.446663010 -20.716224870

H -4.025554752 6.322800807 -23.533614129

H -4.750626801 8.618409567 -22.915580527

H -3.877692316 8.549956460 -21.386368408

H -5.276688798 7.517548337 -21.641196016

H -2.482200781 8.305954801 -23.996773283

H -1.596537757 7.950343110 -22.523047582

H -1.596691077 6.792851161 -23.864274607

N -2.412637253 3.820608832 -19.614298888

C -2.251222034 2.696853220 -18.690786917

H -2.048652180 3.088462906 -17.659601166

H -1.385640248 2.051136998 -18.974670602

H -2.878529570 4.639406707 -19.223285174

C -11.163322088 -1.464244077 -15.325475486

C -10.993722361 -2.115968233 -16.721984809

O -11.935737891 -2.689893273 -17.219150720

C -11.130730650 0.077061448 -15.431131768

C -9.758954472 0.610855205 -15.673148671

C -8.890719175 0.840877755 -14.588865455

C -9.306894639 0.892478831 -16.971482635

C -7.574856684 1.171433985 -14.799087431

C -7.988521392 1.225213233 -17.213155724

C -7.073786067 1.293599274 -16.133341478

O -5.799956676 1.423453533 -16.245628852

H -5.325892336 1.679876242 -17.249438650

H -12.148047694 -1.801243805 -14.906437474

H -11.825805280 0.377913508 -16.253982198

H -11.581437139 0.527390132 -14.519912132

H -9.271483461 0.785675862 -13.569917078

H -10.002436799 0.827152208 -17.813951494

H -6.882135962 1.362192505 -13.978558383

H -7.627761796 1.403516847 -18.224096249

N -9.749102246 -2.052907473 -17.271015916

H -9.009534811 -1.450614054 -16.893738311

C -9.523949509 -2.480547865 -18.660686063

C -8.213170909 -1.781691860 -19.084501635

O -7.212993312 -1.959604237 -18.416460403

H -9.384830321 -3.584939932 -18.707237043

H -10.397776370 -2.240696145 -19.307010539

N -8.301905370 -0.931610790 -20.135322041

H -9.156394471 -0.800079135 -20.680216960

C -7.117185028 -0.250212112 -20.681090868

C -7.571184441 1.183881331 -20.974997557

O -8.738151461 1.462386084 -21.147942157

C -6.711747956 -0.896632255 -22.022724578

C -7.877271075 -1.018066616 -22.998428030

H -6.267472940 -0.291773023 -19.946029140

H -6.276145943 -1.904656322 -21.848353641

H -5.888898844 -0.316692713 -22.485923594

H -8.601567703 -1.786607489 -22.697931495

H -8.426308755 -0.070575882 -23.130963938

N -6.587288948 2.159816167 -21.171654321

C -7.063062000 3.453906838 -21.646829842

C -7.036034138 4.550996023 -20.564776273

C -7.531118574 5.886208610 -21.122731555

C -7.896064588 4.117726979 -19.377582024

H -8.122081812 3.333374271 -22.048286060

H -5.977672394 4.669957921 -20.226888866

H -8.204588825 4.984819761 -18.765902893

H -7.343690735 3.443742315 -18.709109084

H -8.806663448 3.594190728 -19.691132479

H -8.557960647 5.820097760 -21.480010482

H -7.522895635 6.650956704 -20.319053861

H -6.907884839 6.260865872 -21.937356348

H -5.710993686 2.137411796 -20.665836420

C -11.182161909 4.846639918 -21.215643802

C -11.580989809 5.438118318 -19.874832617

C -11.571784918 4.351591128 -18.788886135

C -11.235564276 4.994844089 -17.442793082

C -11.271949751 3.941648007 -16.338822602

H -10.191818489 4.329558118 -21.167768589

H -12.596827658 5.885939167 -19.922722360

H -10.881557416 6.253753092 -19.588107980

H -12.555844624 3.839182276 -18.765875129

H -10.845757665 3.551111763 -19.040880409

H -10.228870053 5.476284042 -17.511050124

H -11.960585444 5.813136283 -17.222833626

H -12.307660239 3.544334691 -16.207912532

H -10.604852108 3.081057094 -16.580863807

C -18.321948209 8.200815549 -12.468675431

C -17.429750840 7.075244815 -12.983121866

C -16.222996181 7.672472536 -13.710737321

H -17.802026036 8.737424050 -11.625564659

H -17.021548603 6.455388664 -12.143140828

H -17.964229232 6.391215254 -13.663921392

H -15.723444175 8.435794595 -13.074524610

H -16.508055370 8.155247713 -14.656013534

C -17.518202041 14.065799503 -14.702397681

C -17.239297360 13.088035592 -15.833966640

C -16.023270606 12.206320534 -15.610293378

O -14.893948809 12.663677182 -15.567294358

N -16.235930683 10.853677557 -15.584618134

H -15.474295253 10.229866022 -15.363906992

H -17.155383231 10.467685307 -15.374220316

H -16.578627468 14.453479272 -14.241210449

H -17.030727409 13.689269556 -16.761698048

H -18.144735604 12.504250257 -16.099182106

C -8.842517244 16.215713331 -16.282178421

C -8.691285419 14.693023641 -16.293204153

C -8.993552302 14.189307468 -17.705769682

C -7.268211244 14.289152703 -15.895899462

H -8.046360999 16.704729730 -16.885469274

H -9.434300903 14.233284709 -15.593143127

H -8.836881730 13.110251487 -17.796135953

H -8.374094838 14.676659839 -18.473073982

H -10.032616658 14.421019645 -18.003074206

H -6.516218723 14.802976689 -16.506910280

H -7.031139141 14.469298014 -14.847447232

H -7.102360891 13.215385421 -16.086920798

C -11.284560019 12.692404878 -21.209801768

C -10.962948035 11.956694000 -19.928473088

C -12.243321779 11.573921630 -19.147634070

C -12.171712220 10.113867820 -18.690198057

C -12.429372507 12.510832353 -17.954139701

H -12.016669184 13.519719487 -21.025363376

H -10.317156887 12.625147003 -19.302795240

H -10.305485835 11.085013328 -20.101411402

H -13.136882170 11.684325648 -19.806781329

H -13.340179445 12.278549906 -17.378305402

H -12.520089006 13.561514817 -18.263706471

H -11.591533986 12.456505239 -17.251194027

H -13.017061888 9.867305606 -18.029121972

H -12.187363687 9.411517310 -19.521366135

H -11.263754307 9.921714266 -18.100951306

C 1.880665922 10.961211890 -14.030347850

C 1.758826791 9.513340924 -13.586224666

C 0.859841782 8.735619294 -14.531902049

H 0.901396326 11.491946509 -13.962764082

H 2.745795545 9.014447453 -13.478239508

H 1.348242603 9.482987316 -12.550480750

H -0.120312172 9.226876402 -14.668277062

H 1.299474838 8.565698176 -15.524403982

C -0.557070700 8.080819592 -10.197622601

C -1.990653628 8.415931318 -10.622555574

O -2.446550199 7.930587226 -11.631771127

C -0.403805025 6.557566678 -10.112937943

C -1.025624263 5.965114886 -8.855172595

H 0.110930837 8.454741504 -11.034186250

H -0.857288327 6.107771412 -11.036170737

H 0.665409815 6.262145150 -10.199737646

H -2.024407017 6.383734535 -8.646282279

H -1.173971454 4.874806571 -8.981787591

N -2.756169493 9.257977573 -9.828957043

H -2.330821489 9.847577781 -9.130528668

C -3.948314187 9.832957489 -10.464187612

C -5.228480823 9.301675898 -9.816485119

C -5.800491211 8.182177032 -10.663212768

O -5.664296135 8.176382153 -11.881740933

N -6.514355485 7.184722827 -10.064337498

H -6.756549271 7.212690191 -9.099419757

H -7.091255762 6.538443580 -10.646471950

H -3.960547122 9.568679429 -11.568973172

H -5.048420017 8.990404070 -8.772965232

H -5.992384314 10.119362738 -9.772641088

C -7.806211918 12.364726518 -12.696569223

C -8.402999247 11.129288640 -11.965569857

O -8.034300800 10.909499746 -10.835933047

C -6.604046580 11.816649887 -13.548335974

O -5.467052520 12.567868497 -13.247652189

H -4.980603220 12.199999077 -12.423583886

H -8.560793301 12.889239450 -13.312895992

H -6.427841808 10.734351579 -13.352898282

H -6.780042499 11.956660725 -14.627905207

N -9.155018980 10.347549691 -12.792353652

H -9.516459633 10.712734078 -13.662966787

C -9.900602423 9.213324853 -12.215269428

C -10.945935130 9.858667057 -11.272950823

O -10.706614330 10.949080583 -10.769446429

C -8.945217121 8.246811152 -11.539363443

H -10.430438101 8.679719121 -13.055337915

H -7.958085252 8.276259188 -12.052086643

H -8.766847268 8.530094543 -10.486104670

N -12.153534951 9.251165643 -11.253338363

C -13.284496953 9.820845258 -10.508205377

C -14.010533751 10.935067519 -11.288978191

C -13.193011254 12.157830577 -11.572829861

C -13.215183006 12.731837518 -12.845650869

C -12.415059775 12.762802653 -10.576432956

C -12.455722246 13.865173794 -13.133358386

C -11.654873361 13.892651164 -10.862829511

C -11.666794263 14.445990953 -12.144987691

H -12.959152613 10.191181605 -9.510948849

H -14.912467888 11.212942330 -10.698427829

H -14.419585170 10.499601496 -12.226418666

H -13.834127870 12.299139667 -13.634410754

H -12.370367243 12.318514491 -9.581835684

H -12.497633305 14.300550331 -14.133254534

H -11.047458519 14.339392875 -10.083705929

H -11.090075877 15.334326034 -12.359070542

H -12.223033062 8.205574978 -11.436765476

O -7.528953103 9.325311941 -18.241739392

S -7.804742593 8.310135844 -17.237826039

O -8.150299229 6.971746818 -17.753464795

C -9.207458170 8.819660831 -16.327972604

C -10.049454902 7.828052878 -15.810812755

C -11.274843580 8.161821326 -15.262342911

C -11.662856074 9.510335753 -15.187266239

C -12.978650211 9.840256787 -14.609797595

C -10.784567320 10.504134005 -15.632978205

C -9.566707954 10.159002816 -16.218729602

C -6.418996852 8.092895635 -16.234706724

N -6.301044272 8.657889201 -14.983107099

N -5.118411999 8.329095417 -14.460029746

H -4.944089346 8.473448316 -13.448971692

C -4.413175058 7.531089271 -15.327387229

C -5.246572547 7.354781353 -16.493954872

C -3.187056836 6.826094582 -15.623620477

N -4.495795386 6.641306850 -17.442249375

N -3.291611755 6.263784313 -16.824671337

C -5.146155743 5.548606330 -18.168477708

H -9.731888973 6.787436909 -15.849913728

H -11.975574371 7.408221055 -14.866644464

H -11.074438189 11.550066447 -15.541695368

H -8.914217393 10.946474481 -16.603581982

H -2.282703491 6.739563418 -14.988197034

H -6.250754650 5.647036700 -18.109837957

H -4.843546849 5.607417764 -19.225100731

H -4.884090633 4.542408380 -17.770774160

H -13.427711994 10.746971254 -15.035695443

H -13.698811825 8.990971085 -14.708772187

H -12.900103600 9.991471251 -13.510398550

Azole **7** with surrounded residues

N -2.564000000 3.856000000 -23.064000000

H -3.696000000 3.943000000 -23.199000000

H -2.379000000 2.887000000 -22.754000000

H -2.141000000 3.977000000 -23.988000000

C -3.507000000 1.800000000 -18.602000000

O -4.587000000 2.270000000 -18.248000000

O -3.357000000 0.571000000 -18.640000000

N -6.914000000 2.232000000 -21.285000000

C -6.212000000 3.896000000 -22.850000000

O -4.998000000 3.971000000 -22.680000000

H -6.757000000 1.618000000 -22.102000000

O -6.685000000 4.051000000 -23.984000000

H -6.086000000 2.140000000 -20.637000000

H -7.741000000 1.860000000 -20.771000000

N -7.810000000 11.109000000 -24.650000000

H -8.743000000 11.542000000 -24.450000000

H -7.089000000 11.821000000 -24.368000000

H -7.738000000 10.969000000 -25.672000000

C -12.247000000 3.846000000 -21.763000000

O -13.295000000 4.220000000 -22.292000000

N -10.825000000 4.567000000 -15.042000000

H -10.536000000 3.867000000 -14.337000000

H -11.621000000 5.128000000 -14.593000000

H -10.047000000 5.231000000 -15.169000000

O -12.039000000 2.635000000 -21.608000000

N -19.659000000 7.628000000 -11.972000000

C -18.674000000 9.165000000 -13.615000000

O -17.964000000 10.150000000 -13.771000000

C -15.176000000 6.582000000 -13.982000000

O -14.245000000 6.852000000 -14.766000000

O -15.229000000 5.495000000 -13.377000000

H -20.305000000 7.493000000 -12.789000000

O -19.607000000 8.949000000 -14.400000000

H -20.132000000 8.241000000 -11.299000000

H -19.490000000 6.710000000 -11.522000000

N -18.408000000 13.436000000 -13.639000000

C -18.350000000 15.230000000 -15.328000000

O -17.787000000 16.280000000 -15.632000000

H -19.415000000 13.554000000 -13.913000000

H -18.202000000 12.396000000 -13.617000000

H -18.280000000 13.815000000 -12.695000000

O -19.552000000 15.081000000 -15.588000000

N -9.636000000 14.455000000 -21.276000000

H -10.382000000 15.078000000 -20.884000000

C -6.586000000 18.273000000 -20.262000000

O -7.384000000 19.025000000 -19.657000000

O -5.359000000 18.261000000 -20.035000000

H -10.062000000 13.877000000 -22.016000000

H -9.340000000 13.790000000 -20.505000000

C -0.785000000 14.060000000 -21.008000000

O -0.324000000 13.047000000 -20.490000000

O -0.141000000 14.634000000 -21.897000000

N 2.386000000 11.001000000 -15.462000000

C 2.933000000 11.709000000 -13.173000000

O 4.133000000 11.724000000 -13.433000000

C 0.578000000 7.333000000 -13.943000000

O 1.397000000 6.783000000 -13.176000000

O -0.520000000 6.819000000 -14.264000000

H 3.436000000 10.939000000 -15.464000000

H 2.002000000 10.209000000 -16.009000000

H 2.133000000 11.889000000 -15.929000000

O 2.522000000 12.292000000 -12.160000000

N -2.154000000 13.882000000 -12.921000000

C -0.940000000 15.358000000 -14.520000000

O -1.238000000 16.336000000 -13.849000000

H -2.312000000 14.873000000 -12.579000000

H -1.546000000 13.413000000 -12.224000000

H -3.054000000 13.389000000 -12.956000000

O -0.166000000 15.495000000 -15.477000000

N -7.232000000 13.290000000 -11.662000000

H -6.900000000 12.716000000 -10.832000000

H -6.410000000 13.743000000 -12.088000000

H -7.860000000 14.022000000 -11.287000000

C -9.461000000 6.806000000 -11.586000000

O -8.656000000 5.868000000 -11.368000000

O -10.685000000 6.617000000 -11.771000000

C -14.265000000 8.614000000 -10.320000000

O -15.444000000 8.727000000 -9.974000000

O -13.806000000 7.488000000 -10.558000000

C -2.017006466 4.847174250 -22.045365591

C -1.619526110 3.985184943 -20.811079285

O -0.471116484 3.585155675 -20.768549152

C -3.002161053 5.965148632 -21.741202851

C -2.966728210 7.084871951 -22.801447204

C -4.270315811 7.895821481 -22.740495216

C -1.770151362 8.009544489 -22.570875673

H -1.061907236 5.283720179 -22.451676334

H -2.770776107 6.399817242 -20.741699917

H -4.040770928 5.573611263 -21.647408530

H -2.889197381 6.628759305 -23.815613221

H -4.237667111 8.732387550 -23.446077212

H -5.133112405 7.270110808 -22.990554703

H -4.429633361 8.310665510 -21.737426331

H -1.704172450 8.785374586 -23.346102056

H -0.817887491 7.469622938 -22.575292548

H -1.837077125 8.535706733 -21.608424128

N -2.585290050 3.658062183 -19.924544930

C -2.291978223 2.736665789 -18.821900010

H -1.382712416 2.132467065 -19.027424554

H -2.110761951 3.308119429 -17.881455759

H -3.542703603 3.964230707 -20.009058866

C -7.079244764 3.703135812 -21.602572139

C -6.539956642 4.511652193 -20.395675487

C -6.715578257 6.005673203 -20.652925780

C -7.245210857 4.101523641 -19.101776749

H -8.148822080 3.955369202 -21.821998789

H -5.442567852 4.299025624 -20.286133847

H -6.765434334 3.227223633 -18.625692724

H -7.156575520 4.898474830 -18.343429541

H -8.314776161 3.925752186 -19.231666595

H -7.736697203 6.324287764 -20.405032755

H -6.034920010 6.597416124 -20.028991146

H -6.536814803 6.272760626 -21.699207240

C -7.594542002 9.793780071 -23.920908961

C -8.886763184 8.996974497 -24.178539414

O -9.917904415 9.628972395 -24.353240856

C -7.403461358 10.036389075 -22.439018538

H -6.691181976 9.290580547 -24.360415141

H -8.170717250 10.706934599 -22.010479738

H -7.462130250 9.097775170 -21.858903368

H -6.427791202 10.489047676 -22.201688911

N -8.779403482 7.654809323 -24.282304543

H -7.934621786 7.139699536 -24.019786216

C -10.007485576 6.837679086 -24.258719006

C -10.112267983 6.364113905 -22.798860565

O -9.077979174 6.374511854 -22.121425326

C -9.832384717 5.609426114 -25.192479204

C -11.101600788 4.760859507 -25.200436044

C -9.519036795 6.082741774 -26.615956947

H -10.893342509 7.446716259 -24.573268220

H -8.972602170 4.986961240 -24.820305464

H -11.034389916 3.958172026 -25.945573874

H -11.999510158 5.342310579 -25.434742335

H -11.285291428 4.249043816 -24.240468130

H -9.419313519 5.226182127 -27.294415160

H -8.576509843 6.635662310 -26.664040972

H -10.309836564 6.727198114 -27.013320842

N -11.283892581 5.946825008 -22.292127173

C -11.266192998 4.936846936 -21.212535938

C -11.789658417 5.482495006 -19.880261721

C -11.584869319 4.425664889 -18.783743616

C -11.509350901 5.057662043 -17.390112081

C -11.266627323 3.956457279 -16.350234768

H -10.243607453 4.516643170 -21.089024383

H -12.869349101 5.735001399 -19.971062839

H -11.281514116 6.425402297 -19.620031846

H -12.414237315 3.686649802 -18.843602238

H -10.666631995 3.843649961 -18.988809092

H -12.456650745 5.608868191 -17.163873509

H -10.709876267 5.823836518 -17.368783620

H -10.491676659 3.239456025 -16.695776009

H -12.209607553 3.380159486 -16.176679844

H -12.176507219 5.964345307 -22.814317198

C -18.322841907 8.205022780 -12.465438913

C -17.430215348 7.078576924 -12.979495485

C -16.223544395 7.675129373 -13.706393425

H -17.806504990 8.741811731 -11.622218837

H -17.964074727 6.396266874 -13.662853691

H -17.021904795 6.457069752 -12.141075903

H -16.503197035 8.160641927 -14.652074585

H -15.717961904 8.433920415 -13.072973541

C -17.504405316 14.090191745 -14.673230690

C -17.107954590 13.107416417 -15.763791452

C -15.783713902 12.402279469 -15.496926939

O -14.714360407 12.985093737 -15.604682100

N -15.840256333 11.073902476 -15.206304777

H -15.008262999 10.546235008 -15.002592940

H -16.719296750 10.589650069 -15.016963175

H -16.608461961 14.529248432 -14.171501669

H -17.925765707 12.390555820 -15.991314154

H -16.981114306 13.679151281 -16.720954292

C -8.487430223 15.255252050 -21.859758270

C -7.538096060 14.207784299 -22.477898933

O -8.001215953 13.106461288 -22.803274595

C -7.847144099 16.148882313 -20.781201644

C -7.143012812 17.341989445 -21.406982858

H -8.895733405 15.904501947 -22.682104317

H -8.615565497 16.542816952 -20.075563315

H -7.133533837 15.579094316 -20.146725288

H -7.815480714 17.966203482 -22.010408002

H -6.271925180 17.075787116 -22.031514701

N -6.261633751 14.562501263 -22.609626802

H -5.952019757 15.562636089 -22.429220222

C -5.151088340 13.624696608 -22.841534968

C -3.942270417 14.465031857 -22.312567246

O -3.894642198 15.640131890 -22.627160115

C -4.948922188 13.270599095 -24.331817947

C -3.490418338 13.021359019 -24.521512789

C -2.899679523 11.791482302 -24.205457045

C -2.667260103 14.114043276 -24.860452174

C -1.536265060 11.689145978 -24.039966798

C -1.310530585 14.048243246 -24.685411134

C -0.721315182 12.867058767 -24.119956287

O 0.455280109 12.894846725 -23.668325845

H 0.414758811 13.974757286 -22.597017789

H -5.302084010 12.696715440 -22.242229981

H -5.562012016 12.397175636 -24.610941051

H -5.285311893 14.109991066 -24.974136677

H -3.528891009 10.918667461 -24.058639080

H -3.128307494 15.037368055 -25.210652688

H -1.049764978 10.761914113 -23.778332653

H -0.660368439 14.888876528 -24.891162193

N -3.136852377 13.887991684 -21.383073018

C -2.138078966 14.726240387 -20.698206413

C -2.426228263 14.793690692 -19.202650558

S -4.160276008 15.306131196 -18.862752446

H -2.136148328 15.767266117 -21.150435197

H -2.275774482 13.808996407 -18.717982003

H -1.715468548 15.486796126 -18.702117033

H -4.177549678 16.589545085 -19.323514590

H -3.095357533 12.893072250 -21.229902520

C 1.883208398 10.958128597 -14.035957018

C 1.762033560 9.509301892 -13.595972083

C 0.889669641 8.721670455 -14.558056616

H 0.907438972 11.487708100 -13.978228200

H 1.341996838 9.460950207 -12.564703252

H 2.750571017 9.015703615 -13.471163873

H -0.077447331 9.210616241 -14.749410828

H 1.372597576 8.510820635 -15.523291428

C -1.507617426 13.912101147 -14.290631299

C -2.511533928 13.623317054 -15.391781944

C -3.056538795 12.183409582 -15.410118012

C -4.121537891 12.064923179 -16.507462011

C -1.941064900 11.166367649 -15.665091816

H -0.652869949 13.196090724 -14.304849611

H -2.006701584 13.842854176 -16.368594384

H -3.345742873 14.352094862 -15.371373475

H -3.542509462 11.950812442 -14.433425222

H -4.617795400 11.086675131 -16.483698310

H -4.907082829 12.819670343 -16.403895644

H -3.687719477 12.184432568 -17.506543313

H -2.350995720 10.153980440 -15.792949494

H -1.386445627 11.402794293 -16.580886772

H -1.233296672 11.113632584 -14.831553315

C -7.877423184 12.441822927 -12.745259908

C -8.279279135 11.086879731 -12.069868234

O -7.722808169 10.860984947 -11.010114450

C -6.820381636 12.213413519 -13.844815694

O -5.574112315 12.062392275 -13.192988952

H -5.402056994 11.111216140 -12.986023644

H -8.788501775 12.954221194 -13.140318132

H -6.682332823 13.092155410 -14.503145116

H -7.054931045 11.339429244 -14.495779710

N -9.103669825 10.316927062 -12.802327084

H -9.520471689 10.636830335 -13.680088579

C -9.851491632 9.205556741 -12.174984916

C -10.909964654 9.884423930 -11.261120007

O -10.674169743 11.018274630 -10.848231068

C -8.937657682 8.244489940 -11.447862815

H -10.401162089 8.674503999 -13.015615801

H -7.896501925 8.291583484 -11.805568180

H -8.869142291 8.470221919 -10.365157278

N -12.072545460 9.224472764 -11.135973281

C -13.269868250 9.819306400 -10.527839145

C -13.929106131 10.835196603 -11.482237566

C -13.117675071 12.069345461 -11.726562669

C -12.923028745 12.533894483 -13.029666330

C -12.553061758 12.786825914 -10.663129903

C -12.181102703 13.690167424 -13.266479711

C -11.807081342 13.936805389 -10.901412282

C -11.616477633 14.394002946 -12.206430061

H -13.031865622 10.280189625 -9.546914562

H -14.159958061 10.312626108 -12.437456968

H -14.920698262 11.105136334 -11.056686651

H -13.357867267 11.997614837 -13.874901432

H -12.666638023 12.417068040 -9.644618656

H -12.062702039 14.054279791 -14.282609071

H -11.370571061 14.476630860 -10.068379609

H -11.056332399 15.301551859 -12.389968335

H -12.143214193 8.182660069 -11.344208443

C -10.738235305 11.227801342 -18.452630411

C -11.030501874 12.008905931 -19.598195163

C -11.192465222 11.692091729 -17.193594980

N -11.457858199 13.310527937 -19.482982719

C -11.799829660 12.923971673 -17.120270626

C -11.844864022 13.736747823 -18.280491292

C -10.091123236 9.984702691 -18.679444520

C -10.086568891 9.436940952 -19.997626682

C -10.970159934 11.440653093 -20.922991967

C -10.568412457 10.153694637 -21.122143847

C -9.366133375 9.038767317 -17.894802249

N -9.035673893 7.972570162 -18.660094648

N -9.448479033 8.204592946 -19.916825984

H -9.414937955 7.461103158 -20.621384334

S -8.716662563 9.218902980 -16.262858053

O -7.682870034 10.239850386 -16.369719797

O -9.826137540 9.560045959 -15.388301070

C -7.993689511 7.706330214 -15.831363176

C -6.927624487 7.207944785 -16.587786518

C -6.262926416 6.056604714 -16.188135309

C -6.636968147 5.397716802 -15.007630342

C -5.863698004 4.218814812 -14.550616142

C -7.714514741 5.889259625 -14.265555058

C -8.389859882 7.032905995 -14.674807514

H -11.112039093 11.045300250 -16.301204107

H -12.278416893 13.271863871 -16.193036843

H -12.226335508 14.766604820 -18.225099322

H -11.350635701 12.039249277 -21.748457429

H -10.619420152 9.676933836 -22.100809325

H -6.592509691 7.717117015 -17.494294105

H -5.421589804 5.676871514 -16.768994937

H -7.975108156 5.429675119 -13.288499581

H -9.215351918 7.413598870 -14.046247729

H -4.866742151 4.527802528 -14.173369595

H -6.343995828 3.672092425 -13.726813125

H -5.661320411 3.498060431 -15.360775634

# **Fig. S9** The first (best) pose of azole **1** docked to Chk1 binding site


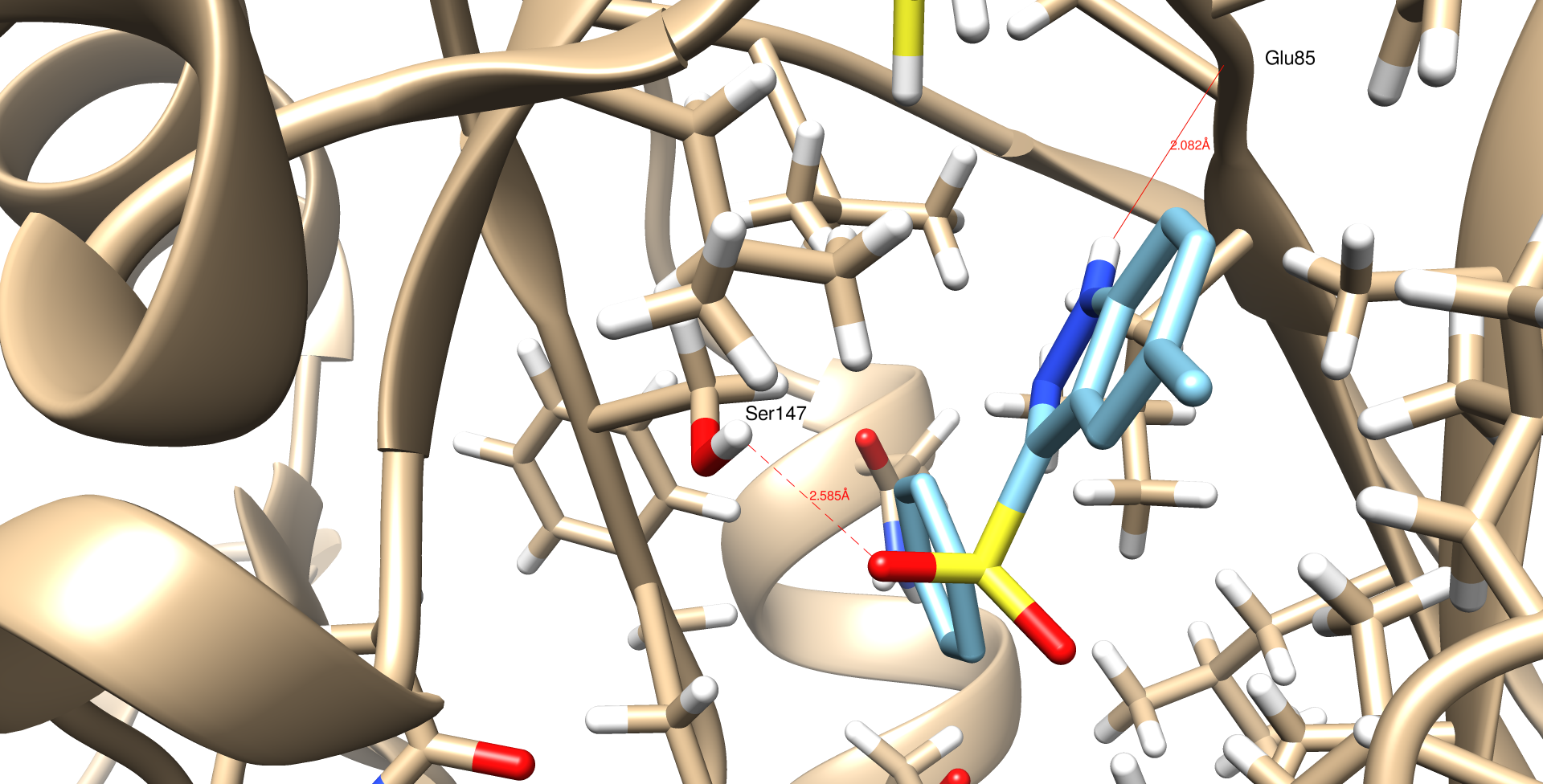


# **Fig. S10** The first (best) pose of azole **2** docked to Chk1 binding site


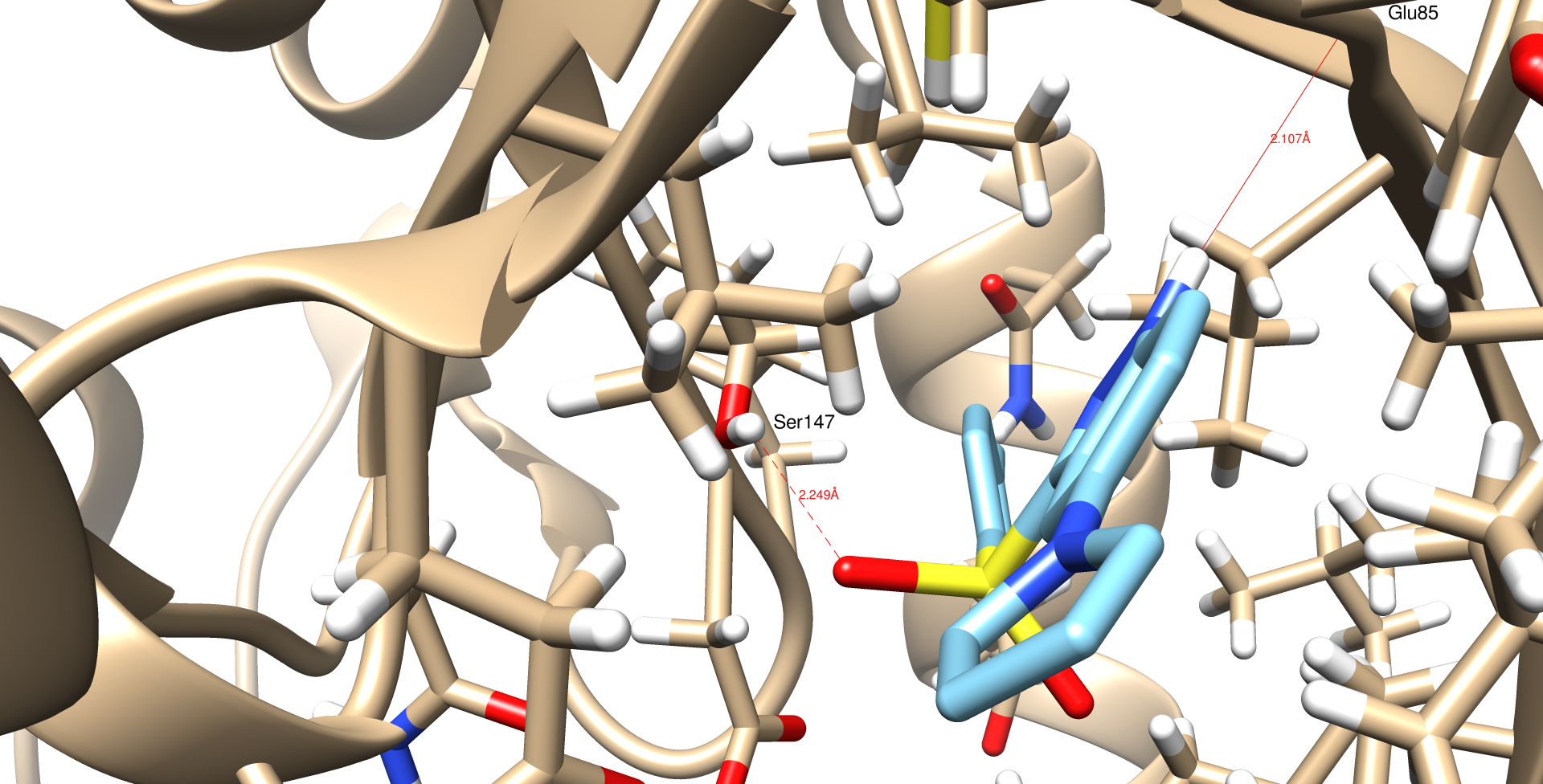


# **Fig. S11** The first (best) pose of azole **3** docked to Chk1 binding site


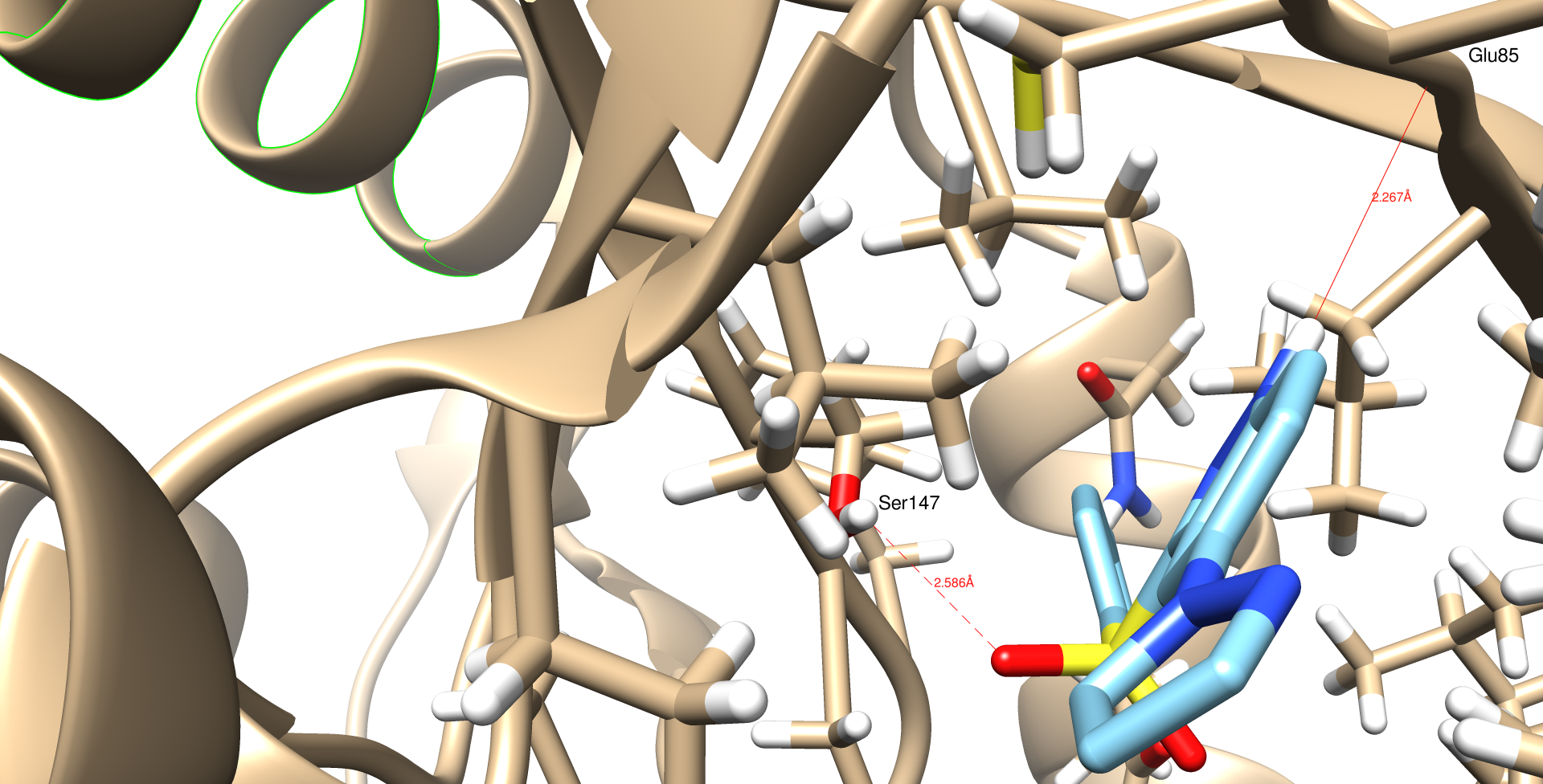


# **Fig. S12** The first (best) pose of azole **4** docked to Chk1 binding site


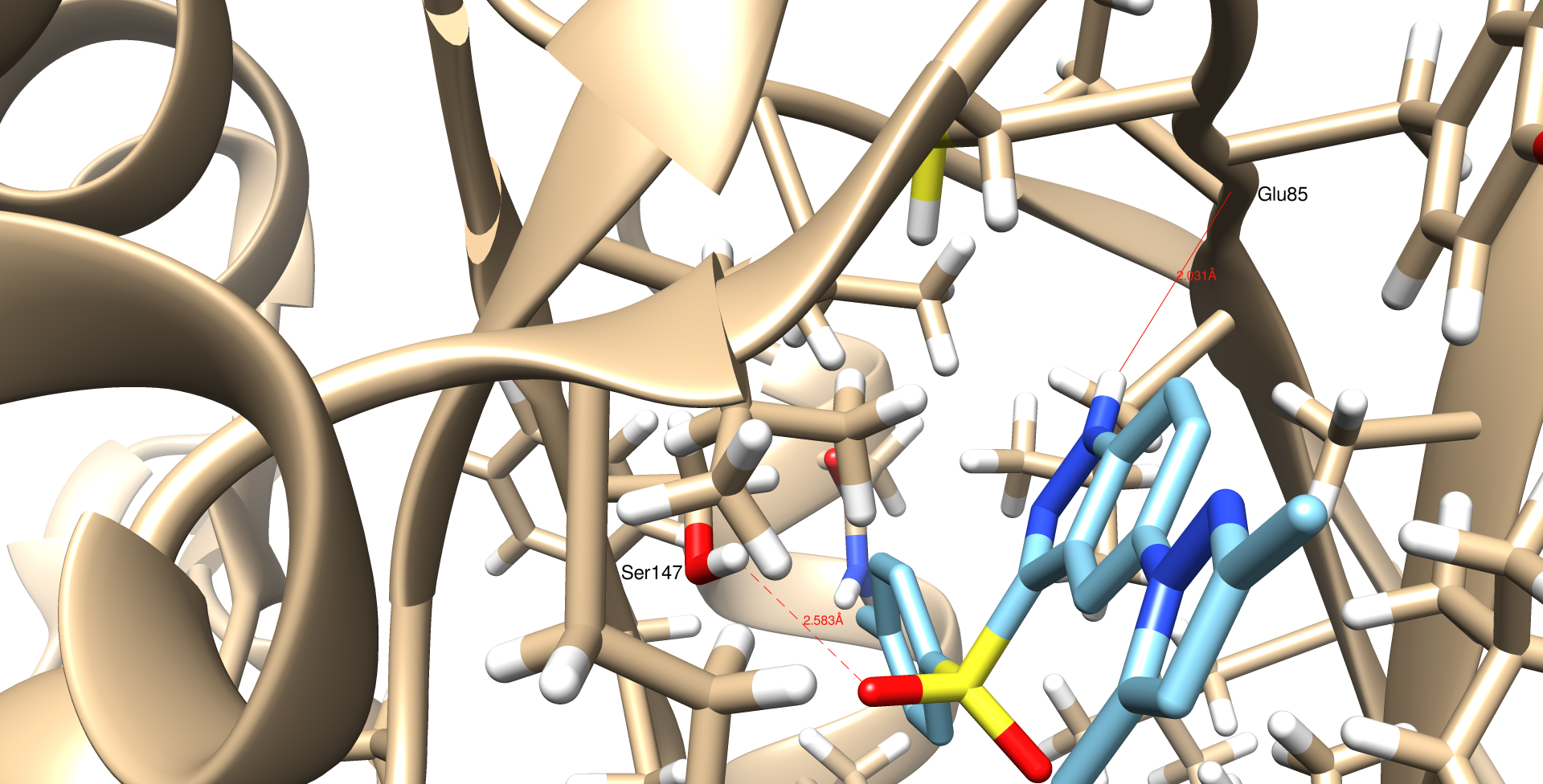


# **Fig. S13** The first (best) pose of azole **5** docked to Chk1 binding site


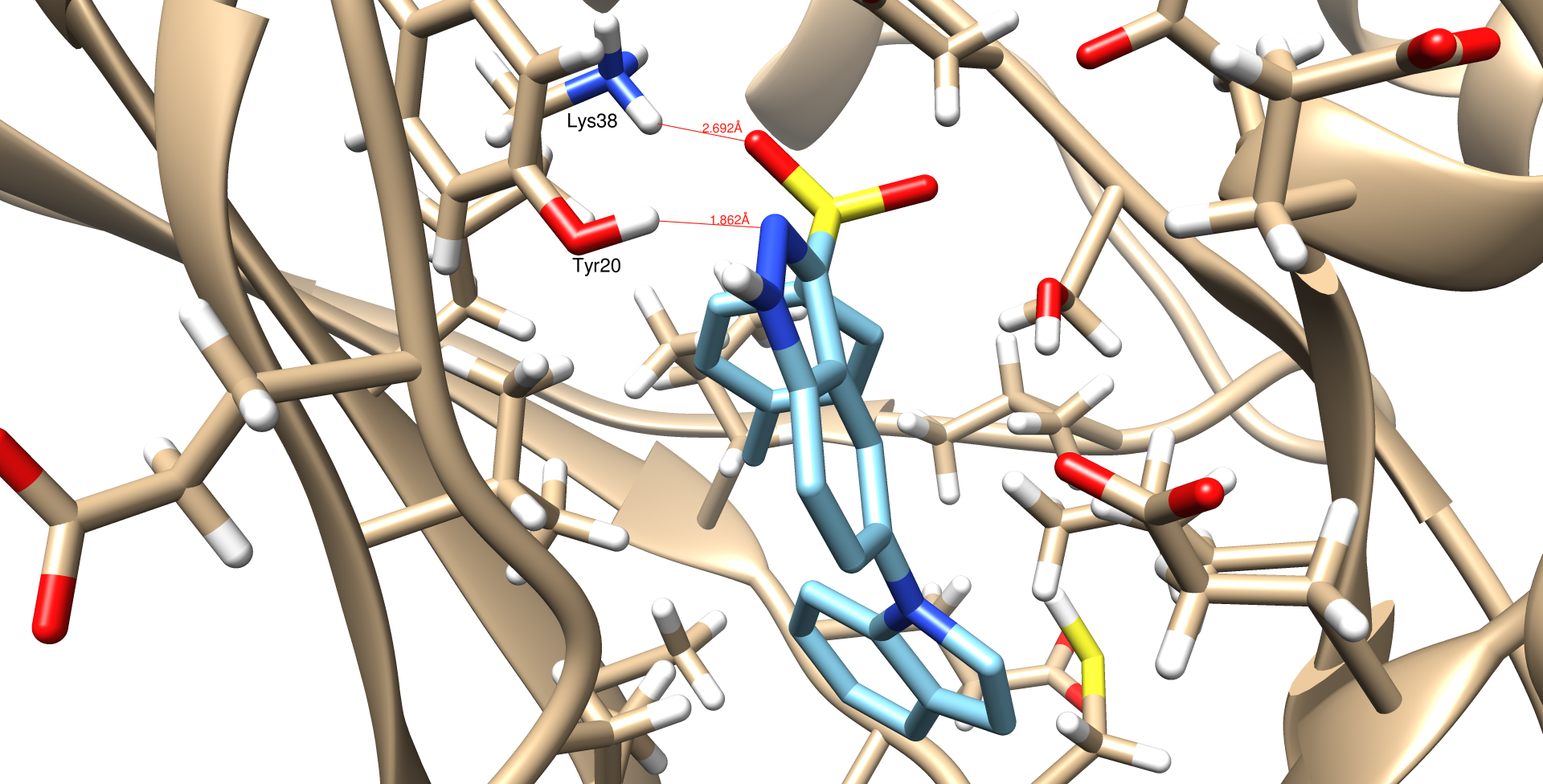


# **Fig. S14** The first (best) pose of azole **6** docked to Chk1 binding site


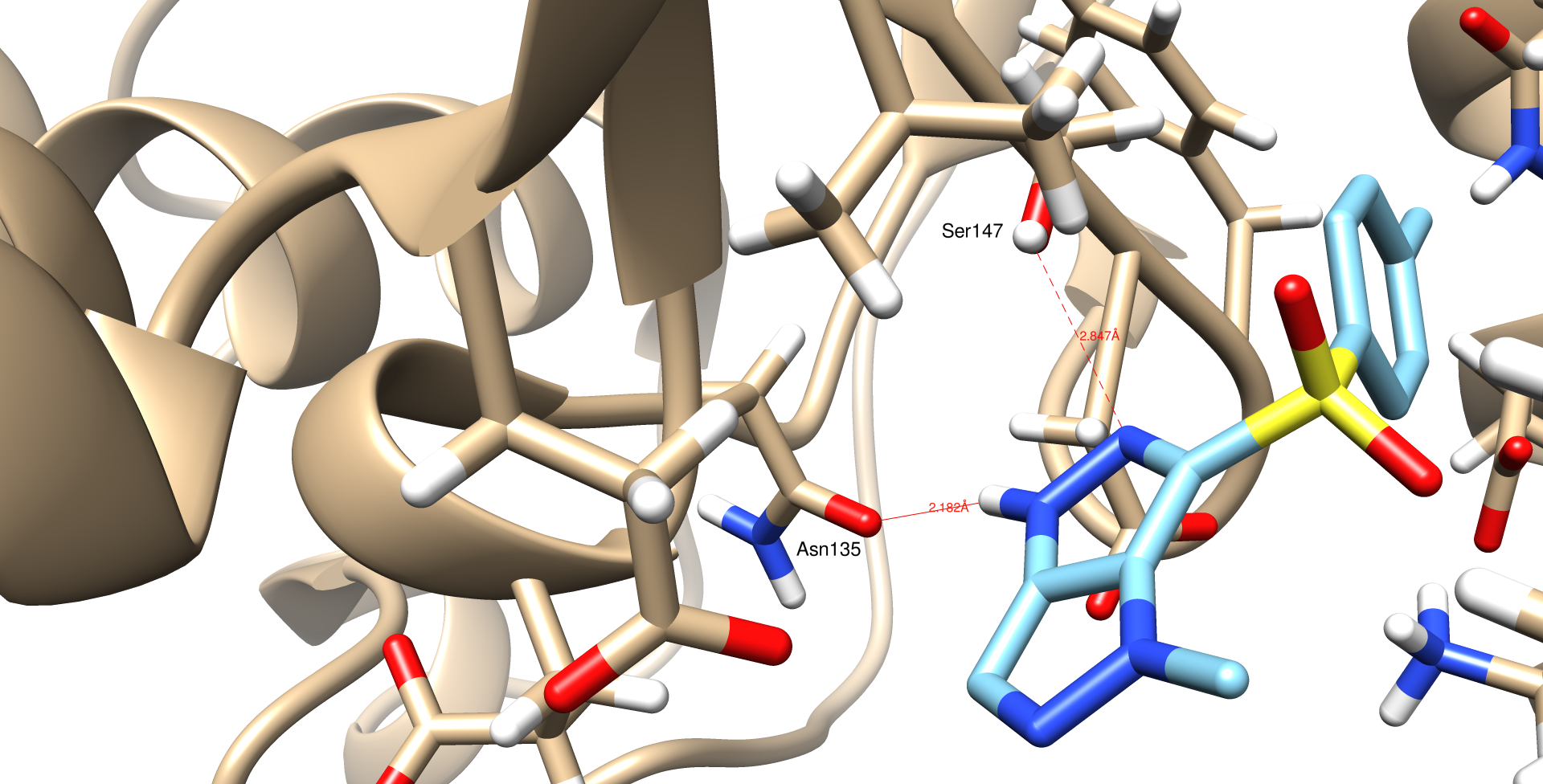


# **Fig. S15** The first (best) pose of azole **7** docked to Chk1 binding site


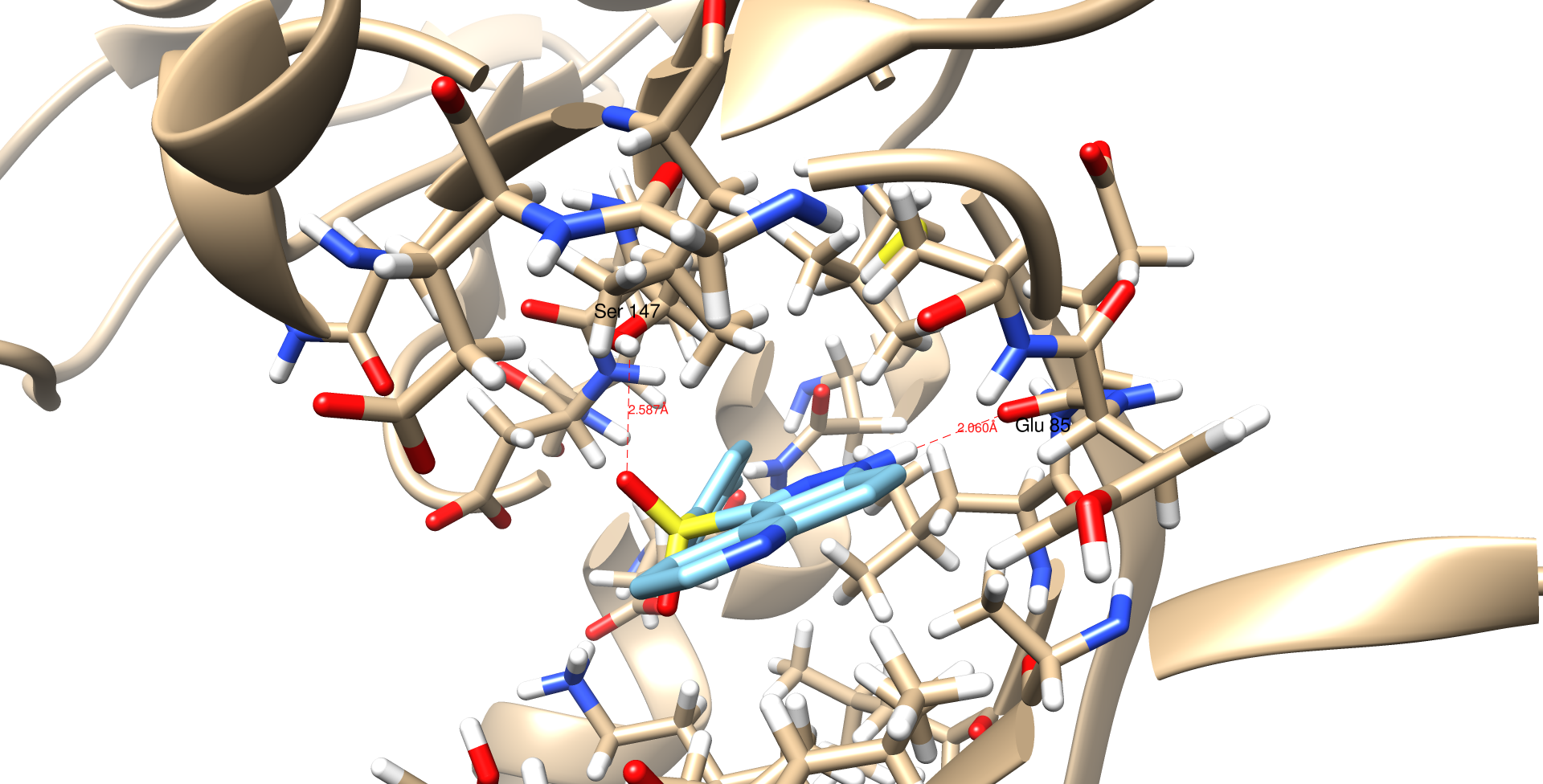


# **Fig. S16** Close contacts within the **1**‑**2e9n** complex (HB marked with dashes, π‑cation contact with Lys38, π‑π stacking contact with Tyr86; *MGLTools 1-5-6* program)


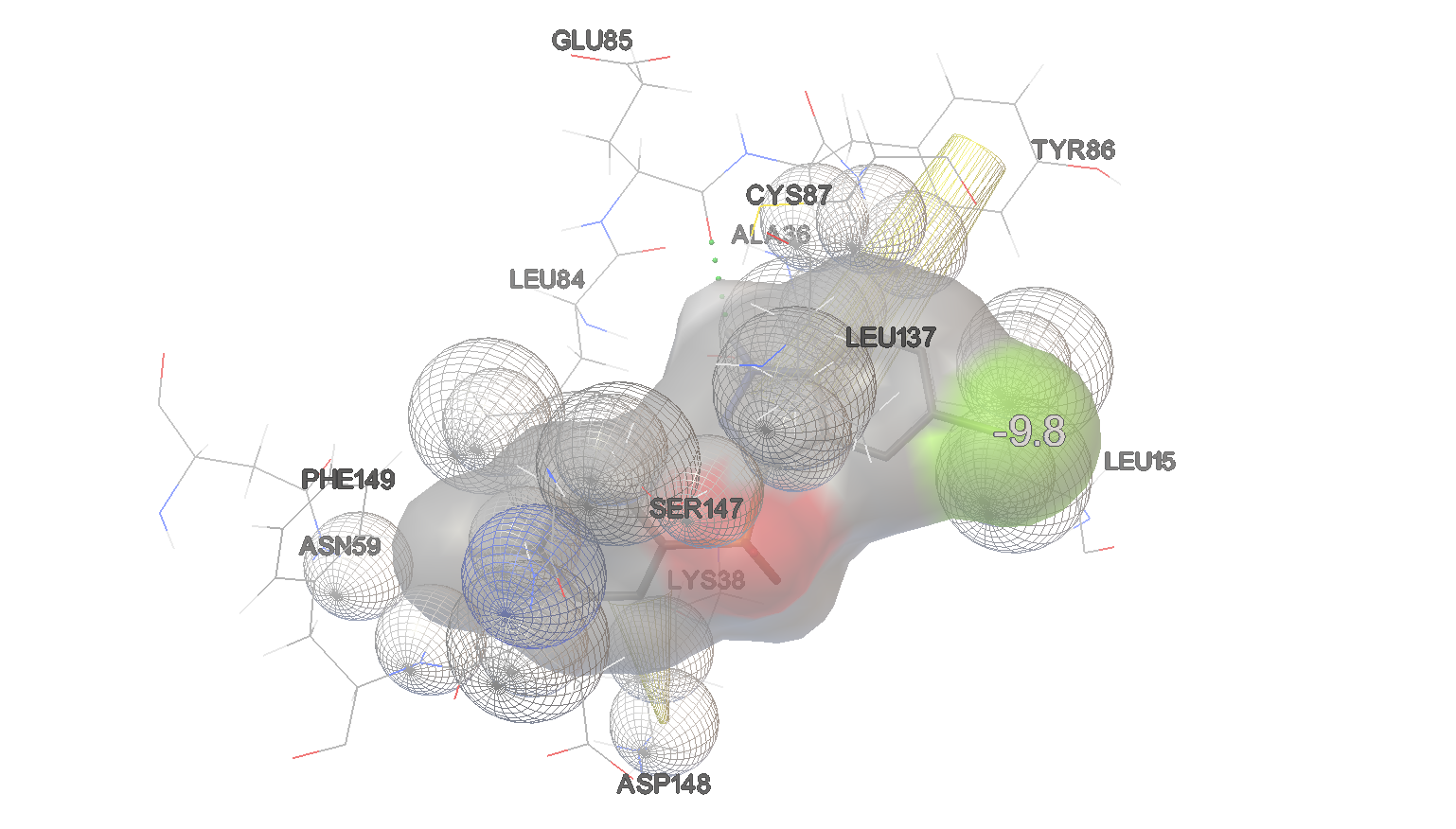


# **Fig. S17** Close contacts within the **2**‑**2e9n** complex (HB marked with dashes, π‑cation contact with Lys38, π‑π stacking contact with Tyr86; *MGLTools 1-5-6* program)


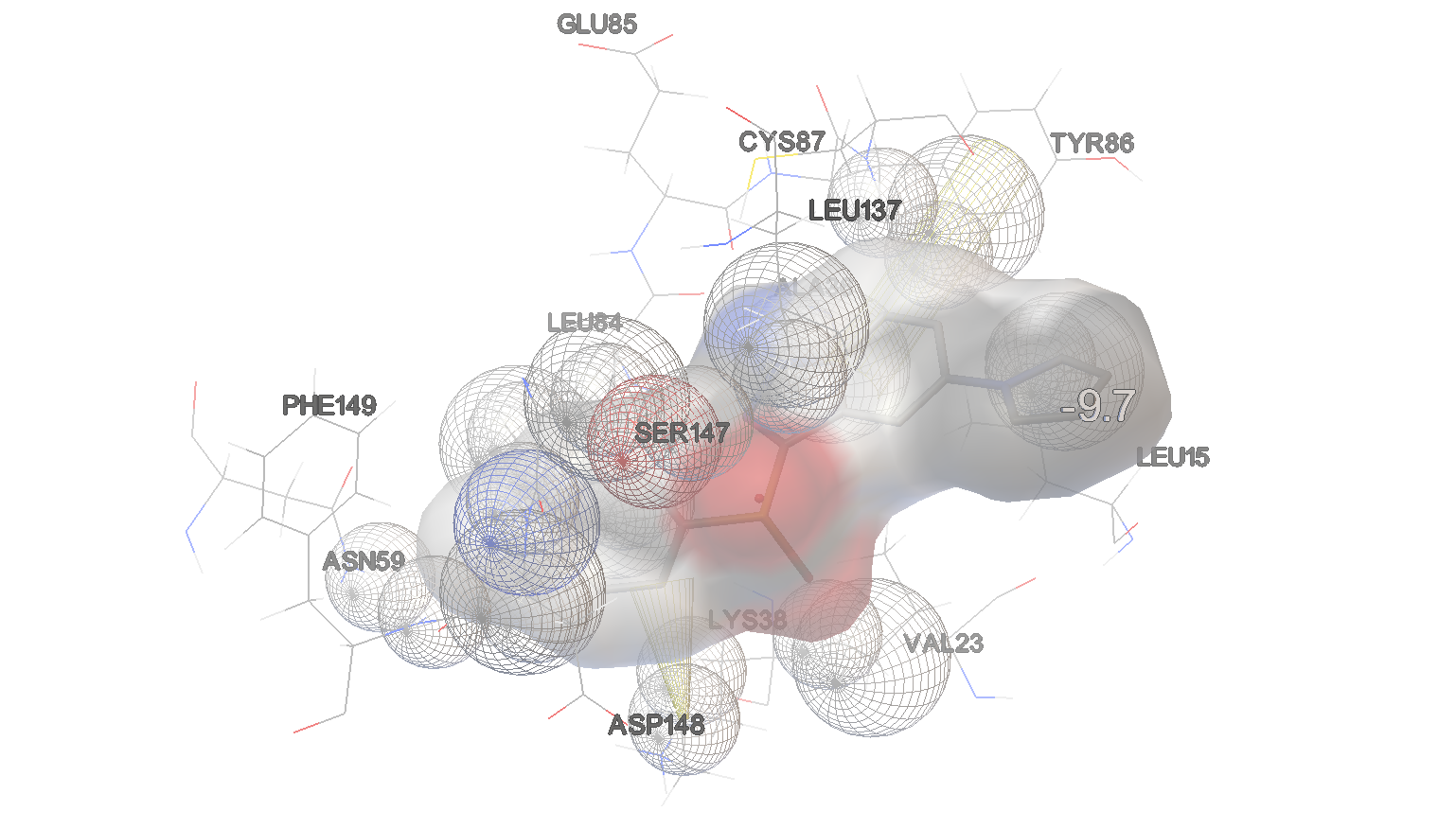


# **Fig. S18** Close contacts within the **3**‑**2e9n** complex (HB marked with dashes, π‑cation contact with Lys38, π‑π stacking contact with Tyr86; *MGLTools 1-5-6* program)


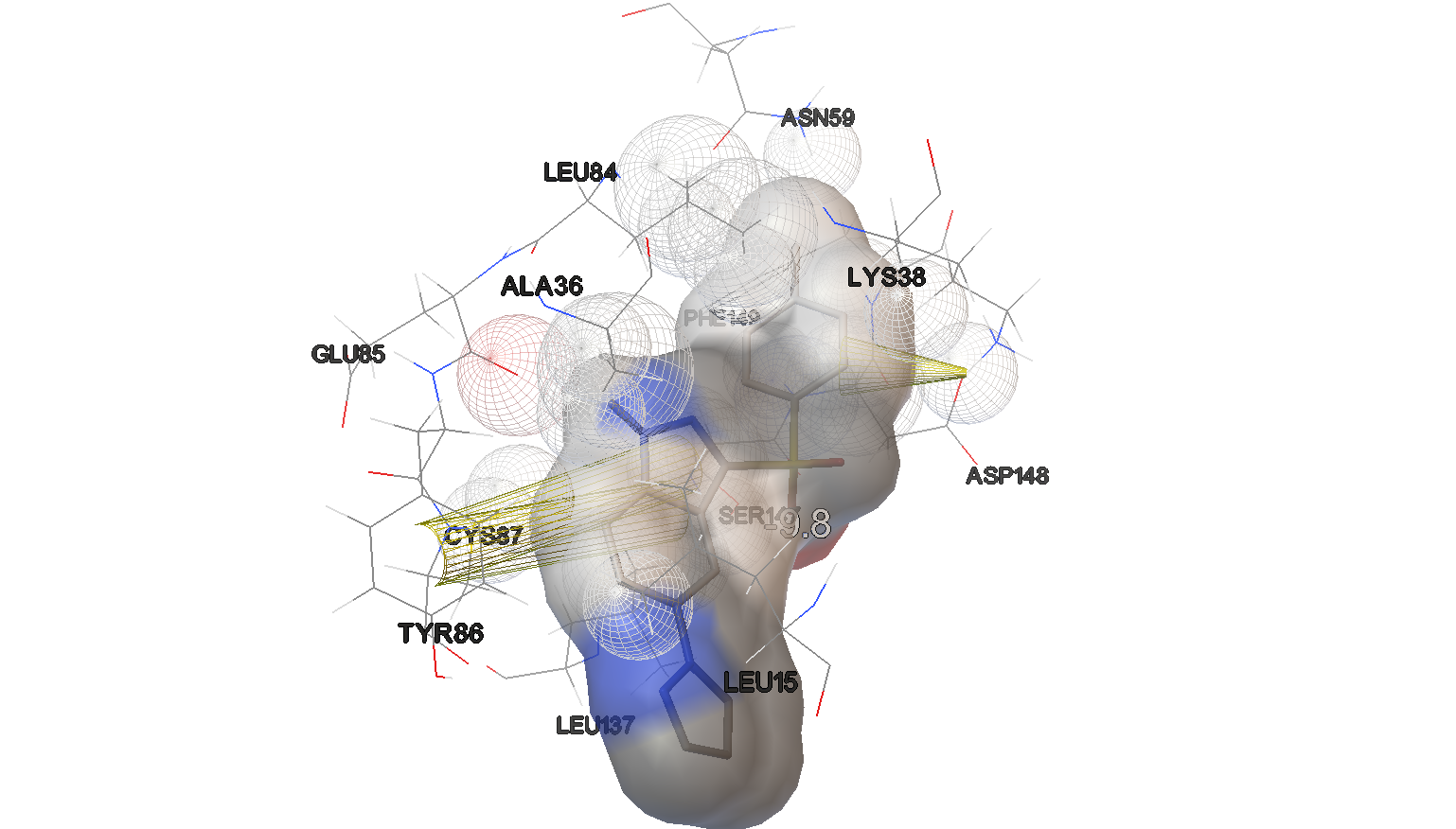


# **Fig. S19** Close contacts within the **4**‑**2e9n** complex (HB marked with dashes, π‑cation contact with Lys38, π‑π stacking contact with Tyr86; *MGLTools 1-5-6* program)


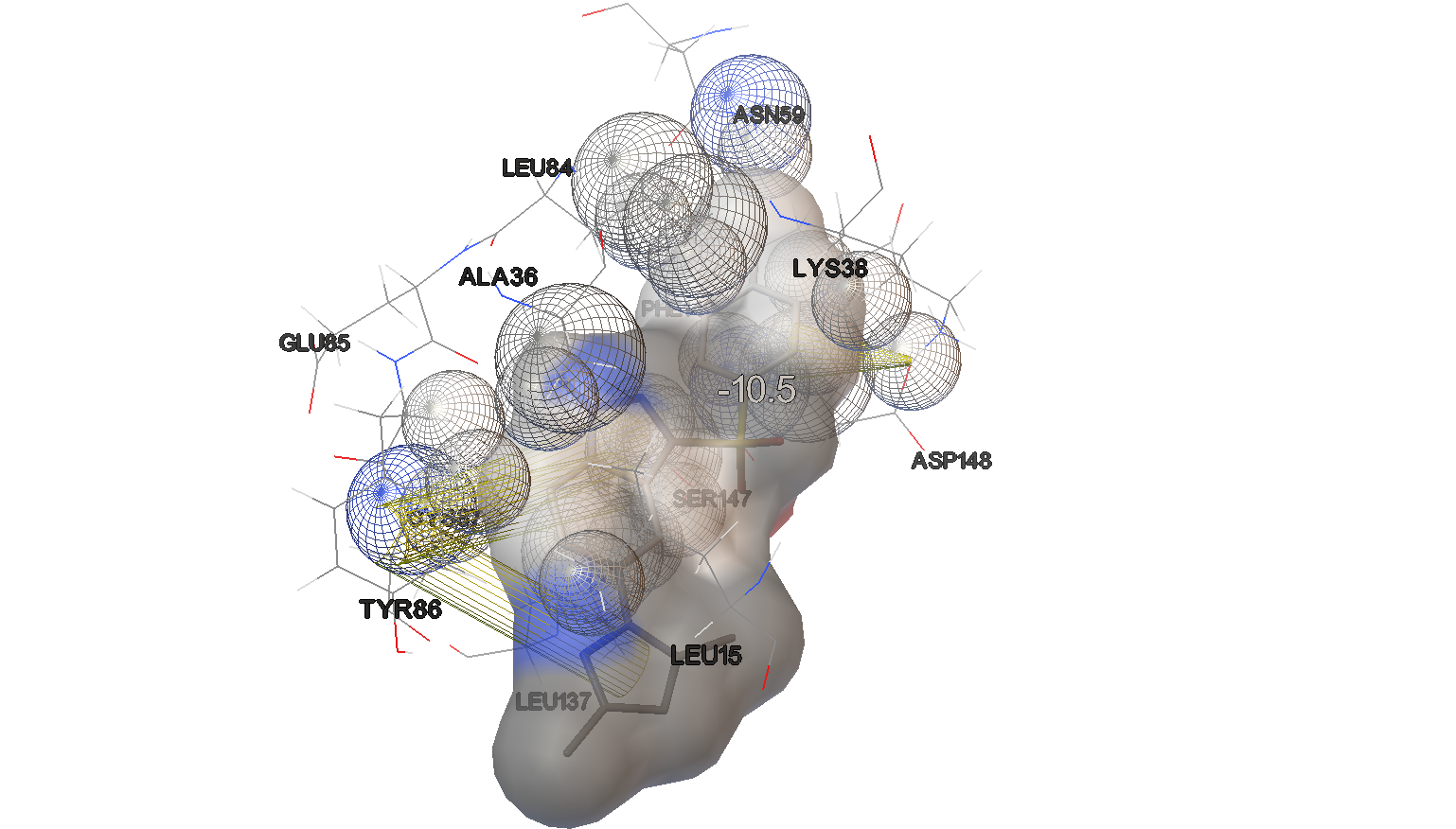


# **Fig. S20** Close contacts within the **5**‑**2e9n** complex (*MGLTools 1-5-6* program)


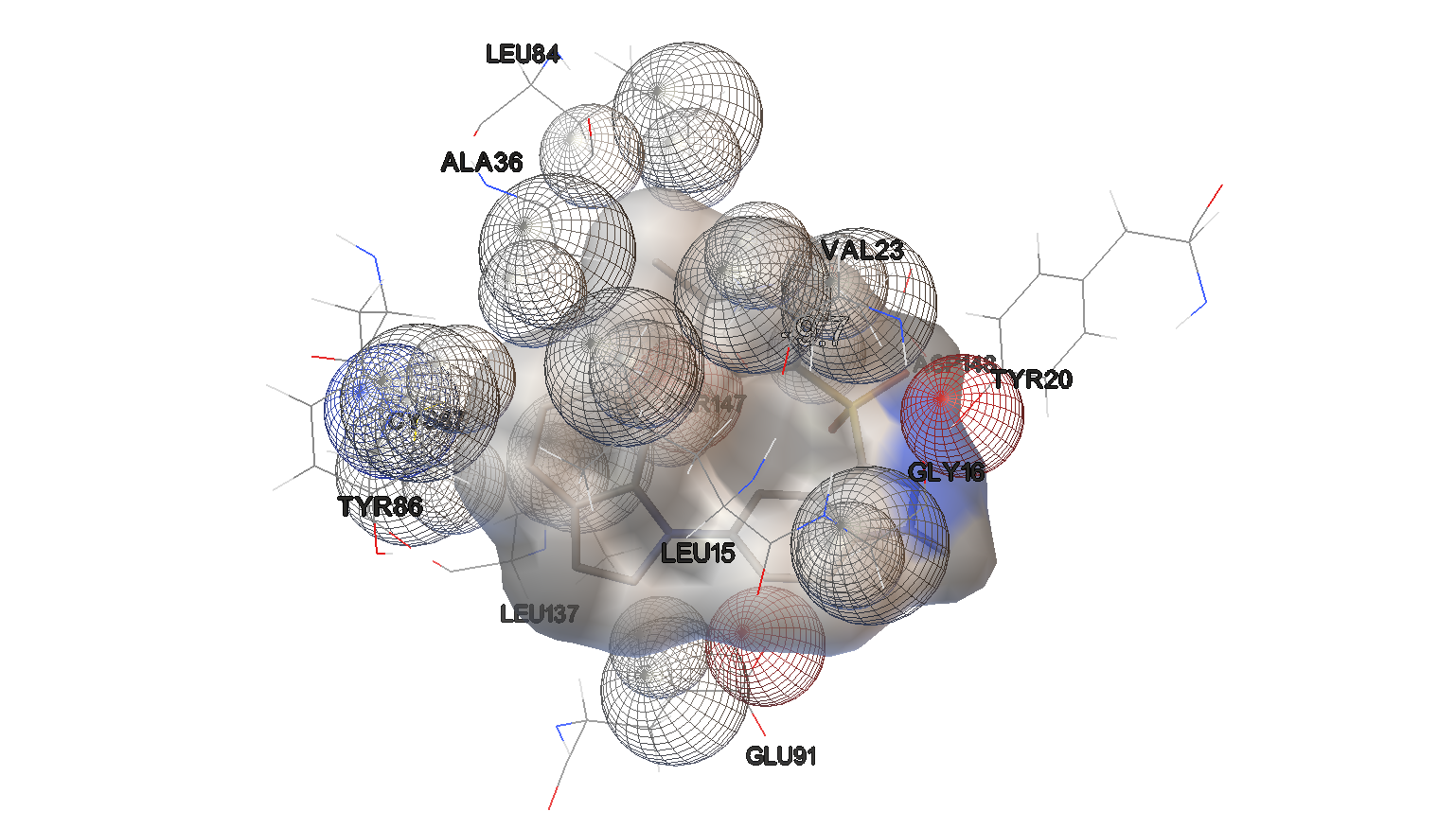


# **Fig. S21** Close contacts within the **6**‑**2e9n** complex (HB marked with dashes, π‑cation contact with Lys38;*MGLTools 1-5-6* program)


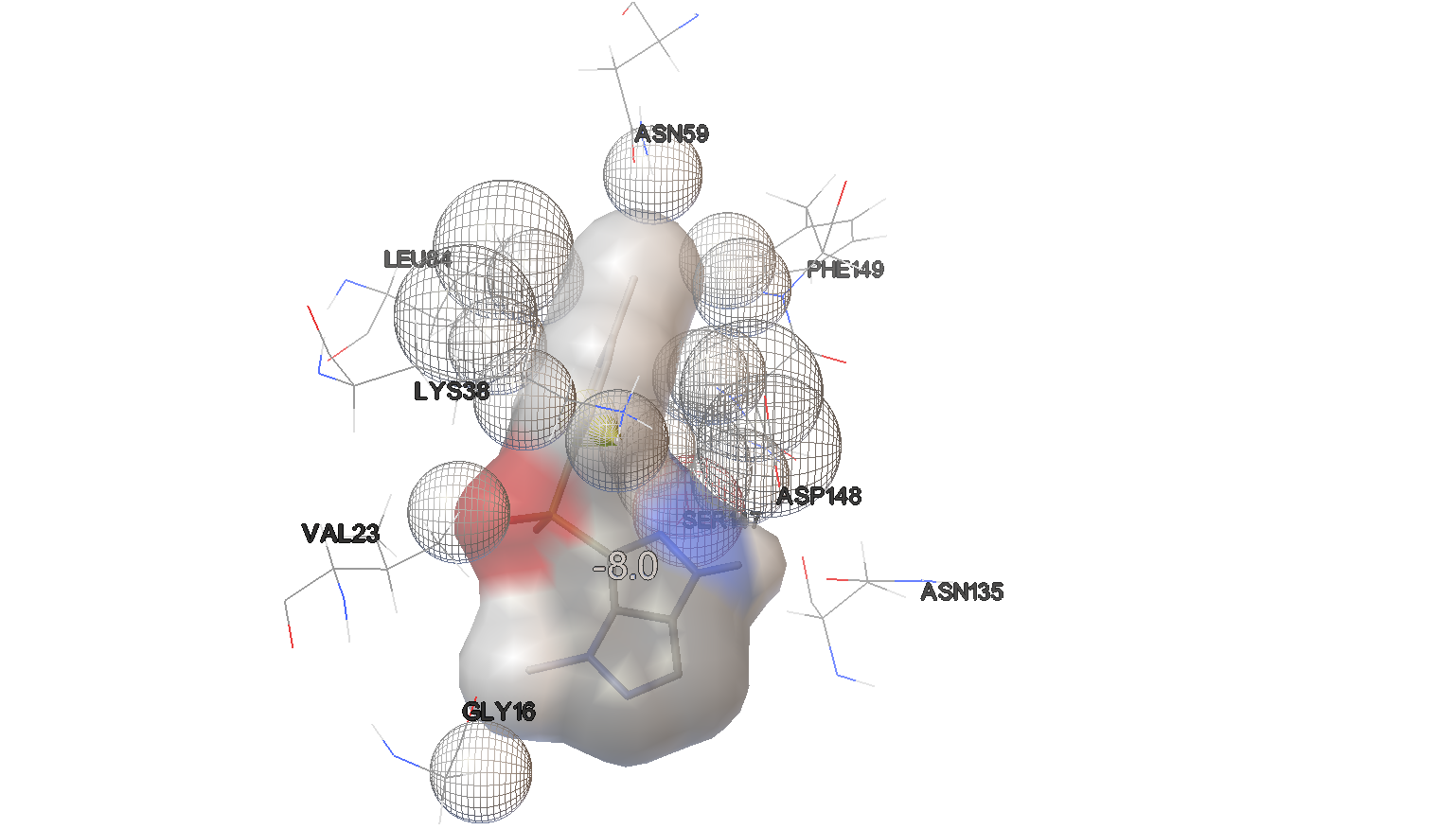


# **Fig. S22** Close contacts within the **7**‑**2e9n** complex (HB marked with dashes, π‑cation contact with Lys38, π‑π stacking contact with Tyr86; *MGLTools 1-5-6* program)


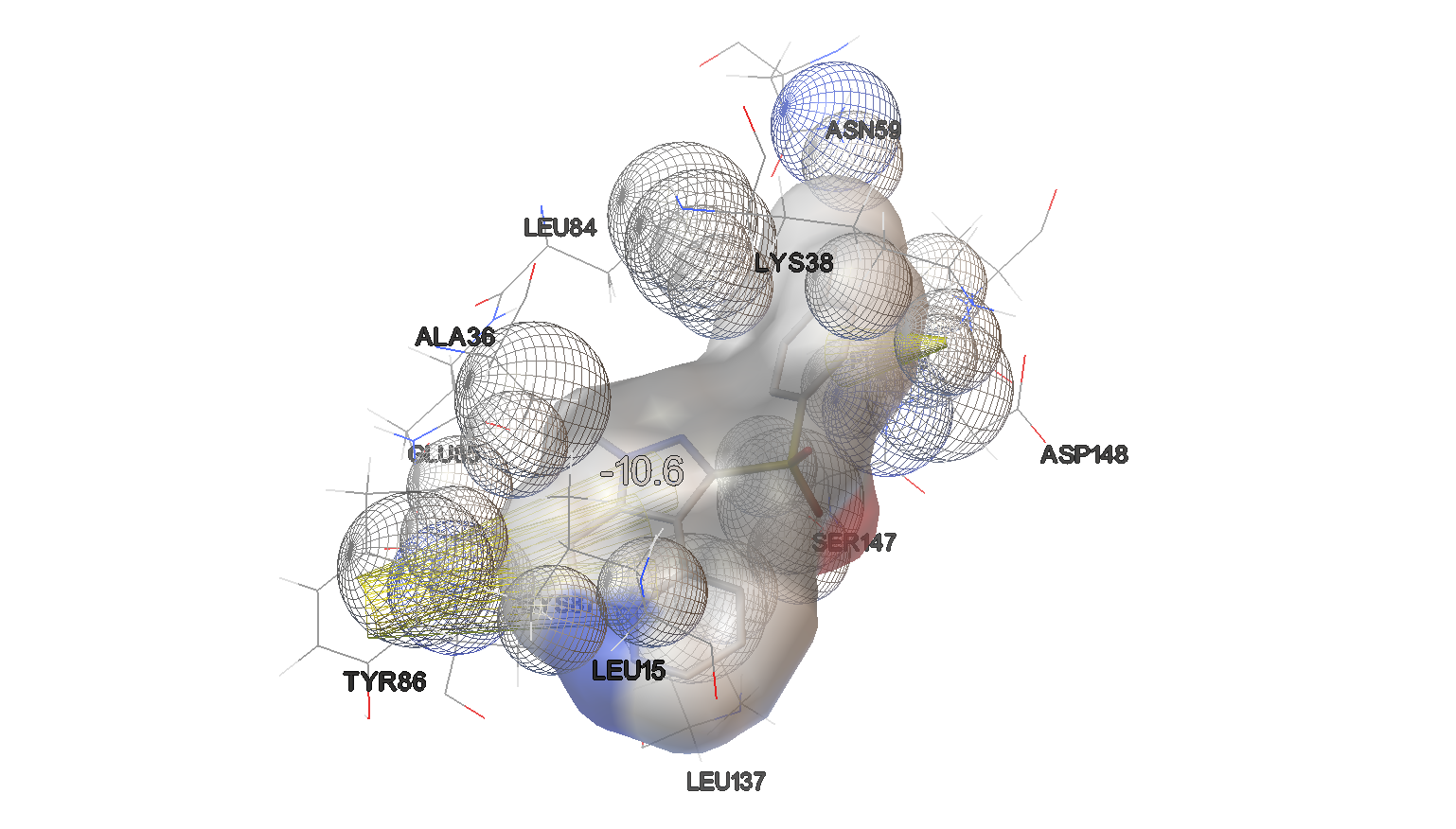


# **Fig. S23** HBs lifetime for **1** (black), **2** (red), **3** (green), **4** (blue), or **7** (yellow) and Glu85 resulted from the MD simulation.


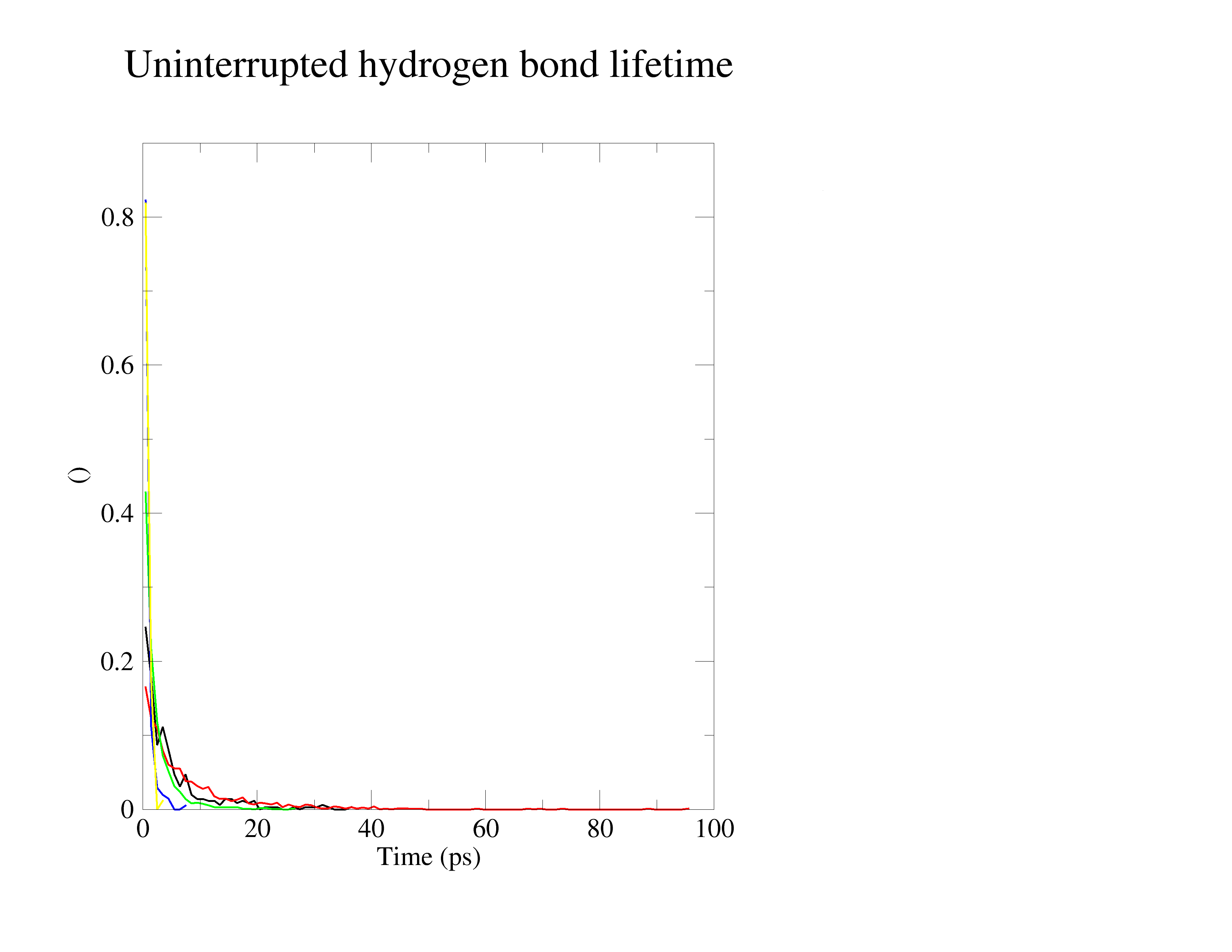


# **Fig. S24** HBs lifetime for the **4** (black) or **7** (red) and Ser147 resulted from the MD simulation.


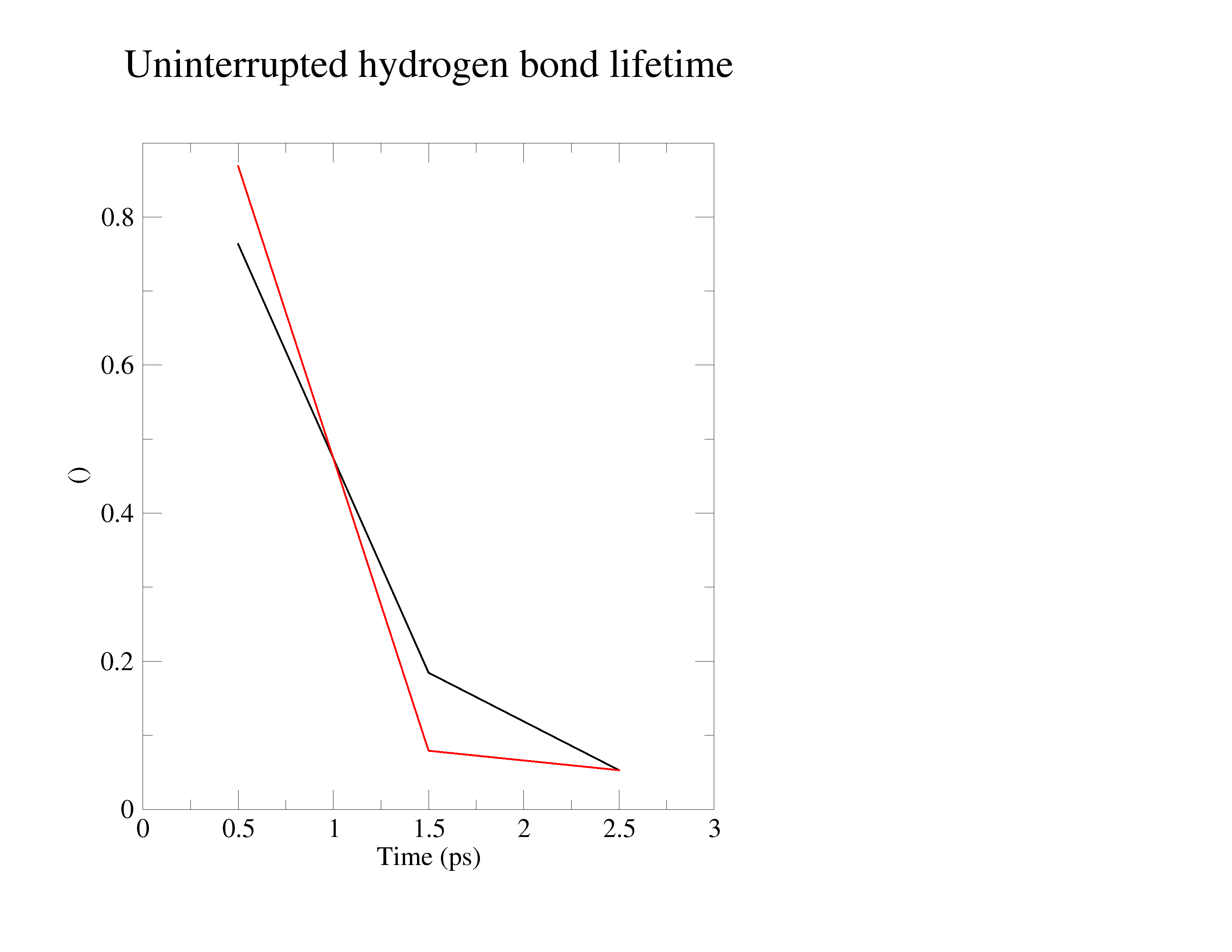


# **Fig. S25** HBs lifetime for the **5** and Tyr20 (black) or Glu17 (red) obtained from the MD simulation.


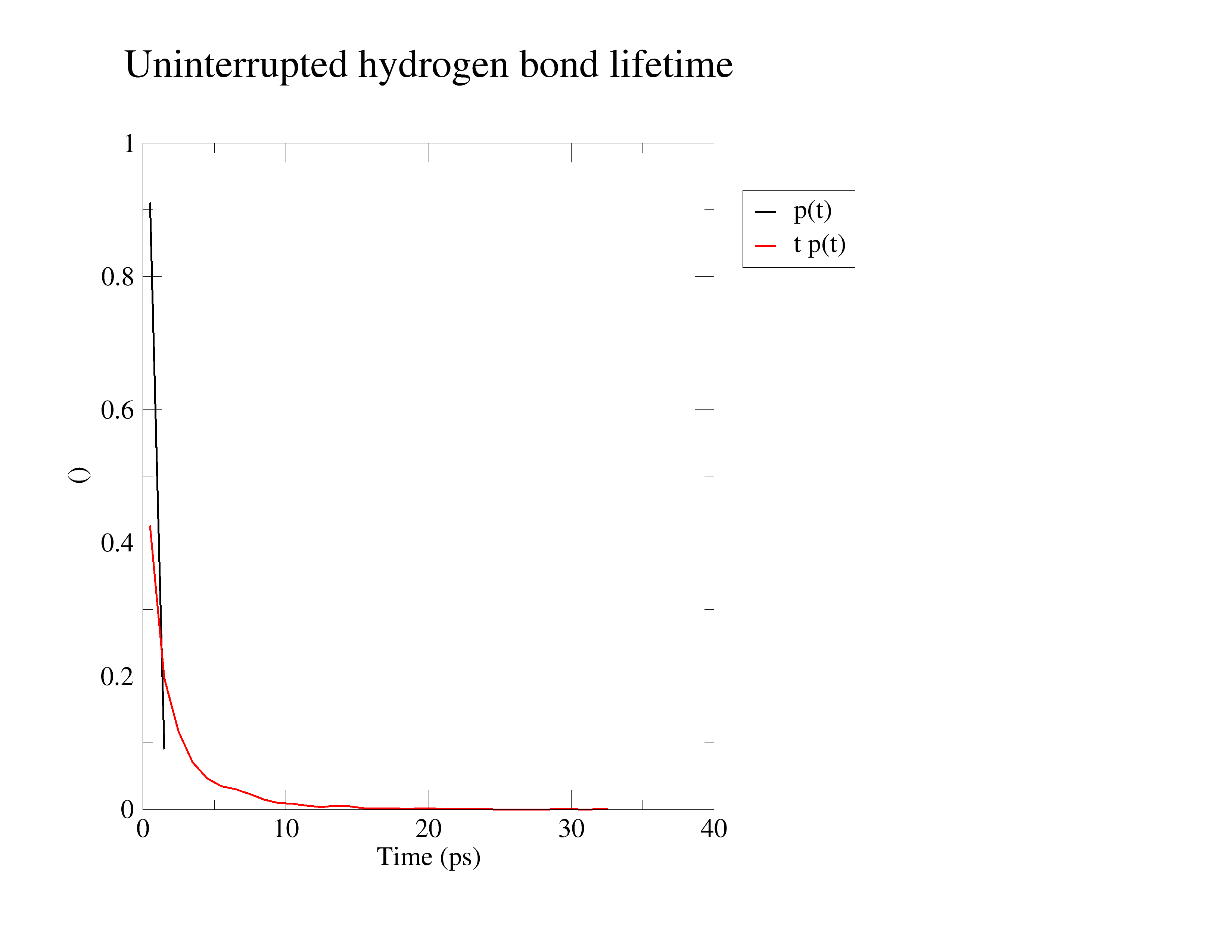

Supplement: Supplementary file 1 — (DOC 41358 kb) [file 894_2020_4407_MOESM1_ESM.doc]
